# Protection by vaccination of children against typhoid fever with a Vi-tetanus toxoid conjugate vaccine in urban Bangladesh: a cluster-randomised trial

**DOI:** 10.1016/S0140-6736(21)01124-7

**Published:** 2021-08-21

**Authors:** Firdausi Qadri, Farhana Khanam, Xinxue Liu, Katherine Theiss-Nyland, Prasanta Kumar Biswas, Amirul Islam Bhuiyan, Faisal Ahmmed, Rachel Colin-Jones, Nicola Smith, Susan Tonks, Merryn Voysey, Yama F Mujadidi, Olga Mazur, Nazmul Hasan Rajib, Md Ismail Hossen, Shams Uddin Ahmed, Arifuzzaman Khan, Nazia Rahman, Golap Babu, Melanie Greenland, Sarah Kelly, Mahzabeen Ireen, Kamrul Islam, Peter O'Reilly, Karin Sofia Scherrer, Virginia E Pitzer, Kathleen M Neuzil, K Zaman, Andrew J Pollard, John D Clemens

**Affiliations:** a International Centre for Diarrhoeal Disease Research, Bangladesh, Dhaka, Bangladesh; b Oxford Vaccine Group, Department of Paediatrics, University of Oxford, and the NIHR Oxford Biomedical Research Centre, Oxford, UK; c University of Maryland School of Medicine, Baltimore MD, USA; d Department of Epidemiology of Microbial Diseases, Yale School of Public Health, Yale University, New Haven, CT USA; e University of California Los Angeles, Fielding School of Public Health, Los Angeles, CA, USA

## Abstract

**Background:**

Typhoid fever remains a major cause of morbidity and mortality in low-income and middle-income countries. Vi-tetanus toxoid conjugate vaccine (Vi-TT) is recommended by WHO for implementation in high-burden countries, but there is little evidence about its ability to protect against clinical typhoid in such settings.

**Methods:**

We did a participant-masked and observer-masked cluster-randomised trial preceded by a safety pilot phase in an urban endemic setting in Dhaka, Bangladesh. 150 clusters, each with approximately 1350 residents, were randomly assigned (1:1) to either Vi-TT or SA 14-14-2 Japanese encephalitis (JE) vaccine. Children aged 9 months to less than 16 years were invited via parent or guardian to receive a single, parenteral dose of vaccine according to their cluster of residence. The study population was followed for an average of 17·1 months. Total and overall protection by Vi-TT against blood culture-confirmed typhoid were the primary endpoints assessed in the intention-to-treat population of vaccinees or all residents in the clusters. A subset of approximately 4800 participants was assessed with active surveillance for adverse events. The trial is registered at www.isrctn.com, ISRCTN11643110.

**Findings:**

41 344 children were vaccinated in April–May, 2018, with another 20 412 children vaccinated at catch-up vaccination campaigns between September and December, 2018, and April and May, 2019. The incidence of typhoid fever (cases per 100 000 person-years) was 635 in JE vaccinees and 96 in Vi-TT vaccinees (total Vi-TT protection 85%; 97·5% CI 76 to 91, p<0·0001). Total vaccine protection was consistent in different age groups, including children vaccinated at ages under 2 years (81%; 95% CI 39 to 94, p=0·0052). The incidence was 213 among all residents in the JE clusters and 93 in the Vi-TT clusters (overall Vi-TT protection 57%; 97·5% CI 43 to 68, p<0·0001). We did not observe significant indirect vaccine protection by Vi-TT (19%; 95% CI −12 to 41, p=0·20). The vaccines were well tolerated, and no serious adverse events judged to be vaccine-related were observed.

**Interpretation:**

Vi-TT provided protection against typhoid fever to children vaccinated between 9 months and less than 16 years. Longer-term follow-up will be needed to assess the duration of protection and the need for booster doses.

**Funding:**

The study was funded by the Bill & Melinda Gates Foundation.

## Introduction

Despite decades of worldwide effort to improve water quality, hygiene, and sanitation, the global burden of typhoid fever, caused by *Salmonella enterica* serovar Typhi (*S* Typhi), remains high. Analyses have estimated that more than 9 million cases and 110 000 deaths occur per year, with the burden concentrated among the poor residing in low-income and middle-income countries.^1^ Although typhoid was traditionally believed to be a disease of school-aged children, it is now known that preschool-aged children (aged 0–5 years) have a high incidence in areas of high overall typhoid incidence, such as urban slums in the developing world.^2^ Augmenting this burden are growing levels of antimicrobial resistance among clinical isolates of *S* Typhi.^3^ For these reasons, development and deployment of safe and effective typhoid vaccines that can protect young children are a global priority. Studies done in the urban slums of Dhaka city in Bangladesh, which is an area with high typhoid endemicity, have shown that the highest incidence is in children younger than 5 years^4^, ^5^ and studies in hospitalised children in Dhaka city have shown a higher detection rate in children 2 years of age and younger.^6^, ^7^ Active fever surveillance done in an urban slum of Dhaka city between 2003 and 2004 reported that the incidence of paratyphoid fever was 0·4/1000 person-years with an incidence of typhoid fever that is seven times higher.^5^

Older-generation, parenterally administered typhoid vaccines, consisting of killed typhoid organisms, were shown in some trials to provide substantial protection, but their use was limited by their reactogenicity.^8^ Next-generation vaccines, consisting of orally administered, genetically attenuated *S* Typhi organisms (strain Ty21a), or of parenterally administered Vi polysaccharide (ViPS, the outer capsule of *S* Typhi), were found to be safe and moderately protective.^8^, ^9^ However, ViPS was limited by protection lasting only 2–3 years, Ty21a was limited by a rigidly timed, multi-dose regimen, and both vaccines were limited by approved ages of administration of over 2 years for ViPS and over 5 years for Ty21a.^8^, ^9^

**Research in context**

**Evidence before this study**

Typhoid vaccines in which the Vi polysaccharide outer capsule of *Salmonella enterica* serovar Typhi is chemically conjugated to a carrier protein have generated considerable interest due to their ability to elicit T-cell-dependent antibody responses, and thus possible protection of infants and young children as well as immunological memory. A Vi-tetanus toxoid conjugate vaccine (Vi-TT) produced in India, which is the subject of the study report here, has been licensed and WHO-prequalified on the basis of trials in typhoid-endemic populations, which evaluated immunological endpoints, but did not evaluate clinical typhoid disease, and a volunteer typhoid challenge experiment in adults in the UK, where typhoid is not endemic. We searched PubMed for reports on randomised trials of the clinical efficacy of Vi-conjugate vaccines using the search terms “Vi polysaccharide vaccine”, “Vi-conjugate vaccine”, and “typhoid vaccine”. We found publications on results of an individually randomised efficacy trial of clinical protection by Vi-rEPA (recombinant *Pseudomonas aeruginosa* exoprotein A) vaccine in Vietnamese children aged 2–5 years given two injections 6 weeks apart, in which 91·5% (95% CI 77·1–96·6%; p<0·001) protection was detected over 27 months of follow-up. Furthermore, a school-based cluster-randomised trial of a two-dose regimen of Vi-TT vaccine (produced with technology different from the Vi-TT vaccine tested in the current trial) found 100% (95% CI 97·6–100%) protection, but this trial was limited by a small sample size of 1765 children. An individually randomised trial of the same Vi-TT vaccine evaluated in the current paper, given as a single dose to children aged 9 months to less than 16 years in Nepal, found 81·6% (95% CI 58·8–91·8; p<0·001) protection in an interim analysis covering 12 months follow-up after dosing.^14^ None of these trials was adequately powered to evaluate clinical protection of infants and children under 2 years of age, and none was designed to evaluate herd protection by a Vi-conjugate vaccine.

**Added value of this study**

Our trial confirms the findings of the earlier reported individually randomised trial in Nepal, in which a single dose of Vi-TT given to children aged 9 months to less than 16 years was safe and conferred approximately 80% protection against typhoid fever, and adds to the Nepal trial in three significant ways.^14^ First, our findings show that the degree of protection by Vi-TT in Nepal was also observed in a setting in which the burden of typhoid was even higher in children younger than 16 years, as reflected in an incidence of typhoid of 635 cases per 100 000 person-years in children who received the control vaccine. Secondly, our trial shows for the first time that high-grade, significant protection by a Vi-TT was achieved when vaccination was given to participants aged from 9 months to 2 years. This finding suggests that Vi-TT can be effectively incorporated into the Expanded Programme on Immunisation (EPI) schedule of immunisation of infants and young children. Thirdly, our trial indicates that considerable overall population protection is achievable when even moderate vaccine coverage is achieved and maintained in children younger than 16 years. However, significant herd protection by Vi-TT in non-recipients of Vi-TT was not observed.

**Implications of all the available evidence**

Taken together with the findings of the Nepal trial, our data confirm that Vi-TT, administered in a single dose, is safe and effective when given to children in settings with a high incidence of endemic typhoid and support efforts by WHO and the Global Alliance for Vaccines and Immunizations to roll out this vaccine in such settings. The finding of significant protection of infants and children under 2 years of age indicate that the Vi-TT can be effectively incorporated into the EPI schedule of immunisation of infants and young children. Finally, to date no study has done sufficiently long post-vaccination follow-up to measure long-term protection by Vi-conjugate vaccines against typhoid. Such data will be needed to fully measure the cost-effectiveness of these vaccines and to establish the need for and timing of booster doses.

Among new-generation vaccines against typhoid, ViPS-protein conjugate vaccines have been most promising, owing to their ability to elicit robust immune responses in infants and young children and their capacity to elicit T-cell-mediated immune memory responses, offering the potential for long-term protection.^10^

One such vaccine, consisting of ViPS conjugated to tetanus toxoid (Vi-TT; Typbar TCV, Bharat Biotech International, Hyderabad, India), has been licensed as a single-dose vaccine for people younger than 12 months in multiple countries (India, Nepal, Cambodia, and Nigeria),^11^ and has been prequalified by WHO for purchase by UN agencies.^12^ The WHO Strategic Advisory Group of Experts on Immunization has recommended that, for programmatic and epidemiological reasons, the vaccine should be started at 9 months of age, coincident with the 9 month Expanded Programme on Immunization visit in many countries. A phase 3 randomised, controlled trial has shown that the vaccine was safe and highly immunogenic (seroconversion, 98%; geometric mean titre, 1937 [95% CI 1785–2103]) in children (n=327) aged 6–23 months.^13^ This vaccine has also been shown in an interim analysis of an individually randomised efficacy trial among children in Nepal to confer 82% protection against typhoid disease.^14^ To better understand the ability of this vaccine to confer protection, including herd protection, under circumstances of mass immunisation, we did a cluster-randomised, controlled, clinical trial of Vi-TT vaccine in urban Bangladesh.

## Methods

### Study design and participants

We did a participant-masked and observer-masked, cluster-randomised trial in Mirpur, a densely populated urban area of Dhaka, Bangladesh using eight clinical facilities known to provide care for enteric fever to the catchment population (Mirpur Field Clinic; Suhrawardy Hospital; Radda Barnen, Mirpur-10; Adhunik Hospital, Mirpur; Shishu Hospital, Kalshi, Mirpur-11; Kurmitola General Hospital; Kingston Hospital, Mirpur; Islami Bank Hospital, Mirpur), which had previously been documented to have a high incidence of typhoid fever (manuscript submitted). Before the trial, we did a small, individually randomised pilot study that showed the safety of a single dose of the Vi-TT vaccine and the SA 14-14-2 Japanese encephalitis (JE) control vaccines in 200 children aged 9 months to less than 16 years in Dhaka. For the main trial, eligible children aged 9 months to less than 16 years residing in an area with a total population of 205 760 were assigned by geographical cluster to receive either a single dose of Vi-TT or the SA 14-14-2 vaccine. The clusters were then followed-up after dosing to detect episodes of typhoid and paratyphoid fever.

We did a baseline census of the entire population of the study area, excluding people who were not permanent residents and those who planned to move away within the next month, between Feb 14 and March 25, 2018, to collect individual-level and household-level demographic, socioeconomic, water–sanitation–hygiene, and geo-positioning information, after obtaining informed consent from participants. For this purpose, a household was defined as people who shared the same cooking pot. Each household and individual were given a unique study number, and each household was given a household card providing basic information about each household member. This population was divided into 150 geographical clusters of contiguous households with approximately equal total populations, by means of natural borders wherever possible (appendix p 5). The census was subsequently updated at approximately 6-month intervals to capture all births, deaths, and migrations.^15^

The trial received ethical approval from the Research and Ethical Review Committees of the International Centre for Diarrhoeal Disease Research, Bangladesh (icddr,b), as well as the institutional review boards of Oxford University, Oxford, UK and University of Maryland, Baltimore, MD, USA. Written informed consent was obtained from all participants, including parent or guardian consent for all those younger than 16 years and participant assent for those aged 11 to less than 16 years. Full details of the study protocol and analysis plan are available in the appendix.

### Randomisation and masking

Each cluster was allocated at random in a 1:1 ratio to receive either Vi-TT or SA 14-14-2 vaccine, with 75 clusters in each group, blocked within 12 prespecified strata. We stratified allocation by the three geographical wards of residence, the distance from the midpoint of the cluster to the nearest surveillance health-care facilities (above the median *vs* at or below the median), and the number of children aged 9 months to less than 16 years in the cluster (above the median *vs* at or below the median). We generated approximately 3000 randomisation lists using stratified block randomisation to identify 1000 randomisation lists meeting the condition of 75 clusters in each group and then randomly selected one as the final randomisation list.

After obtaining informed consent, participants were screened for inclusion and exclusion criteria. If they were found to be eligible for vaccination, the randomisation list, which was computer generated and prepared by the external study statistician, was consulted to allocate the vaccine. The vaccinator then administered the vaccine and advised the participants to wait for 15 min at the vaccination sites. Age-eligible, consenting, non-pregnant residents were offered a single dose of assigned vaccine after excluding people with known allergy to or previous receipt of either of the vaccines; and people with fever, those who had received antipyretics in the past 4 h, and those administered another vaccine in the last 30 days (temporary exclusion criteria). We took several steps to keep participants and observers masked to the assigned and received study agent. First, assessment of eligibility and acquisition of informed consent were done without knowledge of the assigned agent. Secondly, randomisation and vaccine preparation were completed by staff members who were physically shielded by a curtain from the participant. Thirdly, although vaccinators were aware of the agent administered, they were instructed not to inform participants; and fourthly, all people involved in post-vaccination follow-up for assessment of vaccine safety, immunogenicity, and protection were kept unaware of vaccine assignment.

### Procedures

We evaluated the safety, immunogenicity, and protection conferred by a single dose of Vi-TT vaccine, in which ViPS (25 μg) was chemically conjugated to tetanus toxoid. The control agent, also given as a single dose to the same age group, was SA 14-14-2, live attenuated JE vaccine (Chengdu Institute of Biological Products, Chengdu, China). Both vaccines were supplied in their commercial presentations as five-dose vials and stored at 2–8°C. Vi-TT vaccine was given as an intramuscular injection, whereas SA 14-14-2 vaccine was given subcutaneously. Children aged 9 months to less than 16 years were targeted for vaccination. We inoculated both vaccines at the same anatomical location depending on the ages of the children (area of anterolateral aspect of the left thigh for under 2 years old and area of deltoid muscle in upper left arm for over 2 years old) to help maintain the masking.

All participants were requested to remain in the vaccination site for 15 min after vaccination to detect and manage acute events. A subset of approximately 4800 participants at the baseline vaccination campaign, selected in approximately equal numbers from each of the 150 clusters and stratified by age at vaccination (<5 years *vs* older), were given diary cards to record adverse events daily post-vaccination, to help recall symptoms and duration for the active surveillance of adverse events. As part of active surveillance follow-up, parents or guardians of participants were contacted by telephone at 7 days post-vaccination to ascertain both solicited and unsolicited adverse events. In addition, parents or guardians of all participants not in the subset were encouraged to go to the Adverse Event Monitoring Cell at the Mirpur Field Clinic where a medical doctor was available from 0830 h to 2100 h and on call 24 h a day for adverse event monitoring throughout the whole vaccination period and 7 days thereafter. In addition, surveillance for severe adverse events was also done throughout the post-vaccination period of the trial for all participants by study physicians at all clinical sites used for surveillance for enteric fever (see below), via queries of participants during the 6-monthly census follow-ups, and every 2 weeks visits to all households by community health workers. Verbal autopsies were done for deaths detected in the censuses and in the community health worker visits. For these verbal autopsies, information obtained by the trained study staff was further investigated and corroborated by study physicians, who assigned a cause of death. Structured data forms were used for all of these activities. All adverse events were graded as severe, moderate, or mild, as were events classified serious adverse events, and severity was judged by a clinician, as observed by the investigator, members of the study team, or reported by the parent or guardian.^16^

Approximately 1500 vaccinees were enrolled in an immunogenicity subcohort. Participants from 18 randomly selected clusters, yielding an approximate 2:1 ratio of Vi-TT to SA 14-14-2 recipients, were randomly selected in an age-stratified manner (<5 years *vs* older with an allocation ratio of 1:1) by an independent, unmasked statistician. These participants were invited to contribute 3–5 mL of blood just before dosing and at 28 days, 18 months, and 24 months after dosing. Anti-Vi IgG titres in plasma were assessed by means of a commercial enzyme-linked immunosorbent assay kit (VaccZyme, The Binding Site, Birmingham, UK).

We initiated surveillance for enteric fever from Feb 26, 2018. We used a common protocol at all eight clinical facilities (see appendix p 5). Community health workers visited the homes of the entire population every 2 weeks to encourage people to seek care for febrile illness at one of the study facilities. Participants from the study area presenting with a history of 2 or more days of fever or with an axillary temperature of 38°C or more had clinical findings systematically recorded by a study physician, and blood specimens (3 mL blood from participants aged ≤17 years old and 5 mL from participants aged >17 years old) were collected in bottles supplied by the company for culture after giving informed consent. The identities of patients were confirmed at the time of presentation for care with use of household identity cards distributed during the censuses, or for those without such cards, identities were confirmed by means of computerised censuses on electronic tablets available at each clinical site. Blood specimens were transported on the day of collection to laboratories at icddr,b, where culture was carried out by means of a standard automated BacT/ALERT method (3 mL blood in paediatric fastidious antimicrobial neutralisation bottles from children aged 0–10 years; 3 mL blood in fastidious antimicrobial neutralization (FAN) bottles from children aged 11–17 years; 5 mL blood in FAN bottles from others were collected for culture); after getting positive signals from the blood culture machine, subculture was done on MacConkey agar, blood agar, and chocolate agar plates. Salmonella isolates were identified on the basis of colony morphology, microscopic examination of Gram stained smear, biochemical tests, and by slide agglutination tests with *Salmonella*-specific anti-sera (Denka Sieken, Tokyo, Japan). *Salmonella* polyvalent (group A-S), *Salmonella* group D, *Salmonella* H-d, and *Salmonella* Vi antisera were used for identification of *S* Typhi. To identify *S* Paratyphi A, *Salmonella* polyvalent (group A-S), *Salmonella* group A, and *Salmonella* H-a antisera were used. Antimicrobial susceptibility profiles were assessed by the disk diffusion method and the resistance pattern was established following the Clinical and Laboratory Standards Institute guidelines.^17^ Treatment of patients was started on the basis of physicians' discretion and was modified after getting the blood culture and sensitivity report if needed. Whenever *S* Typhi or *S* Paratyphi A was isolated, a team was dispatched within 14 days of isolation to the stated home of the participant to confirm that the person whose name was given at the surveillance site had sought care at the site on the date of presentation, and to ensure that clinical progress was satisfactory, including that antibiotic treatment was appropriate to the antimicrobial sensitivity patterns of blood culture isolates. Febrile visits in which the onset of fever was less than 14 days after discharge from the previous febrile visit were concatenated into a single febrile episode. Febrile episodes in which one or more blood cultures were positive for *S* Typhi were defined as typhoid or for *S* Paratyphi A were defined as paratyphoid fever sodes, and the onset of fever before the first visit of the episode was taken as the date of disease onset. Only episodes in which domiciliary visits confirmed patients' identities were eligible for inclusion in analyses of vaccine protection.

### Outcomes

Primary and secondary analyses were prespecified and were undertaken only after formal locking of the dataset. The primary analyses assessed total vaccine protection by Vi-TT against typhoid (protection of vaccinees owing to the direct immunising effect of the vaccine as well as reduction of transmission due to proximity to other vaccinees) and overall vaccine protection by Vi-TT against typhoid (protection of an entire population by vaccination of a targeted group of the population, including both vaccinees and non-vaccinees, owing to direct and indirect vaccine effects). Secondary analyses were assessment of vaccine safety; vaccine immune responses; indirect vaccine protection by Vi-TT against typhoid (protection of non-vaccinees owing to reduction of transmission due to proximity to vaccinees); and total protection by Vi-TT against paratyphoid fever.

### Statistical analysis

The study protocol originally specified that primary analyses would target the inner 80% of the population of each cluster and would consider 24 months of surveillance after baseline. A sample size of 187 500 residents with two groups of 75 equal-sized clusters was calculated accordingly (available in the study protocol). Before locking and unmasking of the data, and with consent of the Data Safety and Monitoring Boards (DSMBs), we decided to analyse the entire populations of the clusters, as these were the units originally allocated to the two study groups, and to restrict our analyses to episodes and person-time up to the 18-month census update, because the COVID-19 pandemic forced cessation of all study activities in Dhaka, including doing a final study census at 24 months of follow-up.

Because the study population had high migration rates and because we had adopted a strategy of catch-up vaccinations every 6 months after baseline, it was necessary to evaluate vaccine protection in a dynamic population. We defined the start date of residence as the midpoint of the baseline vaccination campaign (April 30, 2018) for non-vaccinees living in the study area at that time, the date of vaccination for individuals vaccinated at baseline, or the date of first migration into or birth in the study area for in-migrants and births post-baseline. Total vaccine protection was assessed by comparing the incidence of disease in recipients of Vi-TT vaccine versus recipients of SA 14-14-2 vaccine, taking the start date for counting events as the day after vaccination. Overall vaccine protection was evaluated by comparing the incidence in all residents of the Vi-TT clusters versus all residents of the SA 14-14-2 clusters, taking the start date for counting events as the day after the start date of residence. Indirect vaccine protection was assessed by comparing the incidence of typhoid in non-recipients of Vi-TT in the Vi-TT clusters versus non-recipients of SA 14-14-2 in the SA 14-14-2 clusters, taking the start date for counting events as the day after the start date of residence. We analysed individuals according to a first in, first out rule, including only individuals from the time of their first residence in a study cluster and censoring their follow-up based on the earliest of the date of death, the end of surveillance, or movement out of the study cluster in which they were first captured in this study (regardless of destination of that movement). For estimation of indirect vaccine protection of initially unvaccinated children who later became vaccinated, an additional censoring criterion was the date of vaccination.

Inter-group comparisons of the occurrence of adverse events and plasma immune responses were statistically evaluated with χ^2^ tests (or Fisher's exact test for sparse data) for categorical data and Student's *t* test (or the Mann-Whitney *U* test when parametric assumptions were not satisfied) for dimensional data. To evaluate vaccine protection against typhoid fever in simple analyses, we compared time to event using Kaplan-Meier survival curves, evaluated statistically with the log-rank test.^18^ To account for the design effect of cluster randomisation and to control for prespecified covariates at baseline (in addition to those used to define strata for randomisation), we fitted mixed-effects Poisson regression models with a random intercept for each cluster to estimate the incidence rate ratio (IRR).^19^ The details of model fitting can be found in the statistical analysis plan. Vaccine protection was calculated as (1 − IRR) × 100%. We did subgroup analyses in different age groups using the same mixed-effects Poisson regression models. For the two primary analyses, p<0·025 (two-tailed) was taken as the threshold of significance; p<0·05 (two-tailed) was used for other analyses. Intracluster correlation coefficients (ICCs) were calculated among control vaccine clusters by means of ANOVA.^20^ All the statistical analyses were carried out by means of R version 3.6.2 (2019-12-12);^21^ the glmmTMB package was used to fit the mixed-effects Poisson regression models,^22^ and the ICC was estimated by means of the ICCbin package.^23^ The trial was overseen by a local DSMB convened by icddr,b as well as an international DSMB convened by the University of Maryland. The trial is registered at www.isrctn.com, ISRCTN11643110 and is ongoing.

### Role of the funding source

The funder of the study had no role in study design, data collection, data analysis, data interpretation, or writing of the report.

## Results

**Figure 1:**
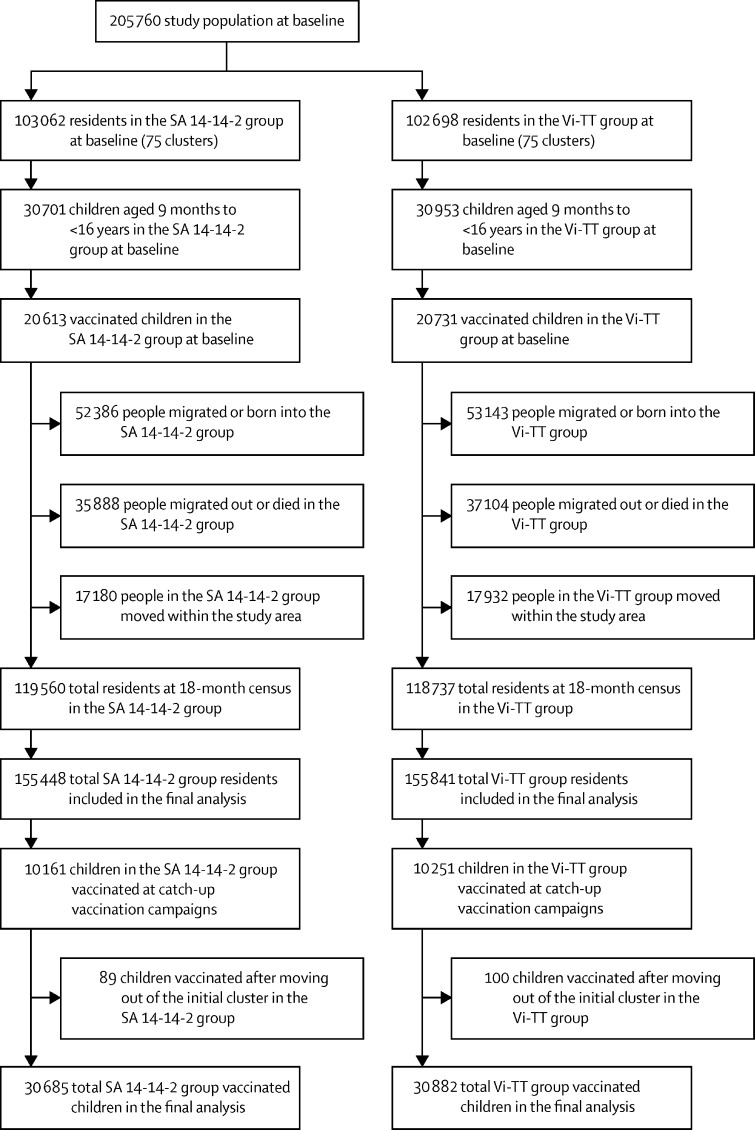
Trial profile SA 14-14-2=Japanese encephalitis vaccine. Vi-TT=Vi-tetanus toxoid conjugate vaccine.

Baseline immunisation was done between April 15 and May 15, 2018. Because of the high migration rate of the study population and the need to maintain stable coverage after baseline, we did catch-up vaccination of children who had not been dosed in earlier rounds at approximately 6-month intervals between September and December, 2018, and April and May, 2019. At baseline, there were 205 760 residents in the study area, including 61 654 children aged 9 months to less than 16 years old who were age-eligible for vaccination. 41 344 age-eligible children were vaccinated in April–May, 2018, with another 20 412 children vaccinated at catch-up vaccination campaigns between September and December, 2018, and April and May, 2019. During an average of 17·1 months of follow-up of the study population, there were 105 529 births and in-migrations, whereas the number of deaths were 1146 and out-migrations were 71 846 (figure 1). 12 348 (12 266 in the initial cluster of residence) children were vaccinated in the 6-month and 8064 (7957 in the initial cluster of residence) were vaccinated in the 12-month catch-up vaccination campaigns, maintaining an average vaccine coverage of children aged 9 months to less than 16 years of 64% during the follow-up period, nearly identical for the Vi-TT and SA 14-14-2 vaccine groups (appendix p 5).

**Figure 2:**
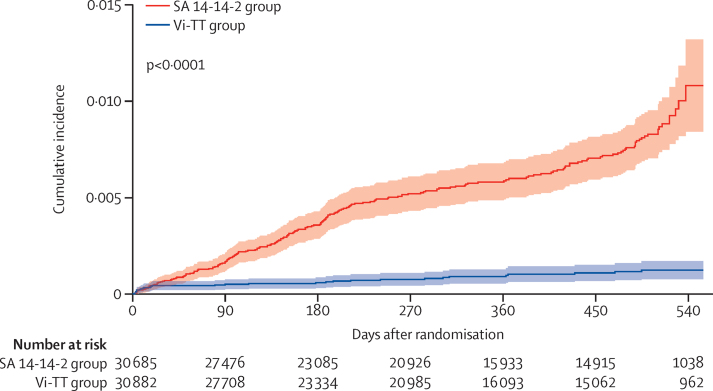
The cumulative incidence of blood culture-confirmed typhoid fever among vaccinees by treatment group SA 14-14-2=Japanese encephalitis vaccine. Vi-TT=Vi-tetanus toxoid conjugate vaccine.

Table 1 and the appendix (pp 1–3) show that the groups under comparison for assessment of overall, total, and indirect Vi-TT protection against typhoid fever were well balanced with respect to individual-level, household-level, and cluster-level features at the onset of follow-up. During follow-up of the 311 289 individuals assessed for overall vaccine protection, 475 episodes of typhoid were diagnosed (including 118 [24·8%] multidrug-resistant [resistant to three first-line antibiotics—chloramphenicol, ampicillin, and cotrimoxazole], 47 [9·9%] ciprofloxacin resistant, and 0 ceftriaxone resistant) typhoid strains; 221 and 254 episodes were detected among 61 567 vaccinees and 269 945 non-vaccinees for whom total and indirect vaccine protection were evaluated. Time to onset of typhoid for analysis of total Vi-TT protection against typhoid is shown in figure 2; times to onset for analyses of overall and indirect Vi-TT protection are shown in the appendix (pp 6–7). For the analysis of total vaccine protection (figure 2), the curves show a substantial and progressive divergence over time (p<0·0001). A less striking, but also progressive divergence was seen for the analysis of overall vaccine protection (p<0·0001; appendix p 6); little divergence of the curves was observed in the analysis of indirect vaccine protection (appendix p 7).

**Table 1:** Baseline characteristics

		**SA 14-14-2 group**	**Vi-TT group**
**Individual-level features**			
All residents		155 448	155 841
Age, years at residence		24·6 (12·3–36·4)	24·3 (12·2-36·3)
Sex			
	Male	77 222 (49·7%)	77 389 (49·7%)
	Female	78 226 (50·3%)	78 452 (50·3%)
Ward of residence			
	2	59 694 (38·4%)	63 083 (40·5%)
	3	36 436 (23·4%)	41 706 (26·8%)
	5	59 318 (38·2%)	51 052 (32·8%)
Formal education			
	Yes	35 424 (22·8%)	35 322 (22·7%)
	No	103 372 (66·5%)	103 112 (66·2%)
	Missing	16 652 (10·7%)	17 407 (11·2%)
Religion			
	Muslim	153 724 (98·9%)	153 696 (98·6%)
	Others	1724 (1·1%)	2145 (1·4%)
**Household level features**			
Number of households		38 621	38 613
Toilet type in the house			
	Flush toilet	2104 (5·4%)	2297 (5·9%)
	Others	36 517 (94·6%)	36 316 (94·1%)
Household drinking water source			
	Own water source	10 760 (27·9%)	9846 (25·5%)
	Others	27 861 (72·1%)	28 767 (74·5%)
Household boiling or filtering of drinking water			
	Yes	28 963 (75·0%)	28 284 (73·2%)
	No	9652 (25·0%)	10 328 (26·7%)
	Missing	6 (<1%)	1 (<1%)
Handwashing before meals			
	Yes	28 486 (73·8%)	29 076 (75·3%)
	No	10 135 (26·2%)	9537 (24·7%)
Handwashing after defecation			
	Yes	37 678 (97·6%)	37 653 (97·5%)
	No	943 (2·4%)	960 (2·5%)
Household monthly saving in Bangladeshi taka		0 (0–0) n=38 011	0 (0–0) n=38 091
Household monthly expenditure in Bangladeshi taka		14 500 (11 000–19 500; [n=38 546])	14 500 (11 000–19 900; [n=38 605])
**Cluster level features**			
Number of clusters		75	75
Number of households		515 (82·5)	515 (76·3)
Number of residents		2073 (259·5)	2078 (257·7)
Number of total age-eligible children for vaccination		647 (82·5)	655 (98·0)
Number of vaccinees		409 (54·8)	412 (59·0)
Age of cluster residents, years		26·0 (0·8)	25·9 (0·8)
Distance of midpoint of cluster to nearest study clinics, m		402 (274–518)	393 (250–551)
	At or below median	37 (49·3%)	38 (50·7%)
	Above median	38 (50·7%)	37 (49·3%)
Number of eligible children at baseline		407 (377–436)	408 (378–442)
	At or below median	38 (50·7%)	37 (49·3%)
	Above median	37 (49·3%)	38 (50·7%)
Ward location			
	2	28 (37·3%)	30 (40·0%)
	3	18 (24·0%)	19 (25·3%)
	5	29 (38·7%)	26 (34·7%)

Data are median (IQR), mean (SD), or n (%). SA 14-14-2=Japanese encephalitis vaccine. Vi-TT=Vi-tetanus toxoid conjugate vaccine.

The incidence rate difference between SA 14-14-2 and Vi-TT groups was 539 (95% CI 443 to 635, p<0·0001) per 100 000 person-years among vaccinees, and 120 (95% CI 92 to 147, p<0·0001) per 100 000 person-years in all residents. Vi-TT conferred 85% (97·5% CI 76 to 91, p<0·0001) total protection against typhoid fever (table 2). Overall protection against typhoid was 57% (97·5% CI 43 to 68, p<0·0001), whereas indirect protection was modest (19%) and did not reach significance (95% CI −12 to 41). Table 3 shows measures of Vi-TT protection stratified by age at start date of residence. Total protection by Vi-TT did not differ significantly by age (p=0·49), ranging from 80% to 88% for children vaccinated at ages younger than 2 years (81%), 2 to 4 years (80%), and 5 to less than 16 years (88%) and 95% CIs for these point estimates did not go below 39%. Overall protection varied more by age at the start date of residence but was significant for all age groups apart from 16 years and older, the age group that included only non-vaccinees. Indirect protection was not significant for any age group, although it was of marginal significance for the group 16 years and older (33%; 95% CI −2 to 55, p=0·060). No protection by Vi-TT was evident against paratyphoid fever (appendix p 3).

**Table 2:** Incidence of blood culture-confirmed typhoid fever and protective effectiveness of Vi-TT*

		**SA 14-14-2 group**	**Vi-TT group**	**Protective effectiveness (%)**	**Intracluster correlation coefficients**	**p value**
Total vaccine protection		n=30 685	n=30 882	..	..	..
	Typhoid fever	192	29	..	..	..
	Person-years of follow-up	30 253	30 349	..	..	..
	Incidence rate, per 100 000 person-years	635 (548–731)	96 (64–137)	85% (97·5% CI 76 to 91)	0·0013	<0·0001
Overall vaccine protection		n=155 448	n=155 841	..	..	..
	Typhoid fever	331	144	..	..	..
	Person-years of follow-up	155 458	154 449	..	..	..
	Incidence rate, per 100 000 person-years	213 (191–237)	93 (79–110)	57% (97·5% CI 43 to 68)	0·0007	<0·0001
Indirect vaccine protection		n=134 835	n=135 110	..	..	..
	Typhoid fever	139	115	..	..	..
	Person-years of follow up	125 205	124 100	..	..	..
	Incidence rate, per 100 000 person-years	111 (93–131)	93 (77–111)	19% (95% CI −12 to 41)	0·0008	0·20

Data are incidence rate (95% CI) or % (95% CI) unless stated otherwise.

* Protective effectiveness, p values, and CIs were adjusted for the stratifying variables for randomisation, including geographical ward, distance to study clinics, number of eligible children at baseline, and other baseline covariates prespecified in the statistical analysis plan, including age, sex, toilet type in the house, drinking water source, treatment of drinking water, handwashing before meals, and handwashing after defecation. SA 14-14-2=Japanese encephalitis vaccine. Vi-TT=Vi-tetanus toxoid conjugate vaccine.

**Table 3:** Incidence of blood culture-confirmed typhoid fever and protective effectiveness of Vi-TT by age group*

	**Events/person-years**†		**Incidence, per 100 000 person-years**		**Protective effectiveness**	**p value**	**p value for interaction**
	SA 14-14-2 group	Vi-TT group	SA 14-14-2 group	Vi-TT group			
**Total vaccine protection**							
9 months to <2 years	23/2804	4/2800	820 (520–1231)	143 (39–366)	81% (39 to 94)	0·0052	0·49
2 to 4 years	62/6413	12/6173	967 (741–1239)	194 (100–340)	80% (62 to 89)	<0·0001	..
5 to <16 years†	107/21 037	13/21 375	509 (417–615)	61 (32–104)	88% (78 to 93)	<0·0001	..
**Overall vaccine protection**							
<2 years	35/7779	13/7861	450 (313–626)	165 (88–283)	63% (20 to 83)	0·011	0·056
2 to 4 years	86/9295	34/9041	925 (740–1143)	376 (260–526)	59% (40 to 73)	<0·0001	..
5 to <16 years	141/32 316	50/32 462	436 (367–515)	154 (114–203)	65% (50 to 75)	<0·0001	..
≥16 years†	69/106 069	47/105 085	65 (51–82)	45 (33–59)	33% (−2 to 55)	0·061	..
**Indirect vaccine protection**							
<2 years	12/4846	8/4913	248 (128–433)	163 (70–321)	32% (−127 to 80)	0·53	0·38
2 to 4 years	24/2884	23/2880	832 (533–1238)	799 (506–1198)	6% (−78 to 51)	0·84	..
5 to <16 years	34/11 415	37/11 227	298 (206–416)	330 (232–454)	−13% (80 to 29)	0·60	..
≥16 years†	69/106 061	47/105 081	65 (51–82)	45 (33–59)	33% (−2 to 55)	0·060	..

Data are incidence rate/person-years, n (95% CI), or % (95% CI). SA 14-14-2=Japanese encephalitis vaccine. Vi-TT=Vi-tetanus toxoid conjugate vaccine.

* Age at vaccination for the subgroup analyses of total vaccine protection, and age at date of residence for that of overall and indirect vaccine protection analyses; protective effectiveness, p values and CIs were adjusted for the stratifying variables for randomisation, including geographical ward, distance to study clinics, number of eligible children at baseline, and other baseline covariates prespecified in the statistical analysis plan, including age, sex, toilet type in the house, drinking water source, treatment of drinking water, handwashing before meals, and handwashing after defecation.

† 11 vaccinees were ≥16 years at vaccination and were included in the total vaccination analysis of the 5 to <16 years age group, resulting in a 12 person-years difference between overall and indirect vaccine protection analysis of the ≥16 years age group.

Among the 7-day, active surveillance subset of 2507 children who received SA 14-14-2 and 2500 who received Vi-TT, including participants in the pilot study, the most common complaints were fever (5·3%), a general feeling of unwellness (4·3%), diarrhoea (2·1%), and pain at the injection site (1·6%). Risks of these events were similar in the two vaccine groups. In the 6 months post-vaccination (the period prespecified for analysis), there were 370 serious adverse events reported in the SA 14-14-2 vaccine group and 314 in the Vi-TT group (p=0·029) among all vaccinees. None was judged related to receipt of vaccine, and no component cause appeared to account for the overall difference (appendix p 8). During the entire period of follow-up, there were 13 deaths in the SA 14-14-2 vaccine group and 25 in the Vi-TT group (p=0·073); none were judged to be related to vaccination. Although there were 12 more deaths in the Vi-TT group than in the SA 14-14-2 group, there were eight more deaths due to trauma or drowning in the Vi-TT group than the SA 14-14-2 group.

Blood samples were obtained from 1515 vaccinees at baseline and 1433 vaccinees 28 days after dosing. Plasma IgG anti-Vi titres were similar for the Vi-TT and SA 14-14-2 vaccine groups at baseline. On day 28 after dosing, the median titres (in ELISA units) were 3·7 (IQR 3·7–3·7) for the SA 14-14-2 group and 3222·4 (IQR 1757·0–5471·6) for the Vi-TT group (p<0·0001; appendix p 4). For participants sampled both at baseline and on day 28, seroconversion, defined as greater than a four-fold rise in plasma antibody titres on day 28 compared with baseline, was found in 950 (99·5%) of 954 patients in the Vi-TT group and eight (1·7%) of 477 patients in the SA 14-14-2 group (p<0·0001). Robust anti-Vi IgG responses were seen in recipients of Vi-TT in all age groups (appendix p 9).

## Discussion

Our results show that a single-dose regimen of Vi-TT, given to participants aged 9 months to less than 16 years in urban Bangladesh, was safe, immunogenic, and highly protective against typhoid fever over an average of 17·1 months of post-dosing follow-up. Our findings are consistent with those of an individually randomised efficacy trial of Vi-TT in Nepal.^14^ Moreover, we show for the first time significant, high-level protection of infants and children vaccinated at ages younger than 2 years and significant overall population-level protection. Our cluster-randomised trial failed to find significant herd protection from this vaccine, although, as discussed below, there might have been methodological reasons for this finding.

Before considering the implications of these findings, it is important to discuss the potential limitations of the study. The trial was not formally double-blinded, although measures were taken to help ensure that it was done in a participant-masked and observer-masked fashion. Also weighing against bias was the observation that Vi-TT conferred no protection against enteric fever due to *S* Paratyphi A (the only serotype isolated in the trial), a disease clinically resembling typhoid fever and with a similar transmission route but not expected to be prevented by Vi-TT due to an absence of ViPS capsule in *S* Paratyphi A organisms. Another limitation was that, in order to maintain a reasonably high degree of Vi-TT coverage of the target age group over time, catch-up vaccination campaigns were done at 6-month intervals, a strategy unlikely to be feasible in routine public health practice.

Our trial failed to find evidence of significant indirect (herd) protection from Vi-TT. This could have occurred for methodological reasons. On the basis of mathematical model simulations, we expected indirect protection to be approximately 20% and established that the trial would not be adequately powered to evaluate this amount of vaccine protection; we therefore evaluated indirect protection in a prespecified secondary analysis.^15^ Moreover, the boundaries of the clusters were unlikely to represent boundaries of short-cycle, person-to-person transmission of typhoid, either directly or via intermediate vehicles, in the densely populated study area and transmission into the clusters from populations outside cluster boundaries was likely to have occurred, which would be predicted to dilute estimates of vaccine herd protection.^24^ In future analyses of vaccine herd protection in the present trial, we will use the so-called fried egg approach, which focuses only on people residing in the inner cores of the clusters, whose risk of typhoid was not as likely to have beeen affected by transmission into the cluster from the outside.^25^ Furthermore, limiting vaccination to children and adolescents might have played a role. Earlier cluster-randomised trials of ViPS vaccine against typhoid showed considerable vaccine herd protection when all people aged 2 years and older in Kolkata were targeted, but no vaccine herd protection when only children aged 2–16 years were targeted in Karachi.^26^, ^27^ In Kolkata, the ViPS vaccine trial was done in 37 673 participants with 2 years of follow-up; the trial reported 80% (95% CI 53 to 91) protective effectiveness for children under 5 years, 56% (18 to 77) for children aged 5–14 years and 46% (−43 to 79) for vaccinees of older age.^26^ The trial in Karachi with the same vaccine, done in 51 965 participants, showed a protective effectiveness of −38% (−192 to 35) for children aged 2–4 years and 57% (6 to 81) for children aged 5–16 years.^27^

A few studies done with typhoid conjugated vaccines have shown different protection level. An experiment on a human challenge model with Vi-TT vaccine done in the UK, a typhoid non-endemic area, evaluated only the immunological endpoints.^28^ An earlier individually randomised efficacy trial of Vi-rEPA (recombinant *Pseudomonas aeruginosa* exoprotein A) vaccine has reported 91·5% (95% CI 77·1–96·6%; p<0·001) protection in Vietnamese children aged 2–5 years.^29^ A school-based cluster-randomised trial of a two-dose regimen of Vi-TT vaccine (PedaTyph), which conferred 100% (97·6–100%) protection, was done in a small number of children (n=1765).^30^

The implications of our findings are both biological and programmatic. From a biological perspective, the protection of children vaccinated at younger than 2 years of age is consistent with the concept that Vi-TT induces T-cell-dependent antibody responses; it is believed that the reason earlier ViPS-only vaccines were not efficacious in very young children was that they fail to elicit T-cell-independent antibody responses. Although these T-cell-dependent antibody responses might also entail induction of immune memory and sustained protection by Vi-TT, this needs to be shown. Programmatically, although our trial was not adequately powered to evaluate the clinical protection by Vi-TT when given at specific contact visits of the schedule, it is nonetheless reassuring that substantial clinical protection was observed when the vaccine was given to participants as young as 9 months of age.

In conclusion, our findings strongly support global efforts by WHO and the Global Alliance for Vaccines and Immunization to introduce Vi-TT vaccine into public health programmes for populations affected by high rates of endemic typhoid fever, including those with a high burden of typhoid in very young children. Fine tuning such efforts, including the potential use of booster doses of Vi-TT, will require further work to define the duration of vaccine protection.

## Data sharing

Deidentified individual participant data including a data dictionary for each variable analysed in the published paper, as well as the study protocol, the statistical analysis plan, and the informed consent form will be made available when the trial is complete, on requests directed to the corresponding author (fqadri@icddrb.org). Only after approval of a proposal can data be shared through a secure online platform. Approval of the proposal will be subject to scientific review by the icddr,b Research Review Committee and by the institutional review board at icddr,b. Sharing of data will also be subject to the published data access rules of the icddr,b. The requestor will need to sign a standard data access agreement required by the icddr,b.

## Declaration of interests

VEP has received reimbursement from Merck and Pfizer for travel expenses to scientific input engagements unrelated to the topic of this manuscript and is a member of the WHO Immunization and Vaccine-related Implementation Research Advisory Committee. All other authors declare no competing interests.
