# Supplementary material for: Protection by vaccination of children against typhoid fever with a Vi-tetanus toxoid conjugate vaccine in urban Bangladesh: a cluster-randomised trial
Source: Lancet. 2021 Aug 21;398(10301):675–84. doi: 10.1016/S0140-6736(21)01124-7 (PMC8387974; doi:10.1016/S0140-6736(21)01124-7)
Supplement: Supplementary appendix [file mmc1.pdf]

THE LANCET

Supplementary appendix

This appendix formed part of the original submission and has been peer reviewed. We post it as supplied by the authors.

Supplement to: Qadri F, Khanam F, Liu X, et al. Protection by vaccination of children against typhoid fever with a Vi-tetanus toxoid conjugate vaccine in urban Bangladesh: a cluster-randomised trial. *Lancet* 2021; published online Aug 9. http://dx.doi.org/10.1016/S0140-6736(21)01124-7.

Table S1 Individual, household and cluster level features at the date of vaccination by vaccine arm for the analysis of total vaccine protection

	SA 14-14-2	Vi-TT
Individual level features		
All vaccinees	N=30685	N=30882
Median (IQR) age (yrs) at vaccination	7.6 (3.9-11.3)	7.7 (4.0-11.4)
Sex		
Male	15391 (50.2%)	15332 (49.6%)
Female	15294 (49.8%)	15550 (50.4%)
Ward of residence		
2	11432 (37.3%)	12329 (39.9%)
3	7237 (23.6%)	8273 (26.8%)
5	12016 (39.2%)	10280 (33.3%)
Formal education		
Yes	18840 (61.4%)	18937 (61.3%)
No	3408 (11.1%)	3531 (11.4%)
Missing	8437 (27.5%)	8414 (27.2%)
Religion		
Muslim	30423 (99.1%)	30526 (98.8%)
Others	262 (0.9%)	356 (1.2%)
Household (with at least one vaccine) level features		
	N=19392	N=19569
Toilet type in the house		
Flush toilet	957 (4.9%)	1104 (5.6%)
Others	18435 (95.1%)	18465 (94.4%)
Household drinking water source		
Own water source	5497 (28.3%)	5020 (25.7%)
Others	13895 (71.7%)	14549 (74.3%)
Household boiling or filtering of drinking water		
Yes	14354 (74.0%)	14133 (72.2%)
No	5034 (26.0%)	5436 (27.8%)
Missing	4 (0.0%)	0 (0.0%)
Handwashing before meals		
Yes	14028 (72.3%)	14608 (74.6%)
No	5364 (27.7%)	4961 (25.4%)
Handwashing after defecation		
Yes	18885 (97.4%)	19083 (97.5%)
No	507 (2.6%)	486 (2.5%)

Median (IQR) household monthly expenditure in Bangladeshi taka	15,000 (12,000-20,000) [n=19360]	15,300 (12,000-20,000) [n=19564]

Table S2 Individual, household and cluster level features among non-vaccinees at the date of residence by vaccine arm for the analysis of indirect vaccine protection

	SA 14-14-2	Vi-TT
	Individual level features	
	N=134835	N=135110
Median (IQR) age (yrs) at residence	27.5 (17.9-38.9)	27.3 (17.8-38.7)
Sex		
Male	66942 (49.6%)	67221 (49.8%)
Female	67893 (50.4%)	67889 (50.2%)
Ward of residence		
2	51981 (38.6%)	54688 (40.5%)
3	31852 (23.6%)	36513 (27.0%)
5	51002 (37.8%)	43909 (32.5%)
Formal education		
Yes	21764 (16.1%)	21457 (15.9%)
No	101336 (75.2%)	101106 (74.8%)
Missing	11735 (8.7%)	12547 (9.3%)
Religion		
Muslim	133279 (98.8%)	133219 (98.6%)
Others	1556 (1.2%)	1891 (1.4%)
	Household level features	
	N=38620	N=38613
Toilet type in the house		
Flush toilet	2104 (5.4%)	2297 (5.9%)
Others	36516 (94.6%)	36316 (94.1%)
Household drinking water source		
Own water source	10760 (27.9%)	9846 (25.5%)
Others	27860 (72.1%)	28767 (74.5%)
Household boiling or filtering of drinking water		
Yes	28962 (75.0%)	28284 (73.2%)
No	9652 (25.0%)	10328 (26.7%)
Missing	6 (0.0%)	1 (0.0%)
Handwashing before meals		
Yes	28485 (73.8%)	29076 (75.3%)
No	10135 (26.2%)	9537 (24.7%)

Handwashing after defecation		
Yes	37677 (97.6%)	37653 (97.5%)
No	943 (2.4%)	960 (2.5%)
Median (IQR) household monthly expenditure in Bangladeshi taka	14,500 (11,000-19,500) [n=38545]	14,500 (11,000-19,900) [n=38605]

Table S3 Incidence of blood culture- confirmed paratyphoid fever and protective effectiveness of Vi-TT*

Analyses	SA 14-14-2	Vi-TT	Protective Effectiveness (%) [95%CI]	P Value
Total Vaccine Protection	N=30,685	N=30,882		
Clinical typhoid fever (no.)	30	30		
Person-years (PYs) of follow up	30,253	30,349		
Incidence rate (per 100,000 PYs) [95%CI]	99 [67, 142]	99 [67, 141]		
			0 [-80,45]	>0.99
Overall Vaccine Protection	N=155,448	N=155,841		
Clinical typhoid fever (no.)	49	45		
Person-years (PYs) of follow up	155,458	154,449		
Incidence rate (per 100,000 PYs) [95%CI]	32 [23, 42]	29 [21, 39]		
			7 [-44,40]	0.74
Indirect Vaccine Protection	N=134,835	N=135,110		
Clinical typhoid fever (no.)	19	15		
Person-years (PYs) of follow up	125,205	124,100		
Incidence rate (per 100,000 PYs) [95%CI]	15 [9, 24]	12 [7, 20]		
			18 [-62,58]	0.57

*Protective effectiveness, P values, and confidence intervals were adjusted for the stratification variables for randomization, including geographic ward, distance to study clinics, number of eligible children at baseline, and other baseline covariates prespecified in the statistical analysis plan, including age at zero time, sex, toilet type in the house, drinking water source, treatment of drinking water, handwashing before meals, handwashing after defecation

Table S4 Plasma IgG anti-Vi titers at baseline and at 28 days, with corresponding fold of titers rises and seroconversion rates*

	SA 14-14-2	Vi-TT	P value§
Day 0	N=505	N=1,010	
% below detection threshold	400 (79.2%)	794 (78.6%)	0.84
Median (Interquartile range, IQR), ELISA Units/ml	3.7 (3.7-3.7)	3.7 (3.7-3.7)	0.96
Day 28	N=477	N=954	
% below detection threshold	367 (76.9%)	3 (0.3%)	<0.0001
Median (IQR), ELISA Units/ml	3.7 (3.7-3.7)	3222.4 (1757-5471.6)	<0.0001
Fold rise			
Median (IQR)	1.0 (1.0-1.0)	693 (366-1198)	<0.0001
Seroconversion			
No	469 (98.3%)	4 (0.4%)	<0.0001
Yes	8 (1.7%)	950 (99.6%)	

*The median between zero and the detection threshold was used to impute data under detection threshold

§Chi-squared test was used for categorical variables and Mann–Whitney U test was used for continuous variables

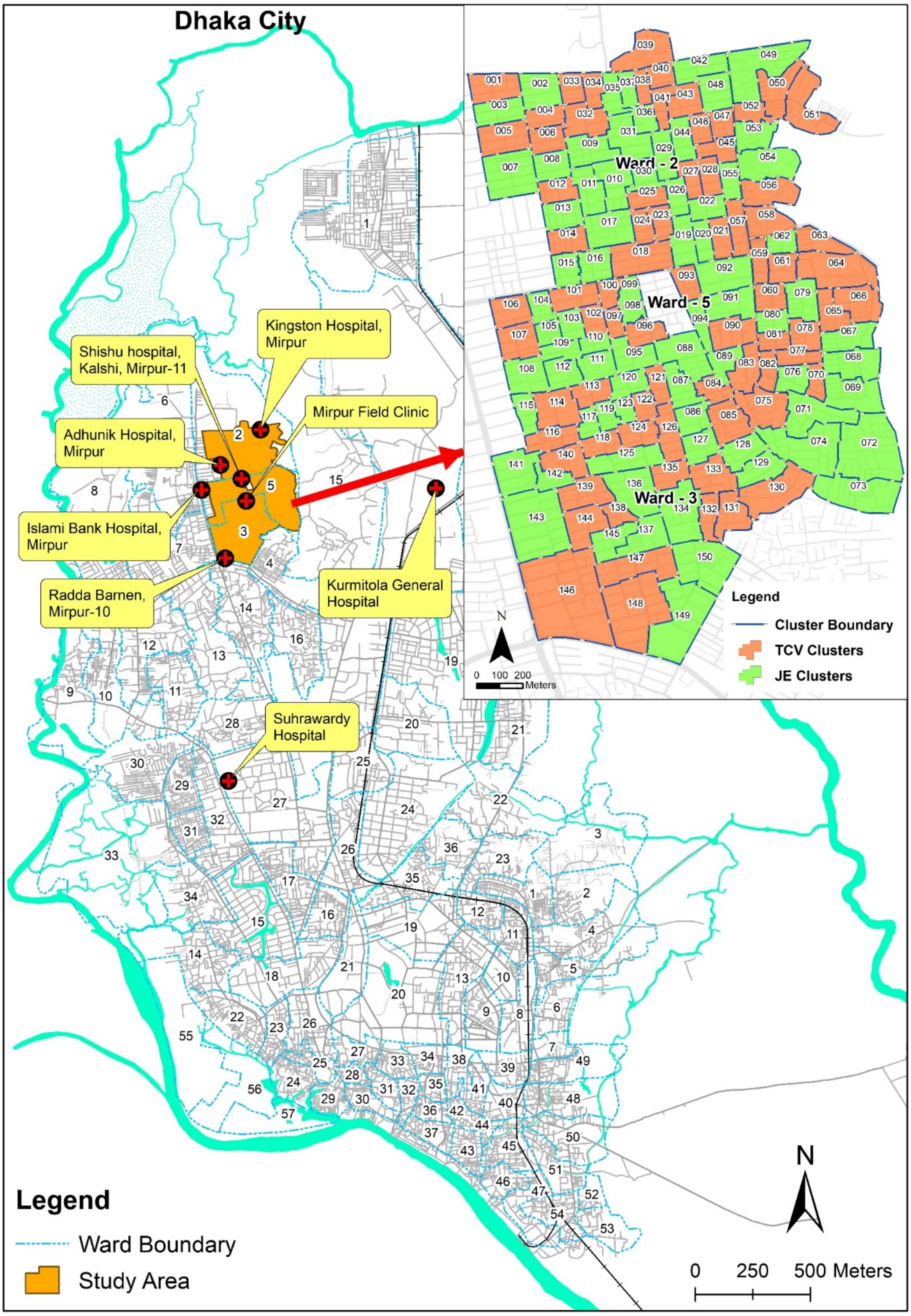

Figure S1 Map of the whole Dhaka city indicating the study area with clinical facilities and with 150 clusters.

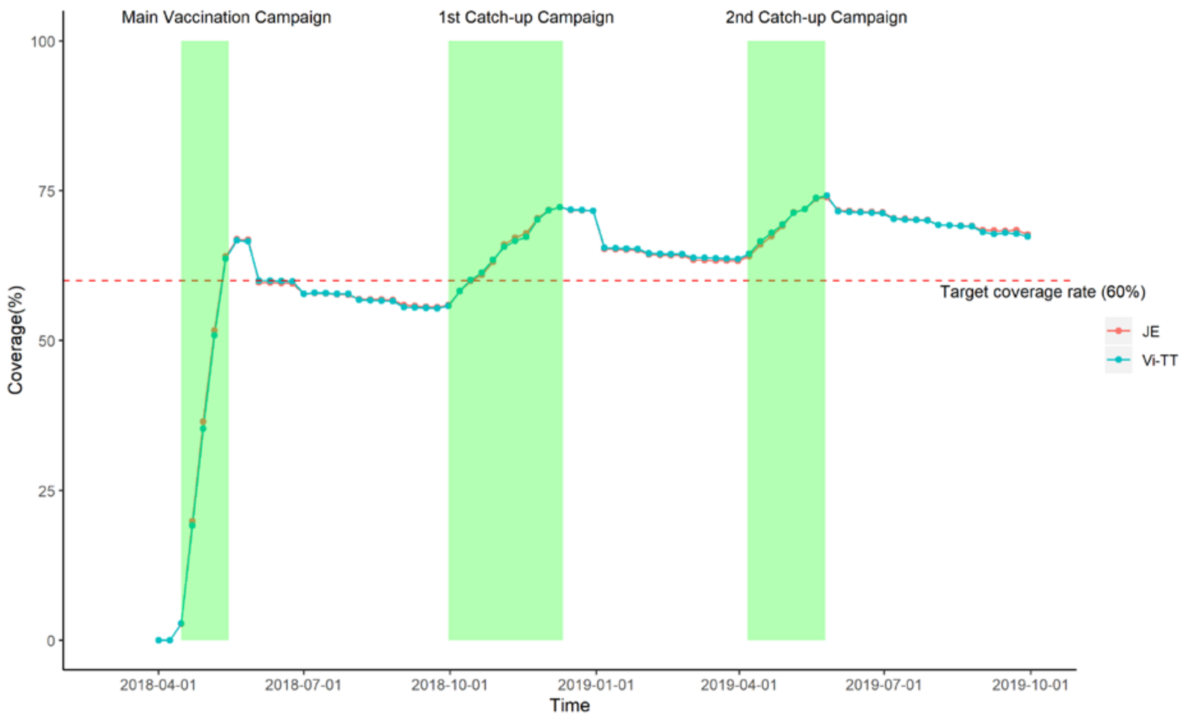

Figure S2 Level of coverage of the target age group (9 months to <16) years with SA 14-14-2 and Vi-TT vaccines during the course of the study.

Data are presented at weekly intervals and the coverage rates are calculated by the number of vaccinees aged 9 months to <16 years divided by the total number of children this age on the first day of each weekly interval.

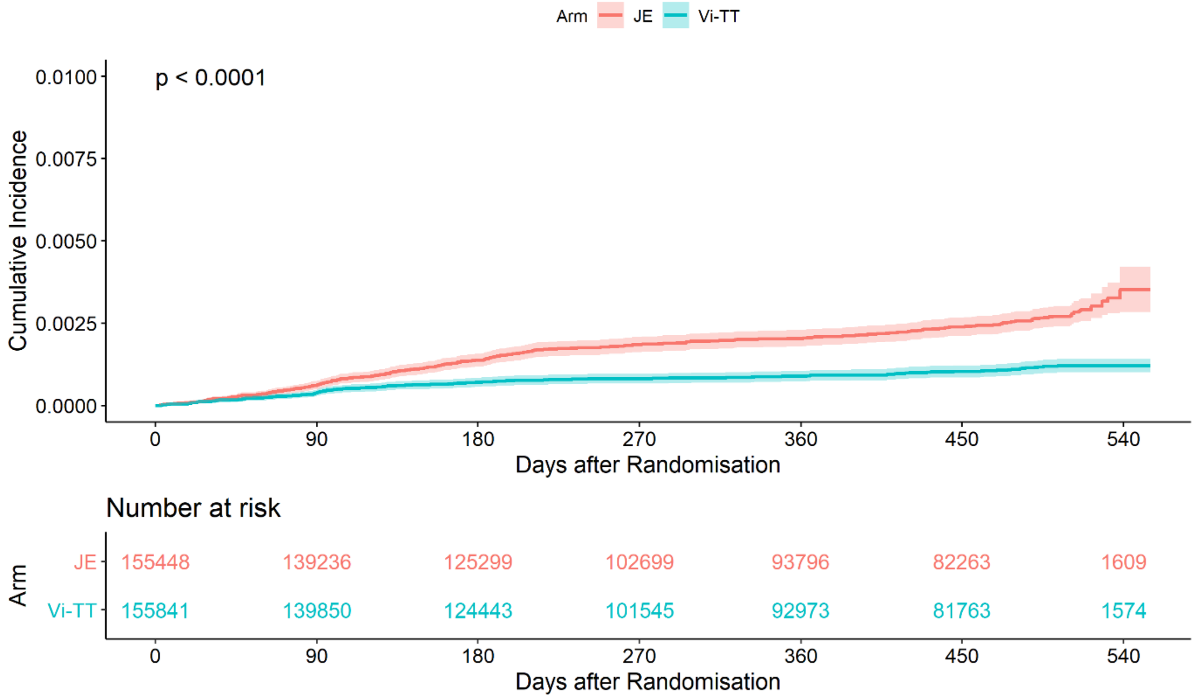

Figure S3 The cumulative incidence of blood culture-confirmed typhoid fever in all residents included in the analysis of overall Vi-TT protection by treatment arm

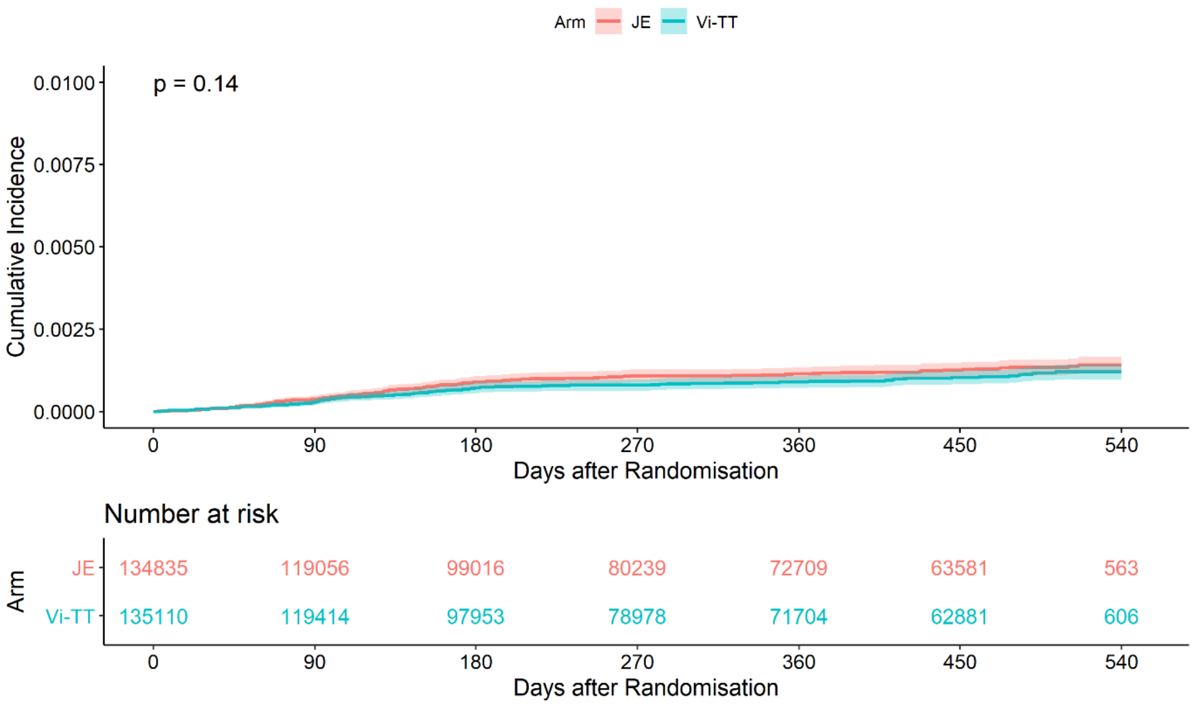

Figure S4 The cumulative incidence of blood culture-confirmed typhoid fever in non-vaccinees included in the analysis of indirect Vi-TT protection by treatment arm

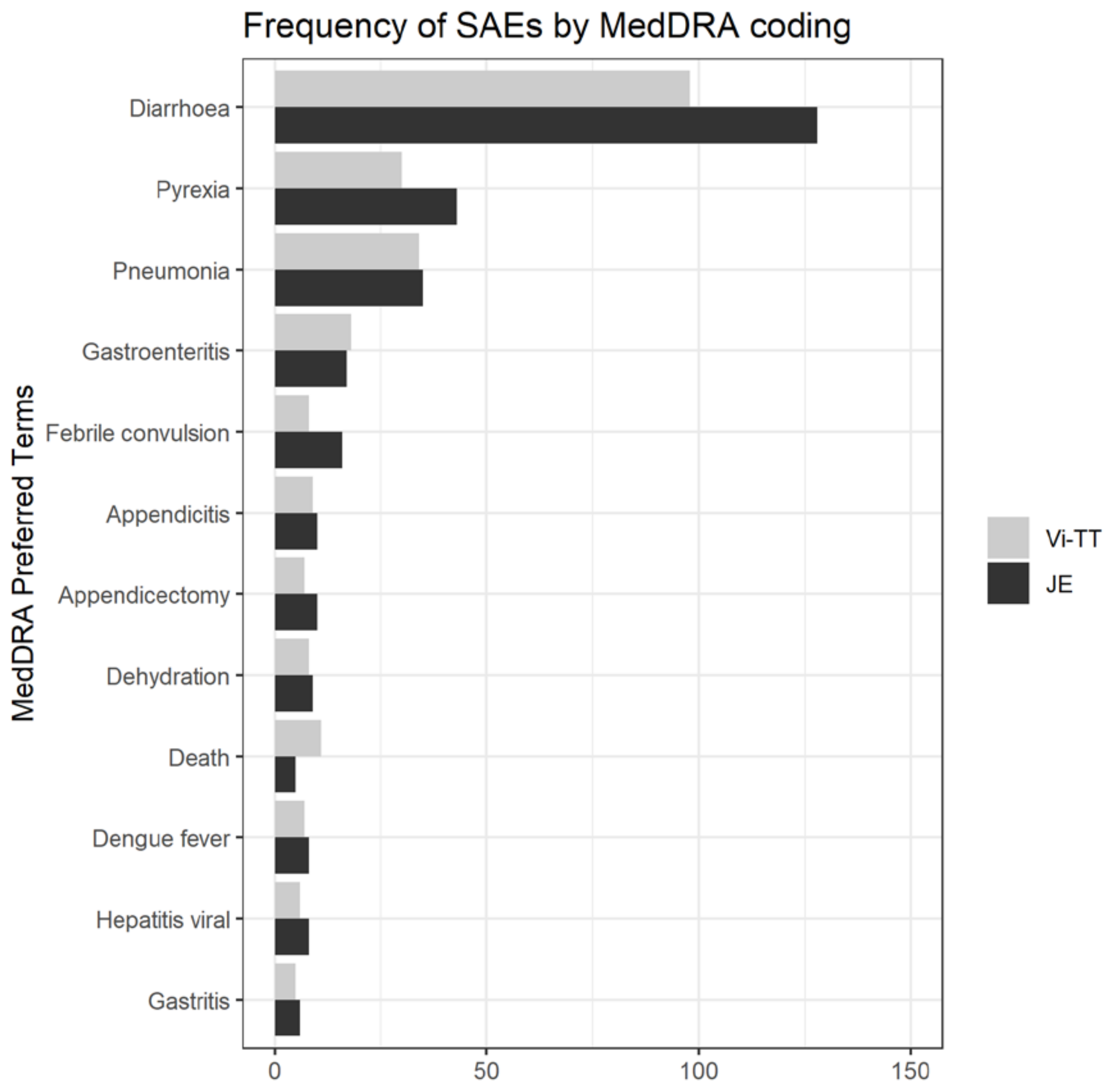

Figure S5 Frequency of severe adverse events (SAEs) during 6 months post vaccination by Preferred Terms of MedDRA Coding (Frequency >10)

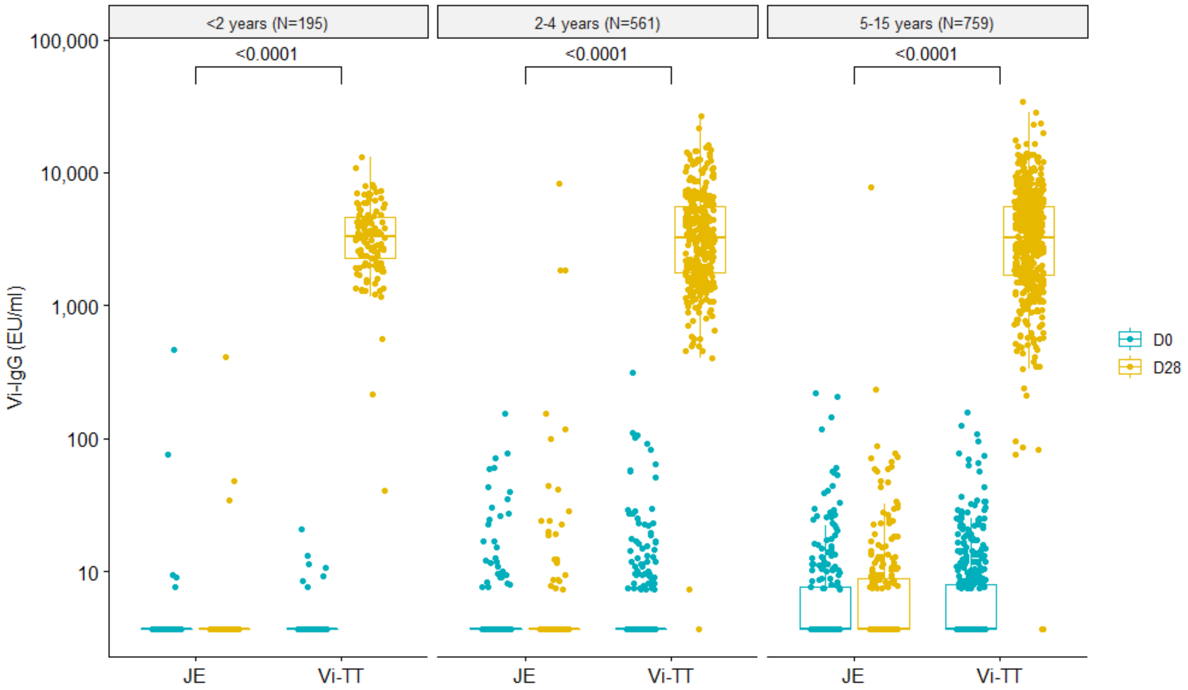

Figure S6 Boxplot of plasma anti-Vi IgG responses (in ELISA unit) in children vaccinated at different ages

The comparisons between SA 14-14-2 and Vi-TT at D28 across different age groups were tested using the Mann–Whitney U test

CONSORT 2010 checklist of information to include when reporting a cluster randomised trial

Section/Topic	Item No	Standard Checklist item	Extension for cluster designs	Page No *
Title and abstract				
	1a	Identification as a randomised trial in the title	Identification as a cluster randomised trial in the title	1
	1b	Structured summary of trial design, methods, results, and conclusions (for specific guidance see CONSORT for abstracts)[i],[ii]	See table 2	2, 3
Introduction				
Background and objectives	2a	Scientific background and explanation of rationale	Rationale for using a cluster design	4, 5
	2b	Specific objectives or hypotheses	Whether objectives pertain to the cluster level, the individual participant level or both	5
Methods				
Trial design	3a	Description of trial design (such as parallel, factorial) including allocation ratio	Definition of cluster and description of how the design features apply to the clusters	5-8
	3b	Important changes to methods after trial commencement (such as eligibility criteria), with reasons		11 (lines 223-230)

Participants	4a	Eligibility criteria for participants	Eligibility criteria for clusters	6
	4b	Settings and locations where the data were collected		5, 6, 10
Interventions	5	The interventions for each group with sufficient details to allow replication, including how and when they were actually administered	Whether interventions pertain to the cluster level, the individual participant level or both	6 (heading of intervention), 7
Outcomes	6a	Completely defined pre-specified primary and secondary outcome measures, including how and when they were assessed	Whether outcome measures pertain to the cluster level, the individual participant level or both	13-15
	6b	Any changes to trial outcomes after the trial commenced, with reasons		Not applicable
Sample size	7a	How sample size was determined	Method of calculation, number of clusters(s) (and whether equal or unequal cluster sizes are assumed), cluster size, a coefficient of intracluster correlation (ICC or k), and an indication of its uncertainty	11, protocol as supplementary
	7b	When applicable, explanation of any interim analyses and stopping guidelines		Not applicable

Randomisation:				
Sequence generation	8a	Method used to generate the random allocation sequence		8
	8b	Type of randomisation; details of any restriction (such as blocking and block size)	Details of stratification or matching if used	8
Allocation concealment mechanism	9	Mechanism used to implement the random allocation sequence (such as sequentially numbered containers), describing any steps taken to conceal the sequence until interventions were assigned	Specification that allocation was based on clusters rather than individuals and whether allocation concealment (if any) was at the cluster level, the individual participant level or both	7, 8 (lines 138-145)
Implementation	10	Who generated the random allocation sequence, who enrolled participants, and who assigned participants to interventions	Replace by 10a, 10b and 10c	
	10a		Who generated the random allocation sequence, who enrolled clusters, and who assigned clusters to interventions	7
	10b		Mechanism by which individual participants were included in clusters for the	6, 7

			purposes of the trial (such as complete enumeration, random sampling)	
	10c		From whom consent was sought (representatives of the cluster, or individual cluster members, or both), and whether consent was sought before or after randomisation	6, 7
Blinding	11a	If done, who was blinded after assignment to interventions (for example, participants, care providers, those assessing outcomes) and how		7, 8
	11b	If relevant, description of the similarity of interventions		7
Statistical methods	12a	Statistical methods used to compare groups for primary and secondary outcomes	How clustering was taken into account	12, 13
	12b	Methods for additional analyses, such as subgroup analyses and adjusted analyses		12, 13
Results				

Participant flow (a diagram is strongly recommended)	13a	For each group, the numbers of participants who were randomly assigned, received intended treatment, and were analysed for the primary outcome	For each group, the numbers of clusters that were randomly assigned, received intended treatment, and were analysed for the primary outcome	31
	13b	For each group, losses and exclusions after randomisation, together with reasons	For each group, losses and exclusions for both clusters and individual cluster members	31
Recruitment	14a	Dates defining the periods of recruitment and follow-up		6, 8, 10
	14b	Why the trial ended or was stopped		Not Applicable
Baseline data	15	A table showing baseline demographic and clinical characteristics for each group	Baseline characteristics for the individual and cluster levels as applicable for each group	23, 24
Numbers analysed	16	For each group, number of participants (denominator) included in each analysis and whether the analysis was by original assigned groups	For each group, number of clusters included in each analysis	23, 24, 31
Outcomes and estimation	17a	For each primary and secondary outcome, results for	Results at the individual or cluster level as applicable and a	25, 26, 29

		each group, and the estimated effect size and its precision (such as 95% confidence interval)	coefficient of intracluster correlation (ICC or k) for each primary outcome	
	17b	For binary outcomes, presentation of both absolute and relative effect sizes is recommended		14 (lines 299-301)
Ancillary analyses	18	Results of any other analyses performed, including subgroup analyses and adjusted analyses, distinguishing pre-specified from exploratory		26
Harms	19	All important harms or unintended effects in each group (for specific guidance see CONSORT for harms[iii])		15
Discussion				
Limitations	20	Trial limitations, addressing sources of potential bias, imprecision, and, if relevant, multiplicity of analyses		16
Generalisability	21	Generalisability (external validity, applicability) of the trial findings	Generalisability to clusters and/or individual participants (as relevant)	16 (lines 333-335)

Interpretation	22	Interpretation consistent with results, balancing benefits and harms, and considering other relevant evidence	17, 18
Other information			
Registration	23	Registration number and name of trial registry	6
Protocol	24	Where the full trial protocol can be accessed, if available	Yes (submitted)
Funding	25	Sources of funding and other support (such as supply of drugs), role of funders	21

Table: Sex and Gender Equity in Research (SAGER) guidelines

General principles		Author's response
• Authors should use the terms sex and gender carefully in order to avoid confusing both terms.		We have only used the term "sex" in the manuscript.
• Where the subjects of research comprise organisms capable of differentiation by sex, the research should be designed and conducted in a way that can reveal sex-related differences in the results, even if these were not initially expected.		Not applicable.
• Where subjects can also be differentiated by gender (shaped by social and cultural circumstances), the research should be conducted similarly at this additional level of distinction.		Not applicable.
Recommendations per section of the article		
Title and abstract	If only one sex is included in the study, or if the results of the study are to be applied to only one sex or gender, the title and the abstract should specify the sex of animals or any cells, tissues and other material derived from these and the sex and gender of human participants.	The study included both male and female participants.
Introduction	Authors should report, where relevant, whether sex and/or gender differences may be expected.	Not applicable
Methods	Authors should report how sex and gender were taken into account in the design of the study, whether they ensured adequate representation of males and females, and justify the reasons for any exclusion of males or females.	This was a cluster randomized trial where we have enrolled all children aged 9 months to <16 years residing in both intervention and control clusters and vaccinated them based on the randomization list of clusters irrespective of their sex and gender. However, there was adequate representation of males and females in the study.
Results	Where appropriate, data should be routinely presented disaggregated by sex and gender. Sex- and gender-based analyses should be reported regardless of positive or negative outcome. In clinical trials, data on withdrawals and dropouts should also be reported disaggregated by sex.	Numbers (%) of males and females enrolled in the intervention and control clusters are mentioned in the Tables 1, S1, S2.
Discussion	The potential implications of sex and gender on the study results and analyses should be discussed. If a sex and gender analysis was not conducted, the rationale should be given. Authors should further discuss the implications of the lack of such	We did not expect any potential implications of sex and gender on the study results of this phase IIIb vaccine efficacy trial and thus did not address them in the Discussion.

	analysis on the interpretation of the results.	

icddr,b

RRC APPLICATION FORM

RESEARCH PROTOCOL
Number: PR-17115
Version No. 14.00
Version date: 13-12-2020

FOR OFFICE USE ONLY

RRC Approval:	☒ Yes	☐ No	Date:19-12-2017
ERC Approval:	☒ Yes	☐ No	Date:21-01-2018
AEEC Approval:	☐ Yes	☐ No	Date:
External IRB Approval	☐ Yes	☐ No	Date:

Name of External IRB: ______________________________________

Protocol Title:* Assessing the impact of a Vi-Polysaccharide Conjugate Vaccine in preventing typhoid infection among Bangladeshi children – a Phase IIIb trial

Short Title: (maximum **100** characters including space) TyVAC Bangladesh: Typhoid Vaccine Trial

Key Words:* Typhoid fever, Vi-Polysaccharide Conjugate Vaccine, Bangladeshi children, TyVOID (Elimination of typhoid fever through vaccination)

Name of the Research Division Hosting the Protocol:*

☐ Health Systems and Population Studies Division (HSPSD)
☐ Nutrition and Clinical Services Division (NCSD)
☒ Infectious Diseases Division (IDD)
☐ Maternal and Child Health Division (MCHD)
☐ Laboratory Sciences and Services Division (LSSD)
☐ Other (specify) ______________________________

Has the Protocol been Derived from an Activity:* ☒ No ☐ Yes (please provide following information):

Activity No. :
Activity Title:
PI:
Grant No.: Budget Code: Start Date: End Date:

icddr,b Strategic Priority/ Initiative (SP 2015-8):* (check all that apply)

☐ Reducing maternal and neonatal mortality
☒ Controlling enteric and respiratory infections
☐ Preventing and treating maternal and childhood malnutrition
☐ Detecting and controlling emerging and re-emerging infections
☐ Achieving universal health coverage
☐ Examining the health consequences of climate change
☐ Preventing and treating non-communicable diseases
☐ Others (specify) ______________________________

Research Phase (4 Ds):* (check all that apply**)**

☐ Discovery
☒ Development
☒ Delivery
☒ Evaluation of Delivery

Anticipated Impact of Research:* (check all that apply and please provide details below**)**

☒ Knowledge Production
☐ Capacity Building
☒ Informing Policy
☐ Health and Health Sector Benefits
☐ Economic Benefits

Please provie details here: It is important to evaluate the effectiveness of the Vi-TCV vaccine in Bangladeshi children. The results of this study will be used to inform country decision making for introduction of Vi-TCV into the routine expanded programme on immunisation (EPI) in Bangladesh.

Which of the Sustainable Development Goal This Protocol Relates to?:* (check all that apply)

- ☐ 1. End poverty in all its forms everywhere
- ☐ 2. End hunger, achieve food security and improved nutrition and promote sustainable agriculture
- ☒ 3. Ensure healthy lives and promote well-being for all at all ages
- ☐ 4. Ensure inclusive and equitable quality education and promote lifelong learning opportunities for all
- ☐ 5. Achieve gender equality and empower all women and girls
- ☐ 6. Ensure availability and sustainable management of water and sanitation for all
- ☐ 7. Ensure access to affordable, reliable, sustainable and modern energy for all
- ☐ 8. Promote sustained, inclusive and sustainable economic growth, full and productive employment and decent work for all
- ☐ 9. Build resilient infrastructure, promote inclusive and sustainable industrialization and foster innovation
- ☐ 10. Reduce inequality within and among countries
- ☐ 11. Make cities and human settlements inclusive, safe, resilient and sustainable
- ☐ 12. Ensure sustainable consumption and production patterns
- ☐ 13. Take urgent action to combat climate change and its impacts
- ☐ 14. Conserve and sustainably use the oceans, seas and marine resources for sustainable development
- ☐ 15. Protect, restore and promote sustainable use of terrestrial ecosystems, sustainably manage forests, combat desertification, and halt and reverse land degradation and halt biodiversity loss
- ☐ 16. Promote peaceful and inclusive societies for sustainable development, provide access to justice for all and build effective, accountable and inclusive institutions at all levels
- ☐ 17. Strengthen the means of implementation and revitalize the global partnership for sustainable development

**Does this Protocol Use the Gender Framework:***<br>(Please visit: http://www.icddrb.net.bd/jahia/Jahia/pid/684 for Gender Alanysis Tool with instructions)	☒ Yes (please complete Gender Analysis Tool)<br>☐ No
If 'no' is the response, its reason(s) in brief:	
**Will this Research Specifically Benefit the Disadvantaged (**economically, socially and/or otherwise**):**	☒ Yes<br>☐ No
**Does this Protocol use Behaviour Change Communication:**	☐ Yes<br>☒ No
**Principal Investigator (Should be icddr,b staff):*** Sex ☒ Female ☐ Male<br><br>**Dr Firdausi Qadri**<br>(Position, phone no, extension no, cell, and email address**):**<br>Acting Senior Director<br>+880-2-9827001 (Ext:2431)<br>+88-01711595367<br>fqadri@icddrb.org<br><br>Do you have ethics certification? ☐ No ☒ Yes (please attach in your CV below)<br>Do you have RBM training certification? ☒ No ☐ Yes (please attach the certificate with CV below)	Primary Scientific Division of the PI<br>IDD

**Co-Principal Investigator(s) Internal:** Sex ☐ Female ☒ Male<br>**Dr. John Clemens**<br>(Position, phone no, extension no, cell, and email address):<br>Executive Director<br>Office of ED, icddr,b,<br>68, Shaheed Tajuddin Ahmed Sarani, Mohakhali, Dhaka-1212, Bangladesh.<br>Tel: 880 2 9841751 to 9841760, Ext. 3100; Fax: 8802- 8823116;<br>e-mail: jclemens@icddrb.org<br><br>Signature or written consent of Co-PI: ____________<br>(electronic signature or email or any sort of written consent)<br>[if more than one, please copy and paste this row for additional Co-PIs]<br><br>Do you have ethics certification? ☐ No ☒ Yes (please attach in your CV below)<br><br>Do you have RBM training certification? ☒ No ☐ Yes (please attach the certificate with CV below)	Primary Scientific Division/ Programme of the Co-PIExecutive Director, icddr,b<br><br>____________<br>Approval of the Respective Senior Director/ Programme Head<br><br>(Signature)
**Co-Principal Investigator(s) Internal:** Sex ☒ Female ☐ Male<br>**Dr Farhana Khanam**<br>(Position, phone no, extension no, cell, and email address):<br>Deputy Project Coordinator<br>Phone: (+880-2) 9827001 -10, Ext: 3498<br>farhanak@icddrb.org<br><br>Signature or written consent of Co-I: ____________<br>(electronic signature or email or any sort of written consent)<br>[if more than one, please copy and paste this row for additional Co-Is<br><br>Do you have ethics certification? ☐ No ☒ Yes (please attach in your CV below)<br><br>Do you have RBM training certification? ☐ No ☒ Yes (please attach the certificate with CV below)	Primary Scientific Division of the Co-I IDD<br><br>____________<br>Approval of the Respective Senior Director/ Programme Head<br><br>(Signature)
**Co-Principal Investigator(s) - External:** Sex ☐ Female ☒ Male<br>Address (provide full official address, including land phone no(s), extension no. (if any), cell phone number, and email address).<br><br>Signature or written consent of Co-PI: ____________<br>(electronic signature or email or any sort of written consent)<br>[if more than one, please copy and paste this row for additional Co-PIs]	
**Co-Investigator(s) - Internal:** Sex ☐ Female ☒ Male<br>**Dr. Md. Arifuzzaman Khan**<br>(Position, phone no, extension no, cell, and email address):<br>Senior Research Investigator<br>Phone: (+880-2) 9827001 -10, Ext: 3464<br>e-mail: arifkhan@icddrb.org<br><br>Signature or written consent of Co-I: ____________<br>(electronic signature or email or any sort of written consent)<br>[if more than one, please copy and paste this row for additional Co-Is<br><br>Do you have ethics certification? ☐ No ☒ Yes (please attach in your CV below)<br><br>Do you have RBM training certification? ☐ No ☒ Yes (please attach the certificate with CV below)	Primary Scientific Division of the Co-I IDD<br><br>____________<br>Approval of the Respective Senior Director/ Programme Head<br><br>(Signature)

**Co-Investigator(s) - Internal:** Sex ☒ Female ☐ Male<br>**Dr Fahima Chowdhury**<br>Project Coordinator<br>(Position, phone no, extension no, cell, and email address **):**<br>Phone: (+880-2) 9827001 -10, Ext: 3458<br>fchowdhury@icddrb.org<br><br>Signature or written consent of Co-I: ____________<br>(electronic signature or email or any sort of written consent)<br>[if more than one, please copy and paste this row for additional Co-Is<br><br>Do you have ethics certification? ☐ No ☒ Yes (please attach in your CV below)<br><br>Do you have RBM training certification? ☒ No ☐ Yes (please attach the certificate with CV below)	Primary Scientific Division of the Co-I IDD<br><br>____________<br>Approval of the Respective Senior Director/ Programme Head<br><br>(Signature)
**Co-Investigator(s) - Internal:** Sex ☐ Female ☒ Male<br>**Dr. Ashraful Islam Khan**<br>(Position, phone no, extension no, cell, and email address):<br>Scientist<br>Phone: (+880-2) 9827001 -10, Ext: 3459<br>e-mail: ashrafk@icddrb.org<br><br>Signature or written consent of Co-I: ____________<br>(electronic signature or email or any sort of written consent)<br>[if more than one, please copy and paste this row for additional Co-Is<br><br>Do you have ethics certification? ☐ No ☒ Yes (please attach in your CV below)<br><br>Do you have RBM training certification? ☒ No ☐ Yes (please attach the certificate with CV below)	Primary Scientific Division of the Co-I IDD<br><br>____________<br>Approval of the Respective Senior Director/ Programme Head<br><br>(Signature)
**Co-Investigator(s) - Internal:** Sex ☐ Female ☒ Male<br>**Dr. Md. Khalequzzaman**<br>(Position, phone no, extension no, cell, and email address):<br>Emeritus Scientist<br>Phone: (+880-2) 9827001 -10, Ext: 3806<br>e-mail: kzaman@icddrb.org<br><br>Signature or written consent of Co-I: ____________<br>(electronic signature or email or any sort of written consent)<br>[if more than one, please copy and paste this row for additional Co-Is<br><br>Do you have ethics certification? ☐ No ☒ Yes (please attach in your CV below)<br><br>Do you have RBM training certification? ☒ No ☐ Yes (please attach the certificate with CV below)	Primary Scientific Division of the Co-I IDD<br><br>____________<br>Approval of the Respective Senior Director/ Programme Head<br><br>(Signature)

**Co-Investigator(s) - Internal:** Sex ☐ Female ☒ Male<br>**Dr. Niyaz Ahmed Abdus Samad**<br>(Position, phone no, extension no, cell, and email address):<br>Senior Director<br>Phone: (+880-2) 9827063, Ext: 2400<br>e-mail: niyaz.ahmed@icddrb.org<br><br>Signature or written consent of Co-I: ________<br>(electronic signature or email or any sort of written consent)<br>[if more than one, please copy and paste this row for additional Co-Is<br><br>Do you have ethics certification? ☐ No ☒ Yes (please attach in your CV below)<br><br>Do you have RBM training certification? ☒ No ☐ Yes (please attach the certificate with CV below)	Primary Scientific Division of the Co-I LSSD<br><br>Approved<br>________<br>Approval of the Respective Senior Director/ Programme Head<br><br>(Signature)
**Co-Investigator(s) - Internal:** Sex ☐ Female ☒ Male<br>**Dr. Md. Shafiqul Alam Sarkar**<br>(Position, phone no, extension no, cell, and email address):<br>Emeritus Scientist, NCSD Administration<br>Phone(+880-2) 9827001 -10, Ext: 2347<br>e-mail: sasarkar@icddrb.org<br><br>Signature or written consent of Co-I: ________<br>(electronic signature or email or any sort of written consent)<br>[if more than one, please copy and paste this row for additional Co-Is<br><br>Do you have ethics certification? ☐ No ☒ Yes (please attach in your CV below)<br><br>Do you have RBM training certification? ☒ No ☐ Yes (please attach the certificate with CV below)	Primary Scientific Division of the Co-I NCSD<br><br>________<br>Approval of the Respective Senior Director/ Programme Head<br><br>(Signature)
**Co-Investigator(s) – External:** Sex ☐ Female ☒ Male<br>**Address** (provide full official address, including land phone no(s), extension no. (if any), cell phone number, and email address):<br>**Andrew J Pollard FRCPCH PhD**<br>Professor of Paediatric Infection and Immunity<br>Department of Paediatrics, University of Oxford<br>Room 02-46-07, Level 2, Children's Hospital, Oxford OX3 9DU<br>Tel +44 (0)1865 234226/225956<br>www.ovg.ox.ac.uk<br><br>Signature or written consent of Co-I: E-MAIL CONSENT IS ATTACHED WITH THE PROTOCOL<br>(electronic signature or email or any sort of written consent)<br>[if more than one, please copy and paste this row for additional Co-Is]	
**Co-Investigator(s) – External:** Sex ☒ Female ☐ Male<br>**Address** (provide full official address, including land phone no(s), extension no. (if any), cell phone number, and email address):<br>**Dr. Katherine Theiss-Nyland**<br>Oxford Vaccine Group, Department of Paediatrics<br>University of Oxford<br>e-mail: katherine.theiss-nyland@paediatrics.ox.ac.uk<br><br>Signature or written consent of Co-I: E-MAIL CONSENT IS ATTACHED WITH THE PROTOCOL<br>(electronic signature or email or any sort of written consent)<br>[if more than one, please copy and paste this row for additional Co-Is]	

**Co-Investigator(s) – External:** Sex ☐ Female ☒ Male<br>Samir K. Saha<br>Professor of Microbiology; Dhaka Shishu Hospital<br>& Executive Director; Child Health Research Foundation<br>Sher-E-Banglanagar, Dhaka 1207, Bangladesh<br>Phone: 880 2 9104211 Ext. 171-180<br>Email: samirk.sks@gmail.com<br>Signature or written consent of Co-I: email consent is attached with the protocol__________________<br>(electronic signature or email or any sort of written consent)<br>[if more than one, please copy and paste this row for additional Co-Is]
**Co-Investigator(s) – External:** Sex ☐ Female ☒ Male<br>Dr. Mohammad Aminul Islam<br>Director and Project Director, Kurmitola General Hospital<br>Dhaka, Bangladesh<br>Phone: 880 01769010201<br>Email: abdullahelkafee@yahoo.com<br>Signature or written consent of Co-I: email consent is attached with the protocol__________________<br>(electronic signature or email or any sort of written consent)<br>[if more than one, please copy and paste this row for additional Co-Is]
**Co-Investigator(s) – External:** Sex ☐ Female ☒ Male<br>Dr. Md. Abdullahel Kafee<br>Assistant Professor of Medicine; Kurmitola General Hospital<br>Dhaka, Bangladesh<br>Phone: 880 01711192140<br>Email: abdullahelkafee@yahoo.com<br>Signature or written consent of Co-I: email consent is attached with the protocol__________________<br>(electronic signature or email or any sort of written consent)<br>[if more than one, please copy and paste this row for additional Co-Is]
**Co-Investigator(s) – External:** Sex ☐ Female ☒ Male<br>Dr. Md. Mubin<br>Medical Officer; Kurmitola General Hospital<br>Dhaka, Bangladesh<br>Phone: 01711059294<br>Email: mubin_dr99@yahoo.com<br>Signature or written consent of Co-I: email consent is attached with the protocol__________________<br>(electronic signature or email or any sort of written consent)<br>[if more than one, please copy and paste this row for additional Co-Is]
**Co-Investigator(s) – External:** Sex ☒ Female ☐ Male<br>Rachel Colin-Jones<br>Clinical Lead/Program Manager<br>Oxford Vaccine Group<br>CCVTM<br>Churchill Hospital<br>OX37LE<br>Phone: 01865611310<br>Email: rachel.colin-jones@paediatrics.ox.ac.uk<br>Signature or written consent of Co-I: email consent is attached with the protocol__________________<br>(electronic signature or email or any sort of written consent)<br>[if more than one, please copy and paste this row for additional Co-Is]

**Co-Investigator(s) – External:** Sex ☒ Female ☐ Male<br>Dr. Farhana Noman<br>Senior Consultant; Kurmitola General Hospital<br>Dhaka, Bangladesh<br>Phone: 01715261000<br>Email: drfarhananoman@gmail.com<br>Signature or written consent of Co-I: email consent is attached with the protocol__________________<br>(electronic signature or email or any sort of written consent)<br>[if more than one, please copy and paste this row for additional Co-Is]	
**Co-Investigator(s) – External:** Sex ☐ Female ☒ Male<br>Dr Xinxue Liu<br>Senior Statistician<br>Oxford Vaccine Group<br>CCVTM<br>Churchill Hospital<br>OX37LE<br>Phone: 01865611350<br>Email: xinxue.liu @paediatrics.ox.ac.uk<br>Signature or written consent of Co-I: email consent is attached with the protocol__________________<br>(electronic signature or email or any sort of written consent)<br>[if more than one, please copy and paste this row for additional Co-Is]	
**Co-Investigator(s) – External:** Sex ☒ Female ☐ Male<br>Dr Merryn Voysey<br>Lead Statistician<br>Oxford Vaccine Group<br>CCVTM<br>Churchill Hospital<br>OX37LE<br>Phone: 01865611416<br>Email: merryn.voysey@paediatrics.ox.ac.uk<br>Signature or written consent of Co-I: email consent is attached with the protocol__________________<br>(electronic signature or email or any sort of written consent)<br>[if more than one, please copy and paste this row for additional Co-Is]	
**Student Investigator(s) - Internal:** Sex ☐ Female ☐ Male<br><br>(Position, phone no, extension no, cell, and email address **):**<br><br>Signature or written consent of Student Investitor: __________________<br>(electronic signature or email or any sort of written consent)<br><br>Have ethics certificate? ☐ No ☐ Yes (If Yes, please attach to your CV below)	Students Affiliation<br><br>___________________________<br>Approval of the Respective Senior Director/ Programme Head<br><br>(Signature)
**Student Investigator(s) - External:** Sex ☐ Female ☐ Male<br>Address (provide full official address, including land phone no(s), extension no. (if any), cell phone number, and email address):<br><br>Signature or written consent of Student Investitor: __________________<br>(electronic signature or email or any sort of written consent)	

Collaborating Institute(s): Please provide full official address

Institution # 1

Country	United Kingdom
Contact person	Andrew J Pollard FRCPCH PhD<br>Professor of Paediatric Infection and Immunity
Department<br>(including Division, Centre, Unit)	Department of Paediatrics
Institution<br>(with official address)	University of Oxford<br>Room 02-46-07<br>Level 2, Children's Hospital<br>Oxford OX3 9DU<br>Tel +44 (0)1865 234226/225956<br>www.ovg.ox.ac.uk
Directorate<br>(in case of GoB i.e. DGHS)	
Ministry (in case of GoB)	

Institution # 2

Country	Bangladesh
Contact person	Samir K. Saha
Department<br>(including Division, Centre, Unit)	
Institution<br>(with official address)	Child Health Research Foundation<br>Sher-E-Banglanagar, Dhaka 1207, Bangladesh
Directorate<br>(in case of GoB i.e. DGHS)	
Ministry (in case of GoB)	

Institution # 3

Country	Bangladesh
Contact person	Dr. Mohammad Aminul Islam
Department<br>(including Division, Centre, Unit)	
Institution<br>(with official address)	Kurmitola General Hospital<br>Dhaka, Bangladesh
Directorate<br>(in case of GoB i.e. DGHS)	
Ministry (in case of GoB)	

Note: If less than or more than three collaborating institutions, please delete or insert blocks as needed.

Contribution by the Members of the Scientific Team:

Members' Name	Contribution								
	Research idea/ concept	Study design	Protocol writing	Respond to external reviewers' comments	Defending at IRB	Developing data collection Tool(s)	Data Collection	Data analysis/ interpretation of results	Manuscri writing
Dr. Firdausi Qadri	☒	☒	☒	☒	☒	☐	☐	☒	☒
Dr. John D Clemens	☒	☒	☒	☒	☐	☐	☐	☒	☒
Dr. Farhana Khanam	☐	☐	☒	☒	☒	☒	☒	☒	☒
Dr. Md. Arifuzzaman Khan	☐	☐	☒	☒	☒	☒	☒	☒	☒
Dr. Fahima Chowdhury	☐	☐	☒	☒	☒	☒	☒	☒	☒
Dr. Ashraful Islam Khan	☐	☐	☒	☒	☒	☒	☒	☒	☒
Dr. Md. Khalequzzaman	☐	☐	☐	☐	☒	☐	☐	☒	☒
Dr. Niyaz Ahmed Abdus Samad	☐	☐	☐	☐	☒	☐	☐	☒	☒
Dr. Md. Shafiqul Alam Sarkar	☐	☐	☐	☐	☒	☐	☐	☒	☒

Study Population: Sex, Age, Special Group and Ethnicity

Research Subject:

☒ Human
☐ Animal
☐ Microorganism
☐ Other (specify): ______________

Sex:

☒ Male
☒ Female
☒ Transgender

Age:

☒ 0 – 4 years
☒ 5 – 10 years
☒ 11 – 17 years
☐ 18 – 64 years
☐ 65 +

Special Group:

☐ Pregnant Women
☐ Fetuses
☐ Prisoners
☐ Destitutes
☐ Service Providers
☐ Cognitively Impaired
☐ CSW
☐ Expatriates
☐ Immigrants
☐ Refugee
☐ Others (specify): _________________

Ethnicity:

☒ No ethnic selection (Bangladeshi)
☐ Bangalee
☐ Tribal group
☐ Other (specify): _________________

NOTE: It is icddr.b's policy to include men, women, children and transgender in its research projects involving participation of humans, unless there is strong justification(s) for their exclusion.

Consent Process: (Check all that apply)

☒ Written
☒ Oral
☐ Audio
☐ Video
☐ None

Language:

☒ Bangla
☒ English
☐ Other (specify): ________________

Project/Study Site: (Check all that apply)

- ☐ Chakaria
- ☐ Bandarban
- ☐ Dhaka Hospital
- ☐ Kamalapur Field Site/HDSS
- ☒ Mirpur (Dhaka)
- ☐ Matlab DSS Area
- ☐ Matlab non-DSS Area
- ☐ Matlab Hospital
- ☐ Mirzapur
- ☐ Bianibazar (Sylhet)
- ☐ Kanaighat (Sylhet)
- ☐ Jakigonj (Sylhet)
- ☐ Other community in Dhaka
  Name: ____________________________________
- ☐ Other sites in Bangladesh
  Name: ____________________________________
- ☐ Multi-national Study
  Name of the country________________________

Project/Study Type: (Check all that apply)

- ☐ Case Control Study
- ☒ Clinical Trial (Hospital/Clinic/Field)*
- ☐ Community-based Trial/Intervention
- ☐ Cross Sectional Survey
- ☐ Family Follow-up Study
- ☐ Longitudinal Study (cohort or follow-up)
- ☐ Meta-analysis
- ☐ Programme Evaluation
- ☐ Programme (Umbrella Project)
- ☐ Prophylactic Trial
- ☐ Record Review
- ☐ Secondary Data Analysis
  Protocol No. of Data Source: ______________
- ☐ Surveillance/Monitoring
- ☐ Systematic Review
- ☐ Other (specify): ___________________________

***Note:** International Committee of Medical Journal Editors (ICMJE) defines Clinical Trial as "*Any research project that prospectively assigns human participants to intervention and comparison groups to study the cause-and-effect relationship between a medical intervention and a health outcome*".

PI of the RRC- and ERC-approved Clinical Trials should provide necessary information to IRB Secretariat (Research Administration) for registration and uploading into relevant websites (usually at the https://register.clinicaltrials.gov/). They should also provide relevant information to the IRB Secretariat in the event of amendment/modification after their approval by RRC and ERC.

Biological Specimen:

a) Will the biological specimen be stored for future use?	☒ Yes ☐ No ☐ Not applicable
b) If the response is 'yes', how long the specimens will be preserved?	____05______ years
c) What types of tests will be carried out with the preserved specimens?	Microbiological, Immunological and genetic tests.
d) Will the consent be obtained from the study participants for use of the preserved specimen for other initiative(s) unrelated to this study, without their re-consent?	☒ Yes ☐ No ☐ Not applicable
e) Will the specimens be shipped to other country/ countries? If yes, name of institution(s) and country/countries.	☒ Yes ☐ No ☐ Not applicable<br>Oxford Vaccine Group, Department of Paediatrics, University of Oxford, United Kingdom<br>University of Melbourne, Australia<br>Wellcome Trust Sanger Institute, United Kingdom<br>______________________________
f) If shipped to another country, will the surplus/unused specimen be returned to icddr,b? If the response is 'no', then the surplus/unused specimen must be destroyed.	☐ Yes ☒ No ☐ Not applicable
g) Who will be the custodian of the specimen at icddr,b?	Firdausi Qadri
h) Who will be the custodian of the specimen when shipped outside Bangladesh?	Andrew J Pollard
i) Who will be the owner(s) of the specimens?	icddr,b

j) Has a MoU been signed with regards to collection, storage, use and ownership of specimen?<br>If the response is 'yes', please attach a copy of the MoU..<br>If the response is 'no', appropriate justification should be provided for not signing a MoU.	☒ Yes ☐ No ☐ Not applicable<br><br>The Academic Collaborative Agreement with the University of Oxford is attached with the protocol.<br><br>The MoU with the new collaborating institutes is under process. We will submit the MoU to the IRB.

Proposed Sample Size:
Sub-group (Name of subgroup e.g. Men, Women) and Number

Name	Number	Name	Number
(1) Children (9 months to <16 years) eligible for vaccination	54,188	(3)	
(2) Children (9 months to <16 years) eligible for vaccination in the inner clusters	43,350	(4)	
		**Total sample size**	54,188

Determination of Risk: Does the Research Involve (Check all that apply**)**

☐ Human exposure to radioactive agents?
☐ Foetal tissue or abortus?
☐ Investigational new device?
Specify: ___________________
☐ Existing data available from Co-investigator?

☐ Human exposure to infectious agents?
☒ Investigational new drug?
☐ Existing data available via public archives/sources?
☒ Pathological or diagnostic clinical specimen only?
☐ Observation of public behaviour?
☐ New treatment regime?

	Yes	No
Will the information be recorded in such a manner that study participants can be identified from the information directly or through identifiers linked to the study participants?	☒	☐
Does the research deal with sensitive aspects of the study participants' sexual behaviour, alcohol use or illegal conduct such as drug use?	☐	☒

Could information on study participants, if available to people outside of the research team:

	Yes	No
a) Place them at risk of criminal or civil liability?	☐	☒
b) Damage their financial standing, reputation or employability, or social rejection, or lead to stigma, divorce etc.?	☐	☒

Do you consider this research: (check one)

☐ Greater than minimal risk	☒ No more than minimal risk	☐ Only part of the diagnostic test

Note: Minimal Risk: The probability and the magnitude of the anticipated harm or discomfort to participants is not greater than those ordinarily encountered in daily life or during the performance of routine physical, psychological examinations or tests, e.g. the risk of drawing a small amount of blood from a healthy individual for research purposes is no greater than when the same is performed for routine management of patients.

**Risk Group of Infectious Agent and Use of Recombinant DNA**	
a) Will specimens containing infectious agent be collected?	☒ Yes ☐ No ☐ Not applicable
b) Will the study involve amplification by culture of infectious agents?	☒ Yes ☐ No ☐ Not applicable

d) Does the study involve experiments with recombinant DNA?	☐ Yes	☒ No	☐ Not applicable

Does the study involve any biohazards materials/agents or microorganisms of risk group 2, 3, or 4 (GR2, GR-3 or GR4)?

☒ Yes ☐ No

[If the response is 'yes'] I, (print name of the PI) affirm that we will use the standard icddr,b laboratory procedures for biosafety of the hazardous materials/agents or microorganisms in the conduction of the study.

23/11/2017

Signature of the Principal Investigator **Date**

Dissemination Plan: [please explicitly describe the plans for dissemination, including how the research findings would be shared with stakeholders, identifying them if known, and the mechanism to be used; anticipated type of publication (working papers, internal (institutional) publication, international publications, international conferences/seminars/workshops/ agencies. [Check all that are applicable]

**Dissemination type**	**Response**		**Description (if the response is a yes)**
Seminar for icddr,b scientists/ staff	☐ No	☒ Yes	Will be shared with icddr,b scientists and staff through centers' scientific forum.
Internal publication	☐ No	☒ Yes	Will be published in icddr,b publication hub and seminar.
Working paper	☒ No	☐ Yes	
Sharing with GoB (e.g. DGHS/ Ministry, others)	☒ No	☐ Yes	
Sharing with national NGOs	☒ No	☐ Yes	
Presentation at national workshop/ seminar	☐ No	☒ Yes	Findings of the study will be disseminated through meeting and seminar in the form of presentation
Presentation at international workshop/ conference	☐ No	☒ Yes	In the event of a presentation at an international workshop, icddr,b and other consortium member will collaborate accordingly on all presentation materials including power point slides and/or submitted abstract
Peer-reviewed publication	☐ No	☒ Yes	Potential publications will be written collaboratively between icddr,b and other consortium members; It will require draft manuscripts be reviewed internally prior to journal submission.
Sharing with international agencies	☒ No	☐ Yes	
Sharing with donors	☐ No	☒ Yes	icddr,b and other members of the consortium will disseminate generated data and findings as appropriate to Donors (BMGF)
Policy brief	☒ No	☐ Yes	
Other			
Other			

Funding:

Is the protocol fully funded?	☒ Yes	☐ No
If the answer is yes, please provide sponsor(s)'s name	1. Award from Bill and Melinda Gates Foundation (BMGF) to the University of Maryland, Baltimore, with the University of Oxford as a collaborating institution	
	2.	
Is the protocol partially funded?	☐ Yes	☒ No
If the answer is yes, please provide sponsor(s)'s name	1.	
	2.	

If fund has not been identified:

Is the proposal being submitted for funding?	☐ Yes	☐ No
If yes, name of the funding agency	1.	
	2.	

Conflict of interest:
Do any of the participating investigators and/or member(s) of their immediate families have an equity relationship (e.g. stockholder) with the sponsor of the project or manufacturer and/or owner of the test product or device to be studied or serve as a consultant to any of the above?

☒ No ☐ Yes (please submit a written statement of disclosure to the Executive Director, icddr,b)

Proposed Budget:

Dates of Proposed Period of Support

(Day, Month, Year - DD/MM/YY)

Beginning Date : March 2018

End Date : February 2022

Cost Required for the Budget Period ($)

Years	Direct Cost	Indirect Cost	Total Cost
Year-1	1,633,212	244,982	1,878,193
Year-2	2,165,532	324,830	2,490,362
Year-3	2,011,250	301,687	2,312,938
Year-4	1,296,547	194,482	1,491,030
Year-5			0
Total	7,106,541	1,065,981	8,172,522

Certification by the Principal Investigator:

I certify that the statements herein are true, complete and accurate to the best of my knowledge. I am aware that any false, fictitious, or fraudulent statements or claims may subject me to criminal, civil, or administrative penalties. I agree to accept the responsibility for the scientific conduct of the project and to provide the required progress reports including updating protocol information in the NAVISION if a grant is awarded as a result of this application.

I also certify that I have read icddr,b Data Policies and understand the PIs' responsibilities related to archival and sharing of research data, and will remain fully compliant to the Policies. (Note: The Data Policies can be found here: http://www.icddrb.org/who-we-are/data-policies)

F Qadri 21/1/2018
Signature of PI

F Qadri 21/1/2018
Date

Approval of the Project by the Division Director of the Applicant:

The above-mentioned project has been discussed and reviewed at the Division level.

Dr Firdausi Qadri
Name of the Division Director

F Qadri 21/1/18
Signature

21/1/2018
Date of Approval

Table of Contents

RRC APPLICATION FORM 1
Project Summary 15
Hypothesis to be tested: 18
Specific Objectives: 18
Background of the Project including Preliminary Observations: 19
Research Design and Methods 24
Sample Size Calculation and Outcome (Primary and Secondary) Variable(s) 39
Data Analysis 41
Data Safety Monitoring Plan (DSMP) 43
Ethical Assurance for Protection of Human rights 44
Use of Animals 43
Collaborative Arrangements 43
Facilities Available 44
Literature Cited 474
Budget .... **Error! Bookmark not defined.**
Other Support 49
Biography of the Investigators 52
Gender Analysis Tool 103
Appendices including consent forms 104
Response to the comments of external reviewers 184
Check-List 185
Abbreviation 186

☒ Check here if appendix is included

Project Summary

[The summary, within a word limit of 300, should be stand alone and be fully understandable.]

Principal Investigator: Dr Firdausi Qadri
Research Protocol Title: Assessing the impact of a Vi-Polysaccharide Conjugate Vaccine in preventing typhoid infection among Bangladeshi children – a Phase IIIb trial
Proposed start date: March, 2018 Estimated end date: March 2023

Background (brief):

a. Burden:

Enteric fever is a systemic illness caused by the human restricted pathogens *Salmonella enterica* serotypes Typhi (*S*. Typhi) and Paratyphi A, B, C. It is estimated to affect >20 million people worldwide annually, with an estimated 200,000 fatalities per annum, primarily in lower income countries with poor sanitation. Areas with an incidence of >100/100,000 are considered endemic, including many countries in Africa, South Asia, South-East Asia and Central Asia. The burden of disease and mortality is increasingly recognised in under 5 years of age group, as well as in older children and young adults. Enteric fever also remains a concern in developed countries for travellers to endemic regions and laboratory workers.

b. Knowledge gap:

Control of enteric fever, historically, has been established primarily through improved sanitation and infrastructure, leading to the elimination of disease as a public health problem from most developed countries. This remains the case, but there are substantial costs and difficulties implementing these measures in high typhoid incidence areas. Since the available vaccines are not effective in children, the conjugate vaccines have been tested and found safe and immunogenic. It is important to evaluate the effectiveness of the conjugate vaccines to reduce the typhoid fever, especially in children.

c. Relevance:

An effective vaccination programme in the higher risk populations will be useful to apply the cost-effective control measures. Given the causative organisms are human-restricted, global eradication is possible and an effective vaccine will contribute to this.

Hypothesis (if any):

The Vi-Polysaccharide Conjugate Vaccine will be safe and effective in preventing typhoid infection among Bangladeshi children.

Objectives:

Primary:

a) To determine the relative and absolute rate reduction of symptomatic infection caused by *S*. Typhi in recipients of Vi-TCV (total vaccine protection)
b) To determine the relative and absolute rate reduction of symptomatic infection caused by *S*. Typhi among all residents in the clusters allocated to Vi-TCV (overall vaccine protection)

Secondary:

1. To investigate safety outcomes associated with Vi-TCV vaccination, within the study population
2. To determine the relative and absolute rate reduction of symptomatic infection caused by *S*. Typhi in non-recipients of Vi-TCV (indirect vaccine protection)
3. To determine the impact of vaccination with Vi-TCV on the incidence of inpatient/outpatient admission rates for fever and abdominal discomfort among vaccinees
4. To determine the impact of vaccination with Vi-TCV on the incidence of inpatient/outpatient admission rates for fever and abdominal discomfort among all residents of the clusters
5. To determine the impact of vaccination with Vi-TCV on the incidence of inpatient/outpatient

admission rates for clinical typhoid fever among vaccinees

6. To determine the impact of vaccination with Vi-TCV on the incidence of inpatient/outpatient admission rates for clinical typhoid fever among all residents of the clusters
7. To determine the effectiveness of and rate reduction by Vi-TCV in preventing blood culture-confirmed symptomatic infection caused by *S*. Paratyphi in recipients of the vaccine (total vaccine protection)
8. To assess the change of Vi-TCV vaccine protection in the medium-term compared with the initial 2-year after vaccination
9. To determine the persistence of antibodies induced by Vi-TCV in stratified age groups
10. To determine the sero-incidence of typhoid fever and to assess the sero-efficacy of Vi-TCV

Exploratory:

1. To measure differences in all-cause hospitalization rates by treatment arm
2. To determine the immunogenicity of Vi-TCV in a subset of participants, stratified by age groups
3. To determine the persistence of antibodies induced by Vi-TCV in stratified age groups
4. To determine the incidence of absenteeism from school/work as a result of typhoid infection among vaccinees, by treatment arm
5. To determine the incidence of absenteeism from work/school as a result of typhoid infection among all cluster residents by treatment arm
6. To measure all-cause mortality, mortality with fever, and mortality with fever and abdominal pain among vaccinees by treatment arm
7. To measure all-cause mortality, mortality with fever, and mortality with fever and abdominal pain among all cluster residents by treatment arm
8. To measure the rate of surgical intervention for acute abdominal complaints by treatment arm among vaccinees by treatment arm
9. To measure the rate of surgical intervention for acute abdominal complaints by treatment arm among all cluster residents by treatment arm

Methods:

This is a multi-site phase IIIb, parallel, cluster-randomized, controlled trial of typhoid Vi-Polysaccharide Conjugate Vaccine. The vaccine will be tested among children of 9 months to <16 years of age residing in Mirpur, Dhaka. The Japanese Encephalitis vaccine will be used as the control. Before vaccination a census in the study area will be conducted to enumerate the study population. The census will be updated biannually. 150 clusters of 1250 people will be randomized in a 1:1 ratio to allocate Vi-TCV or control vaccine. At six month intervals during the two years after baseline, census updates of the population will be done in all clusters, and children fulfilling the eligibility criteria for participation who have not received the study vaccine allocated to the cluster will be offered the vaccine. At the end of the two years follow-up after baseline ~~the study~~ children in the control group will be offered the Vi-TCV. After vaccination of the control group with Vi-TCV, the study will follow-up the study population for another two years with census updates of the population at six month intervals. study children in the control group will be offered the Vi-TCV. Adverse events following vaccination will be reported up to 7 days post vaccination. Serious adverse events (SAEs), including serious adverse reactions (SARs) will be reported for 30 days post vaccination, after which only SARs will be reported for the duration of the trial. Passive surveillance for typhoid fever will carried out in different health facilities in the catchment area of the study population. Independent safety monitors will be individuals not associated with study operations and who have clinical trials and infectious disease experience. All study update including adverse events and serious adverse events, as described above will be reported to the local (icddr,b) and international Data Safety Monitoring Board (DSMB).

Outcome measures/variables:

Primary:

a) The incidence of blood culture confirmed typhoid fever in vaccinees in intervention clusters compared to control clusters.
b) The incidence of blood culture confirmed typhoid fever in all residents of the intervention clusters compared to control clusters

Secondary:

1. The proportion of participants developing adverse events within the first 7 days post-vaccination, and serious adverse events within 6 months of vaccination, as determined through sub-sample at 7 days, and self-reporting at follow-up contact
2. Rates of participants with a history of ≥2 days of persistent fever, and/or a temperature of ≥38 degrees C upon presentation, together with abdominal discomfort, in vaccinees in intervention clusters compared to control clusters
3. Rates of participants with a history of ≥2 days of persistent fever, and/or a temperature of ≥38 degrees C upon presentation, together with abdominal discomfort, among all residents of the Vi-TCV clusters compared to the control vaccine clusters
4. Rates of patients with clinical diagnoses of typhoid fever in vaccinees in intervention clusters compared to control clusters
5. Rates of patients with clinical diagnoses of typhoid fever among all residents of the Vi-TCV clusters compared to the control vaccine clusters
6. The incidence of blood culture confirmed paratyphoid fever in vaccinees in intervention clusters compared to control clusters
7. The incidence of blood culture confirmed typhoid fever in vaccinees in previously Vi-TCV vaccinated clusters (original Vi-TCV clusters) compared with recently Vi-TCV vaccinated clusters (original control clusters)
8. Assay of anti-Vi IgG in blood samples collected at baseline, 28 day, 12 months, 18 months, 24 months post baseline vaccination, and 12 months and 24 months post unblinding visit in a subset of participants receiving Vi-TCV and control vaccine
9. Assay of antibodies against the typhoid toxin (CdtB) in blood samples collected at baseline, 28 day, 12 months, 18 months, 24 months post baseline vaccination, and 12 months and 24 months post unblinding visit in a subset of participants receiving Vi-TCV and control vaccine

Exploratory:

1. Incidence of all cause hospitalization in community-based surveys of all residents of the Vi-TCV clusters compared to the control vaccine clusters
2. Assay of anti-Vi antibodies in blood samples collected at baseline (Day 0), at one month (Day 28), in a subset of participants from each treatment arm
3. Assay of anti-Vi IgG antibodies in blood samples collected at baseline (day 0), 18 months (day 545) and two years (day 730) in a subset of participants receiving intervention and control vaccinations
4. Incidence of absenteeism from school/work in vaccinated children and their guardians as a result of confirmed typhoid infection in intervention clusters compared to control clusters
5. Incidence of absenteeism from school/work in all cluster residents and their guardians/carers (if applicable) as a result of culture confirmed typhoid infection in intervention clusters compared to control clusters
6. Rates and circumstances of mortality, recorded from hospital records and verbal autopsies via six-monthly census updates, in vaccinees in intervention clusters compared to control clusters
7. Rates and circumstances of mortality, recorded from hospital records and verbal autopsies via six-monthly census updates, in all residents of the Vi-TCV clusters compared to the control vaccine clusters
8. Rates of acute abdominal surgery, and of surgery for intestinal perforation, in vaccinees in intervention clusters compared to control clusters
9. Rates of acute abdominal surgery, and of surgery for intestinal perforation, in all residents of the Vi-TCV clusters compared to the control vaccine clusters

Description of the Research Project

Hypothesis to be tested:

In a hypothesis testing research proposal, briefly mention the hypothesis to be tested and provide the scientific basis of the hypothesis, critically examining the observations leading to the formulation of the hypothesis.

Does this research proposal involve testing of hypothesis: ☐ No ☒ Yes (describe below)

The typhoid Vi-polysaccharide conjugate vaccine will be safe and effective in preventing typhoid fever among Bangladeshi children.

Specific Objectives:

Describe the specific objectives of the proposed study. State the specific parameters, gender aspects, biological functions, rates, and processes that will be assessed by specific methods.

Primary:

a) To determine the relative and absolute rate reduction of symptomatic infection caused by *S*. Typhi in recipients of Vi-TCV (total vaccine protection)
b) To determine the relative and absolute rate reduction of symptomatic infection caused by *S*. Typhi among all residents in the clusters allocated to Vi-TCV (overall vaccine protection)

Secondary:

1. To investigate safety outcomes associated with Vi-TCV vaccination, within the study population
2. To determine the relative and absolute rate reduction of symptomatic infection caused by *S*. Typhi in non-recipients of Vi-TCV (indirect vaccine protection)
3. To determine the impact of vaccination with Vi-TCV on the incidence of inpatient/outpatient admission rates for fever and abdominal discomfort among vaccinees
4. To determine the impact of vaccination with Vi-TCV on the incidence of inpatient/outpatient admission rates for fever and abdominal discomfort among all residents of the clusters
5. To determine the impact of vaccination with Vi-TCV on the incidence of inpatient/outpatient admission rates for clinical typhoid fever among vaccinees
6. To determine the impact of vaccination with Vi-TCV on the incidence of inpatient/outpatient admission rates for clinical typhoid fever among all residents of the clusters
7. To determine the effectiveness of and rate reduction by Vi-TCV in preventing blood culture-confirmed symptomatic infection caused by *S*. Paratyphi in recipients of the vaccine (total vaccine protection)
8. To assess the change of Vi-TCV vaccine protection in the medium-term compared with the initial 2-year after vaccination
9. To determine the persistence of antibodies induced by Vi-TCV in stratified age groups
10. To determine the sero-incidence of typhoid fever and to assess the sero-efficacy of Vi-TCV

Exploratory:

1. To measure differences in all-cause hospitalization rates by treatment arm
2. To determine the immunogenicity of Vi-TCV in a subset of participants, stratified by age groups
3. To determine the persistence of antibodies induced by Vi-TCV in stratified age groups
4. To determine the incidence of absenteeism from school/work as a result of typhoid infection among vaccinees by treatment arm
5. To determine the incidence of absenteeism from work/school as a result of typhoid infection among all cluster residents by treatment arm
6. To measure all-cause mortality, mortality with fever, and mortality with fever and abdominal pain among vaccinees by treatment arm

7. To measure all-cause mortality, mortality with fever, and mortality with fever and abdominal pain among all cluster residents by treatment arm
8. To measure the rate of surgical intervention for acute abdominal complaints by treatment arm among vaccinees by treatment arm
9. To measure the rate of surgical intervention for acute abdominal complaints by treatment arm among all cluster residents by treatment arm

Background of the Project including Preliminary Observations:

Provide scientific validity of the hypothesis based on background information of the proposed study and discuss previous works on the research topic, including information on sex, gender and diversity (ethnicity, SES) by citing specific references. Critically analyze available knowledge and discuss the questions and gaps in the knowledge that need to be filled to achieve the proposed aims. If there is no sufficient information on the subject, indicate the need to develop new knowledge.

Enteric fever is a systemic illness caused by the human restricted pathogens *Salmonella enterica* serotypes Typhi (*S*. Typhi) and Paratyphi A-C. It is estimated to affect >20 million people worldwide annually, with an estimated 200,000 fatalities per annum, primarily in lower income countries with poor sanitation(1). Areas with an incidence of >100/100,000 are considered endemic, including many countries in Africa, South Asia, South-East Asia and Central Asia(2). The burden of disease and mortality is increasingly recognised in the under 5 age group, as well as in older children and young adults(3–6). Enteric fever also remains a concern in developed countries for travellers to endemic regions and laboratory workers (7,8).

Control of enteric fever, historically, has been established primarily through improved sanitation and infrastructure, leading to the elimination of disease as a public health problem from most developed countries. This remains the case, but there are substantial costs and difficulties implementing these measures in high typhoid incidence areas. As such, use of an effective vaccination programme targeting the highest risk populations will likely be a useful and cost-effective addition to control measures. Given the causative organisms are human-restricted, global eradication is possible and an effective vaccine will contribute to this.

Typhoid Vaccines

Currently, licensed vaccines exist only for the most prevalent serovar causing enteric fever, *S.* Typhi. The existing options are as follows:

Inactivated Whole Cell vaccine

This vaccine consists of heat-phenol-inactivated whole cell *S*. Typhi, which is injected subcutaneously in two doses four weeks apart. It had efficacy of 51-67% in controlled trials. It was associated with high degree of reactogenicity, causing fever and systemic symptoms in 9-34% or recipients leading to school absence in 2-17% of cases (9). Due to these side-effects it has largely dropped out of mainstream use, however it is still used in several developing countries.

Vi Polysaccharide Vaccine (Vi-PS)

Developed in the 1980s this vaccine consists of purified Virulence factor (Vi antigen) capsular polysaccharide (Vi-PS) that forms the capsule of, and is specific to, *S.* Typhi. It elicits a T-cell independent antibody response, which means it has poor immunological memory and repeat doses do not result in an additional boosting response (10,11).
Similar to other polysaccharide vaccines, Vi-PS vaccine is poorly immunogenic and not licensed for use in children under 2 years old, presumably due to the absence of specific splenic marginal zone B-cells that are needed to produce an immunological response to polysaccharides. In clinical trials, clinical protection is non-comprehensive with protective efficacies of 64-72% (12–14). Additionally, protection is short lived, lasting only 2-3 years (15,16).

Live attenuated oral vaccine (Ty21a)

Also developed in the 1980s, this is an attenuated strain of *S*. Typhi (Ty21a) that has had many virulence genes mutated chemically, including the gene leading to failure to produce the Vi antigen. Ingestion of this strain induces local gut mucosal immunity as well as systemic antibody and cell mediated response(17,18). The strain is lyophilised and administered in either an oral enteric capsule or a liquid solution and requires 3-4 doses to induce effective protective immunity.

Clinical trials preformed in Chile and Indonesia demonstrated Ty21a vaccine had a protective efficacy of 67% and 53%, respectively (5,19). While the enteric-coated formulation is difficult to administer to young children, the alternative liquid formulation is better tolerated but may be less immunogenic in younger children (18,20). Ty21a vaccine is not licensed for children under the age of 6 years.

Vi-rEPA Vaccine

This is a vaccine was developed by the US National Institute for Health (US NIH) in 1994 utilising Vi-polysaccharide conjugated with a recombinant exoprotein A from *Pseudomonas aeruginosa* (rEPA) (21). A two-dose schedule six weeks apart was shown to be highly immunogenic with a protective efficacy of 91.1% in children aged 2 to 5 years in a trial in Vietnam (22). More recently, a study has demonstrated its immunogenicity in infants (23). However, the licensure of Vi-rEPA has been delayed due to lack of regulatory precedent for the use of rEPA carrier based vaccines.

Vi antigen typhoid conjugate vaccine (Vi-TCV)

Vi-TCV (Tybar-TCV™) is a newly available vaccine developed by Bharat Biotech consisting of 25 µg of Vi polysaccharide antigen conjugated to a nontoxigenic tetanus toxoid carrier protein. Similar to other vaccines which are designed to protect against encapsulated bacterial pathogens and are conjugated to tetanus toxoid carrier proteins, Vi-TCV induces a T-cell dependent response. It can therefore produce an immunogenic response in infants under 2 years of age and has the potential to generate a durable immune responses via induction of immunological memory.

A Phase III randomised controlled trial comparing Vi-TCV with Vi-PS demonstrated seroconversion to anti-Vi IgG in the 6 month to 2 year age group (24). Additionally, a comparison of the sub-groups receiving boosters of either vaccine at two years demonstrated significantly higher anti-Vi IgG titres in the Vi-TCV group compared to the Vi-PS group (titres of 1685.3 EU/ml [95% CI: 1468-1797] in Vi-TCV vs 445.6 EU/ml [95% CI: 323-615] in Vi-PS). Safety data from the same study demonstrated that Vi-TCV was well tolerated by all age groups and that there were no differences in the number or variety of adverse events reported between the vaccine arms (25).

Efficacy data are available from a recent study performed using the typhoid challenge model at the University of Oxford (26). This study measured the efficacy of single-dose Vi-TCV, Vi-PS or a control vaccine in protecting against the development of typhoid infection after oral challenge. The study was conducted in healthy, UK adult volunteers and challenge was performed 28 days after vaccination. Using a composite diagnostic endpoint of clinical and/or microbiologically confirmed typhoid fever, the Vi-TCV and Vi-PS vaccines demonstrated comparable protective efficacy of 54.6% [95% CI: 26.8 – 71.8%] and 52.0% [95%CI: 23.2-70.0%], respectively, when compared to the control vaccine (26). This calculated Vi-TCV vaccine efficacy of 54.6% likely underestimates the protective effect of Vi-TCV in endemic settings. When applying a definition of typhoid fever which more closely approximates diagnosis in health care settings, i.e. fever followed by confirmatory bacteraemia, in a post-hoc analysis the protective efficacy of Vi-TCV vaccine increased to 87.1% [95%CI: 44.2-96.9%] compared to 52.3% [95%CI: -4.2%, 78.2%] for the unconjugated Vi-PS vaccine (26).

While Tybar-TCV is licensed for use in India and Nepal, and the data from seroconversion and efficacy studies are strong, field impact studies for Vi-TCV, demonstrating a reduction in the burden of disease attributable to typhoid infections, have not yet been conducted.

Description of TyVAC

This Vi-TCV trial falls within a larger multi-institution collaboration, called The Typhoid Vaccine Acceleration Consortium (TyVAC). TyVAC is a Bill and Melinda Gates Foundation funded project to generate evidence for Vi-TCV vaccine impact, and accelerate use of Typhoid Conjugate Vaccines in countries with significant typhoid burden. Managed by University of Maryland, in collaboration with University of Oxford, and PATH international, the TyVAC programme includes vaccination trials, health economics studies, country preparedness support for routine vaccine introduction, and the collation and synthesis of typhoid research and evidence.

Three sites have been identified for parallel field impact studies; Kathmandu, Nepal; Dhaka, Bangladesh; and Blantyre, Malawi. Each represents a geographical setting where enteric fever is endemic and has a substantial local burden of disease. In each site, independent studies with differing study designs will be implemented to identify a range of impact scenarios. Between the sites, there is a range of demographic and geographic variation to give confidence in the generalisability of the study results. The trial presented here, is one of these three studies.

Aim of the Project

This study aims to assess the impact of Vi-TCV in a field setting in order to inform and support the use of the vaccine as a control measure for enteric fever in endemic settings, to reduce global morbidity and mortality. Vi-TCV has shown promise from existing studies; it can produce seroconversion in infants; it potentially produces long lasting immunity; and it is efficacious in a controlled challenge setting. As such, it is an obvious candidate to test in a field impact study.

Rationale for Mirpur, Dhaka as the study site

Dhaka is the selected trial site for the following reasons:

- Enteric fever is endemic to Bangladesh, with a documented high incidence in Dhaka.
- Enteric fever is recognised locally as a public health concern, both within the Ministry of Health and the local community.
- The Ministry of Health is receptive to impact studies and subsequent vaccination introduction;
- The Strategic Typhoid Alliance Across Africa and Asia (STRATAA), funded by Wellcome Trust and the Bill and Melinda Gates Foundation, is a typhoid surveillance study, already running in Mirpur, Dhaka, allowing for lower costs and potential synergy (27);
- STRATAA has identified that there is a sufficiently large population of children aged 9 months to <16 years within which to conduct this vaccination trial.

Rationale for Study Design

A cluster-randomised controlled trial will be performed with a two-year follow-up to assess the protective impact of the Vi-TCV vaccine, and another two-year follow-up to assess the change in medium term vaccine protection. A cluster-randomised design has been selected in order to assess both the individual-level impact (on vaccinees) and population-level impact (on both vaccinees and non-vaccinees, including herd protection) of vaccination of children aged 9 months to <16 years of age living in the geographically defined catchment area in the Mirpur area in urban Dhaka, Bangladesh. This age range has been selected because children bear a substantial burden of the disease in both mortality and morbidity, and are likely the source of much of the typhoid transmission within this population. However, there is currently no effective vaccine available in the routine vaccination schedule. Therefore, this demographic group has

most to gain from vaccination with the Vi-TCV and would be the primary target for any subsequent vaccination campaign, and is also the most logical group to target for population-wide prevention of typhoid.

Rationale for Intervention vaccine

As discussed above, the Vi-TCV (Typbar-TCV®) is the most promising vaccine candidate for control of typhoid in an endemic area for the following reasons:

- One dose schedule,
- Immunogenic in children,
- Potentially prolonged immunogenicity,
- Shown to have minimal side effects.

Vi-TCV is licensed for use in India and Nepal and has been pre-qualified by the WHO in January 2018. The results of this cluster randomised trial will be used to inform country decision making for introduction of Vi-TCV into the routine expanded programme on immunisation (EPI) in Bangladesh.

Rationale for control vaccine

In this trial, the control vaccine should have the following features:

- Identical administration regime to Vi-TCV; i.e. one dose,
- Does not provide any direct protection against enteric fever,
- Provides some additional health benefit to the trial participants.

The Japanese encephalitis vaccine, SA14-14-2 JE, is WHO-prequalified and has been shown to be safe and immunogenic in Bangladesh, where Japanese encephalitis is endemic, as well as in trials in many countries. It is a single dose vaccine and is licensed for use from 9 months of age.

Potential benefits to participants

There is no routine vaccination for typhoid or Japanese encephalitis in Bangladesh. Participants in the control group, receiving the JE vaccine will have the benefit of receiving Vi-TCV at the end of the study.
For the duration of the study, all participants will have access to free and accurate health assessments and diagnostics and treatment at the icddr,b hospitals, as well as in other trial health clinics set up in the community, for all episodes of suspected typhoid fever occurring during the study. Participants will also have access to information about general health issues, through trial staff. If a participant of 4800 active post-vaccination surveillance group is diagnosed with typhoid in the follow up period, diagnosis and treatment cost will be provided by the investigators.
Additionally, the trial will help improve the understanding of the impact of the Vi-TCV vaccine on typhoid infection rates, and help guide future implementation of Vi-TCV vaccination programmes. Whilst participants would not necessarily benefit directly from this, a positive result from this trial could lead to implementation of a Vi-TCV vaccine programme in typhoid endemic areas, including Bangladesh. This would provide direct and indirect protection to both those close to the participant and those in the wider population.

Potential risks to the participants

The Vi-TCV vaccine has demonstrated a favourable safety record in the approximately 400 people vaccinated in a Phase III study (24) and the Oxford challenge study (26), with vaccination being well tolerated with no side effects above that shown by comparator Vi-PS vaccines. Post-licensure data, collected from private medical clinics and doctors' practices in India has also been used to generate safety reports and data. This, however, is the first controlled study involving so many participants and there may

be rare adverse events hereto unidentified that may become apparent in this study. While it is not anticipated for this to be the case, participants will be exposed to this potential risk. As a result, we will be conducting safety monitoring as a secondary outcome of this study.

Research Design and Methods

Describe the research design and methods and procedures to be used in achieving the specific aims of the research project. If applicable, mention the type of personal protective equipment (PPE), use of aerosol confinement, and the need for the use BSL2 or BSL3 laboratory for different part of the intended research in the methods.. Define the study population with inclusion and exclusion criteria, the sampling design, list the important outcome and exposure variables, describe the data collection methods/tools, and include any follow-up plans if applicable. Justify the scientific validity of the methodological approach (biomedical, social, gender, or environmental).

Also, discuss the limitations and difficulties of the proposed procedures and sufficiently justify the use of them.

Trial design summary

A pilot phase, prior to the main study, will individually randomize 200 children in an age stratified manner to receive either Vi-TCV or the JE vaccine. Safety data will be presented to the local DSMB prior to initiating the main cluster randomized trial. For the pilot phase, two sequential groups of children will be studied: children 3 to <16 years of age (n=100) followed by children 9 months to <3 years of age (n=100), with movement to the next phase contingent only on safety at one week post dosing. After approval by the local DSMB, the main Vi-TCV study will be initiated. Pilot study participants will also be invited to attend enteric fever passive surveillance clinics, until the end of the study. For the duration of the two years, until unblinding, pilot study participants will be encouraged to report any serious health events or hospitalisations.

The main trial will be a participant- and observer-blinded, cluster-randomized study of the typhoid conjugate vaccine (Vi-TCV), brand name: Tybar-TCV, in Bangladeshi children. The population within a selected geographical catchment area of Mirpur, Dhaka, will be offered entry into the study. The aim is to enroll at least 32,500 eligible, consenting children within the target age range (9 months to <16 years) residing in the target area at baseline. 150 Residential clusters of ca. 1250 people each will be randomised in a 1:1 ratio, with the eligible children in each cluster receiving Vi-TCV or the control vaccine (SA 14-14-2).

At six month intervals during the two years after baseline, census updates of the population will be done in all clusters, and children fulfilling the eligibility criteria for participation who have not received the study vaccine allocated to the cluster will be offered the vaccine at census updates. Final vaccination of new participants will be done at 18 months.

A subset of approximately 4800 participants will be selected on a 1:1 basis (Vi-TCV vs JE) from all 150 clusters to be contacted by telephone or in person seven (7) days after vaccination for follow-up and to record any adverse events following vaccination. The selection of these participants will be age-stratified (< 5 years vs >= 5 years of age with an allocation ratio of 1:1).

A subset of approximately 1500 participants will be selected on a 2:1 basis (Vi-TCV vs JE) to have blood samples collected at baseline (D0), at D28, at 18 months (D545), at two years (D730), post baseline vaccination, and at one and two years after unbinding, to study the immunogenicity. The selection of these participants will be age-stratified (< 5 years vs >= 5 years of age with an allocation ratio of 1:1).

Surveillance for enteric fever will be undertaken at different healthcare facilities (Appendix F) in Mirpur for all residents of the participating clusters for a minimum of one month preceding baseline and continuing for up to 2 years after unblinding, until the end of the study. In this surveillance all consenting residents from the participating clusters who present with a subjective history of ≥2 days fever and/or a temperature of ≥38°C at presentation will have data on the fever episode collected, a blood sample taken for culture and other tests (6-10 ml, depending on age), and will receive appropriate clinical management. Those with positive blood cultures will be visited at home to confirm their identity given at the treatment

center, to collect information about their illness, and to review the treatment given, and adjust as necessary, based on laboratory testing.

Participants that present to passive surveillance health care facilities with symptoms of Japanese Encephalitis infection will be tested for antibodies using an ELISA kit.

Community Health Workers (CHWs) will visit residents of all clusters bi-weekly to remind them about attending healthcare facilities and identify if any deaths or hospitalisations have occurred in all cluster residents and vaccinees. If a death or hospitalisation has occurred, verbal consent will be obtained to record the residents contact details and arrange a follow up visit. A trained member of staff will conduct either a verbal autopsy or record the details of the hospitalisation including surgeries, medications and school/work absenteeism. In order to cross-check data, at the time of the census updates, information about all deaths, births and hospitalizations since the previous survey will be collected. Verbal autopsies will be done for all cluster residents, including vaccinees who die during the trial period.

Every 6 months, vaccinated participants will have a follow-up contact to collect data on fevers, episodes of clinically diagnosed and culture confirmed typhoid, school/work absenteeism and other significant illness till unblinding of the study population.

Pregnant girls will not be vaccinated and female participants of reproductive age should have been advised not to be pregnant. If any pregnancy occurs accidentally prior to or within 3 months of vaccination will be followed up to observe the pregnancy outcomes.

Around three years after the initial vaccination campaign (due to the interruption of COVID-19 pandemic), all participants will be unblinded. At this point, pilot study participants and both control and intervention groups of the main trial will be informed of their vaccination status and have their vaccines documented on the patient record. All participants in the control group will be offered vaccination with the Vi-TCV vaccine. The study follow-up period has now been extended. A further 2 years of surveillance for enteric fever will then be conducted, with a census update at 6-month interval over the extended study period. Methods of surveillance, consent forms, questionnaire and data capturing will be the same as before.

Duration of participation is up to five years from enrolment (shorter than five years for subjects enrolled and vaccinated during the post-baseline, census updates).

The number of planned participant contacts will be as follows:

- All study participants: Unscheduled contacts to collect verbal autopsy and hospitalization data
- All study participants: baseline census, vaccination, census updates and follow ups at 6 months, 12 months, 18 months, 24 months post initial vaccination campaign, and census updates at 6 months, 12 months, 18 months and 24 months after unblinding.
- Additional contacts in a subset for blood sampling: vaccination day 0, day 28, day 545, day 730, post initial vaccination and day 365 and day 730 post unblinding.
- Additional contact in a subset of participants for safety monitoring: day 7 for all adverse events

A schedule of planned trial activities can be seen in Appendix B and C.

Participant identification

Trial participants

Children aged 9 months to <16 years (i.e., up to 15 years 364 days) who are in good health at the time of enrolment and are residents of the study area will be eligible to participate in this study. Participants will be identified as living within the defined catchment area of Mirpur, Dhaka. Trial staff, including local community health volunteers, will identify households with children <16 years of age and approach them for participation.

Inclusion Criteria

The participant must satisfy all the following criteria to be eligible for enrolment:

- Parent/guardian is willing and competent to provide informed consent. If the participant is 11 to <16 years of age, informed assent will also be sought,
- Aged between 9 months (or eligible for measles vaccination according to local protocol) and <16 years (i.e. up to 15 years 364 days) at time of vaccination,
- Apparently healthy (no complaints of febrile illness) on the day of vaccination,
- Parent/guardian confirms that their child will be willing and be able to comply with study requirements including follow-up contact, according the schedule (Appendix B),
- Living within the study catchment area at the time of vaccination.

Exclusion Criteria

The participant will not be enrolled if any of the following criteria apply:

- Has knowingly received a typhoid or Japanese encephalitis vaccine in the last three years,
- Known allergy to any of the vaccine components,
- Medical or social reasons that will prevent the participant from conforming to the study requirements as judged by a medical professional,
- Planning to move away from the catchment area within the next month
- Pregnant at the time of vaccination, as confirmed by a urine test (urine pregnancy test will be done in girls who are married)

Temporary exclusion criteria

Participants will be temporarily excluded from being vaccinated if, at point of vaccination, any of the following apply:

- Receipt of any other vaccines in the last 30 days
- Current temperature of at least 38oC or reported fever within 24 hours prior to vaccination,
- Use of antipyretics within 4 hours prior to vaccination.
- Unmarried girls between the ages of ≥12 and <16 years old whose first day of their last menstrual period (LMP) is more than 28 days ago or who do not know the date they last menstruated upon presentation

If these apply, the participant will be temporarily excluded for vaccination until the temporary exclusion criteria no longer applies. Please note for children with fevers above 38 oC this must be a minimum of 48 hours after the fever has resolved. Unmarried girls between the ages of ≥12 and <16 years whose first day of their LMP is more than 28 days or who do not know the date they last menstruated upon presentation will be asked to return after starting their next menstruation, for vaccination. A re-assessment will be conducted to ensure these temporary exclusion criteria no longer exist.

Withdrawal of Participants

Participants' parents/guardians can withdraw consent at any point. The Investigator may also discontinue a participant from the trial at any time if the Investigator considers it necessary for any reason including:

- Ineligibility (either arising during the trial or retrospectively having been overlooked at screening),
- Significant protocol deviation,
- Significant non-compliance with treatment regimen or trial requirements,
- An adverse event or disease progression resulting in the inability to continue to comply with trial procedures and follow-up,

Depending on which aspects participants wish to withdraw from, withdrawal will result in cessation of any follow-up calls, visits, or blood tests (as applicable to the subset). No further treatment is required in the study, so no additional action will need to take place. Participants' parents/guardians will have the choice when withdrawing, to withdraw from active study procedures only (follow-up calls and visits) but remain in the passive surveillance for the primary outcome, (allowing us to access their hospital records and blood test results), or withdraw from all study contact. In the case of a participant withdrawing from all study contact, we will not collect any data of hospital presentation or blood culture results. Data and blood samples collected prior to the time of participant withdrawal will be kept and analysed as part of the study data. A participant who withdraws from the study has the option to re-engage at a future date if they choose to do so. All participants who withdraw from the study will be given information on how to re-engage with the study if they so choose. Reasons for withdrawal from the study, if known, will be recorded in the participants CRF.

Trial procedures

Baseline Mapping

The baseline map of the geographically defined catchment area will be created based on satellite images and existing GIS Data from earlier projects. The satellite image of the study areas will be extracted from Google Earth Pro 7.1.5. After completion of the extraction process the satellite image will be georeferenced and printed along with existing GIS Data for field mapping and the census. In this process the census field team will visit each structure, road and other salient geographic feature in the images along with existing GIS Data. They will update geographic features on the satellite image and collect census data. The census team will also identify all the public and private health facilities, as well as educational and religious institutions and collect basic information about these facilities. After completion, the census population will be merged with GIS data for cluster formation.

Baseline Census

A baseline census will enumerate all households within the geographically defined catchment area. Demographic information will be collected on all household members, including but not limited to: total size of household, age and sex of all household members, family relationship, etc. Consent for the household will be given by either the head of the household or a key informant.

Cluster formation of the study area

The study population for the cluster-randomized control trial will be recruited from multiple wards in a geographically defined area of Mirpur. The study area will be divided into 150 clusters. Each cluster will consist of a total population of around approximately 1250 population. Clusters will be demarcated by road network and will include landmarks and physical features such as wide drain, canal and entrance of the structure. Others areas such as commercial places, education zone and playground will also be

considered for cluster demarcation. The cluster margins will be adjusted so that they are aligned with natural divisions to separate residences in the community. An imaginary inner cluster line will be drawn to exclude a population of approximately 200-300 people within each cluster which will also support in separating two adjacent clusters. This kind of demarcation will be helpful to minimize the contamination between clusters. The entire cluster population (not just the inner cluster) will be eligible for vaccination. The inner cluster will include approximately 950–1050 population size and will serve as the population under primary analysis, though the full cluster will be analyzed in secondary analyses. GIS mapping tools will be used for identification of all geographical characteristics and demarcation of clusters.

Pilot Study for Safety

A pilot study to evaluate vaccine safety, prior to the main study, will individually randomize 200 children to receive either Vi-TCV or the JE vaccine. These children will be selected from one of the areas in Mirpur that is not selected for the cluster-randomised trial. Eligibility will be assessed according to the same inclusion and exclusion criteria used in for the main trial. In this pilot phase, two sequential groups of children will be studied: first, children 3-<16 year olds (n=100), followed by children 9 months to <3 years old (n=100). The pilot will begin by vaccinating the children 3 to <16 years of age, and will proceed to the younger children after safety of the vaccine is confirmed one week post vaccination. The safety data from the older age group will be reviewed by the DSMB prior to the younger age group being vaccinated. After the pilot study is completed in both age groups, with safety documented one week after dosing for all ages involved, the cluster-randomised trial can begin. Safety data will be presented to the DSMB prior to initiating the main cluster randomized trial. After approval by the DSMB, the Vi-TCV main study will be initiated.

Pilot study participants will also be invited to attend enteric fever passive surveillance clinics, until the end of the study. In this surveillance all participants who present with a subjective history of ≥2 days fever and/or a temperature of ≥38°C at presentation will have a blood sample taken for culture and other tests (6-10 ml, depending on age), and will receive appropriate clinical management. Those with positive blood cultures will be visited at home to confirm their identity given at the treatment centre, to collect information about their illness, and to review the treatment given, and adjust as necessary, based on laboratory testing. This surveillance data collected on pilot study participants will be analysed separately to the main trial passive surveillance data.

For the duration of the two years, until unblinding, pilot study participants will be encouraged to report any serious health events or hospitalisations. The reporting of such information will be done as per the safety reporting section below.

Recruitment for vaccination

Potential participants living in the 150 clusters of Mirpur, Dhaka will be identified. Trial staff, already embedded within the area, including community health volunteers, will systematically approach each household in the area to identify households with children aged 9 months to <16 years (i.e. up to 15 years 364 days). Identified children will be screened as per the process below. Information will be recorded regarding the history of blood culture positive typhoid fever during vaccination.

Screening and eligibility assessment

Once a household containing a potential participant is identified, the parent/guardian of the child will be given basic information about the trial in an invitation letter and invited to attend the nearest study vaccination clinic if they are interested. After arrival at the study clinic, more detailed information will be given and screening according to inclusion/exclusion criteria will occur. Eligible participants will then be

approached for acquisition of informed consent/assent. After consent is taken, a temperature will be taken and parents will be asked about anti-pyretic use and timings of previous vaccination to assess temporary exclusion criteria. A brief medical history will be taken and eligibility will be assessed against inclusion and exclusion criteria. Participants with a temporary exclusion criterion identified will be informed of the reason and asked to return 48 hours after the fever has resolved or 30 days after the last vaccination was given for repeat assessment and re-consent. Unmarried girls between the ages of ≥12 and <16 years whose first day of their LMP is more than 28 days or who do not know the date they last menstruated upon presentation will be asked to return after starting their next menstruation, for vaccination.

Informed consent

The parent/guardian must personally sign and date the latest approved version of the Informed Consent form before any trial specific procedures are performed. Assent will also be sought from children 11 years of age or older, to participate in the trial. Information sheets will be given to children aged 11 -16 years in language that they can understand.

Written and verbal versions of the Participant Information and Informed Consent/Assent will be presented to the participants' parent/guardian in the local language detailing no less than: the exact nature of the trial; what it will involve for the participant; the implications and constraints of the protocol; the known side effects and any risks involved in taking part. It will be clearly stated that the participants' parent/guardian is free to withdraw their child from the trial at any time for any reason without prejudice to future care, without affecting their legal rights and with no obligation to give the reason for withdrawal. Participants will also be informed that they can choose to have their remaining blood samples destroyed and not maintained for future analysis at the end of the trial.

The participant and their parents/guardians will be allowed as much time as they wish to consider the information, within the recruitment period, and the opportunity to question the Investigator or other independent parties to decide whether they will participate in the trial. Written Informed Consent/Assent will then be obtained, with additional opt-in consents to be randomized for blood sampling, by means of participants' parent/guardians dated signature or thumbprint and dated signature of the person who presented and obtained the Informed Consent. The person obtaining consent will record informed assent on the same consent form, for children aged 11 and older. The person who obtained the consent must be suitably qualified and experienced, and have been authorised to do so by the Principal Investigator (PI). A copy of the signed Informed Consent will be given to the participant. The original signed form will be retained at the trial site.

A third party will act as a witness for the parent/guardian to attest that the information in the consent form and any other written information was accurately explained to, and apparently understood by, the parent/guardian and that informed consent was freely given by the parent/guardian. The witness will also sign and date the consent form.

Randomisation and blinding

Randomisation

Computer generated randomisation lists will be prepared by the study statistician. Randomisation lists will allocate each cluster to receive either Vi-TCV or the control vaccine, in equal numbers (75 clusters in each arm), based on population size, using stratified block randomisation. Stratification will be by the geographic wards, the distance to the closest passive surveillance clinics (above the median versus at or below the median), and the number of children 9 months to <16 years age (above the median versus at or below the median). Once participants are enrolled in the study, they will be vaccinated with either Vi-TCV or the control vaccine, according to the residential cluster in which they live.

The result of participant consent, including consent to be selected into the immunogenicity study, will be directly entered onto the eCRF using a handheld device (TAB).

Randomisation codes for the clusters will be kept in a separate database.

Blinding

The trial will aim for participant- and observer-blinding. The vaccines will not be repackaged and relabelled for the purposes of this trial; however all vaccine preparation will be done behind a curtain so that participants, parents/guardians or blinded staff do not observe which vaccine is being administered. . Efforts will be made to conceal the identity of the administered vaccine from both participants and staff involved in follow-up procedures, with only the vaccinating trial staff aware of which vaccination has been given. Subjects will not be told the identity of the administered vaccine. To help maintain observer blinding, the unblinded vaccination team will not take part in the procedures of blood taking, contact at 7 days after dosing for assessment of adverse events, contacts in the 6 monthly censuses and surveys, or follow-up for enteric fever at the surveillance sites. To help ensure blinding of the investigators, all vaccinations will be carried out by trial staff that are not named investigators in the study protocol. Separate vaccination teams will be responsible for the study which will include nurses, trained field assistants and volunteers recruited for this purpose.

Circumstances may arise where unblinding is needed prior to the end of the study e.g. occurrence of a SUSAR or requirement of a medical intervention that would be influenced by knowledge of which vaccine the individual has received. In such circumstances, the PI or delegated study physician will contact the Independent Safety Monitor (ISM) to request an independent assessment of the child. The ISM will be a physician in Bangladesh who is not an investigator for this study. They will be responsible for conducting an independent assessment of the child and any information available relating to the event.

The details of the assessment will be reported to the PI, Chief Investigator, Data Safety Monitoring Boards (DSMBs) and discussed. If deemed necessary, the DSMBs will recommend unblinding to the IRB and contact the study statistician responsible for providing information on the vaccine received by the individual in question

Any event of unblinding will be fully documented in the CRF.

Vaccination Visit

Potential participants will attend the vaccination sites situated within study area. Upon arrival, inclusion/exclusion will be assessed, and written informed consent/assent will be formally obtained with the parent/ guardian of the participant. A basic medical history including typhoid will be taken. Temporary exclusion will then be checked. If these apply, the participant will be temporarily excluded for vaccination until the temporary exclusion criteria no longer applies. Please note for children with fever $\geq 38^{o}$C this must be a minimum of 48 hours after the fever has resolved.

After acquiring consent, a member of the study team will collect/edit demographic information (including age and address) and participant contact details. The participant will then be vaccinated, based on the study cluster in which they live, and if they consent, enrolled in the immunogenicity study. If the participant is included in the immunogenicity study, a blood sample will be taken by a suitably trained staff member.

All details will be recorded in the CRF.

Based on the cluster randomization, the appropriate vaccine will be administered by a trained member of the study team. The site of vaccination (right or left arm or thigh) will be recorded. The participant will be considered enrolled into the study at the point that any medical procedure takes place (i.e. blood sample taken or vaccination administered).

A study card containing the name of the study, the participant ID, and contact details for the study team will be given to parents/guardians to call if they have any concerns, or if their child is admitted to hospital at any time during the duration of the study. The card will also contain instructions to attend the study

health care facilities in the wards if the participant develops fever (≥2 days and/or temperature ≥38ºC at presentation) at any time over the next two years.
Parents/guardians of participants enrolled in the immunogenicity study will be given details of the follow-up appointments.

Subsequent Follow-up and Visits (Appendix B and C for visit timelines)
Adverse Events Follow-up (Day 7, -1/+7)
Follow-up active surveillance at 7 days post-vaccination will collect parent/gaurdian-reported information on adverse events following immunisation (AEFIs) for a subset of approximate 4800 children, with approximately a 1:1 ratio of children in each arm, selected to represent approximately equal numbers from all clusters. Assuming the AEFI incidence rate is 1% in both arms, with 2400 children in each arm, the 95% CI of the incidence rate will yield 0.4% on each side. Parents/guardians will be encouraged to use diary cards to record adverse events post-vaccination, to help recall symptoms and duration. Adverse events follow-up will occur in person at home visits or be conducted via telephone.
Information collected will include:

- Verbal reconfirmation of consent for participation in the trial,
- Report from parent/guardian of adverse events related to vaccination, including: pain, swelling, redness, hardness, fever, etc., (the protocol for action in the event of these occurring in section “safety reporting”) and use of medications following vaccination.
- Reiteration of contact details and instructions to attend the health care facilities in the case of fever (≥2 days and/or temperature ≥38ºC at presentation).

Parents of participants not in this subset will be encouraged to go to the ‘Adverse Event Monitoring Cell’at the Mirpur Field Clinic which will have a medical doctor on-call 24 hours a day for adverse event monitoring throughout the whole vaccination period + 7 days.

Census updates and standard follow-up
A census update will occur every 6 months over the entire follow-up period of the trial. Children aged 9 months to <16 years identified during any census update in the initial 2-year, who have not previously been enrolled in the trial, in-migrated and reached at eligible age group (9 months or older) will be offered the opportunity to participate (including those who initially declined, but have since become interested in participating). If a child is interested, they will be screened and consented, as per the plan described above. The newly enrolled participants will be vaccinated according to their area of residence, in line with the cluster randomisation designated at the beginning of the trial.

As part of the census updates, information about household demographics will be collected, including births and deaths, as well as information about serious illnesses and hospitalisations, and population movement.

For parents/guardians of vaccinated participants there will be routine participant follow-up until unblinding around 3 years after initial vaccination campaign, consisting of a basic questionnaire, for all participants enrolled in the trial (Appendix B). A brief interview will be conducted by trial staff at each follow-up contact.

These interviews will collect parent/gaurdian-reported participant information, including:

- Ensuring participant and family still lives in area,
- A record of mortality and morbidity end points, including:
    - Mortality,

    - Severe illness occurance and duration,
    - Visits to clinics, hospitals, or pharmacies (and records requested if they attended a facility other than the study sites),
- A record of fever occurrence, episodes of clinically diagnosed and culture confirmed typhoid and school/work absenteeism
- A reminder to attend designated health care facilities if they develop fever of ≥2 days.

The results from this follow-up contact will be included in the participant's CRF. Trial staff within the ward clinics will review participant reports of febrile illnesses, documented during follow-up contact to make a clinical judgement of suspected typhoid, especially for those cases that did not result in medical treatment.

Unscheduled follow up contacts will occur when the bi-weekly CHW visits identify deaths or hospitalisations occurring for any cluster resident. If a death or hospitalisation has been identified, verbal consent will be obtained by the CHW to record the residents contact details and arrange a follow up visit. Trained members of staff will conduct the verbal autopsy visit and record the details of the hospitalisation. The data from these contacts will be captured in participant CRFs. We will not collect the details of the hospitalisation after unblinding the trial.

Immunogenicity Study

The subset of participants enrolled in the immunogenicity sub-study will receive three additional face-to-face follow-up visits at their nearest trial clinic 28 days (D28+/-4 days), at 18 months (D545+/-56 days), at two years (D730+/-90 days) post initial vaccination, and at one year (D365 +/-90 days), and at two years (D730+/-90 days) post unblinding. At these additional visits the following procedures will be performed:

- Confirmation of continued participation in the study,
- Draw of 3-5ml blood (depending on age) for transport to the icddr,b Laboratories for serum separation,
- Confirmation of contact details and reiteration of instructions to attend the health care facilities in the case of a prolonged fever ≥2 days.

The above information will be recorded in the participant's CRF.

Cluster Residents Presentation with Fever

Cluster residents, including vaccinated participants, who experience a fever lasting for ≥2 days and/or temperature ≥38°C at presentation will be consented to have their health data recorded as part of the study (as described above in the informed consent section). Individuals will then be assessed by medically trained staff, as per routine health care procedures. Details of this assessment will be recorded and the participant will receive routine standard of care management and treatment, as deemed appropriate by the assessing member of staff. Trial staff will collect information about the participant illness episode and record it in the CRF. This will include information such as temperature and duration, hospital admission, surgeries, and antibiotics prescribed. The only additional study related procedure performed at this visit will be collection of an additional ~6-10 ml blood sample (dependant on age) for culture. Laboratory blood-culture confirmed typhoid and paratyphoid cases will be followed-up within two weeks after presentation with fever to record outcome of illness, school/work absenteeism as a result of illness, and to provide appropriate additional clinical management. For cases with isolates that are not susceptible to the antibiotic regimen initiated, visits will be made immediately on receipt of antibiotic sensitivity results, and appropriate clinical management will be instituted. If illness is not resolved at the two-week follow-up

assessment, an additional follow-up will occur two weeks later (4 weeks after initial presentation with fever) to record outcome of illness and resolution.
Participants that present to passive surveillance health care facilities with symptoms of Japanese Encephalitis infection will be tested for antibodies using an ELISA kit.

Pregnancy follow-up

Dependent on written consent and assent being given, follow-up of pregnancy outcomes will be undertaken on all girls who are identified during routine follow-up as having been pregnant at the time of vaccination or who became pregnant within 3 months of being vaccinated. This follow-up will include monthly visits until the end of the pregnancy, with the final contact occurring after delivery or outcome. From 8 months of gestation there will be additional weekly follow-up phone calls. If any outcome occurs before 6 monthly follow up visit then full questionnaire will be completed.

We will not follow up pregnancy for participants vaccinated at unblinding visit as there will no live attenuated JE vaccine given.

Sample Handling

Blood cultures to confirm typhoid infections:

Blood culture will be used for diagnosis and confirmation of suspected typhoid fever. Blood culture will be carried out using the BacTec/BacT/Alert systems. Blood cultures will be handled, stored, and processed, in accordance with GCP guidelines and standard operating procedures of the facility laboratory. Organisms will be identified by the biochemical tests and final identification will be done by slide agglutination test with Salmonella specific antisera (Denka Sieken, Tokyo, Japan). The pattern of antibiotic susceptibility of S. Typhi and S. Paratyphi causing enteric fever will be assessed by the disc diffusion method on a Mueller-Hinton agar plate with Oxoid disks containing ampicillin, cotrimoxazole, chloramphenicol, azythromycin, ceftriaxone, ciprofloxacin, nalidixic acid, cefixime, and gentamicin. The results of these tests will be recorded in the participant CRF for use in this study.
Remaining samples will be stored at the Mucosal Immunology & Vaccinology laboratory for further analysis.

Immunogenicity study samples:

Blood samples taken for the immunogenicity study will be transported to icddr,b laboratory daily where they will be processed and stored by trained study staff, in accordance with standard operating procedures (SOP).
The plasma will be stored and used to measure the induced immune response in the vaccinees. The plasma will also be used for investigation of novel diagnostic markers, for example, indications of an acute serological response indicating recent typhoid exposure, or identification of a metabolomics signature compatible with infection.
The primary laboratory technique performed will be anti-Vi antibody ELISA performed on the extracted plasma sample, using a commercially available assay assay (VaccZyme, The Binding Site). This assay will be performed according to the manufacturer's instructions.
Laboratory processes will be conducted at the icddr,b laboratory. If necessary the samples will be shipped to other laboratories under MOU/MTAs. At the end of the trial, all remaining samples will be kept for 5 years.

Genetics analysis:

To investigate the host genetics, blood samples that will be collected from the culture positive typhoid patients and the participants of immunogenicity component will be used, with consent. Blood specimens

will be processed to extract the genomic DNA and GWAS approach will be applied, to investigate the role of host genetic controls induced by vaccines and susceptibility to infectious diseases like typhoid.

Discontinuation/Withdrawal of Participants from Trial Treatment

Trial treatment consists of a single vaccination received at the point of enrolment into the study. It is not possible to withdraw from trial treatment after vaccination.

Unblinding

Unblinding will occur following the completion of the 24 month follow-up contact. At this point all participants in the control group will be offered vaccination with Vi-TCV. The procedure of unblinding and final visit can be found in Appendix H.

Definition of End of Trial

The end of trial is the date that the last sample is processed for the purposes of this study.

Investigational Medicinal Product (IMP)

IMP description

Trial vaccine

Vi polysaccharide-tetanus toxoid conjugate vaccine (Vi-TCV). Trade name: Typbar-TCV®, Bharat-Biotech.

Each 0.5ml vaccine dose contains:

- Purified Vi-Capsular Polysaccharide of *S.* Typhi Ty2 conjugated to Tetanus Toxoid 25µg
- Sodium chloride 4.5 mg
- Water for Injection q.s. to 0.5ml

The vaccine is packaged as a pre-filled 2.5ml 5-dose vial. It will be administered as an intramuscular injection in the antero-lateral thigh for younger children, or the upper arm for older children, according to local protocols.

Control vaccine

Japanese Encephalitis vaccine will be the control vaccine. The licensed trade name is SA14-14-2, Japanese Encephalitis Vaccine, Live; it is produced by Chinese Chengdu Institute of Biological Products. The vaccine is produced from a live attenuated strain grown in primary hamster kidney cells. The vaccine is supplied in multi-dose vials containing 5 doses each. The major components of the final vaccine included live attenuated JE viral strain SA 14-14-2 with human serum albumin, gelatin, sucrose, lactose, and carbamide. The lyophilised active component is reconstituted with excipient diluent (buffered saline solution) before use. A single dose of the vaccine is 0.5 ml and given subcutaneously. Storage temperature of vaccine is at 2-8°C.

Supply

The Vi-TCV vaccine (Typbar-TCV®) will be provided by Bharat-Biotech. The JE control vaccine (SA14-14-2 JE) will be obtained from the Chengdu Instititute of Biological Products Co. Ltd. The study vaccines will be shipped to the icddr,b site in Bangladesh and stored at the EPI/ icddr,b cold room facilities.

Storage

The Vi-TCV study vaccine and the JE control vaccine will be stored at 2° to 8° C (35 ^{o} to 46 ^{o} F) in a temperature monitored EPI/icddr,b cold room facilities, when not in use for the daily activities. The vaccines will be stored in temperature monitored refrigerators or cool boxes, when in use for the day's activities. Each vial will be labelled with a "vaccine vial monitor"; a temperature-sensitive dot that provides an indication of the cumulative heat to which the vial has been exposed. It warns the end user when exposure to heat is likely to have degraded the vaccine beyond an acceptable level and should not be used.

Vi-TCV (Typbar-TCV):

The vaccine should not be frozen, and if it has been, it will be discarded. Opened vials will be used within 6 hours when stored under refrigeration at 2 to 8^oC. Refrigerator and cold chain temperatures will be audited during the vaccination campaign to ensure they are within range. The intervention vaccine is presented in 5 dose vials of active vaccine, ready for administration.

SA14-14-2 JE vaccine

The diluent (2.5 ml/vial) will be stored at the icddr,b facilities, according to manufacturer specifications at 2-30° C. The reconstituted vaccine will be stored at 2 - 8^oC and will be protected from direct sunlight. The vaccine can be used for up to 6 hours after reconstitution.

Accountability of the trial treatment

The vaccines will be shipped to a central storage facility in Bangladesh, and passed through customs. They will then be transported and distributed to local clinics whilst maintaining the cold-chain (aiming for temperature between 2-8^oC).

The number of doses of study vaccines that are received, used and wasted will be documented daily during the trial and checked weekly.

Unused vaccines at the end of the trial may be retained for laboratory use only (such as laboratory assay development). Any recall of study vaccines required for use in the study or reporting of defective vaccines will be performed according to trial SOPs.

Safety reporting:

Definitions

Below are the various categories of Adverse Events Following Immunization (AEFIs).

Adverse Event (AE)	Any untoward medical occurrence in a participant to whom a medicinal product has been administered, including occurrences which are not necessarily caused by or related to that product.
Adverse Reaction (AR)	An untoward and unintended response in a participant to an investigational medicinal product which is related to any dose administered to that participant.<br><br>The phrase "response to an investigational medicinal product" means that a causal relationship between a trial medication and an AE is at least a reasonable possibility, i.e. the relationship cannot be ruled out.<br><br>All cases judged by either the reporting medically qualified professional or the Sponsor as having a reasonable suspected causal relationship to the trial medication qualify as adverse reactions.

Serious Adverse Event (SAE)	A serious adverse event is any untoward medical occurrence that:<br>• results in death<br>• is life-threatening<br>• requires inpatient hospitalisation or prolongation of existing hospitalisation<br>• results in persistent or significant disability/incapacity<br>• consists of a congenital anomaly or birth defect.<br>Other 'important medical events' may also be considered serious if they jeopardise the participant or require an intervention to prevent one of the above consequences.<br>NOTE: The term "life-threatening" in the definition of "serious" refers to an event in which the participant was at risk of death at the time of the event; it does not refer to an event which hypothetically might have caused death if it were more severe.
Serious Adverse Reaction (SAR)	An adverse event that is both serious and, in the opinion of the reporting Investigator, believed with reasonable probability to be due to one of the trial treatments, based on the information provided.
Suspected Unexpected Serious Adverse Reaction (SUSAR)	A serious adverse reaction, the nature and severity of which is not consistent with the information about the medicinal product in question set out:<br>• in the case of a product with a marketing authorisation, in the summary of product characteristics (SmPC) for that product<br>• in the case of any other investigational medicinal product, in the investigator's brochure (IB) relating to the trial in question.

NB: to avoid confusion or misunderstanding of the difference between the terms "serious" and "severe", the following note of clarification is provided: "Severe" is often used to describe intensity of a specific event, which may be of relatively minor medical significance. "Seriousness" is the regulatory definition supplied above.
Brighton Collaboration case definitions for anticipated outcomes will be used to standardize the identification and reporting of all AEFIs.
Any pregnancy occurring during the clinical trial and the outcome of the pregnancy should be recorded and followed up for congenital abnormality or birth defect, at which point it would fall within the definition of "serious".
A flow chart for AEFI recording and reporting can be seen in Appendix E. Descriptions of these procedures are listed below (sections 10.3 and 10.4)

Causality

The relationship of each adverse event to the trial medication must be determined by a medically qualified individual according to the following definitions:

Related: The adverse event follows a reasonable temporal sequence from trial medication administration. It cannot reasonably be attributed to any other cause.

Not Related: The adverse event is probably produced by the participant's clinical state or by other modes of therapy administered to the participant.

<u>Procedures for Recording Adverse Events</u>

From vaccination through day 7

All adverse events related to vaccination, as judged by a medically qualified investigator, occurring during the first 7 days post vaccination that are observed by the study team/investigator or reported by the participants' parent/guardian, will be recorded on the CRF. The information will be collected both passively and actively. For a subset of approximate 4800 participant's information will be actively sought via phone calls and home visits, parents of participants not in this subset will be encouraged to go to the 'Adverse Event Monitoring Cell' at the Mirpur Field Clinic which will have a medical doctor on-call 24 hours a day for adverse event monitoring throughout the whole vaccination period + 7 days.

The following information will be recorded: description, date of onset and end date, severity, assessment of relatedness to trial medication, and action taken. Follow-up information should be provided as necessary.

The severity of events will be assessed on the following scale: 1 = mild, 2 = moderate, 3 = severe.

All Serious Adverse Events (SAEs) observed by the Investigator, members of the study team or reported by the parent/guardian will be recorded on the CRF. We will keep collecting SAEs after unblinding vaccination with TCV for another 2 months.

The following information will be recorded: description, date of onset and end date, severity, assessment of relatedness to trial medication, other suspect drug or device and action taken. Follow-up information should be provided as necessary.

Throughout the study period

Serious Adverse Events (SAEs), including Serious Adverse Reactions (SARs), as judged by a medically qualified investigator, observed by the Investigator, members of the study team or reported by the parent/guardian, will be recorded on the CRF till 2 months after unblinding.

The following information will be recorded: description, date of onset and end date, severity, assessment of relatedness to trial medication, and action taken. Follow-up information should be provided as necessary.

All mortality occurring during the duration of the trial will be recorded in the CRF, and investigated by medically qualified trial staff.

All SAEs, including SARs recorded in the CRFs, for the duration of the study, from first vaccination until trial completion, will be followed by a medically qualified investigator either until resolution, or until the event is considered stable.

Reporting Procedures for Adverse Events

For this study, forms will be used for reporting all SAEs and SARs occurring during this study.

All SAEs, including SARs occurring within the first 30 days, post vaccine administration, and then only

SARs occurring until the end of the trial, will be reported to the local and international Data Safety Monitoring Board (DSMB), the Chief Investigator in Oxford, PI in Bangladesh and the other study Investigators within 24 hours of the Site Study Team becoming aware of the event. A more detailed report form will be completed during medical follow-up, and sent within the shortest period possible of the initial report, to all parties mentioned above. Additional and further requested information (follow-up or corrections to the original case) will be detailed in subsequent safety report forms. All SAEs, including SARs must be reported to the trial sponsor (University of Oxford) within 7 days.

Summary reports will be submitted to the icddr,b IRB, DGDA and the Bangladeshi AEFI committee annually. The trial will also maintain records of reports and receipt of report. SAEs/SARs will also be reported to the Oxford REC in the Annual Progress Report.

Expectedness

Expectedness will be determined according to the Summary of Product Characteristics.

SUSAR Reporting

All Suspected Unexpected Serious Adverse Reaction (SUSARs) will be reported by icddr,b PI and Oxford CI to the relevant Competent Authority and bodies mentioned above for the SAEs, as applicable. For fatal and life-threatening SUSARS, this will be done no later than 7 calendar days after the Sponsor or delegate is first aware of the reaction. Any additional relevant information will be reported within 8 calendar days of the initial report. All other SUSARs will be reported within 15 calendar days.

Principal Investigators will be informed of all SUSARs for the relevant IMP for all studies with the same Sponsor, whether the event occurred in the current trial.

Safety Monitoring Committee

An international data safety monitoring board (iDSMB) will be established to assess at intervals the progress of the trial and the safety of the study agent. The 7-member team will be established to oversee all three TyVAC vaccine efficacy trial sites: Bangladesh, Malawi, and Nepal, to ensure that safety data relevant to all three trials is assessed and disseminated across the three sites. The DSMB will consist of three representatives from Bangladesh, including a member of the local Ethical Review Committee (ERC), and a staff members of icddr,b. In addition, representatives from Malawi and Nepal, as well as an independent chair and statistician will complete the DSMB membership. The members will not be involved in any of the studies in any way.

A local DSMB will also be created to oversee the Bangladesh trial specifically. Three DSMB members from Bangladesh will contribute on both the local and international DSMB committees. The local DSMB will be responsible for the oversight of the pre-trial safety study. The local DSMB will also provide safety monitoring during the main trial, in conjunction with the international DSMB.

In addition, a physician in Bangladesh with relevant study-related or therapeutic expertise will be identified as an Independent Safety Monitor (ISM). The ISM will not be an investigator for this study. As this is a single dose vaccine intervention, we anticipate that any circumstances warranting unblinding will be rare. However, circumstances may arise where unblinding is necessary prior to the end of the study e.g. occurrence of a Suspected Unexpected Serious Adverse Reactions (SUSAR) or requirement of a medical intervention that would be influenced by knowledge of which vaccine the individual has received. In such circumstances, the identified ISM would be requested to carry out an independent assessment of the child.

The details of the assessment will be reported to the PI, CI, international and local DSMB and discussed. If deemed necessary, the local and international DSMB will recommend unblinding.

Development Safety Update Reports

Safety reports will be submitted in accordance with the Oxford and icddr,b regulations and guidelines.

Sample Size Calculation and Outcome (Primary and Secondary) Variable(s)

Clearly mention your assumptions. List the power and precision desired. Describe the optimal conditions to attain the sample size. Justify the sample size that is deemed sufficient to achieve the specific aims.

Formulae for computations for sample size:

In cluster RCTs, the individuals within a cluster are generally more similar to each other than to individuals from other clusters. The number of clusters (*c*) required is given by the formula,

$$c = 1 + (z_{\alpha/2} + z_{\beta})^2[(\lambda_0 + \lambda_1)/y + k^2(\lambda_0{}^2 + \lambda_1{}^2)]/(\lambda_0 - \lambda_1)^2$$

,

in which *k*= the between-cluster coefficient of variation (SD/mean) and *y* is the number of person-years of follow up per cluster.[1] Assuming 2 years of follow-up, the total sample size is $c \cdot y/_2$.

In addition to the parameters α, β, λ_0, λ_1, *k*, and *y*, required for the calculations described above, trial planning needs to account for the average vaccine coverage rate of the study population (*g*), and a further inflation of the sample size is included, such that *N*=*n*/*g* where *N* is the final sample size required, *n* is the sample size computed from the above formulae prior to accounting for coverage rate, and *g* is the average proportion of vaccinees among the study population throughout follow-up.

We assumed α=0.05, β=0.2, *k*=*0.5*, and g=0.60.

Sample size calculations are based on the following assumptions:

1. An overall incidence of typhoid fever of 50 cases per year, per 100,000 persons in the entire population, with higher incidence rates in children under 16 years.
2. Age specific incidence rates were determined from the age distribution of typhoid cases from current typhoid surveillance in Mirpur.
3. A direct protective effect of vaccination of 80% (protection of vaccinees) and an indirect effect of 20% (protection of neighboring non-vaccinees), as predicted from mathematical modelling.
4. A coefficient of variation of 0.5.
5. Cluster size (of the inner clusters) of 1,000, of whom an expected 289 will be aged 9 months to <16 years.
6. 60% average vaccine coverage of the target age group throughout follow-up.
7. 2 years of follow-up.

Based on the above assumptions, the sample size calculated for use in this trial will be 43,350 children, within 150 clusters, each on average containing 1,000 participants in the inner-cluster, randomized 1:1 to receive Vi-TCV or JE vaccine. The total number of children eligible for vaccination in both inner and outer clusters, is therefore 43,350 x 1.25 = 54,188, of which we expect approximately 60% to participate, resulting in approximately 32,500 children vaccinated.

With these assumptions, the trial will have 98% power to detect a 82% level of total vaccine protection by Vi-TCV and 74% power to detect a 43% level of overall protection by Vi-TCV, and p-value <0.05 (2 tailed).

Outcome Variables:

Primary:

a) The incidence of blood culture confirmed typhoid fever in vaccinees in intervention clusters compared to control clusters

b) The incidence of blood culture confirmed typhoid fever in all residents of the intervention clusters compared to control clusters

Secondary:

1. The proportion of participants developing adverse events within the first 7 days post-vaccination, and serious adverse events within 6 months of vaccination, as determined through sub-sample at 7 days, and self-reporting at follow-up contact
2. Rates of participants with a history of ≥2 days of persistent fever, and a temperature of ≥38 degrees C upon presentation, together with abdominal discomfort, in vaccinees in intervention clusters compared to control clusters
3. Rates of participants with a history of ≥2 days of persistent fever, and a temperature of ≥38 degrees C upon presentation, together with abdominal discomfort, among all residents of the Vi-TCV clusters compared to the control vaccine clusters
4. Rates of patients with clinical diagnoses of typhoid fever in vaccinees in intervention clusters compared to control clusters
5. Rates of patients with clinical diagnoses of typhoid fever among all residents of the Vi-TCV clusters compared to the control vaccine clusters
6. The incidence of blood culture confirmed paratyphoid fever in vaccinees in intervention clusters compared to control clusters

Exploratory:

1. Incidence of all cause hospitalization in community-based surveys of all residents of the Vi-TCV clusters compared to the control vaccine clusters
2. Assay of anti-Vi antibodies in blood samples collected at baseline (Day 0), at one month (Day 28), in a subset of participants from each treatment arm
3. Assay of anti-Vi IgG antibodies in blood samples collected at baseline (day 0), 18 months (day 545) and two years (day 730) in a subset of participants receiving intervention and control vaccinations
4. Incidence of absenteeism from school/work in vaccinated children and their guardians as a result of confirmed typhoid infection in intervention clusters compared to control clusters
5. Incidence of absenteeism from school/work in all cluster residents and their guardians/carers (if applicable) as a result of culture confirmed typhoid infection in intervention clusters compared to control clusters
6. Rates and circumstances of mortality, recorded from hospital records and verbal autopsies via six-monthly census updates, in vaccinees in intervention clusters compared to control clusters
7. Rates and circumstances of mortality, recorded from hospital records and verbal autopsies via six-monthly census updates, in all residents of the Vi-TCV clusters compared to the control vaccine clusters
8. Rates of acute abdominal surgery, and of surgery for intestinal perforation, in vaccinees in intervention clusters compared to control clusters
9. Rates of acute abdominal surgery, and of surgery for intestinal perforation, in all residents of the Vi-TCV clusters compared to the control vaccine clusters

Data Analysis

Describe plans for data analysis, including stratification by sex, gender and diversity. Indicate whether data will be analysed by the investigators themselves or by other professionals. Specify what statistical software packages will be used and if the study is blinded, when the code will be opened. For clinical trials, indicate if interim data analysis will be required to determine further course of the study.

Analysis Plan due to COVID-19

The COVID-19 global pandemic has caused significant disruptions to both routine and study-based health care systems in Bangladesh. As a result, study activities have been paused for safety reasons. To account for this unexpected change, the primary analysis will be conducted using data censored at the time point of the 18-month census. At a time when it is safe to do so, the final study activities, including the unblinding and vaccination of controls, and a final census, will be conducted, and a follow-up analysis will be conducted to incorporate all study data collected after the 18-month census time point.

Statistics

Description of Statistical Methods

The primary endpoint will be blood culture positive typhoid fever detected in passive surveillance at the study surveillance sites, undertaken for persons residing in residences of the "inner clusters" demarcated geographically at baseline.

We will test the hypothesis that there is no difference in the incidence of blood culture confirmed typhoid fever between Vi-TCV and the control vaccine group using the mixed effects Poisson model adjusting for design effect and pre-specified prognostic factors. The design effect of the cluster randomisation will be adjusted as random effects. Kaplan-Meier survival curves will also be presented.

Vaccine efficacy (VE) will be calculated as (1 – IRR) x 100%, where IRR is the incidence rate ratio (Vi-TCV: control) from the mixed effects Poisson model. We will compare the mean and variance of the primary outcome, and if the data is over-dispersed, i.e. variance >> mean, we will change the primary analysis of primary outcome from mixed effects Poisson model to zero-inflated Poisson or zero-inflated negative binomial model depending on the variability and distribution of the data. The details of model selection under different scenarios will be specified in the statistical analysis plan (SAP).

Participants will be censored in the analysis at the time of last known residence in the surveillance area, death, or at the 18-month census visit. Statistical significance will be determined as a p value of less than 0.05 (2-tailed). Primary analyses will address total vaccine protection (comparing the incidence of typhoid in vaccinees in the two arms) and overall protection (comparing the incidence of typhoid in the two arms regardless of whether the vaccine assigned to the cluster was received). In secondary analyses, we will estimate indirect vaccine protection (comparing the incidence of typhoid in non-vaccinees in the two arms).

To evaluate heterogeneity of Vi-TCV vaccine protection among different subgroups, we will evaluate interaction terms between the vaccination and subgroup variables in these models. P values for these analyses will be calculated as two-tailed. The study is not powered to detect differences between sub-groups and any observed patterns will be interpreted cautiously, owing to the large study population and increased chance of Type I error. The details of the subgroup analysis will be specified in the SAP.
A fully detailed SAP will be prepared and signed off by the Chief Investigator prior to conducting any data analyses.

Procedure for Accounting for Missing, Unused, and Spurious Data:
All available data will be included in the analysis

Inclusion in Analysis
All participants in the entire clusters will be included in the primary analysis and all the participants in the inner clusters will be included in the secondary analysis.

Procedures for Reporting any Deviation (s) from the Original Statistical Plan

Any deviations from the statistical analysis plan will be described in the final study report.

Interim safety and immunogenicity analysis

An unblinded interim analysis of safety and immunogenicity data will be undertaken following completion of the 6 month follow up contact and the Day 28 immunogenicity blood draw. Unblinded aggregated safety and immunogenicity results from the interim analysis will be communicated to policy-makers, funders, and the wider scientific community through presentations and publications. This analysis will provide detailed safety and immunogenicity date associated with Vi-TCV to global decision makers, which may be important as countries consider the introduction of the vaccine into routine schedules. Unblinded analysis will be completed by designated senior statisticians, who are not involved in trial delivery, participant follow-up, and data collection. Procedures will be put in place to ensure trial participants and blinded implementation staff remain blinded (Appendix G). Individual level safety and immunogenicity data and all vaccine efficacy data will remain blinded until the time that official unblinding occurs.

Data management
Source Data
Source documents are where data are first recorded, and from which participants' CRF data are obtained. These will include clinical, laboratory, microbiological and immunological data. These include, but are not limited to, hospital records (from which medical history and previous and concurrent medication may be summarised into the CRF), clinical and office charts, laboratory and pharmacy records, diaries, microfiches, radiographs, and correspondence.
CRF entries will be considered source data if the CRF is the site of the original recording (e.g. there is no other written or electronic record of data). All documents will be stored safely in confidential conditions. On all trial-specific documents, other than the signed consent, the participant will be referred to by the trial participant number/code, not by name.
Access to Data
Direct access will be granted to authorised representatives from the Study team at the icddr,b, Sponsor, and the regulatory authorities to permit trial-related monitoring, audits and inspections.

Data Recording and Record Keeping
Electronic devices and hard copies of CRFs will be used to collect and record all data in trial CRFs. CRF and randomization data will be collected off-line and uploaded to a secure server on a regular basis, when electronic devices are brought back to the central field office, and reliable internet is available. Some CRFs will also be completed online.
Census, 6 monthly follow-up, pregnancy follow-up and hospitalisation data will be collected on an electronic device with an internally designed Android application; this application will be validated by data management and IT staff within Oxford University before use. All other CRFs will be designed and

maintained on REDCap, a secure web application for building and managing online surveys and databases. REDCap will be validated by data management and IT staff within Oxford University. The CRFs will be designed and maintained by a dedicated trial data manager, and quality control checks will be performed on a regular basis.

All participants will be identified by a unique trial specific number and/or codes; this will not include any identifiable information. CRFs will capture participant medical information from the trial clinic records, including but not limited to type of illness, severity, duration of illness, and treatment prescribed. Blood culture confirmed typhoid infections will be recorded in the CRFs. The results of six-monthly follow-up contact will be captured in electronic CRFs, including but not limited to previous illnesses occurring since last contact with or without medical treatment, and type of treatment sought, if any.

Trial staff will have access to both the internally designed android application and REDCap via unique usernames and passwords. Each trial staff member will have an appropriate level of access to CRFs and collected data, according to their roles and responsibilities within the trial.

All participant data will be stored and maintained on local servers in Bangladesh as mentioned above for the duration of the trial. Anonymized data will be shared with Oxford University in the UK. At the end of the trial, all individually identifiably data will be removed, and fully anonymized data will be retained for further analysis. At the end of the trial, all data and records will be electronically retained until the youngest child participating in the trial reaches 21 or for five years following completion of the research, whichever is longer.

Data Safety Monitoring Plan (DSMP)

All clinical investigations (research protocols testing biomedical and/or behavioural intervention(s)) should include the Data and Safety Monitoring Plan (DSMP). The purpose of DSMP is to provide a framework for appropriate oversight and monitoring of the conduct of clinical trials to ensure the safety of participants and the validity and integrity of the data. It involves involvement of all investigators in periodic assessments of data quality and timeliness, participant recruitment, accrual and retention, participant risk versus benefit, performance of trial sites, and other factors that can affect study outcome.

Quality assurance procedures

The study will be conducted in accordance with the current approved protocol, ICH GCP, relevant regulations and standard operating procedures.

Monitoring will be performed by representatives of the sponsor and according to the principles of ICH GCP. Data will be evaluated for compliance with the protocol and accuracy in relation to source documents. Following a risk based monitoring plan, the monitor will verify that the clinical study is conducted and data are generated, documented and reported in compliance with the protocol, GCP and the applicable regulatory requirements. As part of quality assurance procedures, an unblinded monitoring team will monitor all unblinded processes, including checking the unblinded vaccine accountability logs against the randomisation application. To maintain observer blinding, the unblinded monitoring team will be independent from the study team and will not take part in any other study related procedures.

Serious breaches

A serious breach is defined as "A breach of GCP or the trial protocol which is likely to affect to a significant degree –

(a) the safety or physical or mental integrity of the subjects of the trial; or

(b) the scientific value of the trial".

In the event that a serious breach is suspected the DSMB, IRB, CI at Oxford and Sponsor must be contacted within one (1) working day. In collaboration with the C.I., the serious breach will be reviewed by the IRB and the Sponsor and, if appropriate, the Sponsor will report it to the REC committee and Regulatory authority within seven (7) calendar days.

Ethical Assurance for Protection of Human rights

Describe the justifications for conducting this research in human participants. If the study needs observations on sick individuals, provide sufficient reasons for using them. Indicate how participants' rights will be protected, and if there would be benefit or risk to each participants of the study. Discuss the ethical issues related to biomedical and social research for employing special procedures, such as invasive procedures in sick children, use of isotopes or any other hazardous materials, or social questionnaires relating to individual privacy. Discuss procedures safeguarding participants from injuries resulting from study procedures and/or interventions, whether physical, financial or social in nature. [Please see Guidelines]

Ethical and regulatory considerations

Declaration of Helsinki

The Investigator will ensure that this trial is conducted in accordance with the principles of the Declaration of Helsinki.

Guidelines for Good Clinical Practice

The Investigator will ensure that this trial is conducted in accordance with relevant regulations and with Good Clinical Practice.

Approvals

The protocol, informed consent form, participant information sheet and any proposed advertising material will be submitted for approval to the icddr,b Research Review Committee, the Ethical Review Committee of icddr,b Research Ethics Committee (REC) at Oxford, regulatory authorities and other host institution(s) for written approval.

The Principal Investigator at icddr,b will submit and, where necessary, obtain approval from the above parties for all substantial amendments to the original approved documents.

Reporting

The PI shall submit once a year throughout the clinical trial, or on request, an Annual Progress Report to the ERC, REC in Oxford, other host organisations, and the Sponsor. In addition, an End of Trial notification and final report will be submitted to all committees and institutions mentioned above.

Participant Confidentiality

The trial staff will ensure that the participants' anonymity is maintained. The participants will be identified only by a participant ID number on all trial documents and any electronic database, with the exception of the CRF. All documents will be stored securely and only accessible by trial staff and authorised personnel. The trial will comply with the icddr,b Data policy as well as the UK Data Protection Act, which requires data to be anonymised as soon as it is practical to do so and local regulations.

Expenses and Benefits

There will not be any payments or reimbursements made to participants, as incentive for participant recruitment. It is anticipated that provision of vaccination will be enough incentive to reach the necessary sample size. Local hospitals, clinics, and vaccination points are being used to deliver all trial components, which will not add additional travel or expense to participants and their families.

The trial will cover the costs of standard care treatment for participants with suspected enteric fever, presenting with fever (≥2 days and/or 38ºC at presentation) as part of the trial, including the cost of test, antibiotics and/or other prescribed medications, and in-patient hospital stays and care, if medically necessary.
If participant presentation to hospital and ward trial clinics is less than expected, community health volunteers working in the trial area or pharmacy staff in the area may be provided nominal incentives for referring participants with febrile illness to the trial health care facilities.

Other Ethical Considerations

All efforts will be made to conduct the research in a way that is sensitive to the Bangladeshi culture and the social values. Bangladeshi trial staff will be present at all times during the consent process, and the participant study related materials (information sheet, consent forms, etc) will be printed the local language.

The study will be conducted based on the ethical requirements of the icddr,b. Children aged 9 months to <16 years have been selected because children bear a substantial burden of the disease in both mortality and morbidity, without an effective vaccine available. Therefore, this demographic group has most to gain from vaccination with the Vi-TCV and would be the primary target for any subsequent vaccination campaign.

The Japanese Encephalitis vaccine was selected as the control vaccine to ensure that the control group is receiving a beneficial intervention. The control vaccine will provide protection against Japanese Encephalitis, which is currently endemic to 24 countries in Asia, including Bangladesh, and can cause severe disease.

Samples and data collected may be shared with other researchers. For this purpose an MOU and MTA between icddr,b and the collaborating institute will be made. Only anonymised samples and data will be sent outside of icddr,b. At the end of the study, all remaining samples at the icddr,b will be kept for storage in the Biorepository, as required by the icddr,b policy. All remaining samples overseas will be kept for storage under the oversight of Oxford University. All samples will be kept for a minimum of 5 years after the end of the trial. New and better tests may become available in the future. Storage of these samples may also allow important future research to be done without needing to take new samples from Bangladeshi children.

Potential participants or their parents/guardians will be notified that they will be able to refuse to have the relevant biological samples stored, without this otherwise influencing participation in the study or the clinical care of their child. They will also be informed that should they no longer wish for their samples to be retained they may request their destruction.

Use of Animals

Describe if and the type and species of animals to be used in the study. Justify with reasons the use of particular animal species in the research and the compliance of the animal ethical guidelines for conducting the proposed procedures.

Not applicable.

Collaborative Arrangements

Describe if this study involves any scientific, administrative, fiscal, or programmatic arrangements with other national or international organizations or individuals. Indicate the nature and extent of collaboration and include a letter of agreement between the applicant or his/her organization and the collaborating

organization.

This project is a collaborative study between icddr,b and University of Oxford. The award from Bill and Melinda Gates Foundation (BMGF) to the University of Maryland, Baltimore, with the University of Oxford as a collaborating institution has been proceeded. MTA and MoU and other agreement is under process for this collaboration. After finalization, these documents will be submitted to the IRB.

Facilities Available

Describe the availability of physical facilities at site of conduction of the study. If applicable, describe the use of Biosafety Level 2 and/or 3 laboratory facilities. For clinical and laboratory-based studies, indicate the provision of hospital and other types of adequate patient care and laboratory support services. Identify the laboratory facilities and major equipment that will be required for the study. For field studies, describe the field area including its size, population, and means of communications plus field management plans specifying gender considerations for community and for research team members.

Mirpur Field Site: The Mirpur research site is located in the Dhaka Metropolitan area, approximately 7 km from the main icddr,b Dhaka hospital, and has been used for field studies since 1987. Mirpur is densely populated with approximately 3.5 million individuals. This research clinic has enjoyed a long standing relationship with this stable community. The Mirpur research clinic is composed of five floors, with each floor consisting of 4 rooms; approximately 7000 square feet of research space. This dedicated clinic building is situated within the same neighbourhood as the study population. The facility contains a subject waiting area, several examination rooms, a staff work and file room, a specimen processing area, archive room and a meeting room. The specimen processing area contains a refrigerator and a centrifuge with 24 hours generator back-up. The building has 24hrs security coverage and internet service. All clinical and laboratory specimens are sent to the clinical and research laboratories of the main icddr,b campus daily maintaining cold chain requirements. Numerous natural disease studies as well as GCP based vaccine trials have been conducted or planned in this site and include oral cholera, oral ETEC vaccine(s) and typhoid vaccine studies. For measuring the disease burden of typhoid fever in Mirpur area, a multi-site epidemiological study, named STRATAA, is being carried out. Socio-demographic information is also available from this area. A geographic information system (GIS) was used for mapping of this area and identifying households and other information of these households have been completed using PDA (Personal digital assistance) devices and android TABs.

The Mucosal Immunology & Vaccinology Laboratory at the icddr,b is a BSL2 level facility with internal and external quality assurance carried out for tests. All SOPs for studies are updated frequently for meeting study requirements. The laboratory is equipped with six biohazard safety hoods for processing of biological samples and for maintaining sterile conditions for specimen processing. For the fractionation of samples, refrigerated table top centrifuges, high speed centrifuges (Beckman) and ltracentrifuges (Beckman L7-80 and a Beckman L5-65B) are also available. Incubators with carbon-dioxide gas attachment are present for the study of B cell and T cell responses. There is a cryostat for sectioning of frozen sections and a microtome for paraffin sections. The ELISA readers are linked to a computer for determining antibody responses in study samples. Facilities for ELISPOT and “Antibody in Lymphocyte Supernatant” (ALS) assays are available. The CTL automatic counter as well as stereomicroscopes for enumeration of ASCs are also available. There are facilities for carrying out extraction of lymphocytes from gut biopsies. In addition, facilities for carrying out intestinal lavages and fecal extracts are used for assessing mucosal antibody responses. There are six -80°C low temperature freezers, two liquid nitrogen freezers, and a number of additional freezers (-20°C) and refrigerators used for storage of specimens vaccine trail and infectious disease.

Flow Cytometry Core facility is coordinated and used by this lab has a Becton Dickinson Facs Caliburs as well as FACSAria™ III cell sorter. The FACSAria™ III is currently configured with 3 lasers and capable

of detecting 9 fluorochromes (this capacity is expandable if needed) as well as a 96 well plate adapter for single cell sorting.

Literature Cited

Identify all cited references to published literature in the text by number in parentheses. List all cited references sequentially as they appear in the text. For unpublished references, provide complete information in the text and do not include them in the list of Literature Cited. There is no page limit for this section, however, exercise judgment in assessing the "standard" length.

1. Crump JA, Luby SP, Mintz ED. The global burden of typhoid fever. Bull World Health Organ. 2004;82(5):346–53.
2. Bhan MK, Bahl R, Bhatnagar S. Typhoid and paratyphoid fever. Lancet. 2005;366:749–62.
3. Bhutta ZA. Impact of age and drug resistance on mortality in typhoid fever. Arch Dis Child. 1996;75(3):214–7.
4. Lin FYC, Ho VA, Bay P V., Thuy NTT, Bryla D, Thanh TC, et al. The epidemiology of typhoid fever in the Dong Thap Province, Mekong Delta region of Vietnam. Am J Trop Med Hyg. 2000;62(5):644–8.
5. Simanjuntak CH, Totosudirjo H, Haryanto P, Suprijanto E, Paleologo FP, Punjabi NH, et al. Oral immunisation against typhoid fever in Indonesia with Ty21a vaccine. Lancet. 1991;338(8774):1055–9.
6. Sinha A, Sazawal S, Kumar R, Sood S, Reddaiah VP, Singh B, et al. Typhoid fever in children aged less than 5 years. Lancet. 1999;354(9180):734–7.
7. Ackers ML, Puhr ND, Tauxe R V, Mintz ED. Laboratory-based surveillance of Salmonella serotype Typhi infections in the United States: antimicrobial resistance on the rise. JAMA. 2000;
8. Steinberg EB, Bishop R, Haber P, Dempsey AF, Hoekstra RM, Nelson JM, et al. Typhoid fever in travelers: who should be targeted for prevention? Clin Infect Dis. 2004;
9. WHO. Typhoid vaccines: WHO position paper. Wkly Epidemiol Rec. 2008;83(6):49–59.
10. Mond JJ, Lees A, Snapper CM. T Cell-Independent Antigens Type 2. Annu Rev Immunol. 1995 Apr;13(1):655–92.
11. Weintraub A. Immunology of bacterial polysaccharide antigens. Carbohydrate Research. 2003.
12. Acharya IL, Lowe CU, Thapa R, Gurubacharya VL, Shrestha MB, Cadoz M, et al. Prevention of typhoid fever in Nepal with the Vi capsular polysaccharide of Salmonella typhi: A preliminary report. N Engl J Med. 1987;
13. Klugman K, Koornhof H, Schneerson R, Cadoz M, Gilbertson I, Robbins J, et al. Protective Activity of Vi Capsular Polysaccharide Vaccine Against Typhoid Fever. Lancet. 1987;
14. Yang HH, Wu CG, Xie GZ, Gu QW, Wang BR, Wang LY, et al. Efficacy trial of Vi polysaccharide vaccine against typhoid fever in south-western China. Bull World Health Organ. 2001;79(7):625–31.
15. Froeschle JE, Decker MD. Duration of Vi antibodies in participants vaccinated with Typhim Vi (Typhoid Vi polysaccharide vaccine) in an area not endemic for typhoid fever. Vaccine. 2010;
16. Michel R, Garnotel E, Spiegel A, Morillon M, Saliou P, Boutin JP. Outbreak of typhoid fever in vaccinated members of the French Armed Forces in the Ivory Coast. Eur J Epidemiol. 2005;
17. Pasetti MF, Simon JK, Sztein MB, Levine MM. Immunology of gut mucosal vaccines. Immunol Rev. 2011;
18. Cryz SJ, Vanprapar N, Thisyakorn U, Olanratmanee T, Losonsky G, Levine MM, et al. Safety and immunogenicity of Salmonella typhi Ty21a vaccine in young Thai children. Infection and Immunity. 1993.
19. Levine MM, Black RE, Ferreccio C, Germanier R. Large-scale field trial of TY21A live oral typhoid vaccine in enteric-coated casule formulation. Lancet. 1987;329(8541):1049–52.
20. Olanratmanee T, Levine M, Losonsky G, Thisyakorn U, Cryz SJ. Safety and Immunogenicity of Salmonella typhi Ty21a Liquid Formulation Vaccine in 4- to 6-Year-Old Thai Children. J Infect Dis. 1992;
21. Szu SC, Taylor DN, Trofa AC, Clements JD, Shiloach J, Sadoff JC, et al. Laboratory and preliminary clinical characterization of Vi capsular polysaccharide-protein conjugate vaccines. Infect Immun. 1994;
22. Lin FY, Ho VA, Khiem HB, Trach DD, Bay P V, Thanh TC, et al. The efficacy of a Salmonella

typhi Vi conjugate vaccine in two-to-five-year-old children. N Engl J Med. 2001;
23. Thiem VD, Lin FYC, Canh DG, Son NH, Anh DD, Mao ND, et al. The Vi conjugate typhoid vaccine is safe, elicits protective levels of IgG anti-Vi, and is compatible with routine infant vaccines. Clin Vaccine Immunol. 2011;18(5):730–5.
24. Mohan VK, Varanasi V, Singh A, Pasetti MF, Levine MM, Venkatesan R, et al. Safety and Immunogenicity of a Vi Polysaccharide-Tetanus Toxoid Conjugate Vaccine (Typbar-TCV) in Healthy Infants, Children, and Adults in Typhoid Endemic Areas: A Multicenter, 2-Cohort, Open-Label, Double-Blind, Randomized Controlled Phase 3 Study. Clin Infect Dis. 2015;61(3):393–402.
25. World Health Organization. Weekly epidemiological record. 2017 [cited 2017 Apr 25];2(92):13–20. Available from: http://www.who.int/wer.
26. Jin C. [Data from VAST trial - manuscript in progress]. 2017.
27. Darton TC, Meiring JE, Tonks S, Khan MA, Shakya M, Thindwa D, et al. The STRATAA Study Protocol: A programme to assess the burden of enteric fever in Bangladesh, Malawi, and Nepal using prospective population census, passive surveillance, serological studies and healthcare utilisation surveys. summitted.
28. Qadri F, Wierzba TF, Ali M, Chowdhury F, Khan AI, Saha A et al. Efficacy of a Single-Dose, Inactivated Oral Cholera Vaccine in Bangladesh. n engl j med 374;18 nejm.org May 5, 2016.

Budget Justifications

Please provide one page statement justifying the budgeted amount for each major item, including the use of human resources, major equipment, and laboratory services.

Budget for the typhoid conjugate vaccine study is needed for carrying out studies at the icddr,b hospital and the selected field sites of Mirpur area. Immunological and microbiological assays will be carried out at icddr,b. Other important costs are for local and international travel, transportation of vaccine, shipment, training, data management etc.

The total cost of the project has been calculated to be US$8,172,522 which comprises as follows:

Personnel: $5,184,812: Key personnel effort of Dr. Firdausi Qadri (PI) and Dr. John Clemens – 25% each for the year 1 to 3, and 30% each for year 4. The two will be involved in study design, protocol development, meeting with senior GOB (EPI, DCC) personnel and other decision making people, as well as be involved in the actual vaccination planning and program. Also, high level of effort will be needed to complete data analysis, dissemination of the results, manuscript writing for the last 2 years of the study.

The coordinators and co-investigators will function as lead personnel under supervision of the PI for evaluation, consent, enrolment, vaccine administration and monitoring.

Senior Research Investigators and medical Officers will be involved with passive surveillance and vaccine delivery. They will also assist with recruitment and protocol implementation to PI.

Research Investigators will be responsible for sample processing and also immunological and microbiological analysis.

Finance personnel, Project Research Managers, including other support staff needed for Data management, GIS, Census update, Passive surveillance, Quality team and Vaccine delivery program.

Travel: $196,158 including Local travel and Patient Follow-up Conveyance $121,658 and International travel $74,500 through the 4 years project period.

Consultants: Local consultants $294,153. These include support of EPI Specialist, Vaccine Delivery Expert and Data Management specialist.

Sub-award/Intl. Consultant/Service agreement: $207,600 including external experts. External part will be covered for support from foreign expertise, Prof. Ira Longini (University of Florida), Dr. Mohd. Ali, (JHU) and Ms. Yonghee (IVI).

Supplies: $281,425 Lab. Assays, sample testing, laboratory reagents, computer, tab, etc.

Other costs: $897,993, in 4 years including Clinical support at Mirpur Treatment Centre $290,000, Vaccine delivery costs $138,650, service agreement with different hospitals/institutes $15,600 and others $453,743 which includes meeting with different hospitals, high level GoB Officials (DGHS, EPI, Dhaka City), workshop, training, supervision, office rent, utilities, repair maintenance etc.

Equipment: $44,400 which includes Lab equipment, Freezer etc.
We have included $1,065,981 as indirect costs, calculated @15% rate on total direct costs $7,106,541.

Other Support

Describe sources, amount, duration, and grant number of all other research funding currently granted to PI or under consideration.

Not applicable.

Biography of the Investigators

Provide biographical data in the following format for all key personnel including the Principal Investigator. Copy the same format for each of them.
Note: Biography of the External Investigators may, however, be submitted in the format as convenient to them..

Biography of Firdausi Qadri

1 **Name:** Firdausi Qadri

2 **Present Position:** Acting Senior Director, Infectious Diseases Division

Head, Mucosal Immunology & Vaccinology laboratory, icddr,b

INSTITUTION AND LOCATION	DEGREE (if applicable)	YEAR(s)	FIELD OF STUDY
University of Dhaka, Bangladesh	B.S.	1975	Science
University of Dhaka, Bangladesh	Masters	1977	Biochemistry
Liverpool University, United Kingdom	Ph.D.	1980	Biochemistry
ICDDR,B, Bangladesh	Postdoctoral Fellowship	1986<br>1988	Immunology

3 **Educational background:**
(last degree and diploma and training relevant to the present research proposal)

4.0 **List of ongoing research protocols**
(start and end dates; and percentage of time)

4.1. As Principal Investigator

Protocol Number	Starting date	End date	Percentage of time
PR-09064	1-01-2010	31-05-2013	
PR-00240	9-11-2005	31-05-2013	
PR-00562	16-09-2005	31-12-2012	
PR-00708	01-08-2008	31-12-2012	
PR-10061	23-12-2010	22-12-2015	

4.2. As Co-Principal Investigator

Protocol Number	Starting date	End date	Percentage of time
PR-00240	9-11-2005	31-05-2013	
PR-00562	4-07-2006	31-03-2011	
PR-00707	1-08-2008	31-12-2011	
PR-00708	1-08-2008	31-07-2011	

4.3. As Co-Investigator

Protocol Number	Starting date	End date	Percentage of time

5 **Publications**

Types of publications	Numbers
a. Original scientific papers in peer-review journals	
b. Peer reviewed articles and book chapters	
c. Papers in conference proceedings	
d. Letters, editorials, annotations, and abstracts in peer-reviewed journals	
e. Working papers	

f. Monographs	

6 Five recent publications including publications relevant to the present research protocol

1. Uddin T, Harris JB, Bhuiyan TR, Shirin T, Uddin MI, Khan AI, Chowdhury F, Larocque RC, Alam MN, Ryan ET, Calderwood SB, **Qadri F**. Mucosal immunologic responses in cholera patients in Bangladesh.Clin Vaccine Immunol. 2011 Jan 19.
2. Matsuda F, Chowdhury MI, Saha A, Asahara T, Nomoto K, Tarique AA, Ahmed T, Nishibuchi M, Cravioto A, **Qadri F**. Evaluation of a probiotics, Bifidobacteriumbreve BBG-01, for enhancement of immunogenicity of an oral inactivated cholera vaccine and safety: A randomized, double-blind, placebo-controlled trial in Bangladeshi children under 5 years of age.Vaccine. 2011 Jan 12
3. Arifuzzaman M, Rashu R, Leung DT, Hosen MI, Bhuiyan TR, Bhuiyan MS, Rahman MA, Khanam F, Saha A, Charles RC, Larocque RC, Weil AA, Clements JD, Holmes RK, Calderwood SB, Harris JB, Ryan ET, **Qadri F**. Antigen-specific memory T cell responses after vaccination with an oral killed cholera vaccine in Bangladeshi children and comparison with natural cholera.Clin Vaccine Immunol. 2012 Jun 27.
4. Leung DT, Chowdhury F, Calderwood SB, **Qadri F**, Ryan ET.Immune responses to cholera in children.Expert Rev Anti Infect Ther. 2012;10(4):435-44.
5. Larocque RC, Sabeti P, Duggal P, Chowdhury F, Khan AI, Lebrun LM, Harris JB, Ryan ET, **Qadri F**, Calderwood SB A variant in long palate, lung and nasal epithelium clone 1 is associated with cholera in Bangladeshi population. Genes Immun. 2009 Apr;10(3):267-72.

BIOGRAPHICAL SKETCH

Provide the following information for the Senior/key personnel and other significant contributors.
Follow this format for each person. **DO NOT EXCEED FOUR PAGES.**

NAME<br>**John David Clemens**	POSITION TITLE<br>Executive Director, icddr,b and<br>Professor of Epidemiology, University of California, Los Angeles (UCLA), School of Public Health, USA
eRA COMMONS USER NAME (credential, e.g., agency login)	

EDUCATION/TRAINING *(Begin with baccalaureate or other initial professional education, such as nursing, include postdoctoral training and residency training if applicable.)*

INSTITUTION AND LOCATION	DEGREE *(if applicable)*	MM/YY	FIELD OF STUDY
Stanford University, Stanford, California, USA	BS	06/71	Biology
Yale University, New Haven, Connecticut, USA	MD	06/76	Medicine
Case Western Reserve University, Cleveland, Ohio, USA	--	06/79	Internship/Residency Internal Medicine
Yale University, New Haven, Connecticut, USA	--	06/81	Robert Wood Johnson Clinical Scholars Program

A. Personal Statement: Professor Clemens is an infectious disease epidemiologist with more than 30 years of experience designing, conducting, and analyzing large, population-based epidemiologic studies and vaccine field trials in developing countries, including Tanzania, Mozambique, Bangladesh, India, Pakistan, China, Indonesia, Vietnam, and Chile. His research work on bacterial enteric pathogens has included studies on salmonellosis, shigellosis, cholera, and ETEC. Several of these studies have entailed use of GIS, genotyping of pathogens, modeling, as well as conventional cohort and case-control designs to evaluate candidate risk factors. Currently, Professor Clemens has been serving in the icddr,b as Executive Director and co-investigator of a good number of research projects.

B. Positions and Honors

Positions and Employment

1979-1985 Assistant Professor of Medicine, Yale University School of Medicine, USA
1983-1988 Scientist, International Centre for Diarrhoeal Disease Research (icddr,b), Bangladesh
1988-1990 Associate Professor of Medicine and Chief, Epidemiology Section, Center for Vaccine Development, University of Maryland School of Medicine, USA
1990-1999 Senior Investigator and Chief of the Epidemiology Branch, Division of Epidemiology, Statistics and Prevention Research, National Institute of Child Health and Human Development
1996-1999 Director, World Health Organization Collaborating Center for the Clinical Evaluation of Vaccines in Developing Countries
1999-2011 Director General, International Vaccine Institute (ivi), Seoul, Korea
2000-2011 Adjunct Professor, Seoul National University School of Public Health
2011- Professor of Epidemiology and Founding Director, Center for Global Infectious Diseases, UCLA School of Public Health, USA and
2013- Executive Director; icddr,b; Dhaka, Bangladesh

Other Experience and Professional Memberships

1991-2010 Member, Steering Committee on Bacterial Enteric Vaccines, Initiative for Vaccine Research, World Health Organization, Geneva
1989- Member, American Epidemiological Society

1990- Fellow, American College of Epidemiology
1992- Fellow, Infectious Diseases Society of America
1992- US Panel on Cholera and Related Bacterial Diarrheas, U.S - Japan Cooperative Medical Science Program Member: 1992-2007
Chairman: 2007-
1997-2009 Associate Editor, American Journal of Epidemiology
1998 Advisor, World Health Organization,
Project Development Team for Field Trials of Typhoid Vaccines in Uzbekistan
1999-2011 Member, Board of Trustees, International Vaccine Institute (ivi), South Korea
2000- Editorial Advisory Board, Journal of Health, Population, and Nutrition, icddr,b
2000- Editorial Board, Microbes and Infection
2006 Member, UK Medical Research Medical Research Council Expert Advisory Committee on Priorities for Vaccine Research
2007 Member, Advisory Council, Initiative for Vaccine Research, World Health Organization,Geneva
2007- Editorial Board, ExpertReviewofVaccines
2007-2009 Member, GAVI Alliance Board, Representing Technical and Research Organizations
2011 Editorial Board, Emerging Microbes and Infections

Honors

1970 Phi Beta Kappa, Stanford University, USA
1994 US Centers for Disease Control Citation for Outstanding Contribution to Public Health Education for Vibriocholerae and Cholera
1995 US NIH Director's Award for Outstanding Research on Vaccine Evaluation
1999 Delta Omega (Honorary Public Health Society), Johns Hopkins Chapter, USA
2009 NICED (National Institute of Cholera and Enteric Diseases of India) Award
2010 Sabin Gold Medal Award
2011 Honorary Doctor of Philosophy (Medicine), Soonchunhyang University, Korea

C. Selected Peer-reviewed Publications (Selected from 338 peer-reviewed publications)

1. Clemens J, Sack D, Harris J, Chakraborty J, Khan MR, Stanton B, Kay BA, Khan MU, Yunus M, Atkinson W, Svennerholm A-M, Holmgren J. Field trial of oral cholera vaccines in Bangladesh. Lancet 2:124-127, 1986.

2. Clemens J, Stanton B. An educational intervention for altering water-sanitation behaviours to reduce childhood diarrhoea in Bangladesh: I. Application of the case-control method for development of an intervention. AmericanJournalofEpidemiology 125:284-291, 1987.

3. Stanton B, Clemens J. An educational intervention for altering water-sanitation behaviours to reduce childhood diarrhoea in Bangladesh: II. A randomized trial to assess the impact of the intervention on hygienic behaviours and diarrhoea rates. AmericanJournalofEpidemiology 125:292-301, 1987.

4. Clemens JD, Sack D, Harris JR, Chakraborty J, Khan MR, Stanton B, Ali M, Ahmed F, Yunus M, Kay B, Khan MU, Rao MR, Svennerholm A-M, Holmgren J. Impact of B subunit killed whole-cell and killed whole-cell-only oral vaccines against cholera upon treated diarrhoeal illness and mortality in an area endemic for cholera. Lancet 2:1375-1378, 1988.

5. Clemens J, Sack DA, Harris J, van Loon F, Chakraborty J, Ahmed F, Rao MR, Khan MR, Yunus M, Huda N, Stanton B, Kay B, Walter S, Eeckels R, Svennerholm A-M, Holmgren J. Field trial of oral cholera vaccines in Bangladesh: Results from long-term follow-up. Lancet 335: 270-3, 1990.

6. Trach DD, Clemens JD, Ke NT, Thuy HT, Son ND, Canh DG, Hang PVD, Rao MR. Field trial of a locally produced, killed oral cholera vaccine in Vietnam. Lancet 349: 231-5, 1997.

7. Naficy A, Rao M, Paquet C, Antona D, Sorkin A, Clemens J. Treatment and vaccination strategies for the control of cholera epidemics in established Sub-Saharan refugee settings: a cost-effectiveness analysis. JournaloftheAmericanMedicalAssociation 279: 521-6, 1998.

8. Lucas M, Deen JL, von Seidlein L, Wang X-Y, Ampuero J, Puri M, Ali M, Ansaruzzaman M, Amos J, Cavallier P, Guerin P, McChesney M, Mahoudeau C, Kahozi P, Chaignat C-Barreto A, Songane F, Clemens JD. The effectiveness of an internationally licensed, two dose oral cholera vaccine when given in a mass vaccination campaign in Beira, Mozambique. New EnglandJournalofMedicine 352: 757-67, 2005.

9. Clemens J, Jodar L. Introducing new vaccines into developing countries: obstacles, opportunities, and complexities.Nature(Medicine) 11(4 Suppl): S12-15, 2005.

10. Ali M, Emch M, von Seidlein L, Yunus M, Sack D, Rao M, Holmgren J, Clemens J. Herd immunity conferred by killed oral cholera vaccines in Bangladesh. Lancet 366:44-49, 2005

11. Longini I, Nizam A, Ali M, Yunus M, Shenvi N, Clemens J. Controlling endemic cholera with oral vaccines. PLoSMedicine 4:e336, 2007.

12. Sur D, Ochiai RL, Sujit K, Bhattacharya S, Ganguly N, Ali M, Manna B, Dutta S, Donner A, Kanungo S, Park JK, Puri M, Kim DR, Dutta D, Bhaduri B, Acosta C, Clemens J. A cluster-randomized effectiveness trial of Vi typhoid vaccine in India. NewEnglandJournalofMedicine 361: 335-44, 2009.

13. Jeuland M,. Cook J, Poulos C, Clemens J, Whittington D. Cost-effectiveness of new generation oral cholera vaccines: a multi-site analysis. ValueinHealth 12: 899-908, 2009.

14. **Sur D, Lopez A, Kanungo S, Paisley A, Manna B, Al M, Niyogi S, Park JK, Sarkar B, Pur M, Kim D, Deen J, Holmgren J, Carbis R, Rao R, Nguyen TV, Donner A, Ganguly NK, Nair GB, Bhattacharya SK, Clemens JD. Protection and safety of a modified, killed whole cell oral cholera vaccine in India: a cluster-randomized, double-blind, placebo-controlled trial. Lancet 374: 1694-702, 2009.**

15. Waldor M, Hotez P, Clemens J. A national cholera vaccine stockpile - a new humanitarian and diplomatic resource. NewEnglandJournalofMedicine 363: 2279-82, 2010.

16. Clemens J, Shin S, Sur D, Nair GB, Holmgren. New generation vaccines against cholera. NatureReviewsinGastroenterologyandHepatology. November 8 (Epub ahead of print), 2011.

D. Research Support

Ongoing Research Support

5 U01 CI000298-02 and **1 U01 CI000628-01** Steve Luby& Leanne Unicomb 09/30/04 - 09/29/14 (120 Calendar Months)
Donor - Center for Disease Control (CDC), Atlanta, USA
US$ 23,051,834
Program: Addressing Emerging Infectious Disease in Bangladesh. The main objective of this program is to conduct research to find out public health measures to reduce infectious diseases in Bangladesh.
Role: Dr. Clemens is the Scientific and Administrative guide as Executive Director of icddr,b.
GFATM - RCC Program: BAN-202-G13-H-00 TasnimAzim
12/01/09 - 11/30/15 (72 Calendar Months)

Donor - Global Fund to Fight AIDS, Tuberculosis and Malaria (GFATM) **US$ 22,540,005**
Program: Expanding HIV Prevention in Bangladesh under Rolling Continuation Channel (RCC Program Phase I and II)
Role: Dr. Clemens is the Scientific and Administrative guide as Executive Director of icddr,b.

Gates Foundation: Grant# 38874 Scientists of icddr,b and CHRF, Dhaka 09/29/10-08/31/15 (60 Calendar Months)
Donor - Bill & Melinda Gates Foundation, USA
US$ 21,491,580

Program: AEtiology of Neonatal Infection in South Asia (ANISA) Child Health Research Foundation (CHRF) Fund Management Cell. The main objective of this program is to conduct collaborative research by Scientists of the icddr,b and Child Health Research Foundation to find out the Etiology of Neonatal Infection in South Asia.
Role: Dr. Clemens is the Scientific and Administrative guide as Executive Director of icddr,b.

GHS-A-00-09-00015-00 Abbas Bhuiya
02/14/11- 09/30/14 (40 Calendar Months)
Donor - USAID, Washington/University Research Co., LLC, USA
US$ 18,000,000
Program: Implementation Research for Testing Effective Strategies to Expand and Improve Maternal, Newborn and Child Health, Nutrition, Reproductive Health/Family Planning and Tuberculosis Services in Bangladesh. The main objective of this project is to conduct research to find out Effective Strategies to Expand and Improve Maternal, Newborn and Child Health, Nutrition, Reproductive Health/Family Planning and Tuberculosis Services.
Role: Dr. Clemens is the Scientific and Administrative guide as Executive Director of icddr,b.

Gates Foundation: Global Health GR#OPP50419 Firdausi Qadri 09/01/09 - 08/31/14 (60 Calendar Months)
Donor - Bill & Melinda Gates Foundation, USA
US$ 16,621,229
Program: Impact evaluation of cholera vaccine and behavior (Introduction of cholera vaccine in Bangladesh)
The main objective of this project is to understand the impact of large scale interventions using cholera vaccine or promoting hygiene and use of safe water in reducing the high burden of severe dehydrating diarrhoea requiring hospitalization in resource poor, cholera prone urban setting in Dhaka city so as to implement a nationwide program in Bangladesh.
Role: Dr. Clemens is the Scientific and Administrative guide as Executive Director of icddr,b.

Gates Foundation: OPPGD759/Sub 00006966 James Heffelfinger
09/17/09 - 09/30/14 (60 Calendar Months)
Donor - University of California, Berkeley/Bill & Melinda Gates Foundation, USA **US$ 6,274,083**
Program: Measuring the Benefits of Sanitation, Water Quality and Handwashing Interventions for Improving Health and Development (WASH Benefits). The main objective of this program is to assess Benefits of Sanitation, Water Quality and Handwashing Interventions for Improving Health and Development in Bangladesh.
Role: Dr. Clemens is the Scientific and Administrative guide as Executive Director of icddr,b.

AmeriCares, USA Pradip K Bardhan 01/01/12 - 12/31/14 (36 Calendar Months)
Donor - AmeriCares Foundation (AC), USA **US$ 5,445,866**
Program: Global Medical Assistance from AmeriCares for Dhaka Hospital. The main objective of this program is to set up a standard Global Medical Assistance to be provided to the People of Bangladesh by Dhaka, icddr,b.
Role: Dr. Clemens is the Scientific and Administrative guide as Executive Director of icddr,b.

JHBSPH/ Gates Foundation:OPP52956 Abdullah Brooks
10/20/09 - 12/31/13 (51 Calendar Months)
Donor - Johns Hopkins University, USA/ Bill & Melinda Gates Foundation, USA
US$ 5,305,601
Program: Influenza Vaccine Efficacy against Childhood Pneumonia in a Low-Income Tropical Urban Community with its main objective is to administer Influenza Vaccine to assess the Efficacy against Childhood Pneumonia in a Low-Income Tropical Urban Community in Bangladesh.
Role: Dr. Clemens is the Scientific and Administrative guide as Executive Director of icddr,b.

<u>Completed Research Support</u>

Gates Foundation: IVI Project John D Clemens
2006 - 2012

Donor - Bill & Melinda Gates Foundation, USA **US$ $3,160,000**
Program: The Cholera Vaccine Initiative
Main Objective: The major goals of this project are to accelerate the development, licensure, and introduction of oral vaccines against cholera.
Role: Dr. Clemens was the Scientific and Administrative chief as Principal Investigator of the project.

Gates Foundation: WHO Project Claire-LiseChaignat and John D Clemens 2007 - 2010
Donor - Bill & Melinda Gates Foundation, USA **US$ $3,160,000**
Program: Evaluation Killed Oral Cholera Vaccine in Zanzibar
Main Objective: The major goals of this project are to evaluate the costs, acceptability and impact of killed oral cholera vaccine when delivered to the general population of Zanzibar.
Role: Dr. Clemens was the Scientific and Administrative chief as Principal Investigator of the sub-contract.

1. **Name: Dr Farhana Khanam**

2. **Present Position: Deputy Project Coordinator**

3. **Educational background**:

	Institution	Year
Master of Philosophy (M. Phil) on Microbiology	Dhaka Medical College and Hospital, University of Dhaka	2010
MBBS	Sir Salimullah Medical College and Hospital, University of Dhaka	2002

4. **Ethics Certification:**

		If Yes		
		Issuing Authority	Registration No	Valid Until
**No** ☐	Yes ☒	The National Institutes of Health (NIH) Office of Extramural Research	1430942	

5. **List of ongoing research protocols/ activities**

Protocol/ Activity Number	Role in the protocol/ activity (PI, Co-PI, Co-I)	Starting date	End date	Percentage of time
PR#15119	Co-I			
PR#14127	Co-I			
PR#13014	Co-I			

6. **Publications**

Types of publications	Numbers
g. Original scientific papers in peer-review journals	25
h. Peer reviewed articles and book chapters	
i. Papers in conference proceedings	
j. Letters, editorials, annotations, and abstracts in peer-reviewed journals	
k. Working papers	
l. Monographs	

7. **Five recent publications including publications relevant to the present research protocol**

7.1. Darton TC, Meiring JE, Tonks S, Khan MA, **Khanam F**, Shakya M, Thindwa D, Baker S, Basnyat B, Clemens JD, Dougan G, Dolecek C, Dunstan SJ, Gordon MA, Heyderman RS, Holt KE, Pitzer VE, Qadri F, Zaman K, Pollard AJ; STRATAA Study Consortium. The STRATAA study protocol: a programme to assess the burden of enteric fever in Bangladesh, Malawi and Nepal using prospective population census, passive surveillance, serological studies and healthcare utilisation surveys. BMJ Open. 2017 Jul 2;7(6).

7.2. Bhuyan GS, Hossain MA, Sarker SK, Rahat A, Islam MT, Haque TN, Begum N, Qadri SK, Muraduzzaman AK, Islam NN, Islam MS, Sultana N, Jony MH, **Khanam F**, Mowla G, Matin A, Begum F, Shirin T, Ahmed D, Saha N, Qadri F, Mannoor K. Bacterial and

viral pathogen spectra of acute respiratory infections in under-5 children in hospital settings in Dhaka city. PLoS One. 2017 Mar 27;12(3).

7.3. Park KS, Chung HJ, **Khanam F**, Lee H, Rashu R, Bhuiyan MT, Berger A, Harris JB, Calderwood SB, Ryan ET, Qadri F, Weissleder R, Charles RC. A magneto-DNA nanoparticle system for the rapid and sensitive diagnosis of enteric fever. Sci Rep. 2016 Sep 8;6.

7.4. Qadri F, Wierzba TF, Ali M, Chowdhury F, Khan AI, Saha A, Khan IA, Asaduzzaman M, Akter A, Khan A, Begum YA, Bhuiyan TR, **Khanam F**, Chowdhury MI, Islam T, Chowdhury AI, Rahman A, Siddique SA, You YA, Kim DR, Siddik AU, Saha NC, Kabir A, Cravioto A, Desai SA, Singh AP, Clemens JD. Efficacy of a Single Dose Inactivated Oral Cholera Vaccine in Bangladesh. n engl j med 374;18 nejm.org May 5, 2016.

7.5. Khan IH, Sayeed MA, Sultana N, Islam K, Amin J, Faruk MO, Khan U, **Khanam F**, Ryan ET, Qadri F. Development of a simple, peripheral blood-based lateral-flow dipstick assay for accurate detection of patients with enteric fever. Clin Vaccine Immunol. 2016 Mar 9. pii: CVI.00690-15.

Biography of Dr.Md.Arifuzzaman Khan

OMB No. 0925-0001 and 0925-0002 (Rev. 10/15 Approved Through 10/31/2018)

BIOGRAPHICAL SKETCH Applicant **Dr.Md.Arifuzzaman Khan**

Provide the following information for the Senior/key personnel and other significant contributors. Follow this format for each person. DO NOT EXCEED FIVE PAGES.

NAME: Khan, Md Arifuzzaman

eRA COMMONS USER NAME (credential, e.g., agency login):

POSITION TITLE: Research Fellow, CSF Global , Bangladesh

EDUCATION/TRAINING *(Begin with baccalaureate or other initial professional education, such as nursing, include postdoctoral training and residency training if applicable.)*

INSTITUTION AND LOCATION	DEGREE (if applicable)	START DATE MM/YYYY	END DATE MM/YYYY	FIELD OF STUDY
Mymensingh Medical College under University of Dhaka	MBBS	05/2001	10/2006	Medicine
International Vaccine Institute, Seoul	Other training	05/2014	05/2014	International Advanced Course on Vaccinology
icddr,b, Dhaka	Other training	11/2014	12/2014	Evening course on Epidemiological Research Methods and Statistical Package for the Social Sciences (SPSS)
PPD, Caetown	Other training	10/2015	10/2015	Train-The-Trainer workshop on GCP
University of Development Alternative, Dhaka	Other training	07/2016	09/2016	12 weeks certificate course on ‘Introduction to Bioinformatics Methods'

A. Personal Statement

I hold a bachelor degree in medicine ,MBBS – a 5 years medical degree which included community medicine course in third and fourth year that required a research work. I also took part in community placement during the subsequent one year clinical internship. I currently hold the position of research fellow of CSF Global, Bangladesh where I research on neurodevelopment among children, focusing on exploration of causes of disability. I have been conducting different epidemiological and immunological studies as a research clinician for past 6 years with icddr,b and co-authored 6 publications in peer reviewed journals. I have also recently submit first authored paper in IDDT (peer reviewed).

I was first exposed to the community medicine course while I was a third year medical student. And at the end of my medical courses- before the beginning of my internship- I decided myself to build up my career in the field of public health. After the completion of internship I joined in icddr,b as a clinical fellow. My public health career started when I was recruited as a medical officer to a vaccine study in the beginning of 2011 with the project entitled ' Introduction of

cholera vaccine in Bangladesh' which is one of the largest feasibility trials of oral cholera vaccine (OCV). We vaccinated around 160000 people from high risk area of Mirpur Dhaka. We completed the campaign with two doses of Shanchol over a period of two months. It was an enormous experience for me regarding vaccine delivery, AEFI, establishing passive surveillances to determine the effectiveness of vaccine. I co-authored the paper published in The Lancet with the findings from this trial. Later in 2014, I become a co investigator of Phase III individually randomized double blinded placebo controlled trial of single dose OCV (Vaccinated more than 200,000 individuals), results of six months have published in NEJM. I also worked in a small group to design an experiment to the test the various aspects of field applications of OCV i.e. heat stability, dosing schedule and memory responses (published in Vaccine 2015, I am 2nd author). Based on my research experiences, I was promoted to senior research investigator and since, have been working in research projects as a co-investigator/study coordinator; that includes 'Phase I/II ETEC ETVAX Vaccine Trial in Bangladesh' and other PhaseI/II vaccine trials of locally produced OCV.I am now mostly involved with the study called 'STRATAA-Strategic Typhoid Alliance across Africa and Asia' (I also attended STRATAA investigator meeting in Oxford,UK and Kampala,Uganda). I am one of the co-investigators here in Bangladesh. My major responsibilities are vaccine trial management, protocol and SOP development, conduct protocol specific training, immunological data analysis, manuscript writing and submission, training staff, attending national and international seminars, etc. I have an academic training in 'International Advanced Course on Vaccinology' offered by the International Vaccine Institute (IVI), Seoul, South Korea and also have some other trainings on Epidemiology and Biostatistics form icddr, b. In last year (October 2015), I got the training from GCP train the trainer workshop by PPD from Capetown,SA. I have received a travel grant from PATH and attended the PATH's First International Vaccines Against Shigella and Enterotoxigenic Escherichia coli (VASE) Conference, took place at the Willard InterContinental Hotel Washington, DC, USA during at June, 2016. I have also learnt bioinformatics from a workshop, which suppose to help me increasing my efficiency in the field of biomedical research. I have experiences of directly helping my principle investigator of successful submission in top rated journal like 'The Lancet' and 'NEJM'.

1. Akhtar M, Qadri F, Bhuiyan TR, Akter S, Rafique TA, Khan A, Islam LN, Saha A, Svennerholm AM, Lundgren A. Kinetics of antibody-secreting cell and fecal IgA responses after oral cholera vaccination in different age groups in a cholera endemic country. Vaccine. 2017 Jan 5;35(2):321-328. PubMed PMID: 27916412.
2. Qadri F, Wierzba TF, Ali M, Chowdhury F, Khan AI, Saha A, Khan IA, Asaduzzaman M, Akter A, Khan A, Begum YA, Bhuiyan TR, Khanam F, Chowdhury MI, Islam T, Chowdhury AI, Rahman A, Siddique SA, You YA, Kim DR, Siddik AU, Saha NC, Kabir A, Cravioto A, Desai SN, Singh AP, Clemens JD. Efficacy of a Single-Dose, Inactivated Oral Cholera Vaccine in Bangladesh. N Engl J Med. 2016 May 5;374(18):1723-32. PubMed PMID: 27144848.
3. Saha A, Khan A, Salma U, Jahan N, Bhuiyan TR, Chowdhury F, Khan AI, Khanam F, Muruganandham S, Reddy Kandukuri S, Singh Dhingra M, Clemens JD, Cravioto A, Qadri F. The oral cholera vaccine Shanchol™ when stored at elevated temperatures maintains the safety and immunogenicity profile in Bangladeshi participants. Vaccine. 2016 Mar 18;34(13):1551-1558. PubMed PMID: 26896684.
4. Qadri F, Ali M, Chowdhury F, Khan AI, Saha A, Khan IA, Begum YA, Bhuiyan TR, Chowdhury MI, Uddin MJ, Khan JAM, Chowdhury AI, Rahman A, Siddique SA, Asaduzzaman M, Akter A, Khan A, Ae You Y, Siddik AU, Saha NC, Kabir A, Riaz BK, Biswas SK, Begum F, Unicomb L, Luby SP, Cravioto A, Clemens JD. Feasibility and effectiveness of oral cholera vaccine in an urban endemic setting in Bangladesh: a cluster randomised open-label trial. Lancet. 2015 Oct 3;386(10001):1362-1371. PubMed PMID: 26164097.

B. Positions and Honors

<u>Positions and Employment</u>

2016 -	Research Fellow, CSF Global, Dhaka,Bangladesh
2015-	Senior Research Investigator, icddr,b, Dhaka,Bangladesh
2012 - 2015	Research Investigator, icddr,b, Dhaka,Bangladesh
2011-2012	Research Investigator, icddr,b, Dhaka,Bangladesh
2010-2011	Research Physician, Child Health Research Foundation (CHRF),Dhaka Shishu Hospital, Dhaka, Bangladesh
2009-2010	Clinical Fellow, Matlab Hospital, icddr'b , Bangladesh

Other Experience and Professional Memberships

2007 -	Registered as Physician ; Registration No: A 47057 , of Bangladesh from Bangladesh Medical & Dental Council
2015 -	Global Outreach Member ;American Society for Microbiology(ASM),Member#57195265 , American Society for Microbiology(ASM)
2015 -	Life member, One Health , Bangladesh
2016 -	Certified EpiCore Member, Epicore
2016 -	Adjunct Research Fellow, Asian Institute of Disability and Development(AIDD)

C. Contribution to Science

1. I have in my career conducted several clinical trials and epidemiological studies which contributed to global health for the prevention of enteric infections in lower middle income countries

    a. Darton TC, Meiring JE, Tonks S, Khan MA, Khanam F, Shakya M, Thindwa D, Baker S, Basnyat B, Clemens JD, Dougan G, Dolecek C, Dunstan SJ, Gordon MA, Heyderman RS, Holt KE, Pitzer VE, Qadri F, Zaman K, Pollard AJ. The STRATAA study protocol: a programme to assess the burden of enteric fever in Bangladesh, Malawi and Nepal using prospective population census, passive surveillance, serological studies and healthcare utilisation surveys. BMJ Open. 2017 Jul 2;7(6):e016283. PubMed PMID: 28674145.

    b. Khan AI, Ali M, Chowdhury F, Saha A, Khan IA, Khan A, Akter A, Asaduzzaman M, Islam MT, Kabir A, You YA, Saha NC, Cravioto A, Clemens JD, Qadri F. Safety of the oral cholera vaccine in pregnancy: Retrospective findings from a subgroup following mass vaccination campaign in Dhaka, Bangladesh. Vaccine. 2017 Mar 13;35(11):1538-1543. PubMed PMID: 28196715; PubMed Central PMCID: PMC5341737.

    c. Saha A, Khan A, Salma U, Jahan N, Bhuiyan TR, Chowdhury F, Khan AI, Khanam F, Muruganandham S, Reddy Kandukuri S, Singh Dhingra M, Clemens JD, Cravioto A, Qadri F. The oral cholera vaccine Shanchol™ when stored at elevated temperatures maintains the safety and immunogenicity profile in Bangladeshi participants. Vaccine. 2016 Mar 18;34(13):1551-1558. PubMed PMID: 26896684.

    d. Qadri F, Ali M, Chowdhury F, Khan AI, Saha A, Khan IA, Begum YA, Bhuiyan TR, Chowdhury MI, Uddin MJ, Khan JAM, Chowdhury AI, Rahman A, Siddique SA, Asaduzzaman M, Akter A, Khan A, Ae You Y, Siddik AU, Saha NC, Kabir A, Riaz BK, Biswas SK, Begum F, Unicomb L, Luby SP, Cravioto A, Clemens JD. Feasibility and

effectiveness of oral cholera vaccine in an urban endemic setting in Bangladesh: a cluster randomised open-label trial. Lancet. 2015 Oct 3;386(10001):1362-1371. PubMed PMID: 26164097.

e. Akhtar M, Qadri F, Bhuiyan TR, Akter S, Rafique TA, Khan A, Islam LN, Saha A, Svennerholm AM, Lundgren A. Kinetics of antibody-secreting cell and fecal IgA responses after oral cholera vaccination in different age groups in a cholera endemic country. Vaccine. 2017 Jan 5;35(2):321-328. PubMed PMID: 27916412.

f. Qadri F, Wierzba TF, Ali M, Chowdhury F, Khan AI, Saha A, Khan IA, Asaduzzaman M, Akter A, Khan A, Begum YA, Bhuiyan TR, Khanam F, Chowdhury MI, Islam T, Chowdhury AI, Rahman A, Siddique SA, You YA, Kim DR, Siddik AU, Saha NC, Kabir A, Cravioto A, Desai SN, Singh AP, Clemens JD. Efficacy of a Single-Dose, Inactivated Oral Cholera Vaccine in Bangladesh. N Engl J Med. 2016 May 5;374(18):1723-32. PubMed PMID: 27144848.

a.

D. Additional Information: Research Support and/or Scholastic Performance

i. icddr,b protocol no : PR-17016
A Randomized Controlled Trial of Influenza Vaccine to Reduce Adverse Vascular Events
Roll- Co-Investigator
Sponsor: McMaster University, Canada.Funded by: McMaster University, Canada. (2017-2019)
ClinicalTrials.gov Identifier: NCT02762851

ii. icddr,b protocol no : PR-17044
T cell responses to enterotoxigenic Escherichia coli infection. (2017-2019)
Roll- Co-Investigator
Sponsor: icddr,b Funded by: Sida Research fund

iii. icddr,b protocol no : PR-16021
Safety and immunogenicity of locally manufactured new (HL-OCV) oral cholera vaccine (2016-2018)
Roll- Co-Investigator
Sponsor: MSD Wellcome Trust Hilleman Laboratories (P) Ltd. Funded by: Bill & Malinda Gates Foundation
ClinicalTrials.gov Identifier: NCT02823899

iv. icddr,b protocol no : PR-15119
Typhoid alliance across Africa and Asia (STRATAA). (2015-2017)
Roll- Co-Investigator
Sponsor: Oxford Vaccine group (OVG), Funded by: Wellcome Trust
ISRCTN12131979

v. icddr,b protocol no PR-14127/VAC014/OEV-122
ETEC ETVAX Vaccine trial in Bangladesh. (2014-2017)
Roll- Co-Investigator and Study Coordinator.
Sponsor: PATH ClinicalTrials.gov Identifier: NCT02531802

vi. icddr,b protocol no PR-13013

Enterotoxigenic E. coli (ETEC) diarrhoea: study of prevalence, phenotype and immune responses
(2013-2015)
Roll-Co-Investigator
Funded by : icddr,b core fund.

vii. icddr,b protocol no PR-12090
Single Dose Oral Cholera Vaccine Study in Dhaka, Bangladesh (SCVB) (2012-2015)
Roll- Co-Investigator
Sponsor: IVI ClinicalTrials.gov Identifier: NCT02027207

viii. icddr,b protocol no PR-12030
Study of field application of oral cholera vaccine, Shanchol for use in developing country settings. (2012-2015)
Role-Member of scientific team.
Sponsor:icddr,b Funded by Bill & Melinda Gates Foundation
ClinicalTrials.gov Identifier: NCT01762930

ix. icddr,b protocol no PR-10061
Introduction of Cholera Vaccine in Bangladesh (ICVB) (2011-2015)
Roll- Medical Officer
Sponsor: IVI, Funded by Bill & Melinda Gates Foundation
ClinicalTrials.gov Identifier: NCT01339845

Biography of Dr.Fahima Chowdhury

1 **Name: Dr.Fahima Chowdhury**
2 **Present Position:** Project Coordinator**;** Infectious Diseases Division, icddr,b.
3 **Educational background:**
(last degree and diploma and training relevant to the present research proposal)

INSTITUTION AND LOCATION	DEGREE *(if applicable)*	YEAR(s)	FIELD OF STUDY
AgraniBalikaBiddalaya, Dhaka	SSC	1984	Science
Holy Cross College, Dhaka	HSC.	1986	Science
Mymensingh Medical college	M.B.B.S.	1994	Medicine
North South University	M.P.H.	2007	Epidemiology

4.0List of ongoing research protocols
(start and end dates; and percentage of time)
4.1 As Principal Investigator

Protocol Number	Starting date	End date	Percentage of time
15076	15/09/2015	-	70%

4.2 As Co-Principal Investigator

Protocol Number	Starting date	End date	Percentage of time
2008-019	01/06/08	30/05/10	50%

4.3 As Co-Investigator

Protocol Number	Starting date	End date	Percentage of time
2005-030	01.12.2006	30.08.2010	80%
2007-007	01.04.2007	31.03.2009	10%
10061	01.09.2009	31/12/2014	90%

5 **Publications**

Types of publications	Numbers
a. Original scientific papers in peer-review journals	29
b. Peer reviewed articles and book chapters	1
c. Papers in conference proceedings	10
d. Letters, editorials, annotations, and abstracts in peer-reviewed journals	0
e. Working papers	0
f. Monographs	0

6 **Five recent publications including publications relevant to the present research protocol**

1. A variant in long palate, lung and nasal epithelium clone 1 is associated with cholera in a Bangladeshi population.Larocque RC, Sabeti P, Duggal P, **Chowdhury F**, Khan AI,

Lebrun LM, Harris JB, Ryan ET, Qadri F, Calderwood SB. Genes Immun. 2009; 10(3):1-6

2. Chowdhury **F** , Mohammad ArifRahman,Yasmin Begum, Ashraful Khan, Abu Faruque, NirodSaha , Nabilah Baby , M.A. Malek , Anisha Kumar , Ann-Mari Svennerholm, Mark Pietroni, Alejandro Cravioto 'Impact of Rapid Urbanization on the Rates of Infection by Vibrio cholerae O1 and Enterotoxigenic Escherichia coli in Dhaka, Bangladesh. Accepted inPLoS Neglected Tropical Diseases on 5th March 2011
3. **Chowdhury F**, Yasmin A. Begum, Mohammad MurshidAlam, AshrafulI.Khan, Alejandro Cravioto, Firdausi Qadri et al. Concomitant Enterotoxigenic*E. coli* Infection Induces Increased Immune Responses to *Vibrio cholerae* O1 Antigens in Patients with Cholera in Bangladesh. Infection and immunity. 2010; 78(5): 2117–2124
4. **Chowdhury F,** Khan AI, Faruque ASG, Ryan ET. Severe, acute watery diarrhea in an adult. PLoSNeglTropl Dis. 2010 Nov 30:4(11).
5. **Chowdhury F,**Ashraful I. Khan, Jason B. Harris, Regina C. LaRocque, Edward T. Ryan, ASG Faruque, Stephen B.Calderwood, Firdausi Qadri. A comparison of Clinical and Immunologic Features in Children and Older Patients Hospitalized With Severe Cholera in Bangladesh.The Paediatric Infectious Disease Journal. 2008; 27(11): 986-992

BIOGRAPHICAL SKETCH

NAME: **DrAshraful Islam Khan**	POSITION TITLE: Scientist and Project Coordinator
eRA COMMONS USER NAME (credential, e.g., agency login)	

EDUCATION/TRAINING *(Begin with baccalaureate or other initial professional education, such as nursing, and include postdoctoral training.)*

INSTITUTION AND LOCATION	DEGREE *(if applicable)*	YEAR(s)	FIELD OF STUDY
SylhetMAGOsmaniMedicalCollege, Sylhet	MBBS	1994	Medicine, Surgery
University of Dhaka, Bangladesh	M.Sc	2001	Health economics
Uppsala University, Sweden	PhD	2012	International Health

A. Personal Statement

The goal of the proposed research is to identify cost efficient settings and strategies for use of oral cholera vaccine to control endemic disease and optimize health impact. My research has focused on oral cholera vaccine (OCV) including host-pathogen interactions in *V. cholerae* infection and identification of immunologic correlates of protection among household contacts of cholera patients in Bangladesh. I have been involved with Centre for Vaccine Sciences of the International Centre for Diarrhoeal Disease Research in Dhaka, Bangladesh (icddr,b) in pursuit of these works. In recent years, I have focused on studies of mass vaccination program "Introduction of cholera vaccine in Bangladesh (ICVB)" and '"Single dose oral cholera vaccine in Bangladesh (SCVB)" in Mirpur area under the supervision of Dr. Firdausi Qadri. I am co-Principal Investigator (with Dr. Firdausi Qadri) and also coordinate the project activities entitled "Enteric Disease Surveillance in Bangladesh" in 7 divisions of Bangladesh with the collaboration of IEDCR/GoB. Knowledge gained through these projects could critically improve our understanding of immunity during cholera, and could directly lead to an improved cholera vaccine or immunization strategy.

B. Positions and Honors

Employment and Professional Experience

- One year post graduate training on Paediatric Medicine in DhakaShishuHospital as honorary medical officer from July1995 to June 1996. Training period was full time and residential. Training is recognized by Bangladesh Medical and Dental Council (BMDC).
- In recognition of outstanding participation in and completion of an intensive orientation program on the NIH campus in Bethesda, Maryland, USA from July 5-22, 2005 for the Fogarty/Ellison Fellowship in Global Health and Clinical Research.
- Successfully completed a 'Actions in Building Capacity Training Fellowship' 2004 – 2005 funded by the Fogarty International Center, National Institute of Health, USA, between the Massachusetts General Hospital, Harvard Medical School, Boston, Massachusetts and The International Centre for Diarrhoeal Disease Research, Dhaka, Bangladesh
- Successful completion of the Fogarty/Ellison Fellowship in Clinical Research and Global Health for the year 2005 - 2006.
- Completed the 7 week post graduate course 'Introduction to Scientific Research' in 2007, Uppsala University, Sweden.

- Completed 2 weeks intensive course in the Summer School on Modern Methods in Biostatistics and Epidemiology in 2009, Italy

C. Selected Publications

1. Khan IA, Saha A, Chowdhury F, Khan AI, Uddin MJ, Begum YA, Riaz BK, Islam S, Ali M, Luby SP, Clemens JD, Cravioto A, Qadri F. Coverage and cost of a large oral cholera vaccination program in a high-risk cholera endemic urban population in Dhaka, Bangladesh. Vaccine. 2013 Dec 9;31(51):6058-64.
2. Sarker AR, Islam Z, Khan IA, Saha A, Chowdhury F, Khan AI, Qadri F, Khan JA. Cost of illness for cholera in a high risk urban area in Bangladesh: an analysis from household perspective. BMC Infect Dis. 2013 Nov 4;13:518.
3. Wahed T, Kaukab SS, Saha NC, Khan IA, Khanam F, Chowdhury F, Saha A, Khan AI, Siddik AU, Cravioto A, Qadri F, Uddin J. Knowledge of, attitudes toward, and preventive practices relating to cholera and oral cholera vaccine among urban high-risk groups: findings of a cross-sectional study in Dhaka, Bangladesh. BMC Public Health. 2013 Mar 19;13:242.
4. Saha A, Chowdhury MI, Khanam F, Bhuiyan MS, Chowdhury F, Khan AI, Khan IA, Clemens J, Ali M, Cravioto A, Qadri F. Safety and immunogenicity study of a killed bivalent (O1 and O139) whole-cell oral cholera vaccine Shanchol, in Bangladeshi adults and children as young as 1 year of age. Vaccine. 2011 Oct 26;29(46):8285-92.
5. Chowdhury F, Rahman MA, Begum YA, Khan AI, Faruque AS, Saha NC, Baby NI, Malek MA, Kumar AR, Svennerholm AM, Pietroni M, Cravioto A, Qadri F. Impact of rapid urbanization on the rates of infection by Vibrio cholerae O1 and enterotoxigenic Escherichia coli in Dhaka, Bangladesh.PLoSNegl Trop Dis. 2011 Apr 5;5(4):e999.
6. Uddin MJ, Wahed T, Saha NC, Kaukab SS, Khan IA, Khan AI, Saha A, Chowdhury F, Clemens JD, Qadri F. Coverage and acceptability of cholera vaccine among high-risk population of urban Dhaka, Bangladesh. Vaccine. 2014 Sep 29;32(43):5690-5.

D. Research Support (Ongoing)

1. Introduction of cholera vaccine in bangladesh: “impact evaluation of cholera vaccine and behaviour change interventions in urban dhaka” (research protocol number: pr-10061; funding gates foundation).
2. An individually randomized, placebo-controlled trial to measure the protection conferred by a single dose regimen of bivalent, killed, whole cell oral cholera vaccine (shanchol™) in dhaka, Bangladesh (research protocol number: pr-12090; funding gates foundation through ivi).
3. Enteric disease surveillance in bangladesh (research protocol number: pr-12060; funding gates foundation/fao, dghs).
4. Protective immunity to human cholera in bangladesh (research protocol number: 11041; funding nih).
5. Innate and adaptive immune response against oral cholera vaccine in bangladesh (research protocol number: pr-14086; funding nih).

Biography of K Zaman

1. **Name:** K Zaman

2. **Present Position:** Senior Scientist and Epidemiologist

3. **Educational background**: (last degree and diploma & training relevant to the present research proposal)

	Institution	Year
PhD	Johns Hopkins Bloomberg School of Public Health, USA	1999
MPH	Johns Hopkins Bloomberg School of Public Health, USA	1992
MBBS	Rajshahi Medical College, Bangladesh	1978

4. **Ethics Certification:**

		If Yes		
		Issuing Authority	Registration No	Valid Until
**No** ☐	Yes ☒	NIH	1712891	Taken on 02/28/2015

Note: If the response is "no", please get certification from CITI or NIH before study initiation and submit a copy to the Committee Coordination Secretariat

5. **List of ongoing research protocols/ activities**

Protocol/ Activity Number	Role in the protocol/ activity (PI, Co-PI, Co-I)	Starting date	End date	Percentage of time
PR 13097	PI	July 2014	July 2015	25
PR 14115	PI	Dec 2014	Dec 2015	25
PR 12004	PI	March 2012	March 2016	20
PR 15004	PI	March 2015	July 2016	30

6. **Publications**

Types of publications	Numbers
a. Original scientific papers in peer-review journals	140
b. Peer reviewed articles and book chapters	3
c. Papers in conference proceedings	115
d. Letters, editorials, annotations, and abstracts in peer-reviewed journals	4
e. Working papers	
f. Monographs	

7. **Five recent publications including publications relevant to the present research protocol**

1. **Zaman K**, Naser AM. Power M, Yaich M, Zhang L, Ginsburg AS, Luby SP, Rahman M, Hills S, Bhardwaj M, Jorge Flores J. Lot -to-lot consistency of live attenuated SA 14-14-2 Japanese encephalitis vaccine manufactured in a Good Manufacturing Practice facility and non-inferiority with respect to an earlier product. **Vaccine 2014; 32: 6061-66.**

2. **Zaman K**, Yunus M, Arifeen SE, Azim T, Faruque ASG, Huq E, Hossain I, Luby SP, Victor JC, Dallas MJ, Lewis K, Rivers SB, Steele AD, Neuzil K, Ciarlet M, Sack DA. Methodology and lessons-learned from the efficacy clinical trial of the pentavalent rotavirus vaccine in Bangladesh. **Vaccine 2012; 305: A94-A100.**

3. **Zaman K**, Anh DD, Victor JC, Shin S, Yunus M, Dallas MJ, Podder G, Thiem VD, Mai LP, Luby SP, Coia ML, Lewis K, Rivers SJ, Sack DA, Clemens JD, Scodel F, Steele AD, Neuzil KM, Ciarlet M. Efficacy of the pentavalent rotavirus vaccine against sever rotavirus gastroenteritis among infants in developing countries in Asia: A randomized, double-blind placebo-controlled trial. **Lancet 2010 Aug 21;376(9741): 615-23. Epub 2010 Aug 6.**

4. **Zaman K**, Sack DA, Yunus M, Arifeen SE, Podder G, Azim T, Luby S, Breiman RF, Neuzil K, Datta SK, Delem A, Suryakiran PV, Bock HL; the Bangladeshi Rotavirus Vaccine study group. Successful co-administration of a human rotavirus and oral poliovirus vaccines in Bangladeshi infants in a 2-dose schedule at 12 and 16 weeks of age. **Vaccine 2009; 27: 1333-1339**.

5. **Zaman K**, Roy E, Arifeen SE, Rahman, M, Raqib R, Wilson E, Omer SB, Shahid N, Breiman RF, Steinhoff MC. Maternal immunization with influenza vaccine protects both mothers and infants. **N Eng J Med 2008; 15: 1555-1564**

Biography of Dr Niyaz Ahmed

Prof. Dr. Niyaz Ahmed *PhD, FNASc, FRSC*

SENIOR DIRECTOR, INTERNATIONAL CENTRE FOR DIARRHOEAL DISEASE RESEARCH BANGLADESH (ICDDR,B), DHAKA, BANGLADESH

Other appointments:

Professor, Department of Biotechnology & Bioinformatics, University of Hyderabad, India
Adjunct Professor, Academy of Scientific and Innovative Research, India
Academic Icon Professor, University of Malaya, Malaysia
Editor in Chief, *Gut Pathogens* (London)

Date of birth: December 25, 1971

Citizenship and Nationality: India

Gender: Male

Marital status: Married; 3 children

Address and contact details:

Laboratory Sciences and Services Division,
icddr,b, Mohakhali
Dhaka 1212, Bangladesh

Dhaka (headquarters):
+880 (0)2-982-7063 (direct) Fixed Line Office
+880 1708132499 Mobile phone
niyaz.ahmed@icddrb.org
Web: http://www.icddrb.org

Other contact details:
+91 40 67768571 (direct)
+91 9908 202786 Mobile phone
ahmed.nizi@gmail.com / niyaz.ahmed@uohyd.ac.in
Skype: niyazSL
Web: http://www.niyazahmed.org

Education and Training

Degree Program	Completion	Awarding/training institution
Doctor of Veterinary Medicine	1995	Nagpur Veterinary College, India
Master of Science (Infectious/Zoonotic Diseases)	1997	National Dairy Research Institute, Karnal, India (Sunita Grover)
Overseas Training Fellowships (ICMR – INSERM)	2005	Institut Pasteur de Lille (Philip Supply)
Doctor of Philosophy (Infectious Diseases)	2007	Centre for DNA Fingerprinting and Diagnostics (CDFD), Hyderabad, India (S Hasnain)
Post doctoral visiting fellowship	2008	University of Sassari, Italy (Leonardo Sechi)

Employment history and academic positions

Position	Duration	Institution
Staff Scientist and Group Leader	1998-2008	CDFD, Hyderabad, India
Associate Professor	2008-2014	University of Hyderabad, Hyderabad, India
Professor and Chair of the Department	2014- November 2016	University of Hyderabad, Hyderabad, India
Head of Pathogen Biology Laboratory	2008-till date	University of Hyderabad, Hyderabad, India
Senior Director	November 2016-till date	icddr,b; Dhaka, Bangladesh

Statement of scientific leadership, contributions and peer recognition

Dr. Niyaz Ahmed and his research group contributed precious state of art in the area of genomics, epidemiology and disease biology of major human and veterinary pathogens. Ahmed's work unleashed novel pathogenic mechanisms and survival strategies of bacterial pathogens while elevating the standards of microbial research in India, a country battling and challenged with infection burden. Dr Ahmed has supervised 14 PhD students and a dozen post-doctoral scientists were trained in his laboratories. His PhD students and post doctoral colleagues comprise the research team working on 'in country epidemiology' in India and Bangladesh using genomics and Microbiota studies in the context of enteric infections. Dr Ahmed's work embodied in about 140 international publications remains highly cited and recognized. Dr Ahmed sits on editorial boards of reputed journals (Microbial Genomics, PLOSONE, Infectious Agents and Cancer, Scientific Reports, Systems and Synthetic Biology) and edits *Gut Pathogens* as Chief Editor since 2008. Dr Ahmed is invited to different grant and advisory committees, in particular at the WHO, the European Commission and the Skolkovo Foundation of the Russian Federation. He has been awarded with all important science awards in India including the prestigious 'Shanti Swarup Bhatnagar Prize' (2016). In addition, he has been awarded the 'Microsoft Azure for Research in Public Health Award' by Microsoft Corporation. Further, Dr Ahmed was elected to the fellowships of the National Academy of Sciences of India as well as the Royal Society of Chemistry (UK) for his pioneering work in the area of antimicrobial resistance mechanisms and surveillance in India.

List of 15 important publications:

1. Shaik S, Ranjan A, Tiwari SK, Hussain A, Nandanwar N, Kumar N, Jadhav S, Semmler T, Baddam R, Islam MA, Alam M, Wieler LH, Watanabe H, **Ahmed N**. Comparative genomic analysis of globally dominant ST131 clone with other epidemiologically successful extraintestinal pathogenic Escherichia coli (ExPEC) lineages. **mBio. 2017,** 8:e01596-17.
2. Ranjan A, Shaik S, Nandanwar N, Hussain A, Tiwari SK, Semmler T, Jadhav S, Wieler LH, Alam M, Colwell RR, **Ahmed N**. Comparative genomics of *Escherichia coli* isolated from skin and soft tissue and other extraintestinal infections. **mBio.** 2017, 8: e01070-17.
3. Doddam SN, Peddireddy V, **Ahmed N**. Mycobacterium tuberculosis DosR Regulon Gene Rv2004c Encodes a Novel Antigen with Pro-inflammatory Functions and Potential Diagnostic Application for Detection of Latent Tuberculosis. **Front Immunol**. 2017, 8:712.
4. Qumar S, Majid M, Kumar N, Tiwari SK, Semmler T, Devi S, Baddam R, Hussain A, Shaik S, **Ahmed N**. Genome Dynamics and Molecular Infection Epidemiology of Multidrug-Resistant Helicobacter pullorum Isolates Obtained from Broiler and Free-Range Chickens in India. **Appl Environ Microbiol**. 2017, 83:e02305-16.
5. Devi S, Ansari SA, Tenguria S, Kumar N, **Ahmed N**. Multipronged regulatory functions of a novel endonuclease, TieA, from *Helicobacter pylori*. **Nucleic Acids Res**. 2016, 44:9393-9412
6. Ranjan A, Shaik S, Mondal A, Nandanwar N, Hussain A, Semmler T, Kumar N, Tiwari S, Jadhav S, Wieler LH, **Ahmed N**. Molecular epidemiology and genome dynamics of New Delhi metallo-beta-lactamase (NDM) producing extraintestinal pathogenic E. coli (ExPEC) strains from India. **Antimicrob Agents Chemother**. 2016, 60: 6795-6805.
7. Ranjan A, Shaik S, Hussain A, Nandanwar N, Semmler T, Jadhav S, Wieler LH, **Ahmed N**. Genomic and Functional Portrait of a Highly Virulent, CTX-M-15-Producing H30-Rx Subclone of Escherichia coli Sequence Type 131. **Antimicrob Agents Chemother**. 2015, 59:6087-95.
8. Kumar N, Mariappan V, Baddam R, Lankapalli AK, Shaik S, Goh KL, Loke MF,Perkins T, Benghezal M, Hasnain SE, Vadivelu J, Marshall BJ, **Ahmed N**. Comparative genomic analysis of *Helicobacter pylori* from Malaysia identifies three distinct lineages suggestive of differential evolution. **Nucleic Acids Res.** 2015, 43: 324-35.
9. Baddam R, Kumar N, Shaik S, Lankapalli AK and **Ahmed N**. Genome dynamics and evolution of *Salmonella* Typhi strains from the typhoid-endemic zones. **Sci Rep.** 2014, 4:7457.
10. Hussain A, Ranjan A, Nandanwar N, Babbar A, Jadhav S, **Ahmed N**. Genotypic andPhenotypic Profiles of *Escherichia coli* Isolates Belonging to Clinical SequenceType 131 (ST131), Clinical Non-ST131, and Fecal Non-ST131 Lineages from India. **Antimicrob Agents Chemother.** 2014, 58:7240-9.
11. Rani PS, Babajan B, Tulsian NK, Begum M, Kumar A, **Ahmed N**. Mycobacterial Hsp65 potentially cross-reacts with autoantibodies of diabetes sera and also induces (in vitro) cytokine responses relevant to diabetes mellitus. **Mol Biosyst**. 2013, 9:2932-41.
12. Hussain A, Ewers C, Nandanwar N, Guenther S, Jadhav S, Wieler LH, **Ahmed N**. Multiresistant uropathogenic Escherichia coli from a region in India where urinary tract infections are endemic: genotypic and phenotypic characteristics of sequence type 131 isolates of the CTX-M-15 extended-spectrum-β-lactamase-producing lineage. **Antimicrob Agents Chemother**. 2012, 56:6358-65.
13. **Ahmed N**, Dobrindt U, Hacker J, Hasnain SE. Genomic fluidity and pathogenic bacteria: applications in diagnostics, epidemiology and intervention. **Nat Rev Microbiol**. 2008, 6:387-94.
14. Rizwan M, Alvi A, **Ahmed N.** Novel protein antigen (JHP940) from the genomic plasticity region of Helicobacter pylori induces tumor necrosis factor alpha and interleukin-8 secretion by human macrophages**. J Bacteriol**. 2008, 90:1146-51.
15. Alvi A, Devi SM, Ahmed I, Hussain MA, Rizwan M, Lamouliatte H, Mégraud F, **Ahmed N**. Microevolution of Helicobacter pylori type IV secretion systems in an ulcer disease patient over a ten-year period. **J Clin Microbiol**. 2007, 45:4039-43.

2

Biography of Shafiqul A. Sarker

Present Position: Emeritus Scientist

Educational background: (last degree and diploma& training relevant to the present research proposal)

	Institution	Year
Degree	MBBS (Rajshahi University, Bangladesh)	1977
Degree	MD (University of Basel, Switzerland)	1991
Degree	PhD (Karolinska Institute, Sweden)	2006

Ethics Certification:

		If Yes		
		Issuing Authority	Registration No	
**No** ☐	Yes ☒	NIH	1484127	

Note: If the response is “no”, please get certification from CITI or NIH before study initiation and submit a copy to the Committee Coordination Secretariat

List of ongoing research protocols/ activities

Protocol/ Activity Number	Role in the protocol/ activity (PI, Co-PI, Co-I)	Starting date	End date	Percentage of time
PR- 12051	PI	01.05.2012	01.10.2017	35%
PR-14038	PI	24.09.2014	31.01.2018	10%
PR-15089	PI	08.06.2016	07.12.2018	40%
PR- 16007	Co-I	13.03.2016	31.10.2019	15%

Publications

Types of publications	Numbers
g. Original scientific papers in peer-review journals	80
h. Peer reviewed articles and book chapters	4
i. Papers in conference proceedings	1
j. Letters, editorials, annotations, and abstracts in peer-reviewed journals	40
k. Working papers	
l. Monographs	

Five recent publications including publications relevant to the present research protocol

1. **Sarker SA**, McCallin S, Barretto C, Berger B, Piitet A, Sultana S, Krauser L, Bruttin A, Brussow H. Oral T4-Like Phage Cocktail Application To Healthy Adult Volunteers from Bangladesh "Virology 434 (2012) 222–232".

2. Sarker SA, Jäkel M, Sultana S, Alam NH, Bardhan PK, Chisti MJ, Salam MA, Theis W, Hammarström L, Frenken L. Anti-rotavirus protein reduces stool output in infants with diarrhea: a randomized placebo-controlled trial. Gastroenterology 2013 Oct; 145 (4):740-748.

3. Gilles Bourdin, Armando Navarro, **Shafiqul A. Sarker**, Anne-C. Pittet, Firdausi Qadri, Shamima Sultana, Alejandro Cravioto, Kaisar A. Talukder, Gloria Reuteler and Harald Brüssow. Coverage of diarrhoea-associated *Escherichia coli* isolates from different origins with two types of phage cocktails *Microbial Biotechnology* (2014) **7**(2), 165–176 doi:10.1111/1751-7915.12113

4. **Shafiqul Alam Sarker**, Shamima Sultana, Mark Pietroni and Arthur Dover. Safety of a Bioactive Polyphenol Dietary Supplement in Pediatric Subjects with Acute Diarrhoea. Int. J. Pediatrics Volume 2015, Article ID 387159, 10 pages http://dx.doi.org/10.1155/2015/387159

5. Shafiqul Alam Sarker, Shamima Sultana, Gloria Reuteler, Deborah Moine, Patrick Descombes,[c] Florence Charton, Gilles Bourdin, Shawna McCallin and Harald Brüssow. Oral Phage Therapy of Acute Bacterial Diarrhea With Two Coliphage Preparations: A Randomized Trial in Children From Bangladesh. EBioMedicine. 2016 Feb; 4: 124–137.

Biography of Andrew J Pollard

CURRICULUM VITAE

NAME	*POSITION TITLE*
Andrew John Pollard	***Professor of Paediatric Infection & Immunity***, Director of the Oxford Vaccine Centre and Honorary Consultant Paediatrician, University of Oxford, UK

EDUCATION/TRAINING

INSTITUTION AND LOCATION	DEGREE	YEAR(s)	FIELD OF STUDY
University of London	BSc	1986	Experimental Pathology
University of London	MBBS	1989	Medicine
Royal College of Physicians	MRCP (UK)	1993	Paediatrics
Royal College of Paediatrics and Child Health	FRCPCH	1996	Paediatrics
University of London	PhD	1999	Meningococcal immunity
Imperial College of Science Technology and Medicine	DIC	1999	Infectious Disease

Current Positions:
Professor of Paediatric Infection and Immunity, Department of Paediatrics, University of Oxford
Honorary Consultant Paediatrician, Children's Hospital, Oxford;
Director of the Oxford Vaccine Group University of Oxford, University of Oxford
Clinical co-lead of the Oxford AHSN Children's Network
Children's Lead for the NIHR Thames Valley Clinical Research Network
Co-lead for the NIHR Oxford Biomedical Research Centre vaccines theme
Course Director, Oxford University Postgraduate Diploma and MSc in Paediatric Infectious Disease.

Honorary Positions Held:
Chair of the Joint Committee on Vaccination and Immunisation, UK Dept. of Health (2013-)
Chair of the Scientific Advisory Committee on Vaccines at the European Medicines Agency (2012-)
Chair of the review of NIBSC bacteriology Division 2015
Chair of the scientific committee for the World Society for Paediatric Infectious Disease (2009- 2013)
Member of the Commission on Safety of Medicines CTBVEAG
Member of the pneumococcal, HPV, meningococcal and varicella vaccine subcommittees of the Joint Committee on Vaccination and Immunisation, Dept. of Health
Member of the DH Ebola SAGE.
Member of the NERVTAG
Chairman of the NICE topic expert group on meningitis in Children (2011-2013)
Chairman of the NICE guidelines development group for meningitis in children (2006-2010)

Previous positions: *2006-2008, Reader in Paediatric Infection and Immunity, University of Oxford; 2001-2006 University lecturer in Paediatric Infectious Disease, University of Oxford; 2000-2001 Pediatric Infectious Disease Society (USA) fellow in Pediatric Infectious Diseases, University of British Columbia and British Columbia's Children's Hospital, Vancouver, Canada; 1999-2000 Clinical Fellow, British Columbia Children's Hospital, Vancouver, Canada; 1998-1999 Specialist Registrar in Paediatric Intensive Care St Mary's Hospital, London W2; 1995-1998 Action Research Training Fellow Department of Paediatrics, St Mary's Hospital Medical School, London, Supervised by, Professor Michael Levin; 1995 Registrar in Paediatric Infectious Diseases and Intensive Care, in the Department of Paediatrics, St Mary's Hospital Medical School, London W2; Registrar in Paediatric Hepatology, Birmingham Children's Hospital, Birmingham UK; 1991-1994 Senior House Officer in Paediatrics at Birmingham Children's Hospital, Birmingham Maternity Hospital, and Dudley Road Hospital, Birmingham; 1990-1991 Casualty Officer, Whittington Hospital, London; 1989-1990 House Officer Whipps Cross Hospital and St. Bartholomew's Hospital, London.*

Honors: Wheelwright's Prize in Paediatrics, St Bartholomew's and The Royal London Hospitals (1988); Honours Colours, St Bartholomew's Hospital Student's Union (1989); 1st Prize in the Canadian Pediatric Fellows research competition, Winnipeg, Canada (1999); Subspeciality resident award for excellence in teaching Pediatric residents, British Columbia's (2000); Alberta Heritage lecturer, Calgary, Alberta, Canada (2001); BC Research Institute for Children's & Women's Health award for Outstanding Achievement by a Clinical Subspeciality Fellow (2001); "Science, honor and truth" award of the 32nd anniversary of the High Altitude Pathology Institute (Clinica IPPA * Instituto de Patologia en la Altura) in La Paz, Bolivia (2002). Bill Marshall Award of the European Society for Paediatric Infectious Disease (2013)

Training:
NIH Human Subjects Research training course certificate number 461501 (9/6/10)
GCP training, University of Oxford 02/08/13
OUH Life Support certificate January 2013/ December 2014

Graduate supervision

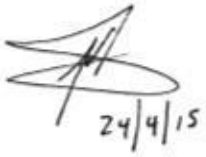

Katherine Theiss-Nyland

66 Oxford Gardens • London, UK • Phone: +44 7539 500027 • Email: katherinetn@gmail.com • Skype: katherinetn

---

DEGREES

- **PhD, Infectious Disease Epidemiology, 2017** London School of Hygiene and Tropical
- **MPH, Epidemiology/Biostatistics, Global Health, 2010,** University of California, Berkeley
- **Bachelor's Degree, Microbiology/Sociology, 2006,** University of British Columbia

RELEVANT WORK EXPERIENCE AND FELLOWSHIPS

TyVAC Programme Coordinator – University of Oxford, *2017-present*
- Coordinate randomized control trials of typhoid conjugate vaccine in Nepal and Bagladesh, as part of a research consortium for typhoid vaccine acceleration
- Manage the broader University of Oxford deliverables and sub-contracts for TyVAC, including health economics studies, typhoid burden of disease estimates and country preparedness work
- Provide epidemiological expertise and support to the Oxford Vaccine Group overseas research projects on typhoid and pneumonia

Consultant – NetWorks, USAID, Presidents Malaria Initiative, *2014-2015*
- Designed research tools and a project proposal for the evaluation of the continuous distribution (CD) of LLINs
- Conducted a qualitative assessment in 4 countries on the barriers and bottle necks to CD of LLINs
- Provided feedback to governments and a report of findings for USAID and stakeholders, identifying areas for improvement and key recommendations

Research Fellow - Partnership for Maternal, Neonatal, and Child Health; IDEAS, London School of Hygiene and Tropical Medicine, *2012-2014*
- Researched key topics within Maternal and Child Health, and synthesized current knowledge and practices into policy briefs, translating scientific findings into actionable policy and practice guidelines
- Liaised with key subject experts, globally, and integrated identified messaging and case-studies into knowledge summaries for diverse audiences
- Conducted web-seminars for global partners and implementers on key subjects within knowledge summaries

Deputy Program Director, Vaccines - Clinton Health Access Initiative, Addis Ababa, Ethiopia, *2011-2012*
- Supported the Federal Ministry of Health (FMOH) to accelerate the introduction of two new vaccines nationwide: Pneumococcal (PCV) and Rotavirus
- Managed the Monitoring and Evaluation of vaccine introduction including the development and implementation of the PCV uptake assessment for backlog cohort identification and schedule adherence
- Managed regional supportive supervision team to ensure proper vaccine handling, cold storage, and delivery to patients within 7 major regions of Ethiopia, and provide policy and implementation guidance to FMOH based on the regional findings
- Ensured adequate training and supply was allocated to regional and facility staff to effectively provide vaccination to all eligible infants

Program Manager, Laboratory Services - Clinton Health Access Initiative, Addis Ababa, Ethiopia, *2010-2012*
- Assisted the Ethiopian Health and Nutrition Research Institute (EHNRI), MOH, to operationalize the "National Master Plan" for laboratory service capacity building, including laboratory quality improvement and accreditation
- Provided technical assistance and training to EHNRI in areas of epidemiology. Specifically: disease surveillance, epidemiology research, and systematic review and meta-analysis
- Managed the national Early Infant Diagnostics (EID) program for HIV including, monitoring, forecasting, procuring, distribution, and capacity building within the government
- Implemented an evaluation of new technologies for diagnostics and mHealth, to improve point-of-care services and turn-around-time (TAT).

Malaria and Maternal Mortality Temporary Advisor - World Health Organization HQ, Geneva, Switzerland, *2009-2010*

Biographical Sketch

**Saha, Samir Kumar**	**Position Title**<br>Professor of Microbiology; Head, Diagnostic Division; Bangladesh Institute of Child Health, Dhaka Shishu Hospital
	Executive Director, Child Health Research Foundation(CHRF)

A. Personal Statement

Throughout my career, my passion has been working with limited resources and gradually building capacities. Starting with almost no microbiological facilities, I have developed a state-of-the-art setup, and formed a multi-disciplinary research team of more than 250 members. In 2007, I founded Child Health Research Foundation (CHRF), a research initiative in collaboration with the department of Microbiology, Dhaka Shishu Hospital, Bangladesh. The core mission of CHRF is to improve child health in Bangladesh and the regions, by facilitating appropriate policy decisions on treatment and preventions through research and advocacy. My team has led and leads multiple projects including the large multi-country community based project on Aetiology of Neonatal Infection in South Asia (ANISA). My research project also includes clinical trials, surveillances for invasive bacterial diseases at hospitals and population based sites, and measurement of impact of vaccines. Of note, we led multiple clinical trials on short course antibiotic therapies to rationalize the use of antibiotic for pneumonia and meningitis. Under my leadership, the team generated evidence and facilitated the decision making process of introducing Hib and pneumococcal vaccines in Bangladesh. As the follow up of vaccine introduction, we are now working to measure the vaccine impact to generate further evidence for policy-makers. All in all, over the last two decades, our setting has become a sustainable model for conducting high-quality research on infectious diseases in low-resource settings.

B. Positions and Honors

Employment

Current positions

2009-Present Head, Diagnostic Division, Dhaka Shishu Hospital, Dhaka, Bangladesh

2007-Present Executive Director, Child Health Research Foundation (CHRF), Dhaka, Bangladesh

2000-Present Professor and Senior Consultant of Microbiology, Dhaka Shishu Hospital, Dhaka, Bangladesh

2012-Present Adjunct Scientist, icddr,b, Bangladesh

Past positions

1995(Apr-Jun) Visiting Professor, Institute of Tropical Medicine, Nagasaki University, Nagasaki, Japan

1994-2000 Associate Professor and Consultant of Microbiology, Dhaka Shishu Hospital, Bangladesh

1990-1993 Assistant Professor and Junior Consultant of Microbiology, Dhaka Shishu Hospital, Bangladesh

Selected National, International and Professional Activities/Affiliations

2017 Member, Scientific Advisory Group member, Vaccines against group B streptococcal disease (GBS VP) project, WHO.
2017 Member, Technical Advisory Group of respiratory syncytial virus (RSV) Vaccine, Department of Immunization, Vaccines and Biologicals, WHO.
2016 Chair, Steering Committee of Coalition against Typhoid (CaT), Sabin Vaccine Institute, Washington.
2013 Member, Laboratory Technical Working Group, VP-IBD Surveillance, WHO.
2012 Member, Technical advisory group member, Vaccine Preventable - Invasive Bacterial Disease (VP- IBD) Surveillance, WHO.
2010 Member, International Scientific Committee of International Society for Infectious Diseases (ISID).
2007 Member, Technical Advisory Board of Immunization, Bangladesh.
2007 Member, National Committee for Immunization Practice, Bangladesh.
2006 Member, Global Pneumococcal Awareness Council of Experts (PACE).
2001 Associate, Department of International Health, Johns Hopkins University, USA

Award/honors

2018 Fellowship in the American Academy of Microbiology.
2017 UNESCO Carlos Finlay Prize for research in Microbiology.
2017 American Society of Microbiology (ASM) award for research in Clinical Microbiology.

C. Contribution to Science

My contribution to science is mainly focused on pediatric infectious diseases like pneumonia, meningitis and enteric fever in order to find the true burden of these diseases.

1. Haemophilus influenzae type b (Hib): One of my primary interests in the field of pneumonia and meningitis Was (Hib). We generate evidence on the burden of Hib diseases and the evidence led to the introduction of Hib vaccine in Bangladesh in 2009. In its aftermath, we measured the Hib vaccine impact and communicated our results to the relevant stake-holders.
2. Invasive Pneumococcal Diseases (IPD): While working on Hib diseases, I started working on IPD to generate evidence on its burden. This led to the introduction of pneumococcal conjugate vaccine (PCV) in Bangladesh (March 2015). At the same time, our data is being used as the baseline to measure the impact of PCV-10.
3. Enteric Fever: Enteric Fever, typhoid and paratyphoid, is endemic and the most common cause of invasive diseases in South Asia. We worked to discern the burden of typhoid among the children, and the dynamics of antimicrobial resistance of this organism in Bangladesh.
4. Different Surveillance Modalities: Surveillance is an important component to measure disease burden. I have

investigated different surveillance modalities with Prof. Steve Luby and designed an alternate model of
population-based study, community adjusted hospital-based surveillance, to overcome the limitations of it. This
model does not involve any intervention in the community, except collection of data (once in a year), on care
seeking at the specific hospital(s). This model is currently being used to measure disease burden and impact of
vaccine.

5. Diagnostics tools on detecting etiology: Effective surveillance of infectious disease is dependent on the
availability and appropriate use of diagnostics.
I have led my team to customize diagnostics and rationally use.
We aimed to address challenges including limitation of resources (e.g serotyping by algorithmic use of
Quellung and PCR), prior antibiotic therapy etc.

6. Group B Streptococcus (GBS): GBS infection is a leading cause of death in newborns in developed countries
and some of the low-income countries in Africa. The preliminary data suggest that GBS disease is rare among
the population in South Asia. To understand this better we are exploring the GBS colonization on newborns'
body surface and its relation with mortality, ii) prevalence of recto-vaginal carriage among the mothers, iii)
transmission of GBS to the babies body surface and iv) distribution of GBS serotypes in Bangladesh.

7. I am also keen to translate our scientific findings to public policies.

D. Research Support

Name of Project	Funded by	Role
Evaluation of 10-valent pneumococcal conjugate vaccine (PCV-10) impact on invasive pneumococcal diseases in Bangladesh (Sentinel sites)	Bill & Melinda Gates Foundation	PI
Impact of PCV-10 on IPD in rural Bangladesh (Mirzapur)	GlaxoSmithKline	PI
Evaluation of 10-valent pneumococcal conjugate vaccine (PCV-10) impact on carriage in Bangladesh children	Institute for Health Metrics and Evaluation (GAVI)	PI
Pneumococcal Vaccine Impact Assessment in Bangladesh (Sylhet)	Johns Hopkins University (BMGF)	CoPI
Aetiology of neonatal infection in South Asia (ANISA)	Bill & Melinda Gates Foundation	PI
Typhoid Diagnostics for Measuring Disease Burden	Fondation Mérieux, France	PI
Vaccination and the Paediatric Microbiome	University of Washington (BMGF)	PI
Invasive Bacterial Disease surveillance	World Health Organization	PI
Maternal Genitourinary Tract infection and adverse prenatal outcomes	Johns Hopkins University (NIH)	Co-PI
Surveillance for Enteric fever in Asia Project	Sabin Vaccine Institute, Inc (BMGF)	PI
Burden of antibiotic Resistance in Neonates from Developing Societies (BARNARDS)	Cardiff University (BMGF)	PI
Group B Streptococcus colonization in mother-newborn dyads and association with anti-capsular serotype-specific antibodies in low and middle income South Asian and African countries	Respiratory & Meningeal Pathogens Research Unit (BMGF)	PI
Multi-centre Evaluation of the Burden of Invasive	Novartis Vaccines & Diagnostics	PI

Neonatal Group B Streptococcal Disease: Incidence and Serotype Distribution		

E. Paper Published (Selected)

1. Sultana NK, **Saha SK**, Al-Emran HM, et al. Impact of introduction of the *Haemophilus influenzae* type b conjugate vaccine into childhood immunization on meningitis in Bangladeshi infants. J Pediatr. 2013; 163 (1 Suppl):S73-8
2. Baqui AH, El Arifeen S, **Saha SK**, et al. Effectiveness of Haemophilus influenzae type b conjugate vaccine on prevention of pneumonia and meningitis in Bangladeshi children: a case-control study. Pediatr Infect Dis J. 2007; 26: 565-71.
3. **Saha SK**, Baqui AH, Darmstadt GL, Amin RM et al. Invasive Haemophilus influenzae type b diseases in Bangladesh, with increased resistance to antibiotics. J Pediatr 2005; 146:227-33.
4. **Saha SK,** Rikitomi N, Ruhulamin M, et al. The increasing burden of disease in Bangladeshi children due to *H. influenzae* type b meningitis. Annals of Trop Paediatr. 1997; 17:5-8.
5. **Saha SK**, Hossain B, Islam M, et al. Epidemiology of invasive pneumococcal disease in Bangladeshi children prior to introduction of pneumococcal conjugate vaccine. Pediatric Infect Dis J. 2016; 35:655-61
6. **Saha SK**, Naheed A, Arifeen SE, et al. Surveillance for invasive *Streptococcus pneumoniae* among children in Bangladesh: Antimicrobial susceptibility and serotype distribution. Clin Infect Dis. 2009; 48.Suppl 2:S75-81.
7. Arifeen SE, **Saha SK**, Rahman S, et al. Invasive Pneumococcal Disease Among Children in Rural Bangladesh: Results from a Population-Based Surveillance. Clin Infect Dis. 2009; 48.Suppl 2:S103-13.
8. **Saha SK**, Rikitomi N, Biswas D, et al. Serotypes of *Streptococcus pneumoniae* causing invasive childhood infections in Bangladesh, 1992 to 1995. J Clin Microbiol. 1997; 35:785-787.
9. Luby SP, **Saha SK** and Andrews JR. Towards sustainable public health surveillance for enteric fever. Vaccine 2015; 33 (Suppl):C3-C7.
10. **Saha SK**, Darmstadt GL, Baqui AH, Crook DW, Islam N, Islam M, Hossain M, Arifeen SE, Santosham M, E Black RE. Highly Ciprofloxacin resistant Salmonella enterica serovar Typhi in Bangladesh: molecular basis of resistance. J Clin Microbiol, 2006; 44:3811-3813.
11. **Saha SK**, Baqui AH, Hanif M, Darmstadt GL, Ruhulamin M, Nagatake T, Santosham M, Black RE. Typhoid fever in Bangladesh: implications for vaccination policy. Pediatr Infect Dis J. 2001;20(5):521-4.
12. **Saha SK**, Saha S, Ruhulamin M, Hanif M, Islam M. Decreasing trend of multiresistant Salmonella typhi in Bangladesh. J Antimicrob Chemother. 1997;39(4):55-6.
13. Sultana NK, **Saha SK**, Al-Emran HM et al. Impact of introduction of the *Haemophilus influenzae* type b conjugate vaccine into childhood immunization on meningitis in Bangladeshi infants. J Pediatr. 2013 Jul; 163(1 Suppl):S73-8.
14. Luby SP, Halder AK, **Saha SK,** et al. A low-cost approach to measure the burden of vaccine preventable diseases in urban areas. Vaccine. 2010;28 (31):4903-12.
15. Saha S, Modak JK, Naziat H, Al-Emran HM, Chowdury M, Islam M, Hossain B, Darmstadt GL, Whitney CG, **Saha SK**. Detection of co-colonization with *Streptococcus pneumoniae* by algorithmic use of conventional and molecular methods. Vaccine. 2015; 33:713-8.
16. **Saha SK**, Darmstadt GL, Baqui AH, et al. Direct detection of the multidrug resistance genome of *Haemophilus influenzae* in cerebrospinal fluid of children: implications for treatment of meningitis. Pediatr Infect Dis J.2008; 27: 49-53.
17. **Saha SK,** Darmstadt GL, Baqui AH, et al. Identification of capsular serotype in culture negative pneumococcal meningitis using sequential multiplex PCR: implications for surveillance and vaccine design. PLoS One. 2008; 3(10):e3576. Epub 2008 Oct 31.
18. **Saha SK,** Darmstadt GL, Yamanaka N, et al. Rapid Diagnosis of Pneumococcal Meningitis: Implications for Treatment and Measuring Disease Burden. Pediatr Infect Dis J 2005; 24: 1093–1098.

19. Russell NJ, Seale AC, O'Driscoll M, O'Sullivan C, Bianchi-Jassir F, Gonzalez-Guarin J, Lawn JE, Baker CJ, Bartlett L, Cutland C, Gravett MG, Heath PT, Le Doare K, Madhi SA, Rubens CE Schrag S, Sobanjo-Ter Meulen A, Vekemans J, **Saha SK**, Ip M; GBS Maternal Colonization Investigator Group. Maternal Colonization With Group B Streptococcus and Serotype Distribution Worldwide: Systematic Review and Meta-analyses. Clin Infect Dis. 2017; 6;65 (suppl_2):S100-S111.
20. **Saha SK**, Ahmed ZB, Modak JK, Naziat H, Saha S, Uddin MA, Islam M, Baqui AH, Darmstadt GL, Schrag SJ. Group B Streptococcus among pregnant women and newborns in Mirzapur, Bangladesh: Colonization, vertical transmission and serotype distribution. J Clin Microbiol. 2017.
21. Islam MS, Saha SK, Islam M, Modak JK, Shah R, Talukder RR, El Arifeen S, Baqui AH, Darmstadt GL, Mullany LC. Prevalence, Serotype Distribution and Mortality Risk Associated With Group B Streptococcus Colonization of Newborns in Rural Bangladesh. Pediatr Infect Dis J. 2016;35(12):1309-1312.
22. Pisanic N, Rahman A, **Saha SK**, et al. Development of an oral fluid immunoassay to assess past and recent hepatitis E virus (HEV) infection. J Immunol Methods. 2017 Sep; 448:1-8. doi: 10.1016/j.jim.2017.04.012. Epub 2017 May 3.

Government of the People's Republic of Bangladesh
Ministry of Health & Family Welfare
Directorate General of Health Services

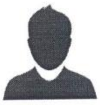

HRIS Bio-Data (Full)

Name: Dr. Md. Abdullahel Kafee
Bangla Name: ডাঃ মোঃ আব্দুল্লাহেল কাফী
Designation: OSD, Type of post: Cadre, Post Pay Scale: 6
Place of Posting: Directorate General of Health Services (DGHS)
Agency Name: DGHS

Status: Posted
Posted as: Attached
HRIS ID: 108572
Code No(Doctors Only): 110088
BCS Batch No: 21

Original Designation:
Asstt. Professor (Medicine)

Additional Designation(s):
Asstt. Professor(Medicine) , Attached,
Kurmitola 500 Bed General Hospital

ACR Availability
2010, 2011, 2012, 2013, 2014, 2015

Personal Information

Father's Name: Md.Abdur Rahman
Mother's Name: Umme Kulsum
Date Of Birth: 31-12-1970
Sex: Male
Marital Status: Married
Religion: Islam
Mobile No.: 01711192140
NID: 2619351180559
TIN:
Email Address: abdullahelkafee@Yahoo.com

**Present Address**	**Permanent Address**
**Detail Address:** 18/B,Dolipara,jasimuddin Avenue,uttara,Dhaka	**Detail Address:** Vill-char Haripur,Po-Bhatara Upozela-Sarisha Bari ,jamalpur
**Upazila:**	**Upazila:**
**District:**	**District:**
**Division:**	**Division:**

Other Information

**Prof. Discipline:** Medicine	**Tribal:** Not Tribal	**Joining Date (Govt. health service):** 21-05-2003
**Staff Professional Category:** Physician	**Freedom Fighter:** No	**Joining Date (Current place):** 29-12-2016
**Job Status:** Posted	**Lives in Govt. Quarter:** No	**Joining Date (Current designation):** 06-09-2016
**Current Pay Scale Hold:** 5	**Current Basic Pay:** 49090	

Service Information

**First Appointment Information:**	**In-service Training (IST):**	**Ad-hoc Information:**
Go. No.: সম/ননি./16/2002-82	Go. No.:	Go. No.:
Sl. No.: 512	Sl. No.:	Sl. No.:
Go. Date: 30-04-2003	Go. Date:	Go. Date:
**BCS/PSC Information**	**Service Confirmation Information**	**Senior Scale Pass:** Yes
BCS Batch No.: 21	Go. No.: Par -2/1A-2/2006/453	**Departmental Examination Pass:** Yes
BCS/PSC Regularization Go.:	Sl. No.: 175	**Experience in village:** Yes
BCS/PSC Regularization Sl. No.: 512	Go. Date: 19-10-2006	
BCS/PSC Regularization Date:		

Additional Designation(s) Information

Sl. No.	Additional Designation	Facility	Posted As	Join/From Date	Release/To Date
1	Asstt. Professor(Medicine)	Kurmitola 500 Bed General Hospital	Attached	29-12-2016	

Family Member Information

Spouse Information

No information available

Children Information

No information available

[illegible]ucational Information

Service exam

No information available

Educations

Sl.No.	Year	Educational Qualification	Name of Degree /Diploma /Certificate	Subject/ Discipline	Institution	Board/University	Division /Class	CGPA	Distinction /Award/ Honor
1	2011	Masters-MD (any of health sciences)	MD	Chest disease					
2	2009	Fellowship (FRCS, FCPS, FRCOG, etc.)	FCPS	Medicine					
3	1996	Bachelor-MBBS	MBBS						

Post Graduation Entry Information

No information available

Registration Information

No information available

Posting Information

Sl. No.	Designation	Posting Reason	Posted As	Place of Posting	Joining Date	Release Date
1	OSD	Promotion	Attached	Directorate General of Health Services (DGHS)	06-09-2016	
2	Jr. Consultant		Regular	Kurmitola 500 Bed General Hospital		03-09-2016

Promotion Information

Sl. No.	Promoted Designation	GO. No.	GO. date	GO. Sl. No.
1	OSD			

Deputation

No information available

Lien

No information available

Training Information (Local)

No information available

Training Information (Foreign)

No information available

Training Information (Foundation)

No information available

Publication/Achievement Information

Publication

No information available

Presentation

No information available

Affiliation

No information available

Award

No information available

Disciplinary Information

No information available

Leave

No information available

[illegible]

Signature 18/3/18

PERSONAL INFORMATION:

Name	:	Mohammad Aminul Islam
BA NO.	:	100591
Designation	:	Director And Project Director, Kurmitola General Hospital, Dhaka Cantonment.
Father's Name	:	Ansar Uddin Ahmed.
Date of Birth	:	01 January1961.
Present Address	:	Brigadier General. Director And Project Director, Kurmitola General Hospital, Dhaka Cantonment.
Permanent Address	:	Vill : Nandbar, PO : Asmankhali, PS : Alamdanga, Dist : Chuadanga.
Nationality	:	Bangladeshi
Religion	:	Islam
Sex	:	Male
Marital Status	:	Married
Blood Group	:	O+ (Positive)
Contact No	:	01769010201
NID	:	19611058598403560
Passport No	:	OC 9208771

Signature

Brigadier General Mohammad Aminul Islam
Director & Project Director
Kurmitola General Hospital
Dhaka Cantonment.

Educational Qualification (s):

Duration		School/College/ University	Examination Passed (Give Subject)	Division/ GPA Obtained	Year Of Passing The Exam
Form	To				
1976	1977	Bheramara High School	SSC	1^{St} Division	1997
1977	1980	Rajshahi Govt. College	HSC	1^{St} Division	1980
1980	1987	Rajshahi Medical College	MBBS	Passed	1987
Jul 2001	Jun 2002	Bangabandhu Medical University	MPH (PHA)	Passed	2002
Jun 2004	-	The College of Practitioners of Bangladesh	FCGP	Passed	2004
2009	2011	State University,Dhaka	MBA(Health Care Management)	Passed	2011

Signature

Brigadier General Mohammad Aminul Islam
Director & Project Director
Kurmitola General Hospital
Dhaka Cantonment.

CURRICULUM VITAE

Name:	
Rachel Colin-Jones	
**Present appointment:**	
Clinical Lead/Programme Manager<br>Oxford Vaccine Group	
**Address:**	
Oxford Vaccine Group<br>CCVTM<br>Churchill Hospital<br>OX3 7LE	
**Telephone number:**	**Email address:**
01865 611310	Rachel.colin-jones@paediatrics.ox.ac.uk
**Qualifications:**	
• 2010 - BSc (Hons) Children's Nursing<br>• 2014 - PGDip Specialist Community Public Health Nursing (Health Visitor)<br>• 2014 - Community Nurse Prescriber<br>• 2017 – MSc Public Health Nursing	
**Professional registration:**	
Nursing and Midwifery Council<br>NMC Pin 10E0300E<br>• 17.07.2010 - Registered Children's Nurse<br>• 01.09.2014 – Registered Specialist Community Public Health Nurse<br>• 01.09.2014 – Registered Community Practitioner Nurse Prescriber	
**Previous and other appointments:** *(Include previous appointments in the last 5 years and other current appointments.)*	
• Clinical Co-ordinator/Project Manager Sept 2017 – April 2018<br>• Clinical Research Nurse Oct 2016 – Sept 2017<br>• Specialist Community Public Health Nurse (Health Visitor) Sept 2014 – Oct 2016<br>• Specialist Community Public Health Nurse (Student) Sept 2013 – Sept 2014<br>• Paediatric Staff Nurse Full-time Sept 2010 – Sept 2013 (Part-time Sept 2013 – July 2015)<br>• Specialist Associate Lecturer Part-time Sept 2010 – Sept 2012	
**Research experience:** *(Summary of research experience, including the extent of your involvement. Refer to any specific clinical or research experience relevant to the current application.)*	
• **Sched 3** – Assessment of post booster antibody responses in UK infants given a reduced primary schedule of meningococcal group B and 13 valent pneumococcal conjugate vaccines **(Research Nurse)**<br>• **MedImmune** - A Phase2b Randomised, Double-blind, Placebo-controlled Study to Evaluate the Safety and Efficacy of MEDI8897, a Monoclonal Antibody With an	

Template CV for research applicants
Version 2, January 2007

Extended Half-life Against Respiratory Syncytial Virus, in Healthy Preterm Infants **(Lead Nurse)**

- **Pneumo 2017** - Cross-sectional study to establish the point prevalence of serotype 19A pneumococcal nasopharyngeal carriage of fully vaccinated children aged 13-48 months 7 years following introduction of PCV-13. **(Clinical Co-ordinator)**
- **TyVAC Nepal** - Assessing the impact of a Vi-Polysaccharide Conjugate Vaccine in preventing typhoid infections among Nepali children – a Phase III trial **(Clinical Lead/Programme Manager)**
- **TyVAC Bangladesh** - Assessing the impact of a Vi-Polysaccharide Conjugate Vaccine in preventing typhoid infection among Bangladeshi children – a Phase IIIb trial **(Clinical Lead/Programme Manager)**

Research training: *(Details of any relevant training in the design or conduct of research, for example in the Clinical Trials Regulations, Good Clinical Practice, consent or other training appropriate to non-clinical research. Give the date of the training.)*

- Good Clinical Practice (GCP) training – 25.05.2017
- Venepuncture training – 23.11.2016
- Infant and child immunisation training – 18.01.2017
- Consent taking training – 26.06.2017
- Conducting study visits training – 23.11.2016
- Understanding data management in clinical trials – 22.05.2017
- Basic Life Support and Anaphylaxis training – 03.07.2017
- Level 3 Safeguarding training – 28.02.2017
- MSc module in Advanced Research Methods –29.09.2017
- MSc Systematic Literature Review (Dissertation) –29.09.2017

Relevant publications: *(Give references to all publications in the last two years plus other publications relevant to the current application.)*

**Signature:** [signature]	**Date:** 01/05/2018

Template CV for research applicants
Version 2, January 2007

Curriculum Vitae

Name : Dr.Farhana Noman
Age : 54 years Sex: Female
Date of Birth : 05.01.1965
Religion : Islam
Marital Status : Married
Mother's Name : Mrs. Rawshan Ara
Father's Name : Md. Nazrul Islam Bhuiyan
Present Address : House-588(2nd fl),Road-08,Ave-04,Mirpur,Dhaka-1216
Permanent Address : 7,Gohailkandi,Mymensingh

Contact No. : 01715261000
E-mail : drfarhananoman@gmail.com

Academic attainments:

QUALIFICATION	INSTITUTION	YEAR OF COMPLETION	SUBJECT/AREA
M.B.B.S	Mymensingh Medical College & Hospital	1988	
DCH(Diploma in Child health)	BSMMU	2002	Paediatrics

History of Employment:

FROM	TO	NAME & ADDRESS OF THE ORGANIZATION	DEPARTMENT	JOB TITLE (Designation)
May 2012	Till	Kurmitola General Hospital	Paediatrics	Senior Consultant

Publication:

Noman F et al. Clinical profile and ultrasonography evaluation of brain in perinatal asphaxia.Bangladesh medical journal.2012

Certificate of Completion

The National Institutes of Health (NIH) Office of Extramural Research certifies that **Dr. Firdausi Qadri** successfully completed the NIH Web-based training course “Protecting Human Research Participants”.

Date of completion: 07/17/2014

Certification Number: 1505608

Certificate of Completion

The National Institutes of Health (NIH) Office of Extramural Research certifies that **JOHN CLEMENS** successlully completed the NIH Web-based training course "Protecting Human Research Participants".

Date of completion: 02/16/2016.

Certification Number: 2005605.

Certificate of Completion

The National Institutes of Health (NIH) Office of Extramural Research certifies that **Farhana Khanam** successfully completed the NIH Web-based training course "Protecting Human Research Participants".

Date of completion: 10/16/2017.

Certification Number: 2534788.

Certificate of Completion

The National Institutes of Health (NIH) Office of Extramural Research certifies that **Md. Arifuzzaman Khan** successfully completed the NIH Web-based training course "Protecting Human Research Participants".

Date of completion: 10/16/2017.

Certification Number: 2533543.

Certificate of Completion

The National Institutes of Health (NIH) Office of Extramural Research certifies that **Fahima Chowdhury** successfully completed the NIH Web-based training course "Protecting Human Research Participants".

Date of completion: 08/16/2016.

Certification Number: 2127647.

Certificate of Completion

The National Institutes of Health (NIH) Office of Extramural Research certifies that **Ashraful Islam Khan** successfully completed the NIH Web-based training course "Protecting Human Research Participants".

Date of completion: 10/16/2017.

Certification Number: 2533566.

Certificate of Completion

The National Institutes of Health (NIH) Office of Extramural Research certifies that **Dr. K Zaman** successfully completed the NIH Web-based training course “Protecting Human Research Participants”.

Date of completion: 02/28/2015

Certification Number: 1712891

Certificate of Completion

The National Institutes of Health (NIH) Office of Extramural Research certifies that **Shafiqul Sarker** successfully completed the NIH Web-based training course "Protecting Human Research Participants".

Date of completion: 06/09/2014

Certification Number: 1484127

NHS
National Institute for
Health Research
Clinical Research Network

Certificate of Attendance

Andrew Pollard

attended

Good Clinical Practice (GCP) Refresher:
A practical guide to ethical and scientific
quality standards in clinical research

on 02/08/2013

Sessions include:
GCP: the standards and why we have them
Study overview
Informed consent
Essential documents
GCP recent changes

Emma Lowe
NIHR CRN Learning and Development Lead

`

THE GLOBAL HEALTH NETWORK

Enabling research by sharing knowledge

Hereby Certifies that

KATHERINE THEISS-NYLAND

has completed the e-learning course

ICH GOOD CLINICAL PRACTICE

with a score of

94%

on

12/04/2017

This e-learning course has been formally recognised for its quality and content by the following organisations and institutions

THE GLOBAL HEALTH NETWORK Enabling research by sharing knowledge · WORLDWIDE ANTIMALARIAL RESISTANCE NETWORK WWARN · East African Consortium for Clinical Research EACCR · LSTM LIVERPOOL SCHOOL OF TROPICAL MEDICINE

This ICH E6 GCP Investigator Site Training meets the Minimum Criteria for ICH GCP Investigator Site Personnel Training identified by ***TransCelerate BioPharma*** *as necessary to enable mutual recognition of GCP training among trial sponsors.*

Global Health Training Centre
globalhealthtrainingcentre.org/elearning

Certificate Number 220174

RBM/RA/2015-048

icddr,b

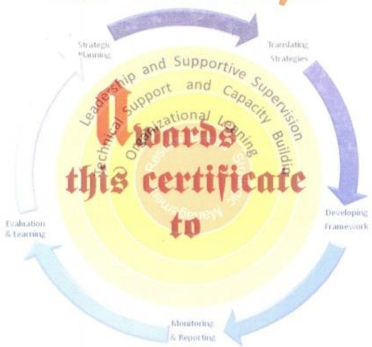

Awards this certificate to

Dr. Farhana Khanam

for attending the Certificate Program on

Results Based Management (RBM)

held during 01 & 02 September 2015

Director, RAS

Director, HR

Executive Director

Research Administration, icddr,b
68, Shahid Tajuddin Ahmed Sharani
Mohakhali, Dhaka 1212, Bangladesh

CITI PROGRAM

Completion Date 13-Sep-2017
Expiration Date 13-Sep-2019
Record ID 24578822

This is to certify that:

Samir K Saha

Has completed the following CITI Program course:

CITI Good Clinical Practice (Curriculum Group)
CITI Good Clinical Practice Course (Course Learner Group)
1 - Basic Course (Stage)

Under requirements set by:

International Vaccine Institute

CITI
Collaborative Institutional Training Initiative

Verify at www.citiprogram.org/verify/?wdde3ffda-ff4f-4a77-aa3f-6e6a3e06ded3-24578822

THE GLOBAL HEALTH NETWORK

Enabling research by sharing knowledge

Hereby Certifies that

MOHAMMAD AMINUL ISLAM

has completed the e-learning course

ICH GOOD CLINICAL PRACTICE E6 (R2)

with a score of

100%

on

19/03/2018

This e-learning course has been formally recognised for its quality and content by the following organisations and institutions

THE GLOBAL HEALTH NETWORK Enabling research by sharing knowledge · WWARN · EACCR · LSTM LIVERPOOL SCHOOL OF TROPICAL MEDICINE

This ICH E6 GCP Investigator Site Training meets the Minimum Criteria for ICH GCP Investigator Site Personnel Training identified by ***TransCelerate BioPharma*** *as necessary to enable mutual recognition of GCP training among trial sponsors.*

Global Health Training Centre
globalhealthtrainingcentre.org/elearning

Certificate Number 375730

THE GLOBAL HEALTH NETWORK

Enabling research by sharing knowledge

Hereby Certifies that

MD. MUBIN

has completed the e-learning course

ICH GOOD CLINICAL PRACTICE E6 (R2)

with a score of

100%

on

18/03/2018

This e-learning course has been formally recognised for its quality and content by the following organisations and institutions

THE GLOBAL HEALTH NETWORK Enabling research by sharing knowledge — WWARN — EACCR — LSTM Liverpool School of Tropical Medicine

*This ICH E6 GCP Investigator Site Training meets the Minimum Criteria for ICH GCP Investigator Site Personnel Training identified by **TransCelerate BioPharma** as necessary to enable mutual recognition of GCP training among trial sponsors.*

Global Health Training Centre
globalhealthtrainingcentre.org/elearning

Certificate Number 375308

THE GLOBAL HEALTH NETWORK

Enabling research by sharing knowledge

Hereby Certifies that

ABDULLAHEL KAFEE

has completed the e-learning course

ICH GOOD CLINICAL PRACTICE E6 (R2)

with a score of

94%

on

18/03/2018

This e-learning course has been formally recognised for its quality and content by the following organisations and institutions

This ICH E6 GCP Investigator Site Training meets the Minimum Criteria for ICH GCP Investigator Site Personnel Training identified by ***TransCelerate BioPharma*** *as necessary to enable mutual recognition of GCP training among trial sponsors.*

Global Health Training Centre
globalhealthtrainingcentre.org/elearning

Certificate Number 375306

Certificate of Attendance

This is to certify that

Rachel Colin-Jones

Attended

Good Clinical Practice Training (CTIMP)
Core Module
Thursday 25th May,2017

At

The Joint Research Office-Meeting Room 1, Block 60,
Churchill Hospital

Signed:……………………………

Heather House
Head of Clinical Trials & Research Governance

THE GLOBAL HEALTH NETWORK

Enabling research by sharing knowledge

Hereby Certifies that

FARHANA NOMAN

has completed the e-learning course

ICH GOOD CLINICAL PRACTICE E6 (R2)

with a score of

100%

on

05/05/2018

This e-learning course has been formally recognised for its quality and content by the following organisations and institutions

THE GLOBAL HEALTH NETWORK Enabling research by sharing knowledge

WORLDWIDE ANTIMALARIAL RESISTANCE NETWORK WWARN

East African Consortium for Clinical Research EACCR

LSTM LIVERPOOL SCHOOL OF TROPICAL MEDICINE

This ICH E6 GCP Investigator Site Training meets the Minimum Criteria for ICH GCP Investigator Site Personnel Training identified by ***TransCelerate BioPharma*** *as necessary to enable mutual recognition of GCP training among trial sponsors.*

Global Health Training Centre
globalhealthtrainingcentre.org/elearning

Certificate Number 399893

Gender analysis tool:

**Relation to diarrheal illness:**	**Are there differences between male and female* in**	**How do biological differences between women and men**influence their**	**How do the different roles and activities of women and men** affect their**	**How do gender norms / values affect women and men's*******	**How do access to and control over resources affect women and **men**'s**
Vulnerability: Incidence Prevalence	No data available				
Health seeking behaviour	No data available but according to our culture it can be assumed that females are neglected.				
Ability to access health services	No data available, females are dependent on their parents or their husbands income				
Preventive and treatment options, responses to treatment, prevention or rehabilitation	No such differences have been reported	No difference due to biological characteristics	Men usually have more treatment options	Men seek care more often as they are usually the head of the family	Men seek care more often as they have better access to resources
Experience with health services and health providers	Most government medical college hospitals in Bangladesh have more male beds than female beds	No data available	No data available	No data available	No data available
Outcome of health problem: e.g. detection, prevention, recovery, disability, death	No difference reported	No data available	Due to late health care seeking women may have negative effect on health outcome.	No difference	No difference reported
Consequences (economic & social, including attitudinal)	Economical burden caused by men and women cusses social burden	Biological differences may have such consequences	Economic burden is more for men as they are usually the wage earner	Not applicable	No data available

APPENDIX A1

Verbal Consent Form for Census

Protocol No. PR-17115	Version No. 5.00	Date: 18-11-2020

Protocol Title: Assessing the impact of a Vi-Polysaccharide Conjugate Vaccine in preventing typhoid infection among Bangladeshi children – a Phase IIIb trial.

Investigator's name: Dr Firdausi Qadri, Principal Investigator (PI) of the protocol

Organization: icddr,b

Purpose of the research

Background

Typhoid fever is a serious infectious disease transmitted by drinking water or eating food that has a certain type of bacteria in it. The disease is common in developing countries including Bangladesh, where the sanitation and pure water supply are not adequate. Now the disease is more commonly found in children, and immediate and appropriate treatment is needed to prevent complications. Other available vaccines against typhoid fever are not always effective in children. The Vi-Polysaccharide Typhoid Conjugate Vaccine (Vi-TCV) is a promising vaccine for controlling typhoid fever in children. It is important to study the effectiveness of the Vi-TCV vaccine by immunizing Bangladeshi children and to inform the decision makers for introduction of Vi-TCV into the routine expanded programme on immunization (EPI) in Bangladesh.

The aim of the overall study is to evaluate the effectiveness of the typhoid vaccine. Efficacy of the Vi-TCV vaccine will be evaluated with the Japanese encephalitis vaccine (SA14-14-2) in a participant- and observer-blinded, cluster-randomized trial design in this study. So, we have vaccinated the children aged 9 month to <16 yearsin the study area of Mirpur.

Reasons for inviting the specific person to participate in the study

You are invited to take part in this study because your household resides in our study area of Mirpur, Dhaka. In order for us to look at how well this typhoid vaccine works, we need to collect some baseline information from the households in Mirpur.

Roles of the participants in this study

If you agree to participate in this study, following activities will be carried out:

1. We will carry out the 6 monthly census in the study area of Mirpur.
2. We would like to diagnose participants who have typhoid fever and follow them up for some time.

There will be separate invitations to participate in the different parts of the study and detailed information will be given by the researchers at a later time.

If you agree to take part in the census, then the following steps will be taken:

We will explain the study to you and we will ask you to provide some information about your household. We will ask you:

- how many people live in your household;
- how old they are;
- where you get your water from;
- where the latrine is and what type it is.

This will take approximately 40 to 60 minutes of your time. This interview will be confidential, and your household will be given a study number; individuals will be given a member number within the household.

A member of our team will visit 6 monthly to ask you, or if you are not at home, another adult member of your household, whether there have been any changes in your household, for example, whether there are more or less people living in your home. If any child from your household has been vaccinated as a part of this study, the parents/guardians of the child will be asked about any significant illnesses, treatments and surgeries occurring since the last census. A vaccine will be offered to any child in your household who is eligible, but has not received the study vaccine allocated to the cluster before. We may also ask you at these visits whether any member of your household has been sick with typhoid fever.

We would appreciate you informing other members of your household about the study after you agree to participate. We may approach them during the study period if you are not at home. These visits will be shorter. Our study will continue for next 3 years.

Genetic test

We will not collect any samples for carrying out the census. If you/your child are involved with other components of the study, we may collect blood samples and ask your permission to carry out genetic tests on that sample.

Risk and benefit

There is no direct benefit to you/your child or other members of your household. However, if you or a member of your household gets sick with a fever that is 38°C and/or above and/or develops a fever that lasts for $\geq$ 2 days during the time of our study, you will have the opportunity to seek medical advice from designated health facilities without any consultation fee.

If at this visit to the healthcare facility, you/your household member were found to have typhoid fever, the cost of your treatment for typhoid fever as an outpatient would be free and covered by the study. We will give you a card with the contact details of the team.

Information from this and the other linked studies will help the Government to make choices about the use of this vaccine to help prevent typhoid fever. It will not cost you anything to take

part in this study.

Principle of compensation

The participants shall also be provided with the best possible, free treatment, for any research related injuries. You will not have to pay to us, and we will also not pay to you for participating in the study.

Privacy, anonymity and confidentiality

All information that we collect during the study is strictly private and confidential. You/your child will be identified by a number. Your/your child's name and address will not be revealed in any output from this study.

Storage of data and biological samples

We will not collect any biological samples as part of the census. We will store all information collected from you in password protected computers and paper documents will be locked in file cabinets. At the end of the trial, all data and records will be electronically retained until the youngest child participating in the trial reaches 21 or for five years following completion of the research, whichever is longer.

Future use of data and/or biological samples

The information collected about you/your child may be shared with representatives of the ethics committee to check that the study is being carried out properly and with researchers in other countries without revealing any personal data such as name and address.

The University of Oxford is responsible for ensuring the safe and proper use of any personal information you provide, solely for research purposes.

Right not to participate and withdraw

It is your choice whether you/your child take part in this study and it is entirely voluntary.
You/your child can withdraw from the study at any time by contacting the research team. You do not need to give a reason. If you choose to withdraw from the study, we will still use the information you gave us before your/your child's withdrawal. We will inform you of any changes to the study.

Communication for Queries, Concerns and Complaints

We will be happy to provide you further information about the study any time. If you want to know more about our research, you may ask now or may contact the following investigators at a later suitable time, either meeting with them personally or by contacting them over telephone, at the following addresses:

Dr. Firdausi Qadri
Address of investigator: Enteric and Respiratory Infections, IDD. icddr,b, Mohakhali, Dhaka

1212. Telephone: +880-2-8812529, Extension No. 2431.

If you want to know more about your rights as a research participant you may contact IRB Secretariat, RA, M. A. Salam Khan, phone no: 9886498 or PABX 8860523-32 Extension. 3206 personally at icddr,b or by contact by telephone.

Thank you for your co-operaton.

Based on the above, I have voluntarily agreed to enrol my household in this research study

(Please place a tick in the box, if you agree) ☐

Name of the personnel securing consent:______________________________________

Signature of the personnel securing consent:_________________Date______________

Note- A signed and dated copy/photocopy of this document will be given to you.

APPENDIX A2(i)

Consent form for vaccinees (Pilot phase) (Age 9 months - <11 years)

Protocol No. PR- 17115	Version No. 4.00	Date: 27-08-2018

Protocol Title: Assessing the impact of a Vi-Polysaccharide Conjugate Vaccine in preventing typhoid infection among Bangladeshi children – a Phase IIIb trial

Investigator's name: Dr Firdausi Qadri, Principal Investigator (PI) of the protocol

Organization: icddr,b

Purpose of the research

Background

Typhoid fever is a serious infectious disease transmitted by taking water and food contaminated with organism. The disease is common in the developing countries including Bangladesh, where the sanitation and pure water supply are not adequate. Now the disease is more commonly found in children, and immediate and appropriate treatment is needed to prevent complication of the patients. Commercially available vaccines against typhoid fever are not so effective in children. The Vi-Polysaccharide typhoid Conjugate Vaccine (Vi-TCV) is the most promising vaccine for controlling typhoid fever. It is important to study the effectiveness of the Vi-TCV vaccine by immunizing Bangladeshi children and to inform the decision makers for introduction of Vi-TCV into the routine expanded programme on immunization (EPI) in Bangladesh.

The aim of the overall study is to evaluate the effectiveness of the typhoid vaccine.

Efficacy of the Vi-TCV vaccine will be evaluated with the Japanese encephalitis vaccine (SA14-14-2) in a participant- and observer-blinded, cluster-randomized trial design in this study. We will vaccinate 200 children between 9 months and <16 years of age to see the safety of the vaccine and the immune response induced after vaccination. We shall vaccinate the children residing in Mirpur with Vi-TCV or SA14-14-2.

Reasons for inviting the specific person to participate in the study

You are invited to take part because your child is aged between 9 months and <16 years and you reside in a ward of Mirpur.

Roles of the participants in this study

If you give consent to allow your child to participate in this study, then the following steps will be done:

1. **Day of vaccination (Day 0):**
    - On the 1st day, we shall examine your child to make sure he/she is healthy. If your child meets all eligibility criteria, your child will get Vi-TCV or SA14-14-2 vaccine. However, we do not want to identify people who are receiving the typhoid vaccine or the control Japanese encephalitis vaccine. This will be done by a process called "randomization" which is like flipping a coin, where all participants will have equal chance to receive typhoid or control vaccine.

2. **Follow-up at Day 7 visit:**
    - As part of follow-up active surveillance at 7 days post-vaccination, we will collect parent/guardian-reported information on adverse events following immunization (AEFIs). We will encourage the parents/guardians to use diary cards to record adverse events post-vaccination, to help recall symptom and duration. If any adverse event happens, follow-up will occur at in person home visit.
    - Information on verbal reconfirmation of consent for participation in the trial will be collected. Report from parent/guardian of adverse events related to vaccination, including: pain, swelling, hardness, fever, etc., and use of medications following vaccination will be collected and reported to the safety monitoring committee.
    - We will reiterate our contact details and give instruction the parents/guardians to attend the health care facilities in the case of a fever for ≥2 days and/or presenting temperature ≥38°C.

At the end of 2 years we will ask you to attend the study healthcare facility and we will let you know what vaccine your child received, and include it in your child's health record. If he/she received the Japanese Encephalitis vaccine, we will offer to give him/her the typhoid vaccine.

Genetic test

We will not perform any genetic test with the specimens collected from your child.

Risk and benefits

There is no routine vaccination for typhoid or Japanese encephalitis in Bangladesh. All participants enrolled will have the benefit of receiving either Vi-TCV, which will likely provide additional protection against typhoid, or Japanese encephalitis whichis endemic in Bangladesh. All participants will have access to free and accurate health assessments and diagnostics at the icddr,b hospitals, as well as in other trial health facilities set up in the community, for all episodes of suspected typhoid fever occurring during the study. Whilst participants would not necessarily benefit directly from this, a positive result from this trial could lead to implementation of a Vi-TCV vaccine programme in typhoid endemic areas, including Bangladesh.

Different studies have shown that the Vi-TCV vaccine is safe and well tolerated. This, however, is the first controlled study involving so many participants and there may be rare adverse events hereto unidentified that may become apparent in this study. We will be conducting safety monitoring as a secondary outcome of this study.

Principle of compensation

The participants shall also be provided best possible, free treatment, for research related injuries. You will not have to pay to us, and we will also not pay to you for participating in the study. However, we will only pay taka 500 to support the conveyance cost for each visit.

Privacy, anonymity and confidentiality

All information that we collect during the study is strictly private and confidential. You/your child will be identified by a number.

Your/your child's name and your address will not be revealed in any output from this study.

Storage of data and biological samples

We will not collect any biological samples. We will store all information collected from you in password protected computers and paper documents will be locked in file cabinets. At the end of the trial, all data and records will be electronically retained until the youngest child participating in the trial reaches 21 or for five years following completion of the research, whichever is longer.

Future use of data and/or biological samples

The information collected about you/your child may be shared with representatives of the ethics committee to check that the study is being carried out properly and with researchers in other countries without revealing any personal data such as name and address.

The University of Oxford is responsible for ensuring the safe and proper use of any personal information you provide, solely for research purposes.

Right not to participate and withdraw

It is your choice whether you/your child take part in this study and it is entirely voluntary.

Your child can withdraw from the study at any time by contacting the research team (details below). You do not need to give a reason. If your child chooses to withdraw from the study, we will still use the information you gave us before your/your child's withdrawal. We will inform you of any changes to the study.

Communication for Queries, Concerns and Complaints

We will be happy to provide you further information about the study any time. If you want to know more about the research, you may ask now or may contact the following investigators at your later suitable time, either meeting with them personally or by contacting them over telephone, at the following addresses:

Dr. Firdausi Qadri, Address of investigator: Enteric and Respiratory Infections, IDD. icddr,b, Mohakhali, Dhaka 1212. Telephone: +880-2-8812529, Extension No. 2431.

If you want to know more about your rights as a research participant you may contact IRB Secretariat, RA, M. A. Salam Khan, phone no: 9886498 or PABX 8860523-32 Extension. 3206 personally at icddr,b or by contact by telephone.
If you agree to our proposal of enrolling your child in our study, please indicate that by putting your signature or your left thumb impression at the specified space below.
Thank you for your cooperation

Indicating consent

The investigators/ study staff has explained to me and I have fully understood the purpose, procedures, risks and benefits of participating in this study, my child's rights as a research participant and the confidential handling of the information and records. I understand that I may withdraw my child from the study at any time without giving any reason. I understand that I would also be able to get further information in future and that my child's name and/or identity will not be used in the analysis of data and in sharing the results with others.

Based on the above, I have voluntarily agreed to enrol my child for vaccination (Please place a tick in the box, if you agree) ☐

Based on the above, I have voluntarily agreed to enrol my child for providing post-vaccination follow-up information (Please place a tick in the box, if you agree) ☐

Participant's age: 09 months - <11 years

Name of participant: ______________________________________

Name of Father/Mother/guardian of a minor participant _________________

Signature or left thumbprint of Father/Mother/guardian: ____________________Date_______________

Name of witness: ____

Signature of witness: _______________________________Date______________

Name of study personnel securing consent: __________________________

Signature of study personnel securing consent:__________________________Date______________

Note- A signed and dated copy/photocopy of this document will be given to you.

APPENDIX A2 (ii)

Consent form for vaccinees (Pilot phase) (Age 11 years - <16 years)

Protocol No. PR- 17115	Version No. 4.00	Date: 27-08-2018

Protocol Title: Assessing the impact of a Vi-Polysaccharide Conjugate Vaccine in preventing typhoid infection among Bangladeshi children – a Phase IIIb trial

Investigator's name: Dr Firdausi Qadri, Principal Investigator (PI) of the protocol

Organization: icddr,b

Purpose of the research

Background
Typhoid fever is a serious infectious disease transmitted by taking water and food contaminated with organism. The disease is common in the developing countries including Bangladesh, where the sanitation and pure water supply are not adequate. Now the disease is more commonly found in children, and immediate and appropriate treatment is needed to prevent complication of the patients. Commercially available vaccines against typhoid fever are not so effective in children. The Vi-Polysaccharide typhoid Conjugate Vaccine (Vi-TCV) is the most promising vaccine for controlling typhoid fever. It is important to study the effectiveness of the Vi-TCV vaccine by immunizing Bangladeshi children and to inform the decision makers for introduction of Vi-TCV into the routine expanded programme on immunization (EPI) in Bangladesh.

The aim of the overall study is to evaluate the effectiveness of the typhoid vaccine.

Efficacy of the Vi-TCV vaccine will be evaluated with the Japanese encephalitis vaccine (SA14-14-2) in a participant- and observer-blinded, cluster-randomized trial design in this study. We will vaccinate 200 children between 9 months and <16 years of age to see the safety of the vaccine and the immune response induced after vaccination. We shall vaccinate the children residing in Mirpur with Vi-TCV or SA14-14-2.

Reasons for inviting the specific person to participate in the study

You are invited to take part because your child is aged between 9 months and <16 years and you reside in a ward of Mirpur.

Roles of the participants in this study

If you give consent to allow your child to participate in this study, then the following steps will be done:

1. Day of vaccination (Day 0):

- On the 1st day, we shall examine your child to make sure he/she is healthy. If your child meets all eligibility criteria, your child will get Vi-TCV or SA14-14-2 vaccine. However, we do not want to identify people who are receiving the typhoid vaccine or the control Japanese encephalitis vaccine. This will be done by a process called "randomization" which is like flipping a coin, where all participants will have equal chance to receive typhoid or control vaccine.

2. Follow-up at Day 7 visit:

- As part of follow-up active surveillance at 7 days post-vaccination, we will collect parent/guardian-reported information on adverse events following immunization (AEFIs). We will encourage the parents/guardians to use diary cards to record adverse events post-vaccination, to help recall symptom and duration. If any adverse event happens, follow-up will occur at in person home visit.
- Information on verbal reconfirmation of consent for participation in the trial will be collected. Report from parent/guardian of adverse events related to vaccination, including: pain, swelling, hardness, fever, etc., and use of medications following vaccination will be collected and reported to the safety monitoring committee.
- We will reiterate our contact details and give instruction the parents/guardians to attend the health care facilities in the case of a fever for ≥2 days and/or presenting temperature ≥38°C.

At the end of 2 years we will ask you to attend the study healthcare facility and we will let you know what vaccine your child received, and include it in your child's health record. If he/she received the Japanese Encephalitis vaccine, we will offer to give him/her the typhoid vaccine.

Genetic test

We will not perform any genetic test with the specimens collected from your child.

Risk and benefits

There is no routine vaccination for typhoid or Japanese encephalitis in Bangladesh. All participants enrolled will have the benefit of receiving either Vi-TCV, which will likely provide additional protection against typhoid, or Japanese encephalitis, which is endemic in Bangladesh. All participants will have access to free and accurate health assessments and diagnostics at the icddr,b hospitals, as well as in other trial health facilities set up in the community, for all episodes of suspected typhoid fever occurring during the study. Whilst participants would not necessarily benefit directly from this, a positive result from this trial could lead to implementation of a Vi-TCV vaccine programme in typhoid endemic areas, including Bangladesh.

Different studies have shown that the Vi-TCV vaccine is safe and well tolerated. This, however, is the first controlled study involving so many participants and there may be rare adverse events

hereto unidentified that may become apparent in this study. We will be conducting safety monitoring as a secondary outcome of this study.

Principle of compensation

The participants shall also be provided best possible, free treatment, for research related injuries. You will not have to pay to us, and we will also not pay to you for participating in the study. However, we will only pay taka 500 to support the conveyance cost for each visit.

Privacy, anonymity and confidentiality

All information that we collect during the study is strictly private and confidential. You/your child will be identified by a number.

Your/your child's name and your address will not be revealed in any output from this study.

Storage of data and biological samples

We will not collect any biological samples. We will store all information collected from you in password protected computers and paper documents will be locked in file cabinets. At the end of the trial, all data and records will be electronically retained until the youngest child participating in the trial reaches 21 or for five years following completion of the research, whichever is longer.

Future use of data and/or biological samples

The information collected about you/your child may be shared with representatives of the ethics committee to check that the study is being carried out properly and with researchers in other countries without revealing any personal data such as name and address.

The University of Oxford is responsible for ensuring the safe and proper use of any personal information you provide, solely for research purposes.

Right not to participate and withdraw

It is your choice whether you/your child take part in this study and it is entirely voluntary.

Your child can withdraw from the study at any time by contacting the research team (details below). You do not need to give a reason. If your child chooses to withdraw from the study, we will still use the information you gave us before your/your child's withdrawal. We will inform you of any changes to the study.

Communication for Queries, Concerns and Complaints

We will be happy to provide you further information about the study any time. If you want to know more about the research, you may ask now or may contact the following investigators at your later suitable time, either meeting with them personally or by contacting them over telephone, at the following addresses:

Dr. Firdausi Qadri, Address of investigator: Enteric and Respiratory Infections, IDD. icddr,b, Mohakhali, Dhaka 1212. Telephone: +880-2-8812529, Extension No. 2431.

If you want to know more about your rights as a research participant you may contact IRB Secretariat, RA, M. A. Salam Khan, phone no: 9886498 or PABX 8860523-32 Extension. 3206 personally at icddr,b or by contact by telephone.

If you agree to our proposal of enrolling your child in our study, please indicate that by putting your signature or your left thumb impression at the specified space below.
Thank you for your cooperation

Indicating consent

The investigators/ study staff has explained to me and I have fully understood the purpose, procedures, risks and benefits of participating in this study, my child's rights as a research participant and the confidential handling of the information and records. I understand that I may withdraw my child from the study at any time without giving any reason. I understand that I would also be able to get further information in future and that my child's name and/or identity will not be used in the analysis of data and in sharing the results with others.

Based on the above, I have voluntarily agreed to enrol my child for vaccination
(Please place a tick in the box, if you agree) ☐

Based on the above, I have voluntarily agreed to enrol my child for providing post-vaccination follow-up information (Please place a tick in the box, if you agree) ☐

Assent of minors (for children aged 11 years - <16years):

Name of participant:____

Signature or left thumbprint of participant:________________________ Date_____________

Name of Mother/Father/guardian of a minor participant _____________________

Signature or left thumbprint of Father/Mother/guardian:__________________Date_____________

Name of witness: ____

Signature of witness:_____________________________Date_____________

Name of study personnel securing consent: ________________________

Signature of study personnel securing consent:________________________Date_____________

Note- A signed and dated copy/photocopy of this document will be given to you.

APPENDIX A3 (i)

Consent Form for Vaccinees (9 months - <11 years)

Protocol No. PR-17115	Version No. 7.00	Date: 14-11-2018

Protocol Title: Assessing the impact of a Vi-Polysaccharide Conjugate Vaccine in preventing typhoid infection among Bangladeshi children – a Phase IIIb trial.

Investigator's name: Dr Firdausi Qadri, Principal Investigator (PI) of the protocol

Organization: icddr,b

Purpose of the research

Background

Typhoid fever is a serious infectious disease transmitted by taking water and food contaminated with organism. The disease is common in the developing countries including Bangladesh, where the sanitation and pure water supply are not adequate. Now the disease is more commonly found in children, and immediate and appropriate treatment is needed to prevent complication of the patients. Commercially available vaccines against typhoid fever are not so effective in children. The Vi-Polysaccharide typhoid Conjugate Vaccine (Vi-TCV) is the most promising vaccine for controlling typhoid fever. It is important to study the effectiveness of the Vi-TCV vaccine by immunizing Bangladeshi children and to inform the decision makers for introduction of Vi-TCV into the routine expanded programme on immunisation (EPI) in Bangladesh.

The aim of the overall study is to evaluate the effectiveness of the typhoid vaccine.

Efficacy of the Vi-TCV vaccine will be evaluated with the Japanese encephalitis vaccine (SA14-14-2) in a participant- and observer-blinded, cluster-randomized trial design in this study. To determine the relative and absolute rate reduction of typhoid fever in recipients of Vi-TCV (total vaccine protection), we will immunize the children aged 9 month to <16 years residing in Mirpur with Vi-TCV or SA14-14-2.

Reasons for inviting the specific person to participate in the study

You are invited to participate in the study, because your child is aged of 9 months to <16 years and your household had already taken part in our census survey.

Roles of the participants in this study

If you agree to our proposal to include your child in our study, then the following steps would be done:

1. **Day of vaccination (Day 0):**

If your child is a married girl, we will collect a sample of urine for pregnancy test before vaccination. Your child will get Vi-TCV or SA14-14-2 vaccine if he/she meets all eligibility criteria. However, we do not want to identify people who are receiving the typhoid vaccine or the control Japanese encephalitis vaccine. This will be done by a process called "randomization" which is like flipping a coin, where all participants will have equal chance to receive typhoid or control vaccine.

2. **Follow-up at 6 monthly visits (for all participants):**

At six month intervals during the following two years since baseline (6 months, 12 months, 18 months, and 24 months), census updates of the population will be done in all clusters. At these updates, vaccinated participants parents/guardians will be queried about any fever or illness your child may have had since the last census, any medications they have received, about any time off school or work for illness, and any surgery or serious adverse events during that period.

3. **Follow-up visits for fever patients:**

If your child has fever for ≥2 days and/or presenting temperature of ≥ 38°C at any time during the following 2 years we would ask you to take him/her to the study healthcare facility. You will receive a card mentioning that your child is in the study and will not have to pay consultation fees.

At the end of 2 years we will ask you to attend the study healthcare facility and we will let you know what vaccine your child received, and include it in your child's health record. If they received the Japanese Encephalitis vaccine, we will offer to give him/her the typhoid vaccine.

Genetic test

We will not collect any sample for this vaccination component. If you/your child are involved with other components of the study, we may collect blood samples and ask your permission to carry out genetic test on that sample.

Risk and benefits

There is no routine vaccination for typhoid or Japanese encephalitis in Bangladesh. All participants enrolled will have the benefit of receiving either Vi-TCV, which will likely provide additional protection against typhoid, or SA14-14-2 against Japanese encephalitis, which is endemic in Bangladesh.

All participants will have access to free and accurate health assessments and diagnostics at the icddr,b hospitals, as well as in other trial health facilities set up in the community, for all episodes of suspected typhoid fever occurring during the study. Whilst participants would not necessarily benefit directly from this, a positive result from this trial could lead to implementation of a Vi-TCV vaccine programme in typhoid endemic areas, including Bangladesh.

Different studies have shown that the Vi-TCV vaccine is safe and well tolerated. This, however, is the first controlled study involving so many participants and there may be rare adverse events that may be identified in this study. We will be conducting safety monitoring as a secondary outcome of this study. All participants will have access to a medical doctor to seek advice and to report any adverse events following vaccination.

Principle of compensation

The participants shall also be provided best possible, free treatment, for research related injuries. You will not have to pay to us, and we will also not pay to you for participating in the study.

Privacy, anonymity and confidentiality

All information that we collect during the study is strictly private and confidential. You/your child will be identified by a number. You/your child's name and your address will not be revealed in any output from this study.

Storage of data and biological samples

We will not collect any biological sample from your child for the purpose of vaccination. We will store all information collected from you in password protected computers and printed copies in locked file cabinet. At the end of the trial, all data and records will be electronically retained until the youngest child participating in the trial reaches 21 or for five years following completion of the research, whichever is longer.

Future use of data and/or biological samples

The information collected about your child may be shared with representatives of the ethics committee to check that the study is being carried out properly and with researchers in other countries without revealing any personal data such as name and address.

The University of Oxford is responsible for ensuring the safe and proper use of any personal information you provide, solely for research purposes.

Right not to participate and withdraw

It is your choice whether your child takes part in this study and it is entirely voluntary.

Your child can withdraw from the study at any time by contacting the research team. You do not need to give a reason. If you choose to withdraw your child from the study, we will still use the

information you gave us before your/your child's withdrawal. We will inform you of any changes to the study.

Communication for Queries, Concerns and Complaints

If you want to know more about our research, you may ask them now or may contact the following investigators at your later suitable time, either meeting with them personally or by contacting them over telephone, at the following addresses:

Dr. Firdausi Qadri

Address of investigator: Enteric and Respiratory Infections, IDD. icddr,b, Mohakhali, Dhaka 1212. Telephone: +880-2-8812529, Extension No. 2431.

If you want to know more about your rights as a research participant you may contact IRB Secretariat, RA, M. A. Salam Khan, phone no: 9886498 or PABX 8860523-32 Extension. 3206 personally at icddr,b or by contact by telephone.

Thank you for your co-operaton.

If you agree to our proposal of enrolling you/your patient in our study, please indicate that by putting your signature or your left thumb impression at the specified space below.

Indicating consent

The investigators/ study staff have explained to me and I have fully understood the purpose, procedures, risks and benefits of participating in this study, my child's rights as a research participant and the confidential handling of the samples, information and records. I understand that I may withdraw my child from the study at any time without giving any reason. I understand that I would also be able to get further information in future and that my child's name and/or identity will not be used in the analysis of data and in sharing the results with others.

Based on the above, I have voluntarily agreed to enrol my child for vaccination
(Please place a tick in the box, if you agree) ☐

Based on the above, I have voluntarily agreed to provide follow-up information
(Please place a tick in the box, if you agree) ☐

I agree to be contacted after the end of this study by the research team
(Please place a tick in the box, if you agree) ☐

Participant's age: 9 months - <11 years

Name of participant: ______________________________________

Name of Father/Mother/ /guardian of a minor participant _____________________

Signature or left thumbprint of Father/Mother/guardian:_________________Date ___________

Name of witness:____

Signature of witness: ____________________________ Date ___________

Name of the personnel securing consent: ______________________________________

Signature of the personnel securing consent:___________ Date ___________

Note- A signed and dated copy/photocopy of this document will be given to you

APPENDIX A3 (ii)

Consent Form for Vaccinees (11 years-<16 years)

Protocol No. PR-17115	Version No. 8.00	Date: 14-11-2018

Protocol Title: Assessing the impact of a Vi-Polysaccharide Conjugate Vaccine in preventing typhoid infection among Bangladeshi children – a Phase IIIb trial.

Investigator's name: Dr Firdausi Qadri, Principal Investigator (PI) of the protocol

Organization: icddr,b

Purpose of the research

Background

Typhoid fever is a serious infectious disease transmitted by taking water and food contaminated with organism. The disease is common in the developing countries including Bangladesh, where the sanitation and pure water supply are not adequate. Now the disease is more commonly found in children, and immediate and appropriate treatment is needed to prevent complication of the patients. Commercially available vaccines against typhoid fever are not so effective in children. The Vi-Polysaccharide typhoid Conjugate Vaccine (Vi-TCV) is the most promising vaccine for controlling typhoid fever. It is important to study the effectiveness of the Vi-TCV vaccine by immunizing Bangladeshi children and to inform the decision makers for introduction of Vi-TCV into the routine expanded programme on immunisation (EPI) in Bangladesh.

The aim of the overall study is to evaluate the effectiveness of the typhoid vaccine.

Efficacy of the Vi-TCV vaccine will be evaluated with the Japanese encephalitis vaccine (SA14-14-2) in a participant- and observer-blinded, cluster-randomized trial design in this study. To determine the relative and absolute rate reduction of typhoid fever in recipients of Vi-TCV (total vaccine protection), we will immunize the children aged 9 month to <16 years residing in Mirpur with Vi-TCV or SA14-14-2.

Reasons for inviting the specific person to participate in the study

You are invited to participate in the study, because your child is aged of 9 months to <16 years and your household had already taken part in our census survey.

Roles of the participants in this study

If you agree to our proposal to include your child in our study, then the following steps would be done:

1. **Day of vaccination (Day 0):**

If your child is a married girl, we will collect a sample of urine for pregnancy test before vaccination.Your child will get Vi-TCV or SA14-14-2 vaccine if he/she meets all eligibility criteria. However, we do not want to identify people who are receiving the typhoid vaccine or the control Japanese encephalitis vaccine. This will be done by a process called "randomization" which is like flipping a coin, where all participants will have equal chance to receive typhoid or control vaccine.

2. **Follow-up at 6 monthly visits (for all participants):**

At six month intervals during the following two years since baseline (6 months, 12 months, 18 months, and 24 months), census updates of the population will be done in all clusters. At these updates, vaccinated participants parents/guardians will be queried about any fever or illness your child may have had since the last census, any medications they have received, about any time off school or work for illness, and any surgery or serious adverse events during that period.

3. **Follow-up visits for fever patients:**

If your child has fever for ≥2 days and/or presenting temperature of ≥ 38°C at any time during the following 2 years we would ask you to take him/her to the study healthcare facility. You will receive a card mentioning that your child is in the study and will not have to pay consultation fees.

At the end of 2 years we will ask you to attend the study healthcare facility and we will let you know what vaccine your child received, and include it in your child's health record. If they received the Japanese Encephalitis vaccine, we will offer to give him/her the typhoid vaccine.

Genetic test

We will not collect any sample for this vaccination component. If you/your child are involved with other components of the study, we may collect blood samples and ask your permission to carry out genetic test on that sample to look at why some people get typhoid and others do not.

Risk and benefits

There is no routine vaccination for typhoid or Japanese encephalitis in Bangladesh. All participants enrolled will have the benefit of receiving either Vi-TCV, which will likely provide additional protection against typhoid, or SA14-14-2 against Japanese encephalitis, which is endemic in Bangladesh.

All participants will have access to free and accurate health assessments and diagnostics at the icddr,b hospitals, as well as in other trial health facilities set up in the community, for all episodes of suspected typhoid fever occurring during the study. Whilst participants would not necessarily benefit directly from this, a positive result from this trial could lead to implementation of a Vi-TCV vaccine programme in typhoid endemic areas, including Bangladesh.

Different studies have shown that the Vi-TCV vaccine is safe and well tolerated. This, however, is the first controlled study involving so many participants and there may be rare adverse events that may be identified in this study. We will be conducting safety monitoring as a secondary outcome of this study. All participants will have access to a medical doctor to seek advice and to report any adverse events following vaccination.

Principle of compensation

The participants shall also be provided best possible, free treatment, for research related injuries. You will not have to pay to us, and we will also not pay to you for participating in the study.

Privacy, anonymity and confidentiality

All information that we collect during the study is strictly private and confidential. You/your child will be identified by a number. Your/your child's name and your address will not be revealed in any output from this study.

Storage of data and biological samples

We will not collect any biological sample from your child for the purpose of vaccination. We will store all information collected from you in password protected computers and printed copies in locked file cabinet. At the end of the trial, all data and records will be electronically retained until the youngest child participating in the trial reaches 21 or for five years following completion of the research, whichever is longer.

Future use of data and/or biological samples

The information collected about you/your child may be shared with representatives of the ethics committee to check that the study is being carried out properly and with researchers in other countries without revealing any personal data such as name and address.

The University of Oxford is responsible for ensuring the safe and proper use of any personal information you provide, solely for research purposes.

Right not to participate and withdraw

It is your choice whether you/your child take part in this study and it is entirely voluntary. You/your child can withdraw from the study at any time by contacting the research team. You do not need to give a reason. If you choose to withdraw your child from the study, we will still use the information you gave us before your/your child's withdrawal. We will inform you of any changes to the study.

Communication for Queries, Concerns and Complaints

If you want to know more about our research, you may ask them now or may contact the following investigators at your later suitable time, either meeting with them personally or by contacting them over telephone, at the following addresses:

Dr. Firdausi Qadri

Address of investigator: Enteric and Respiratory Infections, IDD. icddr,b, Mohakhali, Dhaka 1212. Telephone: +880-2-8812529, Extension No. 2431.

If you want to know more about your rights as a research participant you may contact IRB Secretariat, RA, M. A. Salam Khan, phone no: 9886498 or PABX 8860523-32 Extension. 3206 personally at icddr,b or by contact by telephone.

Thank you for your co-operaton.

If you agree to our proposal of enrolling you/your patient in our study, please indicate that by putting your signature or your left thumb impression at the specified space below.

Indicating consent

The investigators/ study staff has explained to me and I have fully understood the purpose, procedures, risks and benefits of participating in this study, my child's rights as a research participant and the confidential handling of the information and records. I understand that I may withdraw my child from the study at any time without giving any reason. I understand that I would also be able to get further information in future and that my child's name and/or identity will not be used in the analysis of data and in sharing the results with others.

Based on the above, I have voluntarily agreed to enrol my child for vaccination
(Please place a tick in the box, if you agree) ☐

Based on the above, I have voluntarily agreed to provide follow-up information
(Please place a tick in the box, if you agree) ☐

I agree to be contacted after the end of this study by the research team ☐

(Please place a tick in the box, if you agree)

Assent of minors (for children aged 11 years - <16 years):

Name of participant: ____

Signature or left thumbprint of participant:__________________________ Date ___________

Name of Father/Mother /guardian of a minor participant _____________________

Signature or left thumbprint of Father/Mother/guardian: ____________________ Date ___________

Name of witness:____

Signature of witness: ____________________________ Date ___________

Name of the personnel securing consent: ____________________________________

Signature of the personnel securing consent:___________ Date ___________

Note- A signed and dated copy/photocopy of this document will be given to you

Appendix A4 (i)
Consent Form for Passive Surveillance (Day 1 - <11years)

Protocol No. PR-17115	Version No. 7.00	Date: 04-02-2019

Protocol Title: Assessing the impact of a Vi-Polysaccharide Conjugate Vaccine in preventing typhoid infection among Bangladeshi children – a Phase IIIb trial.

Investigator's name: Dr Firdausi Qadri, Principal Investigator (PI) of the protocol

Organization: icddr,b

Purpose of the research

Background
Typhoid fever is a serious infectious disease transmitted by taking water and food contaminated with organism. The disease is common in the developing countries including Bangladesh, where the sanitation and pure water supply are not adequate. Now the disease is more commonly found in children, and immediate and appropriate treatment is needed to prevent complication of the patients. Commercially available vaccines against typhoid fever are not so effective in children. The Vi-Polysaccharide typhoid Conjugate Vaccine (Vi-TCV) is the most promising vaccine for controlling typhoid fever. It is important to study the effectiveness of the Vi-TCV vaccineimmunizing Bangladeshi children and to inform the decision makers for introduction of Vi-TCV into the routine expanded programme on immunisation (EPI) in Bangladesh.
The aim of the overall study is to evaluate the effectiveness of the typhoid vaccine.

Efficacy of the Vi-TCV vaccine will be evaluated with the Japanese encephalitis vaccine (SA14-14-2) in a participant- and observer-blinded, cluster-randomized trial design in this study. So, we have vaccinated the children aged 9 month to <16 years in the study area of Mirpur.
To determine the relative and absolute rate reduction of typhoid fever in recipients of Vi-TCV (total vaccine protection), we are inviting all the residents in the study area including the vaccinated participants, who has complaint of fever for ≥2 days and/or presenting temperature of ≥38˚C to participate in the study (at least 32,500).

Reasons for inviting the specific person to participate in the study

You are invited to take part in the study as you/your child have complaint of fever which is more likely to be typhoid fever, so we are inviting you to participate in the study and help us in our efforts.

Roles of the participants in this study

If you agree to our proposal to participate in this aspect of the study, following steps will be done:

At 1st visit (day 0):

- We will collect detailed history of your/your child's illness, perform thorough physical

examination, and record the findings.

- We will collect 6 ml of blood for carrying out the blood culture for diagnosis of typhoid fever and other tests.
- After getting the report of blood culture, we will inform you the test result at the earliest possible time. You/your child will receive the standard care and treatment if the test result becomes positive for typhoid fever.

Follow-up visits (2 weeks and 4 **weeks after illness**):
We will ask you/your child to visit the nearby study health care facility at two weeks after enrolment. If the disease is still unresolved at two weeks, we will ask you/your child for further follow-up at two weeks after previous follow-up; four weeks after the initial presentation.

Genetic test

With your permission, we would like to carry out genetic tests on the samples to look at why some people get typhoid and others do not.

Risk and benefit

You/your child will get momentary pain during collection of blood from a vein, and there is the possibility of temporary discoloration of the skin surrounding the needle prick due to minor bleeding and rare chance of infection at the site of bleeding. We will use disposable syringe and needle and also other precautions to avoid such risks. In the event you/your child develop such a problem, we will provide their appropriate treatment at our cost.
You/your child will have access to free and accurate health assessments and diagnostics at the icddr,b hospitals, as well as in other trial health facilities set up in the community, for all episodes of suspected typhoid fever occurring during the study.

Principle of compensation

The participants shall also be provided best possible, free treatment, for research related injuries. You will not have to pay to us, and we will also not pay to you for participating in the study. However, we will only pay taka 500 to support the conveyance cost for follow up visit if needed.

Privacy, anonymity and confidentiality
All information that we collect during the study is strictly private and confidential. You/your child will be identified by a number. Samples will also not be labeled using any personal information such as names, rather with numbers. Your/your child's name and your address will not be revealed in any output from this study.

Storage of data and biological samples

We will store all leftover specimens after the test performed, if any, and may use them for future research, but they will not carry your/your child's name or identity. All samples will be kept for a minimum of five years after the end of the trial. You may refuse to store your/your child's leftover samples and without this otherwise influencing participation in the study or the clinical care of you/your child. However, we would appreciate your consent as results of such study might help the society.

We will store your/your child's personal information and all laboratory test results in password protected computers and printed copies in locked file cabinet. At the end of the trial, all data and records will be electronically retained until the youngest child participating in the trial reaches 21 or for five years following completion of the research, whichever is longer.

Future use of data and/or biological samples

Stored samples may be used in future research; some of the specimens will be shipped abroad for further analysis. Before using such unnamed samples for any future study, we would first obtain permission from the Ethical Review Committee (ERC) of icddr,b. We would like to take your permission to use these unnamed samples in the future. Anonymous information and data may be supplied to other researchers, which will not conflict with or violate the maintenance of privacy, anonymity and confidentiality of information.

Microbiological, immunological, molecular and genetic assays will be done in laboratory in Dhaka and in other foreign laboratories (Oxford Vaccine Group, Department of Paediatrics, University of Oxford, United Kingdom; University of Melbourne, Australia; Wellcome Trust Sanger Institute, United Kingdom)

The University of Oxford is responsible for ensuring the safe and proper use of any personal information you provide, solely for research purposes.

Right not to participate and withdraw

It is your choice whether you/your child take part in this study and it is entirely voluntary.
You/your child may withdraw from the study at any time by contacting the research team. You do not need to give a reason. If you/your child choose to withdraw from the study, we will still use the information you gave us before withdrawal. We will inform you of any changes to the study.

Communication for Queries, Concerns and Complaints

We will be happy to provide you further information about the study any time. If you want to know more about the research, you may ask now or may contact the following investigators at your later suitable time, either meeting with them personally or by contacting them over telephone, at the following addresses:

Dr. Firdausi Qadri
Address of investigator: Enteric and Respiratory Infections, IDD. icddr,b, Mohakhali, Dhaka 1212. Telephone: +880-2-8812529, Extension No. 2431.

If you want to know more about your rights as a research participant you may contact IRB Secretariat, RA, M. A. Salam Khan, phone no: 9827084 or PABX 8860523-32 Ext. 3206 personally at icddr,b or by contact by telephone.

Thank you for your co-operaton.

If you agree to our proposal of enrolling you in the study, please indicate that by putting your signature or your left thumb impression at the specified space below

Indicating consent

The investigators/ study staff has explained to me and I have fully understood the purpose, procedures, risks and benefits of participating in this study, my/my child's rights as a research participant and the confidential handling of the information and records. I understand that I may withdraw myself/my child from the study at any time without giving any reason. I understand that I would also be able to get further information in future and that my/my child's name and/or identity will not be used in the analysis of data and in sharing the results with others.

Based on the above, I have voluntarily agreed to enrol myself/ my child in this research study (Please place a tick in the box, if you agree) ☐

I give permission for information about my child's illness to be recorded ☐

I give permission to store and use my child's leftover specimens, including DNA, with the understanding that the samples will not bear the name of my/my child's identity ☐

I am also giving permission to store and use leftover specimens with the understanding that the samples will not bear my/my child's name &identity ☐

I agree to be contacted after the end of this study by the research team
(Please place a tick in the box, if you agree) ☐

Participant's age: Day 1 - <11 years

Name of participant: ____

Name of Father/Mother /guardian of a minor participant ______________________

Signature or left thumbprint of Father/Mother/guardian:________________________ Date ____________

Name of witness:____

Signature of witness: _____________________________ Date ___________

Name of the personnel securing consent: _______________________________________

Signature of the personnel securing consent: ____________Date ___________

Note- A signed and dated copy/photocopy of this document will be given to you

Appendix A4 (ii)
Consent Form for Passive Surveillance (11 years - <18 years)

Protocol No. PR-17115	Version No. 7.00	Date: 04-02-2019

Protocol Title: Assessing the impact of a Vi-Polysaccharide Conjugate Vaccine in preventing typhoid infection among Bangladeshi children – a Phase IIIb trial.

Investigator's name: Dr Firdausi Qadri, Principal Investigator (PI) of the protocol

Organization: icddr,b
Purpose of the research

Background
Typhoid fever is a serious infectious disease transmitted by taking water and food contaminated with organism. The disease is common in the developing countries including Bangladesh, where the sanitation and pure water supply are not adequate. Now the disease is more commonly found in children, and immediate and appropriate treatment is needed to prevent complication of the patients. Commercially available vaccines against typhoid fever are not so effective in children. The Vi-Polysaccharide typhoid Conjugate Vaccine (Vi-TCV) is the most promising vaccine for controlling typhoid fever. It is important to study the effectiveness of the Vi-TCV vaccine by immunizing Bangladeshi children and to inform the decision makers for introduction of Vi-TCV into the routine expanded programme on immunisation (EPI) in Bangladesh.
The aim of the overall study is to evaluate the effectiveness of the typhoid vaccine.

Efficacy of the Vi-TCV vaccine will be evaluated with the Japanese encephalitis vaccine (SA14-14-2) in a participant- and observer-blinded, cluster-randomized trial design in this study. So, we have vaccinated the children aged 9 month to <16 years in the study area of Mirpur.
To determine the relative and absolute rate reduction of typhoid fever in recipients of Vi-TCV (total vaccine protection), we are inviting all the residents in the study area including the vaccinated participants, who has complaint of fever for ≥2 days and/or presenting temperature of ≥38˚C to participate in the study (at least 32,500)

Reasons for inviting the specific person to participate in the study

You are invited to take part in the study as you/your child have complaint of fever which is more likely to be typhoid fever, so we are inviting you to participate in the study and help us in our efforts.

Roles of the participants in this study

If you agree to our proposal to participate in this aspect of the study, following steps will be done:

At 1st visit (day 0):

- We will collect detailed history of your/your child's illness, perform thorough physical examination, and record the findings.

- We will collect following amount of blood for carrying out the blood culture for diagnosis of typhoid fever and other tests.
    - ≤16 years: 6ml
    - >16 years: 10 ml
- After getting the report of blood culture, we will inform you the test result at the earliest possible time. You/your child will receive the standard care and treatment if the test result becomes positive for typhoid fever.

Follow-up visits (2 weeks and 4 weeks after illness):
We will ask you/your child to visit the nearby study health care facility at two weeks after enrolment. If the disease is still unresolved at two weeks, we will ask you/your child for further follow-up at two weeks after previous follow-up; four weeks after the initial presentation.

Genetic test

With your permission we would like to carry out genetic tests on the samples to look at why some people get typhoid and others do not.

Risk and benefit

You/your child will get momentary pain during collection of blood from a vein, and there is the possibility of temporary discoloration of the skin surrounding the needle prick due to minor bleeding and rare chance of infection at the site of bleeding. We will use disposable syringe and needle and also other precautions to avoid such risks. In the event you/your child develop such a problem, we will provide their appropriate treatment at our cost.
You/your child will have access to free and accurate health assessments and diagnostics at the icddr,b hospitals, as well as in other trial health facilities set up in the community, for all episodes of suspected typhoid fever occurring during the study.

Principle of compensation

The participants shall also be provided best possible, free treatment, for research related injuries. You will not have to pay to us, and we will also not pay to you for participating in the study. However, we will only pay taka 500 to support the conveyance cost for follow up visit if needed.

Privacy, anonymity and confidentiality
All information that we collect during the study is strictly private and confidential. You/your child will be identified by a number. Samples will also not be labeled using any personal information such as names, rather with numbers. Your/your child’s name and your address will not be revealed in any output from this study.

Storage of data and biological samples

We will store all leftover specimens after the test performed, if any, and may use them for future research, but they will not carry your/your child's name or identity. All samples will be kept for a minimum of five years after the end of the trial. You may refuse to store your/your child's leftover samples and without this otherwise influencing participation in the study or the clinical care of you/your child. However, we would appreciate your consent as results of such study might help the society.

We will store your/your child's personal information and all laboratory test results in password protected computers and printed copies in locked file cabinet. At the end of the trial, all data and records will be electronically retained until the youngest child participating in the trial reaches 21 or for five years following completion of the research, whichever is longer.

Future use of data and/or biological samples

Stored samples may be used in future research; some of the specimens will be shipped abroad for further analysis. Before using such unnamed samples for any future study, we would first obtain permission from the Ethical Review Committee (ERC) of icddr,b. We would like to take your permission to use these unnamed samples in the future. Anonymous information and data may be supplied to other researchers, which will not conflict with or violate the maintenance of privacy, anonymity and confidentiality of information.

Microbiological, immunological, molecular and genetic assays will be done in laboratory in Dhaka and in other foreign laboratories (Oxford Vaccine Group, Department of Paediatrics, University of Oxford, United Kingdom; University of Melbourne, Australia; Wellcome Trust Sanger Institute, United Kingdom).

The University of Oxford is responsible for ensuring the safe and proper use of any personal information you provide, solely for research purposes.

Right not to participate and withdraw

It is your choice whether you/your child take part in this study and it is entirely voluntary.
You/your child may withdraw from the study at any time by contacting the research team. You do not need to give a reason. If you/your child choose to withdraw from the study, we will still use the information you gave us before withdrawal. We will inform you of any changes to the study.

Communication for Queries, Concerns and Complaints

We will be happy to provide you further information about the study any time. If you want to know more about the research, you may ask now or may contact the following investigators at your later suitable time, either meeting with them personally or by contacting them over telephone, at the following addresses:

Dr. Firdausi Qadri
Address of investigator: Enteric and Respiratory Infections, IDD. icddr,b, Mohakhali, Dhaka 1212. Telephone: +880-2-8812529, Extension No. 2431.

If you want to know more about your rights as a research participant you may contact IRB Secretariat, RA, M. A. Salam Khan, phone no: 9827084 or PABX 8860523-32 Ext. 3206 personally at icddr,b or by contact by telephone.

Thank you for your co-operaton.

If you agree to our proposal of enrolling you in the study, please indicate that by putting your signature or your left thumb impression at the specified space below

Indicating consent

The investigators/ study staff has explained to me and I have fully understood the purpose, procedures, risks and benefits of participating in this study, my/my child's rights as a research participant and the confidential handling of the information and records. I understand that I may withdraw myself/my child from the study at any time without giving any reason. I understand that I would also be able to get further information in future and that my/my child's name and/or identity will not be used in the analysis of data and in sharing the results with others.

Based on the above, I have voluntarily agreed to enrol myself/ my child in this research study
(Please place a tick in the box, if you agree) ☐

I give permission for information about my child's illness to be recorded ☐

I give permission to store and use my child's leftover specimens, including DNA, with the understanding that the samples will not bear the name of my/my child's identity ☐

I am also giving permission to store and use leftover specimens with the understanding that the samples will not bear my/my child's name &identity ☐

I agree to be contacted after the end of this study by the research team
(Please place a tick in the box, if you agree) ☐

Assent of minors (for children aged 11 years - <18 years):

Name of participant: ____

Signature or left thumbprint of participant:___________________________ Date ___________

Name of Father/Mother /guardian of a minor participant ______________________

Signature or left thumbprint of Father/Mother/guardian: _____________________ Date ___________

Name of witness:____

Signature of witness: ______________________________ Date ___________

Name of the personnel securing consent: ____

Signature of the personnel securing consent: ____________Date ___________

Note- A signed and dated copy/photocopy of this document will be given to you

Appendix A4 (iii)
Consent Form for Passive Surveillance (18 years and above)

Protocol No. PR-17115	Version No. 7.00	Date: 04-02-2019

Protocol Title: Assessing the impact of a Vi-Polysaccharide Conjugate Vaccine in preventing typhoid infection among Bangladeshi children – a Phase IIIb trial.

Investigator's name: Dr Firdausi Qadri, Principal Investigator (PI) of the protocol

Organization: icddr,b

Purpose of the research

Background
Typhoid fever is a serious infectious disease transmitted by taking water and food contaminated with organism. The disease is common in the developing countries including Bangladesh, where the sanitation and pure water supply are not adequate. Now the disease is more commonly found in children, and immediate and appropriate treatment is needed to prevent complication of the patients. Commercially available vaccines against typhoid fever are not so effective in children. The Vi-Polysaccharide typhoid Conjugate Vaccine (Vi-TCV) is the most promising vaccine for controlling typhoid fever. It is important to study the effectiveness of the Vi-TCV vaccine by immunizing Bangladeshi children and to inform the decision makers for introduction of Vi-TCV into the routine expanded programme on immunisation (EPI) in Bangladesh.
The aim of the overall study is to evaluate the effectiveness of the typhoid vaccine. Efficacy of the Vi-TCV vaccine will be evaluated with the Japanese encephalitis vaccine (SA14-14-2) in a participant- and observer-blinded, cluster-randomized trial design in this study. So, we have vaccinated the children aged 9 month to <16 years in the study area of Mirpur.
To determine the relative and absolute rate reduction of typhoid fever in recipients of Vi-TCV (total vaccine protection), we are inviting all the residents in the study area including the vaccinated participants, who has complaint of fever for ≥2 days and/or presenting temperature of ≥38˚C to participate in the study

Reasons for inviting the specific person to participate in the study
You are invited to take part in the study as you/your child have complaint of fever which is more likely to be typhoid fever, so we are inviting you to participate in the study and help us in our efforts.

Roles of the participants in this study

If you agree to our proposal to participate in this aspect of the study, following steps will be done:

At 1st visit (day 0):

- We will collect detailed history of your/your child's illness, perform thorough physical examination, and record the findings.

- We will collect 10 ml of blood for carrying out the blood culture for diagnosis of typhoid fever and other tests.
- After getting the report of blood culture, we will inform you the test result at the earliest possible time. You/your child will receive the standard care and treatment if the test result becomes positive for typhoid fever.

Follow-up visits (2 weeks and 4 weeks after illness):
We will ask you/your child to visit the nearby study health care facility at two weeks after enrolment. If the disease is still unresolved at two weeks, we will ask you/your child for further follow-up at two weeks after previous follow-up; four weeks after the initial presentation.

Genetic test

With your permission we would like to carry out genetic tests on the samples to look at why some people get typhoid and others do not.

Risk and benefit

You/your child will get momentary pain during collection of blood from a vein, and there is the possibility of temporary discoloration of the skin surrounding the needle prick due to minor bleeding and rare chance of infection at the site of bleeding. We will use disposable syringe and needle and also other precautions to avoid such risks. In the event you/your child develop such a problem, we will provide their appropriate treatment at our cost.
You/your child will have access to free and accurate health assessments and diagnostics at the icddr,b hospitals, as well as in other trial health facilities set up in the community, for all episodes of suspected typhoid fever occurring during the study.

Principle of compensation

The participants shall also be provided best possible, free treatment, for research related injuries. You will not have to pay to us, and we will also not pay to you for participating in the study. However, we will only pay taka 500 to support the conveyance cost for follow up visit if needed.

Privacy, anonymity and confidentiality
All information that we collect during the study is strictly private and confidential. You/your child will be identified by a number. Samples will also not be labeled using any personal information such as names, rather with numbers. Your/your child's name and your address will not be revealed in any output from this study.

Storage of data and biological samples

We will store all leftover specimens after the test performed, if any, and may use them for future research, but they will not carry your/your child's name or identity.

All samples will be kept for a minimum of five years after the end of the trial. You may refuse to store your/your child's leftover samples and without this otherwise influencing participation in the study or the clinical care of you/your child. However, we would appreciate your consent as results of such study might help the society.

We will store your/your child's personal information and all laboratory test results in password protected computers and printed copies in locked file cabinet. At the end of the trial, all data and records will be electronically retained until the youngest child participating in the trial reaches 21 or for five years following completion of the research, whichever is longer.

Future use of data and/or biological samples

Stored samples may be used in future research; some of the specimens will be shipped abroad for further analysis. Before using such unnamed samples for any future study, we would first obtain permission from the Ethical Review Committee (ERC) of icddr,b. We would like to take your permission to use these unnamed samples in the future. Anonymous information and data may be supplied to other researchers, which will not conflict with or violate the maintenance of privacy, anonymity and confidentiality of information.

Microbiological, immunological, molecular and genetic assays will be done in laboratory in Dhaka and in other foreign laboratories (Oxford Vaccine Group, Department of Paediatrics, University of Oxford, United Kingdom; University of Melbourne, Australia; Wellcome Trust Sanger Institute, United Kingdom)

The University of Oxford is responsible for ensuring the safe and proper use of any personal information you provide, solely for research purposes.

Right not to participate and withdraw

It is your choice whether you/your child take part in this study and it is entirely voluntary.

You/your child may withdraw from the study at any time by contacting the research team. You do not need to give a reason. If you/your child choose to withdraw from the study, we will still use the information you gave us before withdrawal. We will inform you of any changes to the study.

Communication for Queries, Concerns and Complaints

We will be happy to provide you further information about the study any time. If you want to know more about the research, you may ask now or may contact the following investigators at your later suitable time, either meeting with them personally or by contacting them over telephone, at the following addresses:

Dr. Firdausi Qadri

Address of investigator: Enteric and Respiratory Infections, IDD. icddr,b, Mohakhali, Dhaka 1212. Telephone: +880-2-8812529, Extension No. 2431.

If you want to know more about your rights as a research participant you may contact IRB Secretariat, RA, M. A. Salam Khan, phone no: 9827084 or PABX 8860523-32 Ext. 3206 personally at icddr,b or by contact by telephone.

Thank you for your co-operaton.

If you agree to our proposal of enrolling you in the study, please indicate that by putting your signature or your left thumb impression at the specified space below

Indicating consent

The investigators/ study staff has explained to me and I have fully understood the purpose, procedures, risks and benefits of participating in this study, my/my child's rights as a research participant and the confidential handling of the information and records. I understand that I may withdraw myself/my child from the study at any time without giving any reason. I understand that I would also be able to get further information in future and that my/my child's name and/or identity will not be used in the analysis of data and in sharing the results with others.

Based on the above, I have voluntarily agreed to enrol myself/ my child in this research study (Please place a tick in the box, if you agree) ☐

I give permission for information about my illness to be recorded ☐

I give permission to store and use my leftover specimens, including DNA, with the understanding that the samples will not bear the name of my identity ☐

I am also giving permission to store and use leftover specimens with the understanding that the samples will not bear my/my child's name &identity ☐

I agree to be contacted after the end of this study by the research team (Please place a tick in the box, if you agree) ☐

Participant's age: 18 Years and Above

Name of participant: ____

Signature or left thumbprint of participant:__________________________ Date ___________

Name of witness:____

Signature of witness: ____________________________ Date ___________

Name of the personnel securing consent: _____________________________________

Signature of the personnel securing consent: ____________Date ___________

Note- A signed and dated copy/photocopy of this document will be given to you

Appendix A5 (i)
Consent form for Immunogenicity Study (9months - < 11years)

Protocol No. PR-17115	Version No. 8.00	Date: 10-12-2020

Protocol Title: Assessing the impact of a Vi-Polysaccharide Conjugate Vaccine in preventing typhoid infection among Bangladeshi children – a Phase IIIb trial.

Investigator's name: Dr Firdausi Qadri, Principal Investigator (PI) of the protocol

Organization: icddr,b

Purpose of the research:

Background

Typhoid fever is a serious infectious disease transmitted by taking water and food contaminated with organism. The disease is common in the developing countries including Bangladesh, where the sanitation and pure water supply are not adequate. Now the disease is more commonly found in children, and immediate and appropriate treatment is needed to prevent complication of the patients. Commercially available vaccines against typhoid fever are not so effective in children. The Vi-Polysaccharide typhoid Conjugate Vaccine (Vi-TCV) is the most promising vaccine for controlling typhoid fever. It is important to study the effectiveness of the Vi-TCV vaccine by immunizing Bangladeshi children and to inform the decision makers for introduction of Vi-TCV into the routine expanded programme on immunisation (EPI) in Bangladesh.

The aim of the overall study is to evaluate the effectiveness of the typhoid vaccine.

In this study, the safety and immunogenicity of Vi-TCV vaccine will be evaluated with Japanese encephalitis vaccine (SA14-14-2) in a participant- and observer-blinded, cluster-randomized trial design.

Reasons for inviting the specific person to participate in the study

You are invited to participate in the study, because your child is aged of 9 months to <16 years and has agreed to receive the vaccine. A subset of approximately 1500 vaccinated children will be randomly selected for the immunogenicity study to test the immune response induced in the vaccinees. Since your child has been selected for this study, we are inviting your child to participate in this study.

Roles of the participants in this study

If you agree to our proposal to include your child in our study, then the following steps would be done:

1. Day of vaccination (Day 0):

- We will examine your child to make sure he/she is healthy. If your child is a married girl, we will collect a sample of urine for pregnancy test before vaccination. If your child meets all eligibility criteria, we will collect 3-5ml blood (upto one teaspoon) from your child. If your child is under 5 years of age we will collect maximum of 3 ml and if your child is within 5 to <11 years of age we will collect maximum of 5 ml blood.

- Then your child will get Vi-TCV or SA14-14-2 vaccine. However, we do not want to identify people who are receiving the typhoid vaccine or the control Japanese encephalitis vaccine. This will be done by a process called "randomization" which is like flipping a coin, where all participants will have equal chance to receive typhoid or control vaccine.
- You will be asked to bring your child for three additional face-to-face follow-up visits at Mirpur Field clinic after receiving vaccine, aside from regular follow-up schedule of vaccination as stated in the vaccination consent form.

2. Follow-up (at Day 28+/-4 days, Day 545+/-56 days and Day 730+/-90 days) visits:

- You'll be asked for oral confirmation of continued participation in the study.
- We will collect 3-5ml blood (upto one teaspoon) from your child at each visit. If your child is under 5 years of age we will collect maximum of 3 ml and if your child is within 5 to <11 years of age we will collect maximum of 5 ml blood.
- Confirmation of contact details will be sought and instructions to attend the health care facilities in the case of a fever for ≥2 days and/or presenting temperature ≥ 38°C
- All information relevant to study from each visit will be recorded in the participant's CRF.

3. Follow-up (at Day 365 +/-90 days, and Day 730+/-90 days post unblinding) visits:

- You'll be asked for oral confirmation of continued participation in the study.
- We will collect 3-5ml blood (upto one teaspoon) from your child at each visit. If your child is under 5 years of age we will collect maximum of 3 ml and if your child is within 5 to <11 years of age we will collect maximum of 5 ml blood.
- Confirmation of contact details will be sought and instructions to attend the health care facilities in the case of a fever for ≥2 days and/or presenting temperature ≥ 38°C
- All information relevant to study from each visit will be recorded in the participant's CRF.

Genetic test

With your permission we would like to carry out genetic tests on the samples to look at vaccine response and why some people get typhoid and others do not.

Risk and benefit

There is no routine vaccination for typhoid or Japanese encephalitis in Bangladesh. All participants enrolled will have the benefit of receiving either Vi-TCV, which will likely provide additional protection against typhoid, or Japanese encephalitis, which is endemic in Bangladesh.

All participants will have access to free and accurate health assessments and diagnostics at the icddr,b hospitals, as well as in other trial health facilities set up in the community, for all episodes of suspected typhoid fever occurring during the study. Whilst participants would not necessarily benefit directly from this, a positive result from this trial could lead to implementation of a Vi-TCV vaccine programme in typhoid endemic areas, including Bangladesh.

Different studies have shown that the Vi-TCV vaccine is safe and well tolerated. This, however, is the first controlled study involving so many participants and there may be rare adverse events that may be identified in this study. We will be conducting safety monitoring as a secondary outcome of this study.

You will get momentary pain during collection of blood from a vein, and there is the possibility of temporary discoloration of the skin surrounding the needle prick due to minor bleeding and rare chance of infection at the site of bleeding. We will use disposable syringe and needle and also other precautions to avoid such risks. In the event you develop any such problem, we will provide their appropriate treatment at our costs.

Principle of compensation

The participants shall also be provided best possible, free treatment, for research related injuries. You will not have to pay to us, and we will also not pay to you for participating in the study. However, we will only pay taka 500 to support the conveyance cost for each visit.

Privacy, anonymity and confidentiality

All information that we collect during the study is strictly private and confidential. You/your child will be identified by a number. Samples will also not be labeled using any personal information such as names, rather with numbers. Your/your child's name and your address will not be revealed in any output from this study.

Storage of data and biological samples

We will store all leftover specimens after the test performed, if any, and may use them for future research, but they will not carry your child's name or identity.

All samples will be kept for a minimum of five years after the end of the trial. You may refuse to store your child's leftover samples and without this otherwise influencing participation in the study or the clinical care of your child. However, we would appreciate your consent as results of such study might help the society.

We will store your personal information and all laboratory test results in password protected computers and printed copies in locked file cabinet. At the end of the trial, all data and records will be electronically retained until the youngest child participating in the trial reaches 21 or for five years following completion of the research, whichever is longer.

Future use of data and/or biological samples

Stored samples (including DNA) may be used in future research; some of the specimens will be shipped abroad for further analysis. Before using such unnamed samples for any future study, we

would first obtain permission from the Ethical Review Committee (ERC) of icddr,b. We would like to take your permission to use these unnamed samples in the future. Anonymous information and data may be supplied to other researchers, which will not conflict with or violate the maintenance of privacy, anonymity and confidentiality of information.

Microbiological, immunological, molecular and genetic assays will be done in laboratory in Dhaka and in other foreign laboratories (Oxford Vaccine Group, Department of Paediatrics, University of Oxford, United Kingdom; University of Melbourne, Australia; Wellcome Trust Sanger Institute, United Kingdom)

The University of Oxford is responsible for ensuring the safe and proper use of any personal information you provide, solely for research purposes.

Right not to participate and withdraw

It is your choice whether your child takes part in this study and it is entirely voluntary.
Your child can withdraw from the study at any time by contacting the research team (details below). You do not need to give a reason. If your child chooses to withdraw from the study, we will still use the information you gave us before your child's withdrawal. We will inform you of any changes to the study.

Communication for Queries, Concerns and Complaints

We will be happy to provide you further information about the study any time. If you want to know more about the research, you may ask now or may contact the following investigators at your later suitable time, either meeting with them personally or by contacting them over telephone, at the following addresses:

Dr. Firdausi Qadri
Address of investigator: Enteric and Respiratory Infections, IDD. icddr,b, Mohakhali, Dhaka 1212. Telephone: +880-2-8812529, Extension No. 2431.
If you want to know more about your rights as a research participant you may contact IRB Secretariat, RA, M. A. Salam Khan, phone no: 9827084 or PABX 8860523-32 Ext. 3206 personally at icddr,b or by contact by telephone.
Thank you for your co-operation.
If you agree to our proposal of enrolling you in the study, please indicate that by putting your signature or your left thumb impression at the specified space below

Indicating consent

The investigators/ study staff has explained to me and I have fully understood the purpose, procedures, risks and benefits of participating in this study, my child's rights as a research participant and the confidential handling of the information and records. I understand that I may withdraw my child from the study at any time without giving any reason. I understand that I would also be able to get further information in future and that my child's name and/or identity will not be used in the analysis of data and in sharing the results with others.

Based on the above, I have voluntarily agreed to enrol my child in this research study (Please place a tick in the box, if you agree) ☐

I give permission to store my child's DNA samples and use them in future research with the understanding that the samples will not bear the name of my child's identity ☐

I am also giving permission to store my child's blood samples and use them in future research with the understanding that the samples will not bear the name of my child's identity ☐

I agree to be contacted after the end of this study by the research team ☐

Participant's age: 9 months - <11 years

Name of participant: ____

Name of Mother/ Father/guardian of a minor participant ______________________

Signature or left thumbprint of Father/Mother/guardian:___________________Date ____________

Name of witness:____

Signature of witness: ______________________________ Date ____________

Name of the personnel securing consent: ____

Signature of the personnel securing consent: _____________Date ____________

Note- A signed and dated copy/photocopy of this document will be given to you

Appendix A5 (ii)
Consent form for Immunogenicity Study (11 years - <16 years)

Protocol No. PR-17115	Version No. 9.00	Date: 10-12-2020

Protocol Title: Assessing the impact of a Vi-Polysaccharide Conjugate Vaccine in preventing typhoid infection among Bangladeshi children – a Phase IIIb trial.

Investigator's name: Dr Firdausi Qadri, Principal Investigator (PI) of the protocol

Organization: icddr,b

Purpose of the research:

Background

Typhoid fever is a serious infectious disease transmitted by taking water and food contaminated with organism. The disease is common in the developing countries including Bangladesh, where the sanitation and pure water supply are not adequate. Now the disease is more commonly found in children, and immediate and appropriate treatment is needed to prevent complication of the patients. Commercially available vaccines against typhoid fever are not so effective in children. The Vi-Polysaccharide typhoid Conjugate Vaccine (Vi-TCV) is the most promising vaccine for controlling typhoid fever. It is important to study the effectiveness of the Vi-TCV vaccine by immunizing Bangladeshi children and to inform the decision makers for introduction of Vi-TCV into the routine expanded programme on immunisation (EPI) in Bangladesh.
The aim of the overall study is to evaluate the effectiveness of the typhoid vaccine.

In this study, the safety and immunogenicity of Vi-TCV vaccine will be evaluated with Japanese encephalitis vaccine (SA14-14-2) in a participant- and observer-blinded, cluster-randomized trial design.

Reasons for inviting the specific person to participate in the study

You are invited to participate in the study, because your child is aged of 9 months to <16 years and has agreed to receive the vaccine. A subset of approximately1500 vaccinated children will be randomly selected for the immunogenicity study to test the immune response induced in the vaccinees. Since your child has been selected for this study, we are inviting your child to participate in this study.

Roles of the participants in this study

If you agree to our proposal to include your child in our study, then the following steps would be done:

1. Day of vaccination (Day 0):

- We will examine your child to make sure he/she is healthy. If your child is a married girl, we will collect a sample of urine for pregnancy test before vaccination. If your child meets all eligibility criteria, we will collect 3-5ml blood (upto one teaspoon) from your child. If

your child is under 5 years of age we will collect maximum of 3 ml and if your child is within 5 to <16 years of age we will collect maximum of 5 ml blood.

- Then your child will get Vi-TCV or SA14-14-2 vaccine. However, we do not want to identify people who are receiving the typhoid vaccine or the control Japanese encephalitis vaccine. This will be done by a process called "randomization" which is like flipping a coin, where all participants will have equal chance to receive typhoid or control vaccine.
- You will be asked to bring your child for three additional face-to-face follow-up visits at Mirpur Field clinic after receiving vaccine, aside from regular follow-up schedule of vaccination as stated in the vaccination consent form.

2. Follow-up (at Day 28+/-4 days, Day 545+/-56 days and Day 730+/-90 days) visits:

- You'll be asked for oral confirmation of continued participation in the study.
- We will collect maximum of 5 ml blood from your child.
- Confirmation of contact details will be sought and instructions to attend the health care facilities in the case of a fever for ≥2 days and/or presenting temperature ≥ 38° C
- All information relevant to study from each visit will be recorded in the participant's CRF.

3. Follow-up (at Day 365 +/-90 days, and Day 730+/-90 days post unblinding) visits:

- You'll be asked for oral confirmation of continued participation in the study.
- We will collect 3-5ml blood (upto one teaspoon) from your child at each visit. If your child is under 5 years of age we will collect maximum of 3 ml and if your child is within 5 to <11 years of age we will collect maximum of 5 ml blood.
- Confirmation of contact details will be sought and instructions to attend the health care facilities in the case of a fever for ≥2 days and/or presenting temperature ≥ 38°C
- All information relevant to study from each visit will be recorded in the participant's CRF.

Genetic test

With your permission we would like to carry out genetic tests on the samples to look at vaccine response and why some people get typhoid and others do not.

Risk and benefit

There is no routine vaccination for typhoid or Japanese encephalitis in Bangladesh. All participants enrolled will have the benefit of receiving either Vi-TCV, which will likely provide additional protection against typhoid, or Japanese encephalitis, which is endemic in Bangladesh.

All participants will have access to free and accurate health assessments and diagnostics at the icddr,b hospitals, as well as in other trial health facilities set up in the community, for all episodes of suspected typhoid fever occurring during the study. Whilst participants would not necessarily

benefit directly from this, a positive result from this trial could lead to implementation of a Vi-TCV vaccine programme in typhoid endemic areas, including Bangladesh.

Different studies have shown that the Vi-TCV vaccine is safe and well tolerated. This, however, is the first controlled study involving so many participants and there may be rare adverse events hereto unidentified that may become apparent in this study. We will be conducting safety monitoring as a secondary outcome of this study.

You will get momentary pain during collection of blood from a vein, and there is the possibility of temporary discoloration of the skin surrounding the needle prick due to minor bleeding and rare chance of infection at the site of bleeding. We will use disposable syringe and needle and also other precautions to avoid such risks. In the event you develop any such problem, we will provide their appropriate treatment at our costs.

Principle of compensation

The participants shall also be provided best possible, free treatment, for research related injuries. You will not have to pay to us, and we will also not pay to you for participating in the study. However, we will only pay taka 500 to support the conveyance cost for each visit.

Privacy, anonymity and confidentiality

All information that we collect during the study is strictly private and confidential. You/your child will be identified by a number. Samples will also not be labeled using any personal information such as names, rather with numbers. Your/your child's name and your address will not be revealed in any output from this study.

Storage of data and biological samples

We will store (including DNA) all leftover specimens after the test performed, if any, and may use them for future research, but they will not carry your child's name or identity.

All samples will be kept for a minimum of five years after the end of the trial. You may refuse to store your child's leftover samples and without this otherwise influencing participation in the study or the clinical care of your child. However, we would appreciate your consent as results of such study might help the society.

We will store your personal information and all laboratory test results in password protected computers and printed copies in locked file cabinet. At the end of the trial, all data and records will be electronically retained until the youngest child participating in the trial reaches 21 or for five years following completion of the research, whichever is longer.

Future use of data and/or biological samples

Stored samples may be used in future research; some of the specimens will be shipped abroad for further analysis. Before using such unnamed samples for any future study, we would first obtain permission from the Ethical Review Committee (ERC) of icddr,b. We would like to take your permission to use these unnamed samples in the future. Anonymous information and data may be

supplied to other researchers, which will not conflict with or violate the maintenance of privacy, anonymity and confidentiality of information.

Microbiological, immunological, molecular and genetic assays will be done in laboratory in Dhaka and in other foreign laboratories (Oxford Vaccine Group, Department of Paediatrics, University of Oxford, United Kingdom; University of Melbourne, Australia; Wellcome Trust Sanger Institute, United Kingdom)

The University of Oxford is responsible for ensuring the safe and proper use of any personal information you provide, solely for research purposes.

Right not to participate and withdraw

It is your choice whether your child takes part in this study and it is entirely voluntary.
Your child can withdraw from the study at any time by contacting the research team (details below). You do not need to give a reason. If your child chooses to withdraw from the study, we will still use the information you gave us before your child's withdrawal. We will inform you of any changes to the study.

Communication for Queries, Concerns and Complaints
We will be happy to provide you further information about the study any time. If you want to know more about the research, you may ask now or may contact the following investigators at your later suitable time, either meeting with them personally or by contacting them over telephone, at the following addresses:

Dr. Firdausi Qadri
Address of investigator: Enteric and Respiratory Infections, IDD. icddr,b, Mohakhali, Dhaka 1212. Telephone: +880-2-8812529, Extension No. 2431.
If you want to know more about your rights as a research participant you may contact IRB Secretariat, RA, M. A. Salam Khan, phone no: 9827084 or PABX 8860523-32 Ext. 3206 personally at icddr,b or by contact by telephone.
Thank you for your co-operation.
If you agree to our proposal of enrolling you in the study, please indicate that by putting your signature or your left thumb impression at the specified space below

Indicating consent

The investigators/ study staff has explained to me and I have fully understood the purpose, procedures, risks and benefits of participating in this study, my child's rights as a research participant and the confidential handling of the information and records. I understand that I may withdraw my child from the study at any time without giving any reason. I understand that I would also be able to get further information in future and that my child's name and/or identity will not be used in the analysis of data and in sharing the results with others.

Based on the above, I have voluntarily agreed to enrol my child in this research study
(Please place a tick in the box, if you agree) ☐

I give permission to store my child's DNA samples and use them in future research with the understanding that the samples will not bear the name of my child's identity ☐

I am also giving permission to store my child's blood samples and use them in future research with the understanding that the samples will not bear the name of my child's identity ☐

I agree to be contacted after the end of this study by the research team ☐

Assent of minors (for children aged 11 years - <16 years):

Name of participant: ____

Signature or left thumbprint of participant:___________________________ Date ___________

Name of Father/Mother/guardian of a minor participant ______________________

Signature or left thumbprint of Father/Mother/guardian: ________________ Date ___________

Name of witness:____

Signature of witness: _____________________________ Date ___________

Name of the personnel securing consent: _______________________________________

Signature of the personnel securing consent: ____________Date ___________

Note- A signed and dated copy/photocopy of this document will be given to you

Appendix A6

Consent Form for Verbal Autopsy

(Relative of Deceased Participant: 18 Years & Above)

**Protocol No. PR-17115**	**Version No. 2.00**	**Date: 27-08-2018**

Protocol Title: Assessing the impact of a Vi-Polysaccharide Conjugate Vaccine in preventing typhoid infection among Bangladeshi children – a Phase IIIb trial.

Investigator's name: Dr Firdausi Qadri, Principal Investigator (PI) of the protocol
Organization: icddr,b
Purpose of the research

Background
Typhoid fever is a serious infectious disease transmitted by taking water and food contaminated with organism. The disease is common in the developing countries including Bangladesh, where the sanitation and pure water supply are not adequate. Now the disease is more commonly found in children, and immediate and appropriate treatment is needed to prevent complication of the patients. Commercially available vaccines against typhoid fever are not so effective in children. The Vi-Polysaccharide typhoid Conjugate Vaccine (Vi-TCV) is the most promising vaccine for controlling typhoid fever. It is important to study the effectiveness of the Vi-TCV vaccine by immunizing Bangladeshi children and to inform the decision makers for introduction of Vi-TCV into the routine expanded programme on immunization (EPI) in Bangladesh.

The aim of the overall study is to evaluate the effectiveness of the typhoid vaccine. Efficacy of the Vi-TCV vaccine will be evaluated with the Japanese encephalitis vaccine (SA14-14-2) in a participant- and observer-blinded, cluster-randomized trial design in this study. So, we have vaccinated the children aged 9 month to <16 yearsin the study areaof Mirpur **.**

Reason for asking for participation in the study

We are very sorry to hear that one of your relatives has passed away. Please accept my sympathies. To know the probable cause of death, we are collecting the information related to death. I would like to invite you to participate in this study as you are the relative / parents of her/his.

Your role in this interview

If you agree to participate in this verbal autopsy interview as a respondent I will ask you some general questions and some questions about the events and symptoms that she/he had during her/his illness before death. More specially, we want to know details about circumstances and events/clinical accounts that led to death. We know that this may not be an easy topic to discuss for you, but I hope that you will tell about your observation, illnesses and circumstances that led to death. The interview will take up to one hour. But you don't have to spend all of the time if you don't want to. We can always comeback at a later time if you think that will be better for you. You can also stop the interview at any time, if you do not wish to continue once we have started. In this regard we are seeking your permission.

Risk and benefits

There is probably no risk to you or any of your family members for taking part in this study. We recognize that there is potential distress which you may experience when discussing the death of your loved-one in detail. Your participation will help us understand the cause of death. There is no direct benefit to you personally for participating in the study. But we will be able to answer some critical research question.

Privacy, anonymity and confidentiality

Your personal/your deceased relatives' information (all medical and results of the laboratory tests) will remain confidential and will be stored in a file cabinet under lock and key. None other than the investigators of this study and project staff directly dealing with you, the Ethical Review Committee and Data Safety and Monitoring Board (these are two committees are responsible for monitoring results and safety of the participants) will be able to see them. However, disclosure of such information is also guided by the laws of Bangladesh. Your name and identity will not be used in analyzing results of the study and/ or in sharing results with others including publication in journals. By signing this consent, you are authorizing granting of such access only to these specific groups mentioned above.

The University of Oxford is responsible for ensuring the safe and proper use of any personal information you provide, solely for research purposes.

Principle of Compensation

No remuneration will neither be offered nor taken for the participation in this study.

Right not to participate and withdraw

You are the one to decide for or against participation in this study. You will also be able to withdraw yourself from the study at any time without giving any cause.

Communication for Queries, Concerns and Complaints

We will be happy to provide you further information about the study any time. If you want to know more about the research, you may ask now or may contact the following investigators at your later suitable time, either meeting with them personally or by contacting them over telephone, at the following addresses:

Dr. Firdausi Qadri
Address of investigator: Enteric and Respiratory Infections, IDD. icddr,b, Mohakhali, Dhaka 1212. Telephone: +880-2-8812529, Extension No. 2431.
If you want to know more about your rights as a research participant you may contact IRB Secretariat, RA, M. A. Salam Khan, phone no: 9827084 or PABX 8860523-32 Ext. 3206 personally at icddr,b or by contact by telephone.
Name of local Ethical Review Committee: icddr,b , Coordination Committee Secretariat
Local Ethical Review Committee contact: Mr. M.A. Salam Khan, Telephone: 9827084

Indicating consent

The investigators/ study staff has explained to me and I have fully understood the purpose, procedures, risks and benefits of participating in this study, my rights as a research participant and the confidential handling of the information and records. I understand that I may withdraw myself from the study at any time without giving any reason. I understand that I would also be able to get further information in future and that my name and/or identity will not be used in the analysis of data and in sharing the results with others. Based on the above, I have voluntarily agreed to enroll myself in this research study.

Based on the above, I have voluntarily agreed to give interview regarding the death of my relative in this research study
(Please place a tick in the box, if you agree) ☐

Name of respondent: ____

Relation with the deceased:_______________________________________

Signature or left thumbprint of respondent:___________________________ Date ___________

Name of witness:____

Signature of witness: _____________________________ Date ___________

Name of the personnel securing consent: _______________________________________

Signature of the personnel securing consent: _______________________Date ___________

Note- A signed and dated copy/photocopy of this document will be given to you

Appendix A 7 (i)
Consent form for recording hospitalization information (Day 1 - <11years)

Protocol No. PR-17115	Version No. 1.00	Date: 27-08-2018

Protocol Title: Assessing the impact of a Vi-Polysaccharide Conjugate Vaccine in preventing typhoid infection among Bangladeshi children – a Phase IIIb trial.

Investigator's name: Dr Firdausi Qadri, Principal Investigator (PI) of the protocol

Organization: icddr,b
Purpose of the research

Background
Typhoid fever is a serious infectious disease transmitted by taking water and food contaminated with a certain type of bacteria. The disease is common in the developing countries including Bangladesh, where the sanitation and pure water supply are not adequate. Now the disease is more commonly found in children, and immediate and appropriate treatment is needed to prevent complication. Commercially available vaccines against typhoid fever are not so effective in children. The Vi-Polysaccharide typhoid Conjugate Vaccine (Vi-TCV) is the most promising vaccine for controlling typhoid fever. It is important to study the effectiveness of the Vi-TCV by immunizing Bangladeshi children. This will help inform decision makers when considering the introduction of Vi-TCV into the routine expanded programme on immunisation (EPI) in Bangladesh.
The aim of the overall study is to evaluate the effectiveness of the typhoid vaccine.

Efficacy of the Vi-TCV vaccine will be evaluated with the Japanese encephalitis vaccine (SA14-14-2) in a participant- and observer-blinded, cluster-randomized trial design in this study. So, we have vaccinated the children aged 9 month to <16 years in the study area of Mirpur.

We are inviting all the residents in the study area who have been hospitalized to provide information on their hospitalization. This will help us identify if there is any difference in how many people get hospitalized or why people get hospitalized, depending on which group of the study they are in.

Reasons for inviting the specific person to participate in the study

You are invited to take part in the study as your household resides in our study area and your child was hospitalized with a fever or another health issues. So we are inviting you to participate in this aspect of our study.

Roles of the participants in this study

If you agree for your child to take part in this aspect of our study, then we would ask you some questions about your child's hospitalization episode i.e. cause and duration of hospital stay, treatment and outcome etc. Our staff will interview you in your home and it will take 15-20

minutes to complete. We will not contact you again about this study component unless your child or a different member of your household another event of hospitalization in the future.

Genetic Test

We will not collect any samples if you agree for your child to participate in this aspect of the study. If your child is involved with other components of the study, we may collect blood sample and ask your permission to carry out genetic test on that sample.

Risk and Benefit

There is no direct benefit to you, your child or other members of your household. However information from this and other linked studies will help the government to make choices about the use of this vaccine to help prevent typhoid fever. It will not cost you anything to take part in this study.

There is no serious risk to your child in taking part in this aspect of the study.

Principal of Compensation

The participants will be provided with the best possible, free treatment for any research related injuries directly associated with this component of the study. You will not have to pay us and we will also not pay you for participating in the study.

Privacy, anonymity and confidentiality

All information that we collect during the study is strictly private and confidential.Your child will be identified by a number. Your/your child's name and address will not be revealed in any output from this study.

Storage of data and biological samples

We will not collect any biological samples if you agree for your child to participate in this aspect of the study. We will store all information collected from you in password protected computers and paper documents will be locked in the file cabinets. At the end of the trial, all data and records will be electronically retained until the youngest child participating in the trial reaches 21 or for 5 years following completion of the research, whichever is longer.

Future use of data and/or biological samples

Stored data may be used in future research; some of the data will be shipped abroad for further analysis. Before using such data for any future study, we would first obtain permission from the Ethical Review Committee (ERC) of icddr,b. We would like to take your permission to use these data in the future. Anonymous information and data may be supplied to other researchers, which will not conflict with or violate the maintenance of privacy, anonymity and confidentiality of information.

The University of Oxford is responsible for ensuring the safe and proper use of any personal information you provide, solely for research purposes.

Right not to participate and withdraw

It is your choice whether you provide information on your child's hospitalization and it is entirely voluntary. You can withdraw your child from the study at any time by contacting the research team. You do not need to give a reason if you choose to withdraw. We will still use the information you gave us before your child's withdrawal. We will inform you of any changes to the study.

Communication for Queries, Concerns and Complaints

We will be happy to provide you further information about the study any time. If you want to know more about the research, you may ask now or may contact the following investigators at your later suitable time, either meeting with them personally or by contacting them over telephone, at the following addresses:

Dr. Firdausi Qadri
Address of investigator: Enteric and Respiratory Infections, IDD. icddr,b, Mohakhali, Dhaka 1212. Telephone: +880-2-8812529, Extension No. 2431.

If you want to know more about your rights as a research participant you may contact IRB Secretariat, RA, M. A. Salam Khan, phone no: 9827084 or PABX 8860523-32 Ext. 3206 personally at icddr,b or by contact by telephone.

Thank you for your co-operaton.

If you agree to our proposal of enrolling you in the study, please indicate that by putting your signature or your left thumb impression at the specified space below

Indicating consent

The investigators/ study staff has explained to me and I have fully understood the purpose, procedures, risks and benefits of participating in this aspect of the study, my/my child's rights as a research participant and the confidential handling of the information and records. I understand that I may withdraw myself/my child from the study at any time without giving any reason. I understand that I would also be able to get further information in future and that my/my child's name and/or identity will not be used in the analysis of data and in sharing the results with others.

Based on the above, I have voluntarily agreed to provide information about my child's hospitalization as part of this aspect of the research study

(Please place a tick in the box, if you agree) ☐

Participant's age: Day 1 - <11 years

Name of participant: ____

Name of Father/Mother/guardian of a minor participant ______________________

Signature or left thumbprint of Father/Mother/guardian:___________________Date ____________

Name of witness:____

Signature of witness: ______________________________ Date ____________

Name of the personnel securing consent: ____

Signature of the personnel securing consent: ___________________Date ____________

Note- A signed and dated copy/photocopy of this document will be given to you

Appendix A 7 (ii)
Consent form for recording the hospitalization information (11 years - <18years)

Protocol No. PR-17115	Version No. 1.00	Date: 27-08-2018

Protocol Title: Assessing the impact of a Vi-Polysaccharide Conjugate Vaccine in preventing typhoid infection among Bangladeshi children – a Phase IIIb trial.

Investigator's name: Dr Firdausi Qadri, Principal Investigator (PI) of the protocol

Organization: icddr,b
Purpose of the research

Background
Typhoid fever is a serious infectious disease transmitted by taking water and food contaminated with a certain type of bacteria. The disease is common in the developing countries including Bangladesh, where the sanitation and pure water supply are not adequate. Now the disease is more commonly found in children, and immediate and appropriate treatment is needed to prevent complication. Commercially available vaccines against typhoid fever are not so effective in children. The Vi-Polysaccharide typhoid Conjugate Vaccine (Vi-TCV) is the most promising vaccine for controlling typhoid fever. It is important to study the effectiveness of the Vi-TCV by immunizing Bangladeshi children. This will help inform decision makers when considering the introduction of Vi-TCV into the routine expanded programme on immunisation (EPI) in Bangladesh.
The aim of the overall study is to evaluate the effectiveness of the typhoid vaccine.

Efficacy of the Vi-TCV vaccine will be evaluated with the Japanese encephalitis vaccine (SA14-14-2) in a participant- and observer-blinded, cluster-randomized trial design in this study. So, we have vaccinated the children aged 9 month to <16 years in the study area of Mirpur.

We are inviting all the residents in the study area who have been hospitalized to provide information on their hospitalization. This will help us identify if there is any difference in how many people get hospitalized or why people get hospitalized, depending on which group of the study they are in.

Reasons for inviting the specific person to participate in the study

You are invited to take part in the study as your household resides in our study area and your child was hospitalized with a fever or another health issues. So we are inviting you to participate in this aspect of our study.

Roles of the participants in this study

If you agree for your child to take part in this aspect of our study, then we would ask you some questions about your child's hospitalization episode i.e. cause and duration of hospital stay,

treatment and outcome etc. Our staff will interview you in your home and it will take 15-20 minutes to complete. We will not contact you again about this study component unless your child or a different member of your household another event of hospitalization in the future.

Genetic Test

We will not collect any samples if you agree for your child to participate in this aspect of the study. If your child is involved with other components of the study, we may collect blood sample and ask your permission to carry out genetic test on that sample.

Risk and Benefit

There is no direct benefit to you, your child or other members of your household. However information from this and other linked studies will help the government to make choices about the use of this vaccine to help prevent typhoid fever. It will not cost you anything to take part in this study.

There is no serious risk to your child in taking part in this aspect of the study.

Principal of Compensation

The participants will be provided with the best possible, free treatment for any research related injuries directly associated with this component of the study. You will not have to pay us and we will also not pay you for participating in the study.

Privacy, anonymity and confidentiality

All information that we collect during the study is strictly private and confidential.Your child will be identified by a number. Your/your child's name and address will not be revealed in any output from this study.

Storage of data and biological samples

We will not collect any biological samples if you agree for your child to participate in this aspect of the study.. We will store all information collected from you in password protected computers and paper documents will be locked in the file cabinets. At the end of the trial, all data and records will be electronically retained until the youngest child participating in the trial reaches 21 or for 5 years following completion of the research, whichever is longer.

Future use of data and/or biological samples

Stored data may be used in future research; some of the data will be shipped abroad for further analysis. Before using such data for any future study, we would first obtain permission from the Ethical Review Committee (ERC) of icddr,b. We would like to take your permission to use these data in the future. Anonymous information and data may be supplied to other researchers, which will not conflict with or violate the maintenance of privacy, anonymity and confidentiality of information.

The University of Oxford is responsible for ensuring the safe and proper use of any personal information you provide, solely for research purposes.

Right not to participate and withdraw

It is your choice whether you provide information on your child's hospitalization and it is entirely voluntary. You can withdraw your child from the study at any time by contacting the research team. You do not need to give a reason if you choose to withdraw. We will still use the information you gave us before your child's withdrawal. We will inform you of any changes to the study.

Communication for Queries, Concerns and Complaints

We will be happy to provide you further information about the study any time. If you want to know more about the research, you may ask now or may contact the following investigators at your later suitable time, either meeting with them personally or by contacting them over telephone, at the following addresses:

Dr. Firdausi Qadri
Address of investigator: Enteric and Respiratory Infections, IDD. icddr,b, Mohakhali, Dhaka 1212. Telephone: +880-2-8812529, Extension No. 2431.

If you want to know more about your rights as a research participant you may contact IRB Secretariat, RA, M. A. Salam Khan, phone no: 9827084 or PABX 8860523-32 Ext. 3206 personally at icddr,b or by contact by telephone.

Thank you for your co-operaton.

If you agree to our proposal of enrolling you in the study, please indicate that by putting your signature or your left thumb impression at the specified space below

Indicating consent

The investigators/ study staff has explained to me and I have fully understood the purpose, procedures, risks and benefits of participating in this aspect of the study, my/my child's rights as a research participant and the confidential handling of the information and records. I understand that I may withdraw myself/my child from the study at any time without giving any reason. I understand that I would also be able to get further information in future and that my/my child's name and/or identity will not be used in the analysis of data and in sharing the results with others.

Based on the above, I have voluntarily agreed to provide information about my child's hospitalization as part of this aspect of the research study

(Please place a tick in the box, if you agree) ☐

Participant's age: 11 years - <18 years

Name of participant: ____

Signature or left thumbprint of participant:_______________________ Date ____________

Name of Father/Mother/guardian of a minor participant ______________________

Signature or left thumbprint of Father/Mother/guardian:_______________________ Date ____________

Name of witness:____

Signature of witness: ______________________________ Date ____________

Name of the personnel securing consent: ____

Signature of the personnel securing consent: ___________________Date ____________

Note- A signed and dated copy/photocopy of this document will be given to you

Appendix A 7 (iii)
Consent form for recording the hospitalization information
(18 years and above)

Protocol No. PR-17115	Version No. 1.00	Date: 27-08-2018

Protocol Title: Assessing the impact of a Vi-Polysaccharide Conjugate Vaccine in preventing typhoid infection among Bangladeshi children – a Phase IIIb trial.

Investigator's name: Dr Firdausi Qadri, Principal Investigator (PI) of the protocol

Organization: icddr,b
Purpose of the research

Background
Typhoid fever is a serious infectious disease transmitted by taking water and food contaminated with a certain type of bacteria. The disease is common in the developing countries including Bangladesh, where the sanitation and pure water supply are not adequate. Now the disease is more commonly found in children, and immediate and appropriate treatment is needed to prevent complication. Commercially available vaccines against typhoid fever are not so effective in children. The Vi-Polysaccharide typhoid Conjugate Vaccine (Vi-TCV) is the most promising vaccine for controlling typhoid fever. It is important to study the effectiveness of the Vi-TCV by immunizing Bangladeshi children. This will help inform decision makers when considering the introduction of Vi-TCV into the routine expanded programme on immunisation (EPI) in Bangladesh.
The aim of the overall study is to evaluate the effectiveness of the typhoid vaccine.

Efficacy of the Vi-TCV vaccine will be evaluated with the Japanese encephalitis vaccine (SA14-14-2) in a participant- and observer-blinded, cluster-randomized trial design in this study. So, we have vaccinated the children aged 9 month to <16 years in the study area of Mirpur.

We are inviting all the residents in the study area who have been hospitalized to provide information on their hospitalization. This will help us identify if there is any difference in how many people get hospitalized or why people get hospitalized, depending on which group of the study they are in.

Reasons for inviting the specific person to participate in the study

You are invited to take part in the study as your household resides in our study area and your child was hospitalized with a fever or another health issues. So we are inviting you to participate in this aspect of our study.

Roles of the participants in this study

If you agree for your child to take part in this aspect of our study, then we would ask you some questions about your child's hospitalization episode i.e. cause and duration of hospital stay, treatment and outcome etc. Our staff will interview you in your home and it will take 15-20

minutes to complete. We will not contact you again about this study component unless your child or a different member of your household another event of hospitalization in the future.

Genetic Test

We will not collect any samples if you agree for your child to participate in this aspect of the study. If your child is involved with other components of the study, we may collect blood sample and ask your permission to carry out genetic test on that sample.

Risk and Benefit

There is no direct benefit to you, your child or other members of your household. However information from this and other linked studies will help the government to make choices about the use of this vaccine to help prevent typhoid fever. It will not cost you anything to take part in this study.

There is no serious risk to your child in taking part in this aspect of the study.

Principal of Compensation

The participants will be provided with the best possible, free treatment for any research related injuries directly associated with this component of the study. You will not have to pay us and we will also not pay you for participating in the study.

Privacy, anonymity and confidentiality

All information that we collect during the study is strictly private and confidential.Your child will be identified by a number. Your/your child's name and address will not be revealed in any output from this study.

Storage of data and biological samples

We will not collect any biological samples if you agree for your child to participate in this aspect of the study.. We will store all information collected from you in password protected computers and paper documents will be locked in the file cabinets. At the end of the trial, all data and records will be electronically retained until the youngest child participating in the trial reaches 21 or for 5 years following completion of the research, whichever is longer.

Future use of data and/or biological samples

Stored data may be used in future research; some of the data will be shipped abroad for further analysis. Before using such data for any future study, we would first obtain permission from the Ethical Review Committee (ERC) of icddr,b. We would like to take your permission to use these data in the future. Anonymous information and data may be supplied to other researchers, which will not conflict with or violate the maintenance of privacy, anonymity and confidentiality of information.

The University of Oxford is responsible for ensuring the safe and proper use of any personal information you provide, solely for research purposes.

Right not to participate and withdraw

It is your choice whether you provide information on your hospitalization and it is entirely voluntary. You can withdraw yourself from the study at any time by contacting the research team. You do not need to give a reason if you choose to withdraw. We will still use the information you gave us before your child's withdrawal. We will inform you of any changes to the study.

Communication for Queries, Concerns and Complaints

We will be happy to provide you further information about the study any time. If you want to know more about the research, you may ask now or may contact the following investigators at your later suitable time, either meeting with them personally or by contacting them over telephone, at the following addresses:

Dr. Firdausi Qadri
Address of investigator: Enteric and Respiratory Infections, IDD. icddr,b, Mohakhali, Dhaka 1212. Telephone: +880-2-8812529, Extension No. 2431.

If you want to know more about your rights as a research participant you may contact IRB Secretariat, RA, M. A. Salam Khan, phone no: 9827084 or PABX 8860523-32 Ext. 3206 personally at icddr,b or by contact by telephone.

Thank you for your co-operaton.

If you agree to our proposal of enrolling you in the study, please indicate that by putting your signature or your left thumb impression at the specified space below

Indicating consent

The investigators/ study staff has explained to me and I have fully understood the purpose, procedures, risks and benefits of participating in this aspect of the study, my rights as a research participant and the confidential handling of the information and records. I understand that I may withdraw myself from the study at any time without giving any reason. I understand that I would also be able to get further information in future and that my/my child's name and/or identity will not be used in the analysis of data and in sharing the results with others.

Based on the above, I have voluntarily agreed to provide information about my child's hospitalization as part of this aspect of the research study

(Please place a tick in the box, if you agree) ☐

Participant's age: 18 years and above

Name of participant: ____

Signature or left thumbprint of participant:_______________________ Date ___________

Name of witness:____

Signature of witness: ______________________________ Date ___________

Name of the personnel securing consent: ____

Signature of the personnel securing consent: ___________________Date ___________

Note- A signed and dated copy/photocopy of this document will be given to you

Appendix A8 (i)
Consent form for pregnancy follow up and outcome evaluation (below 11 years)

Protocol No. PR-17115	Version No. 1.00	Date: 07-11-2018

Protocol Title: Assessing the impact of a Vi-Polysaccharide Conjugate Vaccine in preventing typhoid infection among Bangladeshi children – a Phase IIIb trial.

Investigator's name: Dr Firdausi Qadri, Principal Investigator (PI) of the protocol

Organization: icddr,b

Purpose of the research

Background

A certain type of bacteria causes typhoid fever. You can catch typhoid fever when you drink water or eat food that has these bacteria in it. The bacteria can get from the gut into the blood and can cause high fever, diarrhoea, upset stomach and headache. The infection can become serious and should be treated with antibiotics to kill the bacteria.

The disease is common in Bangladesh and children suffer the most from typhoid fever. Though there are vaccines available to help prevent typhoid fever, they are not given in Bangladesh routinely and they do not always work very well. The typhoid vaccine we are using in this study is currently the most promising vaccine for stopping typhoid fever. However, it is important for us to study how well this vaccine works in Bangladeshi children. This will help decision makers to choose whether to give all children in Bangladesh this vaccine. In this study we are inviting children aged 9 months to <16 years who live in Mirpur to vaccinate either with the typhoid vaccine, or another vaccine that protects against Japanese encephalitis (JE).

You would have either received the typhoid or JE vaccine. We will come and visit the vaccine recipient every 6 months to find out how he/she has been since we last saw him/her.

Over the next 2 years we would also like to invite you and the vaccine recipient to come and see one of our study doctors if you have a fever of 38°C or more and/or that lasts for 2 days or more. If you or the vaccine recipient have to stay in hospital or are very unwell for any reason in those same 2 years, we will ask you to contact us to tell us. The information we collect at these visits will help us to find out if the vaccine helps stop typhoid.

Reasons for inviting the specific person to participate in the study

Though the vaccine has not been extensively evaluated in pregnancy, it has been shown to be safe in adults and children. The vaccine recipient became pregnant before or within 3 months of vaccination; we are inviting you to participate in our study.

Roles of the participants in this study

If you agree for the vaccine recipient to take part in this aspect of our study, then we would ask you or the vaccine recipient about:

- The pregnancy status
- Information related to the pregnancy and/or
- Details about the birth outcome, if relevant

Our staff will interview you or the vaccine recipient at your home and it will take 15-20 minutes to complete. We will contact you or the vaccine recipient again every month until the end of this pregnancy. In addition from 8 months gestation onwards, we will telephone you or the vaccine recipient once a week.

Genetic Test

This pregnancy follow-up does not include the collection of any sample. So we will not collect any sample if you agree for the vaccine recipient to participate in this aspect of the study.

Risk and Benefit

There is no direct benefit to you, the vaccine recipient or other members of your household. However information from this study will provide information about the vaccination and pregnancy. It will not cost you anything to take part in this study.

There is no serious risk to the vaccine recipient in taking part in this aspect of the study.

Principal of Compensation

No remuneration will either be offered or taken for participation in this study.

Privacy, anonymity and confidentiality

All information that we collect during the study is strictly private and confidential. The vaccine recipient will be identified by a number. The vaccine recipient name and address will not be revealed in any output from this study.

Storage of data and biological samples

We will not collect any biological samples if you agree for the vaccine recipient to participate in this aspect of the study. We will store all medical and personal information about the vaccine recipient in password protected computers and documents will be locked in the file cabinets.

Future use of data and/or biological samples

Stored data may be used in future research; some of the data will be transferred abroad for further analysis. Before using such data for any future study, we would first obtain permission from the Ethical Review Committee (ERC) of icddr,b. We would like to ask your permission to use these data in the future. Anonymous information and data may be supplied to other researchers, which will not conflict with or violate the maintenance of privacy, anonymity and confidentiality of information.

The University of Oxford is responsible for ensuring the safe and proper use of any personal information you provide, solely for research purposes.

Right not to participate and withdraw

It is your or your child's choice whether you provide information about the pregnancy and it is entirely voluntary. You can withdraw from the study at any time by contacting the research team. You do not need to give a reason if you choose to withdraw. We will still use the information you gave us before withdrawal. We will inform you of any changes to the study.

Communication for queries, concerns and complaints

We will be happy to provide you further information about the study any time. If you want to know more about the research, you may ask now or may contact the following investigators at your later suitable time, either meeting with them personally or by contacting them over telephone, at the following addresses:

Dr. Firdausi Qadri

Address of investigator: Enteric and Respiratory Infections, IDD. icddr,b, Mohakhali, Dhaka 1212. Telephone: +880-2-8812529, Extension No. 2431.

If you want to know more about your rights as a research participant you may contact IRB Secretariat, RA, M. A. Salam Khan, phone no: 9827084 or PABX 8860523-32 Ext. 3206 personally at icddr,b or by contact by telephone.

Thank you for your co-operaton.

If you agree to our proposal of enrolling you in the study, please indicate that by putting your signature or your left thumb impression at the specified space below

Indicating consent

The investigators/ study staff has explained to me and I have fully understood the purpose, procedures, risks and benefits of participating in this aspect of the study, my/my child's rights as a research participant and the confidential handling of the information and records. I understand that I may withdraw the vaccine recipient from the study at any time without giving any reason. I understand that I would also be able to get further information in future and that name and/or identity will not be used in the analysis of data and in sharing the results with others.

Based on the above, I have voluntarily agreed to provide information about the pregnancy as part of this aspect of the research study

(Please place a tick in the box, if you agree) ☐

Participant's age: below 11 years

Name of participant: ______________________________________

Name of Father/Mother/guardian of a minor participant ______________________

Signature or left thumbprint of Father/Mother/guardian:__________________Date ___________

Name of witness:____

Signature of witness: _____________________________ Date ___________

Name of the personnel securing consent: _______________________________________

Signature of the personnel securing consent: __________________Date ___________

Note- A signed and dated copy/photocopy of this document will be given to you

/

Appendix A8 (ii)
Consent form for pregnancy follow up and outcome evaluation (11 years or older)

Protocol No. PR-17115	Version No. 1.00	Date: 07-11-2018

Protocol Title: Assessing the impact of a Vi-Polysaccharide Conjugate Vaccine in preventing typhoid infection among Bangladeshi children – a Phase IIIb trial.

Investigator's name: Dr Firdausi Qadri, Principal Investigator (PI) of the protocol

Organization: icddr,b

Purpose of the research

Background
A certain type of bacteria causes typhoid fever. You can catch typhoid fever when you drink water or eat food that has these bacteria in it. The bacteria can get from the gut into the blood and can cause high fever, diarrhoea, upset stomach and headache. The infection can become serious and should be treated with antibiotics to kill the bacteria.

The disease is common in Bangladesh and children suffer the most from typhoid fever. Though there are vaccines available to help prevent typhoid fever, they are not given in Bangladesh routinely and they do not always work very well. The typhoid vaccine we are using in this study is currently the most promising vaccine for stopping typhoid fever. However, it is important for us to study how well this vaccine works in Bangladeshi children. This will help decision makers to choose whether to give all children in Bangladesh this vaccine. In this study we are inviting children aged 9 months to <16 years who live in Mirpur to vaccinate either with the typhoid vaccine, or another vaccine that protects against Japanese encephalitis (JE).

You would have either received the typhoid or JE vaccine. We will come and visit you or the vaccine recipient every 6 months to find out how he/she has been since we last saw him/her.

Over the next 2 years we would also like to invite you or the vaccine recipient to come and see one of our study doctors if you have a fever of 38°C or more and/or that lasts for 2 days or more. If you have to stay in hospital or are very unwell for any reason in those same 2 years, we will ask you to contact us to tell us. The information we collect at these visits will help us to find out if the vaccine helps stop typhoid.

Reasons for inviting the specific person to participate in the study

Though the vaccine has not been extensively evaluated in pregnancy, it has been shown to be safe in adults and children. You or the vaccine recipient became pregnant before or within 3 months of vaccination; we are inviting you to participate in our study.

Roles of the participants in this study

If you agree for you or the vaccine recipient to take part in this aspect of our study, then we would ask you or the vaccine recipient about:

- The pregnancy status
- Information related to the pregnancy and/or
- Details about the birth outcome, if relevant.

Our staff will interview you or the vaccine recipient at your home and it will take 15-20 minutes to complete. We will contact you or the vaccine recipient again every month until the end of this pregnancy. In addition from 8 months gestation onwards, we will telephone you once a week to find out how you or the vaccine recipient is.

Genetic Test

This pregnancy follow-up does not include the collection of any sample. So we will not collect any sample if you agree for you/or the vaccine recipient to participate in this aspect of the study.

Risk and Benefit

There is no direct benefit to you or the vaccine recipient or other members of your household. However information from this study will provide information about the vaccination and pregnancy. It will not cost you anything to take part in this study.

There is no serious risk to you or the vaccine recipient in taking part in this aspect of the study.

Principal of Compensation

No remuneration will either be offered or taken for participation in this study.

Privacy, anonymity and confidentiality

All information that we collect during the study is strictly private and confidential. You or the vaccine recipient will be identified by a number. Your or the vaccine recipient name and address will not be revealed in any output from this study.

Storage of data and biological samples

We will not collect any biological sample if you agree for you or the vaccine recipient to participate in this aspect of the study. We will store all medical and personal information about you or the vaccine recipient in password protected computers and documents will be locked in the file cabinets.

Future use of data and/or biological samples

Stored data may be used in future research; some of the data will be transferred abroad for further analysis. Before using such data for any future study, we would first obtain permission from the Ethical Review Committee (ERC) of icddr,b. We would like to ask your permission to use these data in the future. Anonymous information and data may be supplied to other researchers, which will not conflict with or violate the maintenance of privacy, anonymity and confidentiality of information.

The University of Oxford is responsible for ensuring the safe and proper use of any personal information you provide, solely for research purposes.

Right not to participate and withdraw

It is your or the vaccine recipient's choice whether you provide information about the pregnancy and it is entirely voluntary. You or the vaccine recipient / can withdraw from the study at any time by contacting the research team. You do not need to give a reason if you choose to withdraw. We will still use the information you gave us before withdrawal. We will inform you of any changes to the study.

Communication for queries, concerns and complaints

We will be happy to provide you further information about the study any time. If you want to know more about the research, you may ask now or may contact the following investigators at your later suitable time, either meeting with them personally or by contacting them over telephone, at the following addresses:

Dr. Firdausi Qadri

Address of investigator: Enteric and Respiratory Infections, IDD. icddr,b, Mohakhali, Dhaka 1212. Telephone: +880-2-8812529, Extension No. 2431.

If you want to know more about your rights as a research participant you may contact IRB Secretariat, RA, M. A. Salam Khan, phone no: 9827084 or PABX 8860523-32 Ext. 3206 personally at icddr,b or by contact by telephone.

Thank you for your co-operaton.

If you agree to our proposal of enrolling you in the study, please indicate that by putting your signature or your left thumb impression at the specified space below.

Indicating consent

The investigators/ study staff has explained to me and I have fully understood the purpose, procedures, risks and benefits of participating in this aspect of the study, my or the vaccine recipient's rights as a research participant and the confidential handling of the information and records. I understand that I may withdraw myself from the study at any time without giving any reason. I understand that I would also be able to get further information in future and that my or the vaccine recipient's name and/or identity will not be used in the analysis of data and in sharing the results with others.

Based on the above, I have voluntarily agreed to provide information about the pregnancy as part of this aspect of the resear dch study.

(Please place a tick in the box, if you agree) ☐

Participant's age: 11 Years or older

Name of participant: ___________________________________

Signature or left thumbprint of participant:_________________Date __________

Name of Father/Mother/guardian of a minor participant ____________________

Signature or left thumbprint of Father/Mother/guardian:_________________Date __________

Name of witness:____

Signature of witness: __________________________ Date __________

Name of the personnel securing consent: ___________________________________

Signature of the personnel securing consent: _________________Date __________

Note- A signed and dated copy/photocopy of this document will be given to you

APPENDIX B: SCHEDULE OF PLANNED PROCEDURES (PARTICIPANT SCHEDULE)

Table 1: Visit and sample schedule

Visit	1	2	3	4	5	6	7	8 (&)	Final contact (a)
Days post vaccination	0	7	28	180	365	545	730 post trial start**		
Permissible time window (days)		+7/-1	+/-4	+/- 28	+/-56	+/-56	+/-90		
Screening	X								
Consent	X								
Randomization	X								
Vaccination	X								
Medical history and exam	X								
Blood collection (b)	X*		X			X	X	X	
Blood volume	~3-5ml		~3-5ml			~3-5ml	~3-5ml	~3-5ml	
AEFI follow-up (c)		X							
Census update and Follow-up contact (d)*** (&)				X	X	X	X (follow-up)	X (census)	
Un-blinding, vaccination with Vi-TCV (if in control group) and documentation of vaccination									X

a) All participants will be un-blinded at the same time, after the final follow-up contact has been completed, regardless of when an individual joined the study. Participants who were enrolled during census updates will only complete visits occurring between their enrollment and the subsequent completion of the trial.

b) Blood collection in a subset of 1000 Vi-TCV, 500 control participants. Total volume ~12-20ml per participant
   - *Blood draw on day 0 will occur before vaccination

c) At day 7 a subset of participants will be contacted to collect all AEFIs

d) Follow-up contact includes:
   - Census update, and ensure participant and family still lives in area and happy to continue with study,
   - Reminder to attend trial health care facility if they develop fever of ≥2 days and/or temperature of ≥38°C at presentation.

*** Verbal autopsies and hospitalization follow-up contacts will occur when CHWs, who visit residents bi-weekly, identify a death or a hospitalization in the household

& Final census and final blood draws will b conducted when it is safe to do so, after COVID-19, as close to the planned timeline as possible. Final follow-up for all participants, regardless of the number of days since vaccination, will happen at the end of the study, before unblinding, when it is safe to do so, as close to the originally planned time-frame as possible.

APPENDIX C: PLANNED TRIAL ACTIVITIES (TRIAL STAFF SCHEDULE)

Day	0	7	28	90	180	270	365	455	545	635	730	Final contact
Vaccination of new participants #	X				X		X		X			
Active surveillance of Subset Population for 4800 children												
Post vaccination census update					X		X		X		X&	
informed written consent with questionnaire*	X				X		X		X		X	
Passive surveillance\ Unblinding, vaccination of control group with Vi-TCV and documentation of vaccination												X

Vaccination of new participants will be carried out every 6 months following the census update: children who will be in-migrated and reached at eligible age group (9 months or older)

∞Vaccination will be started after completion of baseline census. Passive surveillance will be carried out up to 24 months. Last group of new participants will be vaccinated following the 3rd post vaccination census (18 months) and surveillance will be carried out for once after 6 months during close out census

*Written informed consent will be obtained if any participant had hospitalization for fever/or abdominal discomfort in last 6 months\blood will be taken for the febrile patients as well as adverse event will be recorded for all participants.

& Final census will occur when it is safe to do so, post COVID-19

APPENDIX D: UNSCHEDULED PROCEDURES FLOWCHART

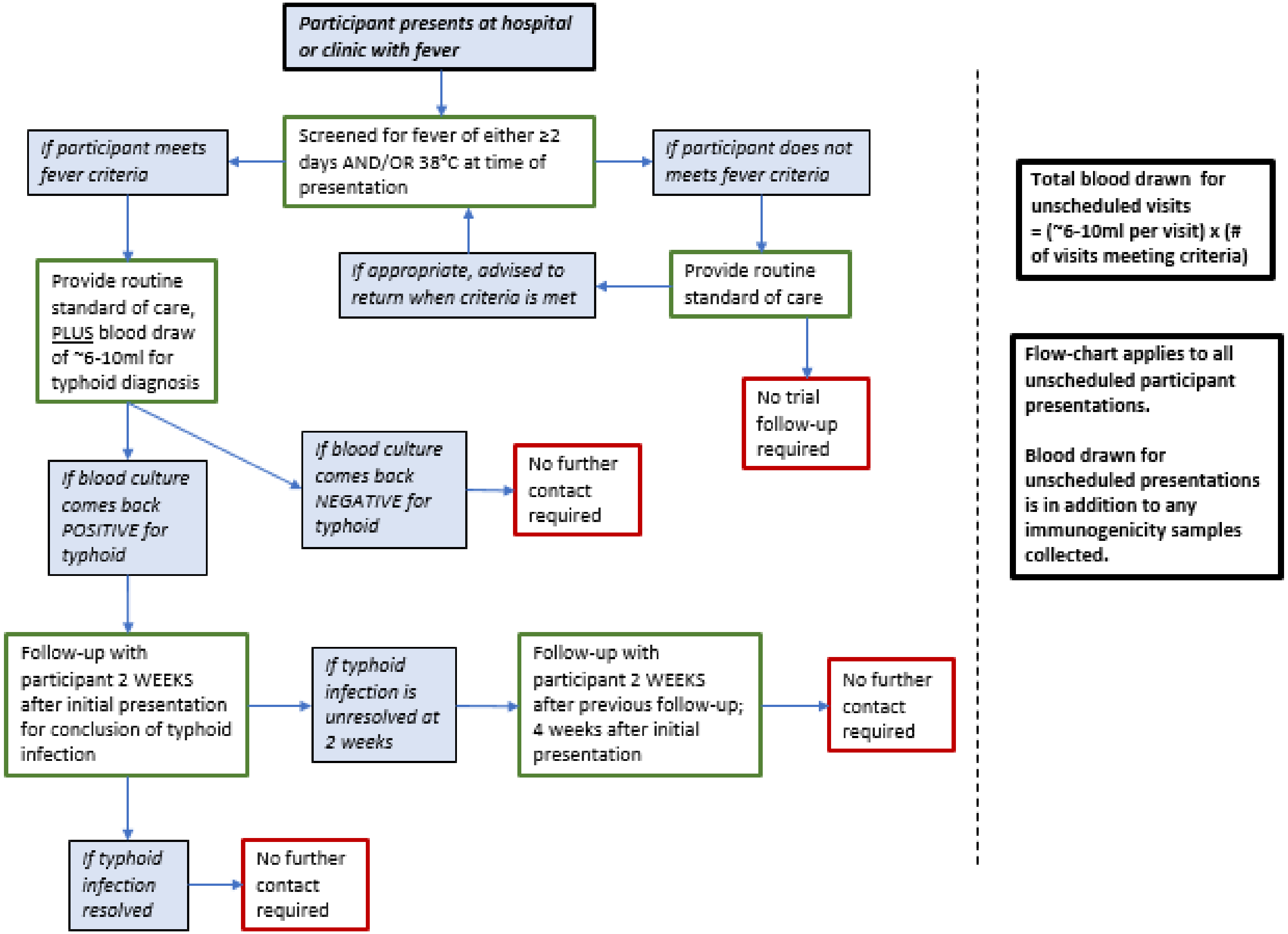

APPENDIX E: SAE REPORTING FLOW CHART

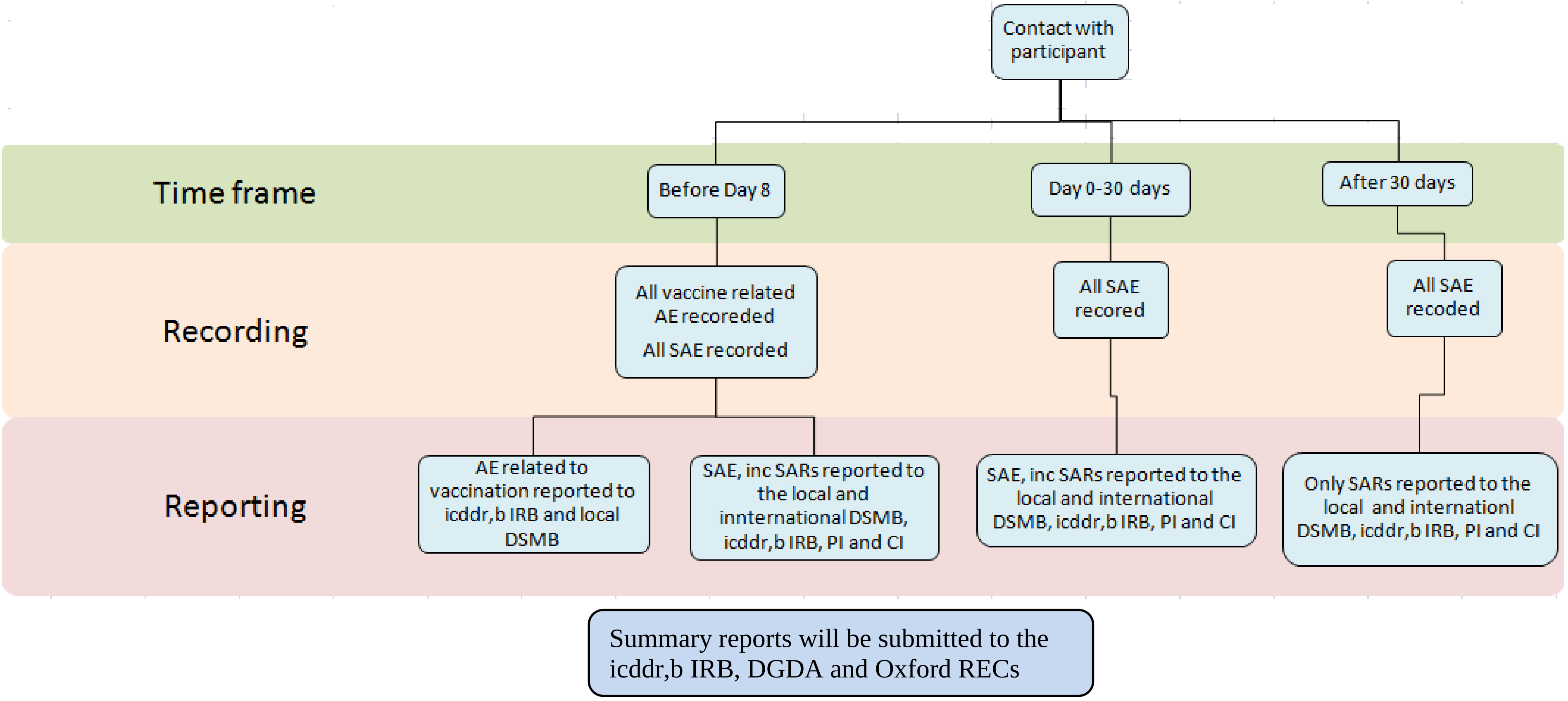

APPENDIX F
HEALTHCARE FACILITIES FOR PASSIVE SURVEILLANCE

1. Mirpur Field Clinic
2. Suhrawardy Hospital
3. Radda Barnen Mirpur-10
4. Adhunik Hospital Mirpur
5. Shishu hospital, Kalshi, Mirpur
6. Kurmitola General Hospital
7. Kingston Hospital, Mirpur
8. Islami Bank Hospital, Mirpur

APPENDIX G
RISK ASSESSMENT AND PROCEDURES FOR UNBLINDED ANALYSIS

INTERIM ANALYSIS FOR TyVAC-BANGLADESHS AFETY AND IMMUNOGENICITY DATA

	NAME	TITLE	SIGNATURE	DATE
Written by:	Xinxue Liu	Trial Statistician		20th Feb 2019
Reviewed by:	Merryn Voysey	Unblinded Statistician		20th Feb 2019
Approved by:	Andrew J Pollard	Chief Investigator		21st Feb 2019
	Firdausi Qadri	Principal Investigator		
	John D. Clemens	Principal Investigator		

	NAME	TITLE	SIGNATURE	DATE
Written by:	Xinxue Liu	Trial Statistician		
Reviewed by:	Merryn Voysey	Unblinded Statistician		
Approved by:	Andrew J Pollard	Chief Investigator		
	Firdausi Qadri	Principal Investigator		22/02/2019
	John D. Clemens	Principal Investigator		22/02/2019

Introduction:

The design of TyVAC-Bangladesh is a participant- and observer-blinded, cluster-randomised controlled trial. The interim data analyses proposed should follow sound statistical principles and should comply with the study protocol. A statistical analysis plan (SAP) should be developed before carrying out any formal interim analysis.

1. **Purpose:**

   The purpose of this document is to define the procedure for handling, analyzing and presenting unblinded safety and immunogenicity data. This document will also evaluate the risk of unblinding the study by disseminating unblinded results of safety and immunogenicity.

2. **Scope:**

   This document applies to all staff working on TyVAC-Bangladesh.

3. **Definitions:**

Randomisation

Introduces a deliberate element of chance into the assignment of treatments to subjects in a clinical trial. During subsequent analysis of the trial data, it provides a sound statistical basis for the quantitative evaluation of the evidence relating to treatment effects.

Blinding

Intended to limit the occurrence of conscious and unconscious bias in the conduct and interpretation of a clinical trial arising from the influence which the knowledge of treatment may have on the recruitment and allocation of subjects, their subsequent care, the attitudes of subjects to the treatments, the assessment of endpoints, the handling of withdrawals, the exclusion of data from analysis, and so on. The overall aim is to prevent identification of the treatments until all opportunities for bias have gone.

4. **Blinding matrix before and after presenting the unblinded results**

By “unblinded results”, we mean the results that are summarised by Vi-TCV group and control vaccine group.

Role	Access to individual level data	Before*	After*
Chief investigator	No	Blinded	Blinded
Principal investigators	No	Blinded	Blinded
Senior researchers	Yes	Blinded	Blinded
Trial manager/coordinator	Yes	Blinded	Blinded
Trial statistician	All data except immunogenicity data and unblinded randomisation list	Partial blinded	Unblinded
Unblinded statistician	All data	Unblinded	Unblinded
IT manager (OVG)	All data except immunogenicity ELISA data	Unblinded	Unblinded
IT manager (icddr,b)	All data except unblinded randomisation list and	Blinded	Blinded
Data management team	All data except immunogenicity ELISA data	Blinded	Blinded
Unblinded vaccination team	Unblinded randomisation list; vaccination data	Unblinded	Unblinded
Follow-up research team	Follow-up data, including passive surveillance, hospitalisation, 6-month follow up data	Blinded	Blinded
Safety study team	AEFI, SAE data	Blinded	Blinded (potential risk for unblinding)
Blinded lab study team (immunogenicity)	Immunogenicity ELISA data	Blinded	Blinded (potential risk for unblinding)
Blinded lab study team	All lab outputs except immunogenicity	Blinded	Blinded (potential risk for unblinding)

* Before and after presenting the unblinded aggregated data.

Blinded: Having no knowledge of the vaccine allocation for each cluster

Partial blinded: Knowing part of the treatment allocation, e.g. masked randomisation list (vaccine A and vaccine B)

Potential risk: Knowing the vaccine allocation if accessing to additional results.

N.B. The above matrix is based on the fact that there are no overlapping roles in this study between blinding and unblinding team.

5. Specific procedures

Figure 1 shows the procedures to carry out the unblinded safety/immunogenicity analysis.

5.1 Presenting unblinded aggregated safety results

Since the trial statistician has access to the safety data, the trial statistician will be unblinded once the unblinded safety data are presented. The procedures to analyse the unblinded safety datawill be as follows:

- The international and local, data and safety monitoring board (DSMB) and ethics committee should be consulted and approval to be given before carrying out any analysis;
- The trial statistician should liaise with CI and PIs to sign off a statistical analysis plan (SAP);
- The trial statistician will liaise with the unblinded statistician to work on the analysis of unblinded safety data and validation of the unblinded safety results
- The trial statistician should ensure any safety events occurring in only one of the vaccine groupsis not presented in the unblinded safety results to avoid unblinding the safety team. Events occurring in small numbers of participants in one arm only, can be combined in presentations (by having an "other" category for rare events) or removed from presentations.

Potential impact

The trial statistician will become unblinded and there will be no effect on the status of blinding for all the other parties if the above procedures are strictly followed.

5.2 Presenting unblinded aggregated immunogenicity results

As the trial statistician will be unblinded after analysing and interpreting the unblinded safety data, the trial statistician will lead the analysis of immunogenicity data. The blood samples for the immunogenicity study are collected from a subset of 1,500 children in 18 clusters on a 2:1 basis (Vi-TCV vs JE) at baseline (D0), at D28, at 18 months (D545), and at two years (D730) post-vaccination. A blindedlab study team at icddr,b will process the blood samples and measure

the anti-Vi antibody. The procedures to store and analyse the unblinded immunogenicity ELISA datawill be as follows:

- The international and local, data and safety monitoring board (DSMB) and ethics committee should be consulted and approval to be given before carrying out any analysis;
- The trial statistician should liaise with CI and PIs to sign off a statistical analysis plan (SAP);
- As there was no re-labelling process prior to processing at icddr,b and the immunogenicity data includes participants' study ID (3xxxxx), the immunogenicity data should be stored in a secure server/computer, which limits the access to unblinded icddr,b lab team only.
- IT team should control the access to the main study database (RedCAP, census and follow up database) and the lab team should not have access to the above databases and data extracts from these database.
- The immunogenicity data should be transferred from the lab team at icddr,b to the trial statistician/unblinded statistician at University of Oxford via a secure data transfer process. Any data transferred should be password protected.
- The immunogenicity data should be stored at a secure server at University of Oxford, which limits the access to statisticians at the Oxford Vaccine Group.

The trial statistician will analyse the immunogenicity data. All the unblinded immunogenicity results should be validated by a second statistician.

Potential impact

The trial statistician will become unblinded and there will be no effect on the status of blinding for all the other parties if the above procedures are strictly followed.

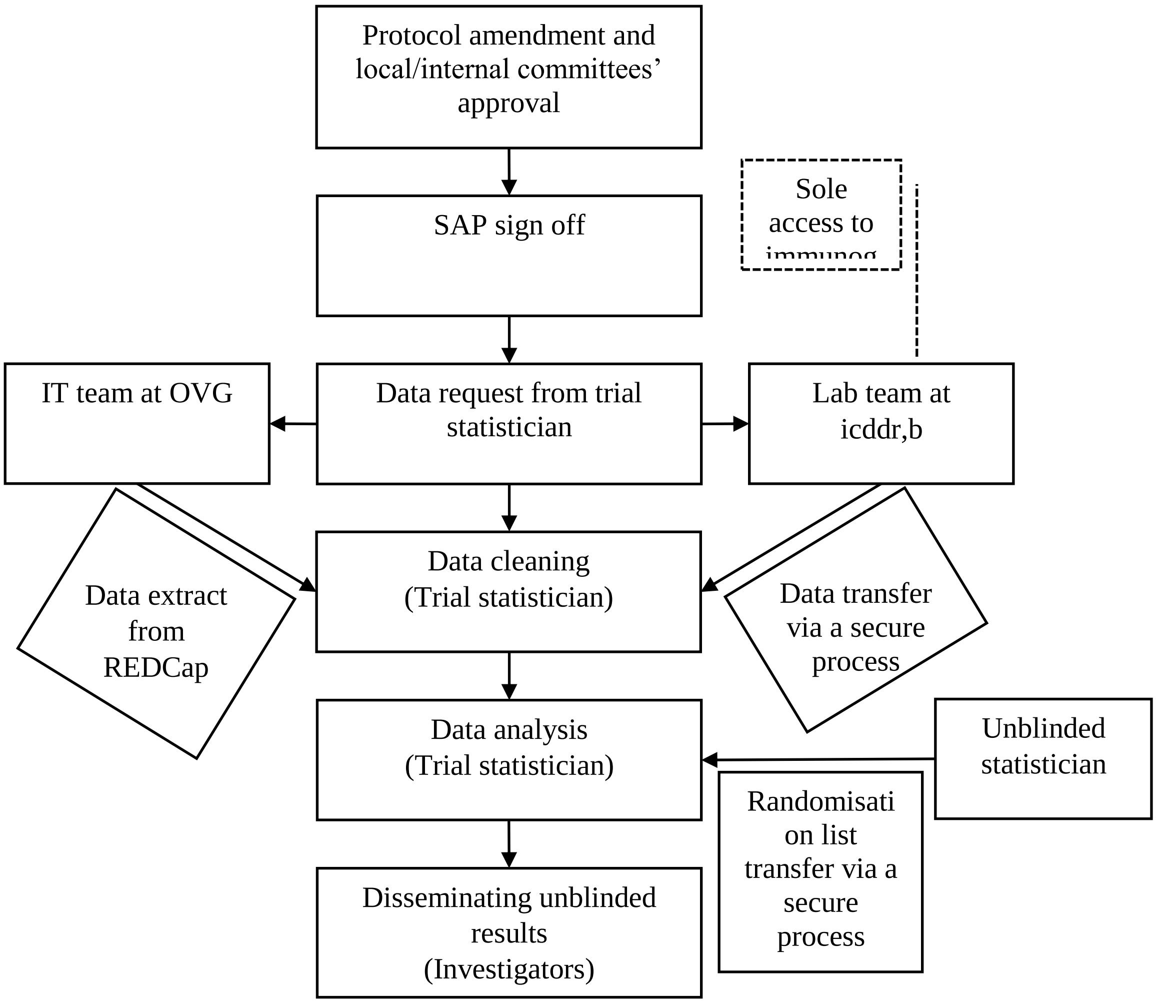

Figure 1 Flowchart of the unblinded safety/immunogenicity analysis procedures

APPENDIX H: PROCEDURE OF UNBLINDING AND FINAL VISIT

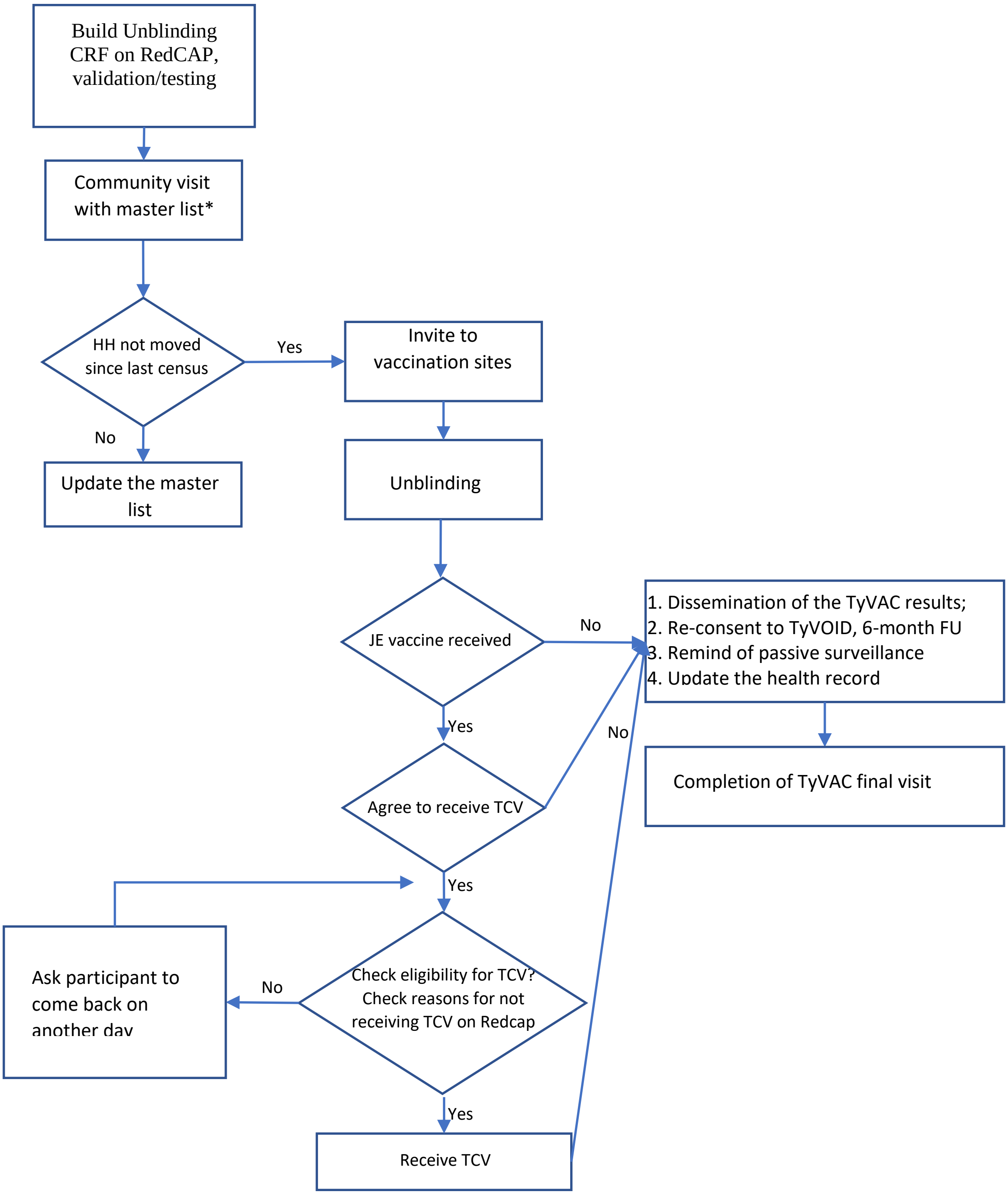

APPENDIX I: PLANNED TRIAL ACTIVITIES FOR TyVOID

Year	2021												2022												2023		
Month	Jan	Feb	Mar	Apr	May	Jun	Jul	Aug	Sep	Oct	Nov	Dec	Jan	Feb	Mar	Apr	May	Jun	Jul	Aug	Sep	Oct	Nov	Dec	Jan	Feb	Mar
Vaccination of control group with Vi-TCV																											
Blood collection Immunogenicity																											
Census																											
Verbal autopsy																											
Invitation and Passive Surveillance																											

Response to the comments of external reviewers:

Reviewer #1

Query: The only query is about the choice of the control JE vaccine, which differs from the test vaccine in being given s.c. rather than i.m. and needs fresh preparation from lyophilized vials within 6 hours before administration compared to the ready-made prefilled test vaccine syringes. These differences suggest that it will be difficult to maintain the blinding of vaccination. It is suggested that IPV in ready-made syringes might be preferable as control vaccine ? – it would be relevant to have a comment on this point with further motivation for their proposed control vaccine choice from the investigators.

Response: The Japanese encephalitis vaccine, SA14-14-2 JE, is WHO-prequalified and has been shown to be safe and immunogenic in Bangladesh, where Japanese encephalitis is endemic, as well as in trials in many countries. It has an identical administration single dose regime to Vi-TCV and is licensed for use from 9 months of age. We appreciate the reviewer's concern regarding the maintenance of the blinding of vaccination, however, the main trial will be a participant- and observer-blinded, and cluster-randomized study. The trial will aim for participant- and observer-blinding. The vaccines will not be repackaged and relabelled for the purposes of this trial; however the label on multi-dose vials will be covered to conceal vaccine names. Efforts will be made to conceal the identity of the administered vaccine from both participants and staff involved in follow-up procedures, with only the vaccinating trial staff aware of which vaccination has been given. Subjects will not be told the identity of the administered vaccine. To help maintain observer blinding the staff members undertaking bleeding, contact at 7 days after dosing for assessment of adverse events, contacts in the 6 monthly censuses and surveys, or follow-up for enteric fever at the surveillance sites will not be involved in these subsequent study procedures in the clusters where they had vaccinated participants. To help ensure blinding of the investigators, all vaccinations will be carried out by trail staffs that are not named investigators in the study protocol. Separate vaccination teams will be responsible for the study which will include nurses, trained field assistants and volunteers recruited for this purpose.

Reviewer #2

No comment to respond.

icddr,b

Check-List

Check-list for Submission of Research Protocol
For Consideration of the Research Review Committee (RRC)
[Please check all appropriate boxes]

1. Has the proposal been reviewed, discussed and cleared by all listed investigators?

   ☒ Yes ☐ No

   If the response is No, please clarify the reasons:

2. Has the proposal been peer-reviewed externally?

   ☒ Yes ☐ No ☐ External Review Exempted

   If the response is 'No' or "External Review Exempted", please explain the reasons:

   If the response is "Yes", please indicate if all of their comments have been addressed?

   ☒ Yes (please attach)

   ☐ No (please indicate reason(s)):

3. Has the budget been reviewed and approved by icddr,b's Finance?

   ☒ Yes ☐ No (reason):

4. Has the Ethics Certificate(s) been attached with the Protocol?

   ☒ Yes ☐ No

   If the answer is 'No', please explain the reasons:

F Qadri
Signature of the Principal Investigator

23/11/2017
Date

LIST OF ABBREVIATIONS

AE	Adverse event
AR	Adverse reaction
BMGF	Bill and Melinda Gates Foundation
CI	Chief Investigator
CRF	Case Report Form
CT	Clinical Trials
DGDA	Directorate General of Drug Administration
DSMB	Data and Safety Monitoring Board
EPI	Expanded Programme on Immunizations
GCP	Good Clinical Practice
IB	Investigators Brochure
ICF	Informed Consent Form
ICH	International Conference on Harmonisation
iDSMB	International DSMB
IMP	Investigational Medicinal Product
IRB	Independent Review Board
LDSMB	Local DSMB
PI	Principal Investigator
SAE	Serious Adverse Event
SAR	Serious Adverse Reaction
SMPC	Summary of Medicinal Product Characteristics
SOP	Standard Operating Procedure
STRATAA	Strategic Typhoid alliance across Africa & Asia
SUSAR	Suspected Unexpected Serious Adverse Reactions
Vi-PS	Vi antigen polysaccharide vaccine
Vi-TCV	Vi antigen typhoid conjugate vaccine
VVM	Vaccine Vial Monitor

STATISTICAL ANALYSIS PLAN

ASSESSING THE IMPACT OF A VI-POLYSACCHARIDE CONJUGATE VACCINE IN PREVENTING TYPHOID INFECTION AMONG BANGLADESHI CHILDREN – A PHASE IIIB TRIAL

Short title: TyVAC Bangladesh: Typhoid Vaccine Trial

Ethics Ref: OxTREC 5-18

OVG Ref: OVG 2017/11

idccr,b Protocol No.: PR-17115

Oxford Protocol Date and Version No.: V8 20-07-2020

Icddr,b Protocol Date and Version No.: V13.00 27-07-2020

Sponsor: University of Oxford

SAP version No: 1.2 21-09-2020

	NAME	TITLE	SIGNATURE	DATE
**Written by:**	Xinxue Liu	Senior Statistician		21 Sep 2020
**Reviewed by:**	Merryn Voysey	Lead Statistician		21 Sep 2020
**Approved by:**	Andrew Pollard	Chief Investigator		21 Sep 2020

	John Clemens	Principal Investigator		Sept 23, 2020
	Firdausi Qadri	Principal Investigator		Sep 23, 2020

Table of Contents

1 Introduction 5
1.1 Description of TyVAC 5
1.2 Purpose and scope of the plan 5
2 Study Methods 6
2.1 Trial design 6
2.2 Sample size 7
2.3 Randomisation 8
2.4 Objectives and Outcome Measures 9
3 Analysis – General considerations 15
4 Definition of study population 16
5 Primary analysis - Total and Overall Protection by Typhoid Vaccine against Typhoid Fever (Whole clusters) 17
5.1 Population and typhoid episodes for analysis 17
5.2 Statistical analysis 18
5.3 Subgroup analyses 19
5.4 Sensitivity analyses 20
5.5 Multiple episodes of typhoid fever 21
6 Secondary analyses 21
6.1 Safety of Typhoid Vaccine 21
6.2 Protection by Typhoid Vaccine against Suspected Typhoid and Clinical Typhoid 22
6.3 Total Protection by Typhoid Vaccine against Paratyphoid Fever 23
6.4 Indirect Protection by Typhoid Vaccine against Typhoid 24
6.5 Vaccine Protection among the Inner 80% Cluster Residents 24
7 Exploritory analyses 26
7.1 Hospitalisation 26
7.2 Immunogenicity 26
7.3 Absenteeism 27
7.4 Mortality 28
7.5 Abdominal surgery 28
7.6 Vaccine Protection by Different Size of Inner Cluster 29

8 Reference 30

1 INTRODUCTION

1.1 Description of TyVAC

The Typhoid Vaccine Acceleration Consortium (TyVAC) is a Bill and Melinda Gates Foundation funded project to generate evidence for Vi-TCV vaccine impact, and accelerate use of Typhoid Conjugate Vaccines in countries with significant typhoid burden. Managed by University of Maryland, in collaboration with University of Oxford, and PATH, the TyVAC programme includes vaccination trials, health economics studies, country preparedness support for routine vaccine introduction, and the collation and synthesis of typhoid research and evidence.

Three sites have been identified for parallel field impact studies; Kathmandu, Nepal; Dhaka, Bangladesh; and Blantyre, Malawi. Each represents a geographical setting where enteric fever is endemic and has a substantial local burden of disease. In each site, independent studies with differing study designs will be implemented to identify a range of impact scenarios. Between the sites, there is a range of demographic and geographic variation to give confidence in the generalisability of the study results. The statistical analysis plan for the Dhaka, Bangladesh field impact study is presented here.

1.2 Purpose and scope of the plan

This document details the proposed analysis of the main paper(s) reporting results from the TyVAC Bangladesh: Typhoid Vaccine Study. The results reported in these papers should follow the strategy set out here. Subsequent analyses of a more exploratory nature will not be bound by this strategy, though they are expected to follow the broad principles set out here. The principles are not intended to curtail exploratory analysis, nor to prohibit accepted practices, but they are intended to establish the principles that will be followed, as closely as possible, when analysing and reporting the trial.

2 STUDY METHODS

2.1 Trial design

A pilot phase, prior to the main study, individually randomised 200 children, in an area separate from the main trial site, in an age stratified manner to receive either Vi-TCV or the JE vaccine. Safety data were presented to the local DSMB (LDSMB), IRB and to the Directorate General of Drug Administration (DGDA), the National Regulatory Authority of Bangladesh prior to initiating the main cluster randomised trial.

The main trial was a participant- and observer-blinded, cluster-randomised study of the typhoid conjugate vaccine (Vi-TCV), brand name: Tybar-TCV, in Bangladeshi children. The population within a selected geographical catchment area of Mirpur, Dhaka, was offered entry into the study. The aim was to vaccinate at least 32,500 eligible, consenting children/guardians within the target age range (9 months to <16 years) residing in the catchment area at baseline. 150 Residential clusters of ca. 1250 people each were randomised in a 1:1 ratio to receive Vi-TCV or the control vaccine (Japanese encephalitis vaccine (JE): SA 14-14-2).

At six month intervals following baseline, census updates of the population were done in all clusters, and children fulfilling the eligibility criteria for participation who have not received the study vaccines allocated to the cluster will be offered the vaccine following census updates, whilst retaining blinding. Final vaccination will be done following the final census update.

A subset of approximately 4800 participants were selected on a 1:1 basis (Vi-TCV vs JE) from all 150 clusters to be contacted by telephone or in person seven (7) days after vaccination for follow-up and to record any adverse events following vaccination. The selection of these participants will be age-stratified (< 5 years vs ≥5 years of age with an allocation ratio of 1:1).

A subset of approximately 1500 participants was selected on a 2:1 basis (Vi-TCV vs JE) to have blood samples collected at baseline (D0), at D28, at 18 months (D545), and at two years (D730) post-vaccination to study immunogenicity. The selection of these participants was age-stratified stratified (< 5 years vs ≥ 5 years of age with an allocation ratio of 1:1).

Surveillance for enteric fever was undertaken in at least 9 health care facilities in Mirpur for all residents of the participating clusters for a minimum of one month preceding baseline and continuing until the end of the study. In this surveillance, all consenting residents from the participating clusters who present with a subjective history of ≥2 days fever and/or a temperature of ≥38°C had data on the fever episode collected, a blood culture taken (6-10 ml, depending on age), and received appropriate clinical management. Those with positive blood cultures was visited at home to confirm their identity given at the treatment centre, to

collect information about their illness, and to review the treatment given, and adjust as necessary, based on laboratory testing.

Community Health Workers (CHWs) visited residents of all clusters weekly to remind them about attending healthcare facilities and identify if any deaths or hospitalisations have occurred in all cluster residents and vaccinees. If a death or hospitalisation had occurred, verbal consent was obtained to record the residents contact details and arrange a follow up visit. A trained member of staff conducted either a verbal autopsy or recorded the details of the hospitalisation including surgeries, medications and school/work absenteeism. In order to cross-check data, at the time of the census updates, information about all deaths and births since the previous survey was collected. Verbal autopsies were done for all cluster residents, including vaccinees who die during the 2 year trial period.

Every 6 months, vaccinated participants had a follow-up contact to collect data on fevers, episodes of clinically diagnosed and culture confirmed typhoid, school/work absenteeism and other significant illness.

The original plan to carry out the final study visit around two years after initial vaccination campaign and base the primary analysis on this accrued data had to be modified due to the world pandemic of COVID-19. The timing to start the final visit will be reviewed by the study team regular. At the final study visit all participants and trial staff will be unblinded. At this point, pilot study participants and both control and intervention groups of the main trial will be informed of their vaccination status and have their vaccines documented on the patient record. All participants in the control group will then be offered vaccination with the Vi-TCV vaccine.

Two additional changes were implemented. First, because the COVID pandemic delayed the final study visit and because field surveillance for typhoid was disrupted by the pandemic, the study team decided to carry out the final analysis based on the 18-month follow up data with the approval of the safety and monitoring board. A second change concerned the population to analyse in the primary analysis. The original protocol specified that the inner 80% of the population in each cluster would be analysed. However, with the subsequent recognition that this strategy, which was designed to shield estimates of vaccine herd protection from inward transmission of typhoid, would generalise only to an inner core of a population targeted to receive mass typhoid immunization, rather than to the entire targeted population, it was decided that the entire clusters should constitute the population for the primary analysis, and the 80% inner population would be analysed as a secondary analysis. Once the final study visit is completed, a follow-up analysis will be carried out using all the data.

2.2 Sample size

The original sample size calculations were based on the following assumptions:

1. An overall incidence of typhoid fever of 50 cases per year, per 100,000 persons in the entire population, with higher incidence rates in children under 16 years.
2. Age specific incidence rates were determined from the age distribution of typhoid cases from earlier cohort studies in Bangladesh.
3. A direct protective effect of vaccination of 80% (protection of vaccinees) and an indirect effect of 20% (protection of neighboring non-vaccinees), as predicted from mathematical modelling.
4. A coefficient of variation of 0.5.
5. Cluster size (of the inner clusters) of 1,000, of whom an expected 289 will be aged 9 months to <16 years.
6. 60% average vaccine coverage of the target age group throughout follow-up.
7. 2 years of follow-up.

Based on the above assumptions, the sample size calculated for use in this trial was 43,350 children, within 150 clusters, each on average containing 1,000 participants in the inner-cluster, randomised 1:1 to receive Vi-TCV or JE vaccine. As the innermost 80% of the population was specified for the original primary analysis (now changed, *vide supra*), the total number of children eligible for vaccination in both inner and outer clusters, was therefore 43,350 x 1.25 = 54,188, of which we expected approximately 60% to participate, resulting in approximately 32,500 children vaccinated at baseline. Due to the high migration rate in the study area, catch up vaccination campaign was conducted for every 6 months to maintain the expected vaccine coverage rate.

With these assumptions, the trial had 98% power to detect a 82% level of total vaccine protection by Vi-TCV and 74% power to detect a 43% level of overall protection by Vi-TCV, and p-value <0.05 (2 tailed).

2.3 Randomisation

Computer generated randomisation list was prepared by the study statistician. Randomisation lists will allocate each cluster to receive either Vi-TCV or the control vaccine, in equal numbers (75 clusters in each arm) using stratified block randomisation. Stratification will be by the geographic wards, the distance to the closest surveillance clinic (above the median versus at or below the median), and the number of children 9 months to <16 years of age, (above the median versus at or below the median). As we had odd number of clusters in some strata, the randomisation list could end up with unequal number of clusters between the two arms. To maximise the study power, we only considered randomisation list with 75 clusters in each arm as the final randomisation list. To achieve this, we generated around 3,000 randomisation lists using stratified block randomisation to acquire 1000 randomisation lists meeting the condition of 75 clusters in each arm. We randomly selected one as the final randomisation list from the 1000 lists.

2.4 Objectives and Outcome Measures

	Objective	Outcome Measure	Endpoints and variables for analysis
Primary	1. To determine the relative and absolute rate reduction of symptomatic infection caused by S. Typhi in recipients of Vi-TCV (total vaccine protection)	The incidence of blood culture confirmed typhoid fever in vaccinees in intervention clusters compared to control clusters	**Endpoints:**<br>Blood culture confirmed typhoid at Passive surveillance;<br>**Variables:**<br>REDCap: fev_result; fev_6; fev_11_days
	2. To determine the relative and absolute rate reduction of symptomatic infection caused by S. Typhi among all residents in the clusters allocated to Vi-TCV (overall vaccine protection)	The incidence of blood culture confirmed typhoid fever in all residents of the intervention clusters compared to control clusters	**Endpoints:**<br>Blood culture confirmed typhoid at passive surveillance;<br>**Variables:**<br>REDCap: fev_result; fev_6; fev_11_days
Secondary	1. To investigate safety outcomes associated with Vi-TCV vaccination, within the study population	The proportion of participants developing local and solicited adverse events within the first 7 days post-vaccination, and serious adverse events within 6 months of vaccination, as determined through sub-sample at 7 days, and self-reporting at follow-up contact	**Endpoints:**<br>Actively collected and self-reported AEFI;<br>SAE<br>**Variables:**<br>REDCap: all variables in AEFI form and SAE form

	2. To determine the relative and absolute rate reduction of symptomatic infection caused by S. Typhi in non-recipients of Vi-TCV (indirect vaccine protection)	The incidence of blood culture confirmed typhoid fever in non-vaccinees in intervention clusters compared to control clusters.	**Endpoints:**<br>Blood culture confirmed typhoid at passive surveillance;<br>**Variables:**<br>REDCap: fev_result; fev_6; fev_11_days
	3. To determine the impact of vaccination with Vi-TCV on the incidence of inpatient/outpatient admission rates for fever and abdominal discomfort among vaccines	Rates of participants with a history of ≥2 days of persistent fever, and/or a temperature of ≥38 degrees C upon presentation, together with abdominal discomfort in vaccinees in intervention clusters compared to control clusters.	**Endpoints:**<br>Temperature of ≥38 ˚C and/or persistent fever (≥2 days) with gastrointestinal symptoms at passive surveillance;<br>**Variables:**<br>REDCap: fev_4_temp; fev_6; fev_8_start; fev_10_end; fev_11_days; fev_12
	4. To determine the impact of vaccination with Vi-TCV on the incidence of inpatient/outpatient admission rates for fever and abdominal discomfort among all residents of the clusters	Rates of participants with a history of ≥2 days of persistent fever, and/or a temperature of ≥38 degrees C upon presentation, together with abdominal discomfort among all residents of the Vi-TCV clusters compared to the control vaccine clusters	**Endpoints:**<br>Temperature of ≥38 ˚C and/or persistent fever (≥2 days) with gastrointestinal symptoms at passive surveillance;<br>**Variables:**<br>REDCap: fev_4_temp; fev_6; fev_8_start; fev_10_end; fev_11_days; fev_12

	5. To determine the impact of vaccination with Vi-TCV on the incidence of inpatient/outpatient admission rates for clinical typhoid fever among vaccinees	Rates of patients with clinical diagnoses of typhoid fever in vaccinees in intervention clusters compared to control clusters	**Endpoints:**<br>Clinical typhoid at passive surveillance;<br>**Variables:**<br>REDCap: fev_14
	6. To determine the impact of vaccination with Vi-TCV on the incidence of inpatient/outpatient admission rates for clinical typhoid fever among all residents of the clusters	Rates of patients with clinical diagnoses of typhoid fever among all residents of the Vi-TCV clusters compared to the control vaccine clusters	**Endpoints:**<br>Clinical typhoid at passive surveillance;<br>**Variables:**<br>REDCap: fev_14
	7. To determine the effectiveness of and rate reduction by Vi-TCV in preventing blood culture-confirmed symptomatic infection caused by S. Paratyphi in recipients of the vaccine (total vaccine protection)	The incidence of blood culture confirmed paratyphoid fever in vaccinees in intervention clusters compared to control clusters.	**Endpoints:**<br>Blood culture confirmed paratyphoid at Passive surveillance;<br>**Variables:**<br>REDCap: fev_result
Exploratory	1. To measure differences in all-cause hospitalisation rates by treatment arm	Incidence of all cause hospitalisation in community-based surveys of all residents of the Vi-TCV clusters compared to the control vaccine clusters	**Endpoints:**<br>Hospitalisation at Passive surveillance;<br>Hospitalisation at hospitalisation visit;<br>**Variables:**<br>REDCap: fev_22_hospital;<br>Hospitalisation: HosSin; PSIDY

	2. To determine the immunogenicity of Vi-TCV in a subset of participants, stratified by age groups.	Assay of anti-Vi antibodies in blood samples collected at baseline (Day 0), at one month (Day 28), in a subset of participants from each treatment arm	**Endpoints:**<br>Assay of anti-Vi antibodies in blood samples at baseline (Day 0), one month (Day 28), 18 months (day 545) and two years (day 730)<br>**Variables:**<br>All variables in the external immunogenicity data.
	3. To determine the persistence of antibodies induced by Vi-TCV in stratified age groups.	Assay of anti-Vi IgG antibodies in blood samples collected at baseline (day 0), 18 months (day 545) and two years (day 730) in a subset of participants receiving intervention and control vaccinations	
	4. To determine the incidence of absenteeism from school/work as a result of typhoid infection, by treatment arm	Incidence of absenteeism from school/work in vaccinated children and their guardians as a result of confirmed typhoid infection in intervention clusters compared to control clusters.	**Endpoints:**<br>Absenteeism recorded at Passive surveillance;<br>**Variables:**<br>REDCap: fu_21_absence; fu_24; fu_27_abs_rel; fu_30
	5. To determine the incidence of absenteeism from work/school as a result of typhoid infection among all cluster residents by treatment arm	Incidence of absenteeism from school/work in all cluster residents as a result of culture confirmed typhoid infection in intervention clusters compared to control clusters	**Endpoints:**<br>Absenteeism recorded at passive surveillance;<br>**Variables:**<br>REDCap: fu_21_absence; fu_24; fu_27_abs_rel; fu_30

	6. To measure all-cause mortality, mortality with fever, and mortality with fever and abdominal pain among vaccinees by treatment arm	Rates and circumstances of mortality, recorded from hospital records and verbal autopsies via six-monthly census updates, in vaccinees in intervention clusters compared to control clusters.	**Endpoints:**<br>Death recorded at 6-month vaccinees follow-up;<br>Death at verbal autopsy<br>**Variables:**<br>6-month follow up: ChWell;<br>All variables in verbal autopsy database
	7. To measure all-cause mortality, mortality with fever, and mortality with fever and abdominal pain among all cluster residents by treatment arm	Rates and circumstances of mortality, recorded from hospital records and verbal autopsies via six-monthly census updates, in all residents of the Vi-TCV clusters compared to the control vaccine clusters	**Endpoints:**<br>Death recorded at 6-month vaccinees follow-up;<br>Death at verbal autopsy<br>**Variables:**<br>6-month follow up: ChWell;<br>All variables in verbal autopsy database
	8. To measure the rate of surgical intervention for acute abdominal complaints by treatment arm among vaccinees by treatment arm	Rates of acute abdominal surgery, and of surgery for intestinal perforation, in vaccinees in intervention clusters compared to control clusters.	**Endpoints:**<br>Abdominal surgery for intestinal perforation recorded at passive surveillance;<br>Abdominal surgery for intestinal perforation recorded at hospitalisation visit;<br>**Variables:**<br>REDCap: fev_30_surgery; fev_32_type; fev_33_abdom<br>Hospitalisation: Surg; SurgType; SurgAbd
	9. To measure the rate of surgical intervention for acute abdominal	Rates of acute abdominal surgery, and of surgery for intestinal	**Endpoints:**

	complaints by treatment arm among all cluster residents by treatment arm	perforation, in all residents of the Vi-TCV clusters compared to the control vaccine clusters	Abdominal surgery for intestinal perforation recorded at passive surveillance;<br>Abdominal surgery for intestinal perforation recorded at hospitalisation visit;<br>**Variables:**<br>REDCap: fev_30_surgery; fev_32_type; fev_33_abdom<br>Hospitalisation: Surg; SurgType; SurgAbd
Non-protocol exploratory	1. To explore the herd protection of Vi-TCV among residents living in the innermost 25%, 50% and 80% households of the clusters	The outcomes include:<br>Primary outcome 1 & 2;<br>Secondary outcome 2, 3, 4, 5 &6	**Endpoints and variables:**<br>See the endpoints and variables for the corresponding outcomes

3 ANALYSIS – GENERAL CONSIDERATIONS

All the primary analyses will be on an intention-to-treat basis, i.e. the study participants will be analysed based on the allocation randomised to the cluster where they live. Histograms and boxplots will be used to check the distribution and possible outliers for continuous variables. The outliers will be examined closely to confirm the validity of the data. Mathematical transformations ($\log_{10}$) will be applied, where appropriate, in order to render a normal distribution. If an appropriate transformation does not normalise the distribution, variables will be categorized. Continuous variables that follow an approximately normal distribution will be summarised using means, standard deviations and range values, and number of missing. Skewed continuous variables will be summarised using medians/geometric mean (where appropriate), inter-quartile ranges and range values, and number of missing. Categorical/binary variables will be summarised using frequencies and percentages.

Baseline characteristics will be summarised for each group to describe the study population. No formal statistical comparisons of baseline characteristics between randomised groups will be conducted. Patient throughput from census, enrolment, through randomisation, follow up and analysis will be presented in a CONSORT flow diagram. This will contain the numbers of participants randomly assigned to each group, receiving vaccination, completing the study and analysed for the primary outcome. It will also include a breakdown of reasons for withdrawal.

For two primary analyses (addressing the two primary objectives), the statistical tests will be 2-sided and p value less than 0.025 will be considered significant. The significance level for all the other secondary and exploratory analyses will be 2-sided 0.05, unless specified otherwise in sections 5 below.

4 DEFINITION OF STUDY POPULATION

Figure 1 shows the Venn diagram of the TyVAC Bangladesh baseline study population (this group will be expanded to include post-baseline births and immigrants, as described below). The primary analysis will be the entire study population within the whole clusters, and the secondary analysis will include the study population within the inner clusters, which include around 80% of each cluster's population. Exploratory analysis will also be conducted by changing the size of the inner clusters. Table 1 lists different groups of study population.

Table 1 Definition of study population groups

Vaccinees	All the study participants that are vaccinated at baseline during the study period
Residents	All the people that are identified during the baseline census and are confirmed living in the study area
Vaccinees in the AEFI study	The ~4,800 vaccinees that are selected into the subset of safety study
Vaccinees in the immunogenicity study	The ~1,500 vaccinees that are selected into the subset of immunogenicity study

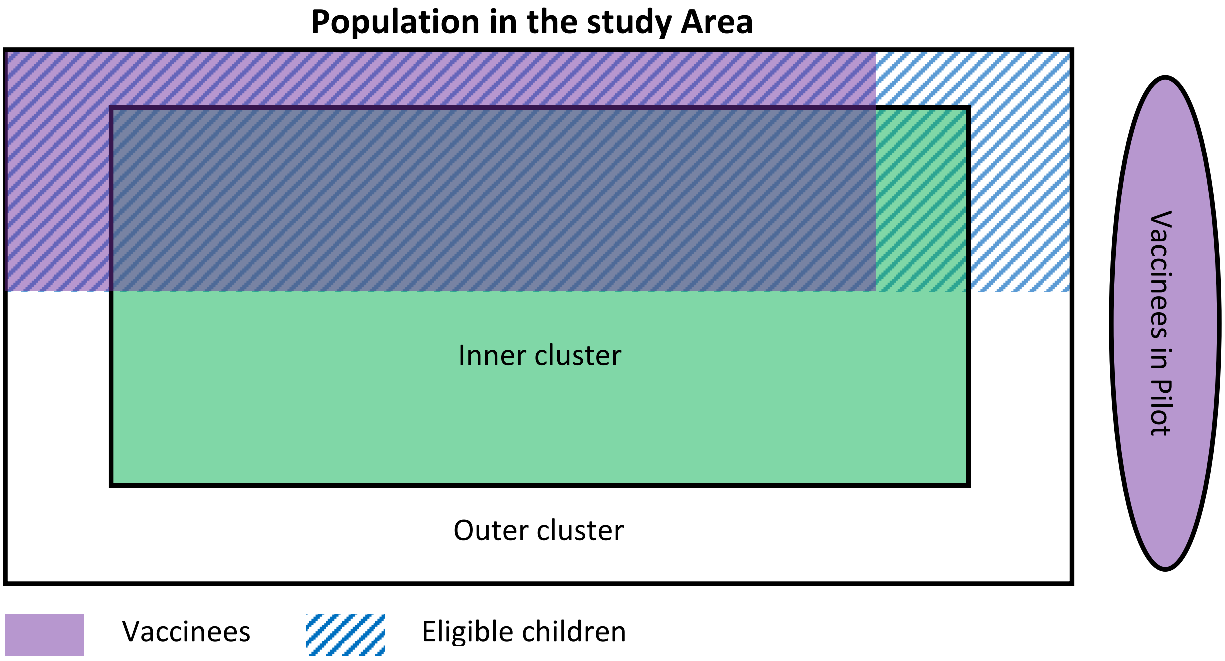

Figure 1 Study population in TyVAC Bangladesh

5 PRIMARY ANALYSIS - TOTAL AND OVERALL PROTECTION BY TYPHOID VACCINE AGAINST TYPHOID FEVER (WHOLE CLUSTERS)

The primary analyses covers the below outcomes:

1. The incidence of blood culture confirmed typhoid fever in **vaccinees** in intervention clusters compared to control clusters (total vaccine protection)

2. The incidence of blood culture confirmed typhoid fever in **all residents** of the intervention clusters compared to control clusters (overall vaccine protection)

5.1 Population and typhoid episodes for analysis

The population for the primary analyses (total and overall typhoid vaccine protection against typhoid) will be persons present at baseline and persons who enter the clusters post-baseline via birth or in-migration from the outside (not from another cluster) (Figure 2). The "date of residence" to calculate the follow-up time for overall vaccine protection will be defined as median date of baseline vaccination campaign (30 April 2018) for non-vaccinees living in the study area before the start of the trial, or date of vaccination for those vaccinated at the baseline vaccination campaign, or date of first migration into or birth into the study area for those moved or born into the area post-baseline. Follow-up of this population will end when, in the same cluster, death, migration out, loss of follow up, or follow-up at the 18-month census occurs, whichever comes first. Analysis of total vaccine protection will follow the subset of this population from the time of vaccination (either at baseline or at catch-up vaccination) until the termination of follow-up, as just defined.

For the analysis of total vaccine protection, episodes of typhoid fever will be counted if the onset of the episode begins one or more days after vaccination. In this respect, passive surveillance visits for care of fever will be concatenated to define a febrile episode if the date of fever onset for one visit is within 14 days of the date of discharge for the previous visit. Febrile episodes in which at least one blood culture was positive for *S. typhi* will be defined as typhoid fever episodes, and the date of fever onset for the first visit of a typhoid fever episode will define the date of onset of that typhoid fever episode.

For the analysis of overall vaccine protection, episodes of typhoid fever will be counted if the onset of the episode begins one or more days after the "date of residence". Thus, estimation of overall typhoid vaccine protection will be based on the incidence of typhoid amongst all persons followed from the "date of residence", and estimation of total typhoid vaccine protection will be based on the incidence of typhoid among the subset of the overall protection population who become vaccinated in the cluster in which their "date of residence" occurred.

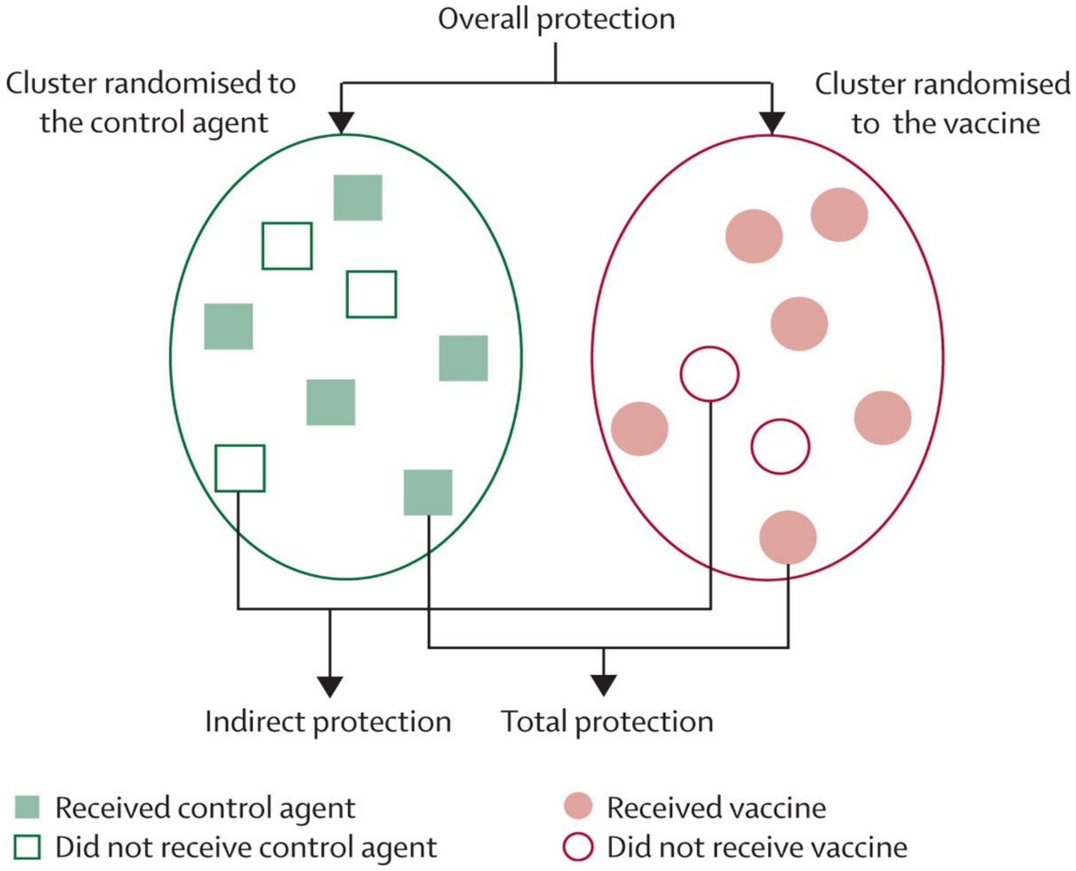

Figure 2 Measurement of vaccine-induced herd protective effects in a cluster-randomised vaccine trial (1)

5.2 Statistical analysis

For analysis of total and overall typhoid vaccine protection, person-years of follow-up will be calculated, and typhoid episodes will be counted according to the rules outlined in ***5.1***.

Let d_{ijk} be the number of typhoid fever episodes observed in the individual k in the ith arm (i= 1: TCV; i=0: JE) from the jth cluster (j=1, 2 …, 150), and y_{ijk} be the person-years observed from this individual. Then, d_{ijk} follows the Poisson distribution with mean:

$$E(d_{ijk}) = \lambda_{ijk} \times y_{ijk}$$

where λ_{ijk} is incidence rate of blood culture confirmed typhoid fever in the kth individual in the ith arm and j^{th} cluster.

The mixed effects statistical model (Poisson model) for λ_{ijk} is:

$$log(\lambda_{ijk}) = \alpha + \beta_i + \sum \gamma_l z_{ijkl} + \mu_j$$

In this equation, we assume that there are a number of covariates z_l (l=1, 2 …, L) and that z_{ijkl} is the observed value of z_l in the individual k in the ith arm from the j^{th}cluster. The covariates z_l include variables measured at the cluster level and those measured at individual level. The fixed effects in this model include β_i, and γ_l, while μ_j is the random effect for the j^{th}cluster, representing the between-cluster variability and α is the overall intercept.

In the above equation, we assume β_0 to be 0 and μ_j to be normally distributed with mean 0. Therefore, the mean incidence rate in the JE clusters and TCV clusters are given by:

$$\lambda_0 = \exp(\alpha + \sum \gamma_l z_l)$$

$$\lambda_1 = \exp(\alpha + \beta_1 + \sum \gamma_l z_l)$$

The average incidence rate ratio (IRR) of Vi-TCV clusters compared to JE clusters is: $\lambda_1/\lambda_0 = \exp(\beta_1)$. The 95% confidence internal (CI) for IRR will be presented. The vaccine protection will be calculated as (1 – IRR) x 100%, where IRR is the incidence rate ratio (Vi-TCV: control).

We will test the hypotheses in the primary analyses that there is no difference in the incidence of blood culture confirmed typhoid fever between relevant members of the TCV and JE groups using the above mixed effects Poisson model. The covariates z_l adjusted in the model include:

Design variables: the number of children 9 months to <16 years of age, ward and distance of cluster to the nearest health facility;

Other covariates: age at vaccination for total vaccine protection or age at date of residence for overall protection, gender, WASH variables captured at "date of residence", including household toilet type, household source of drinking water, household type of drinking water, hand wash before meal, and hand wash after defecation.

The cumulative incidence of typhoid will be summarised using the Kaplan-Meier method. For any participant with more than one blood culture confirmed typhoid episodes, only the first event will be used in the Kaplan-Meier analysis. The time to typhoid fever will be computed as time from onset of follow-up to the date of fever onset of the blood culture confirmed typhoid. Persons with no confirmed typhoid will be censored as discussed in section 5.1.

5.3 Subgroup analyses

Subgroup analyses for the primary outcome will be conducted using above mixed effects Poisson model after excluding the subgroup variables, where needed. The adjusted IRR and 95% CI will be presented for each subgroup. The subgroups analyses include:

- Ward (ward 2, ward 3 and ward 5)
- Age at vaccination for total protection, or Age at "date of residence" for overall protection (< 2 years, 2-4 years, 5-15 years and ≥ 16 years)
- Gender (male and female)
- Socioeconomic status at "date of residence" (quartiles)
- Household toilet type at "date of residence"
- Household source of drinking water at "date of residence"
- Household type of drinking water at "date of residence" (treated and not treated)
- Hand wash before meal at "date of residence" (with soap and without soap)
- Hand wash after defecation at "date of residence" (with and without soap/ash/soil)

5.4 Sensitivity analyses

Counting process model

In the sensitivity analysis, we will include all the follow-up time in the analysis for participants moving between clusters in the study area. The person-years of follow-up will be computed as the time from vaccination (total protection) or "date of residence" (overall protection) to the last known date of residence in the study area. The follow-up time after moving out the cluster, in which their "date of residence" occurred, will be included in the analysis. The data will be organised in a counting process form, i.e. each row of the data represents a time interval (time0, time1] when the corresponding participant lives in one location. The time dependent variables for a time interval include all the cluster level variables of the cluster where the participant lives during the interval, e.g. cluster number, cluster distance to clinics, vaccine allocation. In the total protection analysis, all vaccinee's follow-up data after vaccination will contribute to the arm of the vaccine they received in the analysis. The data before vaccination will be treated as non-vaccinees and thus will not contribute to the analysis of total vaccine protection. For the overall protection analysis, the data of a time interval will contribute to the corresponding arm of the cluster in that time interval. For vaccinees who were vaccinated at a catch up vaccination campaign, the data before vaccination will be treated as non-vaccinees and included in the analysis.

We will use the Andersen–Gill Cox model with robust standard errors to fit the data. If the model fails to converge, we will explore other possible survival models to fit the data.

Missing data imputation

Missing data on primary outcome occurs mainly when people met the fever criteria and attended the passive surveillance, but was not willing to give blood.

In the sensitivity analyses, we will describe the frequency and proportion of the above people between the two arms. We will impute the blood culture results for these children using multiple imputation and explore the impact on the total and overall vaccine protection.

Exclusion of early typhoid cases and less severe typhoid cases

Further sensitivity analyses will be carried out to estimate total and overall vaccine protection after excluding:

- blood culture positive cases whose onset dates are within two weeks of vaccination;
- blood culture positive cases that occurred in participants with fewer than 3 days of fever;
- Blood culture positive cases that not hospitalised.

5.5 Multiple episodes of typhoid fever

One of the assumptions for Poisson regression is that the observation of one event must be independent of another. Theoretically, a child can have more than one episode of blood culture confirmed typhoid. However, we believe that the probability of having a second typhoid fever will be different from the probability of the first one for the same individual. This is because the antibody level changes after a typhoid infection and the antibody level is associated with the chance of infection. Given that multiple episodes of typhoid fever in one individual is rare, we decided to only use the first blood confirmed typhoid fever in the analysis.

We will compare the mean and variance of the primary outcome, and if the data is over-dispersed, i.e. variance >> mean, we will change the primary analysis of primary outcome in 5.1.2 from mixed effects Poisson model to zero-inflated Poisson or zero-inflated negative binomial model depending on the variability and distribution of the data.

6 SECONDARY ANALYSES

6.1 Safety of Typhoid Vaccine

The proportion of participants developing local and solicited adverse events within the first 7 days post-vaccination, and serious adverse events within 6 months of vaccination, as determined through sub-sample at 7 days, and self-reporting at follow-up contact

6.1.1 Populations for analysis

The safety analysis will be carried out in the below four populations:

- The 200 vaccinees in the pilot safety study;

- The ~4,800 vaccinees that are selected into the subset of AEFI study at initial vaccination campaign;
- The vaccinees not in the subset but reported AEFI during the study follow-up (including all vaccination campaigns).
- The vaccinees with recorded SAEs within 6 months of vaccination

6.1.2 Statistical analysis

Counts and percentages of each local and systemic solicited adverse event will be presented for each vaccine arm among the ~5,000 actively followed up vaccinees (the combination of pilot study and the subset of vaccinees in main study), and the vaccinees not in the subset of ~4,800 vaccinees in the main study, separately. The reason is that the AEFIs in the latter group are self-reported and the AEFIs in the former group are actively collected by the trial team.

SAE will be coded using MedDRA by a blinded medical coder. Counts and percentages of SAEs will be presented by MedDRA system organ class and preferred term for each group separately and overall.

Data will also be presented by severity, expectedness, and relatedness to study medication.

6.2 Protection by Typhoid Vaccine against Suspected Typhoid and Clinical Typhoid

The outcomes covered in this section includes:

a. Rates of participants with a history of ≥2 days of persistent fever, and/or a temperature of ≥38 degrees C upon presentation, together with abdominal discomfort, in **vaccinees** in intervention clusters compared to control clusters.
b. Rates of participants with a history of ≥2 days of persistent fever, and/or a temperature of ≥38 degrees C upon presentation, together with abdominal discomfort, among **all residents** of the Vi-TCV clusters compared to the control vaccine clusters
c. Rates of patients with clinical diagnoses of typhoid fever in **vaccinees** in intervention clusters compared to control clusters
d. Rates of patients with clinical diagnoses of typhoid fever **among all residents** of the Vi-TCV clusters compared to the control vaccine clusters

6.2.1 Population and typhoid episodes for analysis

The analyses in vaccinees follow the population definition for total vaccine protection, while the analyses in all residents follow the population definition for overall vaccine protection, see section 5.1. For the analysis of outcome ***a*** and ***b***, we will count a febrile episode as a suspected typhoid fever episode, if the person had a history of ≥2 days of persistent fever,

and/or a temperature of ≥38 degrees C upon presentation, together with abdominal discomfort for at least one visit in that episode. The date of fever onset for the first visit of a suspected typhoid fever episode will define the date of onset of that suspected typhoid fever episode. For outcome ***c*** and ***d,*** febrile episodes in which a clinical typhoid diagnosis was made for at least one visit will be defined as clinical typhoid fever episodes, and the onset date will be the date of fever for the first visit.

6.2.2 Statistical analysis

The statistical analysis will follow section ***5.2***.

6.2.3 Subgroup analyses

The subgroup analyses in vaccinees and all residents will follow ***5.3***.

6.2.4 Sensitivity analyses

We will use alternative definitions for suspected typhoid to carry out sensitivity analyses, including:

- Fever ≥2 days together with abdominal discomfort identified at all study surveillance sites or hospitals, or identified during 6-monthly follow-ups (vaccinees only)
- Fever ≥3 days with or without abdominal discomfort identified at all study surveillance sites or hospitals, or identified during 6-monthly follow-ups (vaccinees only)

6.3 Total Protection by Typhoid Vaccine against Paratyphoid Fever

The incidence of blood culture confirmed paratyphoid fever in vaccinees in intervention clusters compared to control clusters

6.3.1 Population for analysis and paratyphoid episodes for analysis

The population for analysis will be the same vaccinees population as primary analysis, see section ***5.1***. Febrile episodes in which at least one blood culture was positive for *S. paratyphi* will be defined as paratyphoid fever episodes, and the date of fever onset for the first visit of a paratyphoid fever episode will define the date of onset of that paratyphoid fever episode. Episodes of paratyphoid fever will be counted if the onset of the episode, begins one or more days after vaccination.

6.3.2 Statistical analysis

The statistical analysis will follow section ***5.2***.

6.4 Indirect Protection by Typhoid Vaccine against Typhoid

The incidence of blood culture confirmed typhoid fever in non-vaccinees in intervention clusters will be compared to control clusters

6.4.1 Population for analysis

The population for this analyses will be persons present at baseline and persons who enter the clusters post-baseline via birth or in-migration from the outside (not from another cluster). We will exclude from the analysis persons who are vaccinated, from the time of their vaccination. Follow-up of this eligible, non-vaccinated population will begin at the "date of residence", and will end when, in the same cluster, death, migration out, loss of follow up, vaccination, or 18-month census occurs, whichever comes first. As such, the population under analysis for indirect vaccine protection will be a subset of the population analysed for overall vaccine protection.

6.4.2 Statistical analysis

The statistical analysis will follow section ***5.2***.

6.4.3 Subgroup analyses

The subgroup analyses will follow ***5.3***. in below subgroups:

- Ward (ward 2, ward 3 and ward 5)
- Age at "date of residence" (< 2 years, 2-4 years, 5-15 years and ≥ 16 years)
- Gender (male and female)
- Socioeconomic status at "date of residence" (quartiles)
- Household toilet type at "date of residence"
- Household source of drinking water at "date of residence"
- Household type of drinking water at "date of residence" (treated and not treated)
- Hand wash before meal at "date of residence" (with soap and without soap)
- Hand wash after defecation at "date of residence" (with and without soap/ash/soil)

6.5 Vaccine Protection among the Inner 80% Cluster Residents

The linear distance to the nearest cluster perimeter for every household will be measured by the geographic information system (GIS) mapping team. We will include the 80% households within each cluster defined as their distance among the furthest 80% to the nearest cluster perimeter.

The population for the analyses will be persons present at baseline in the inner clusters, and persons who enter the inner clusters post-baseline via birth or in-migration from the outside

(not from another cluster or the outer of the same cluster). The “date of residence” to calculate the follow-up time for analyses in residents will be defined as median date of baseline vaccination campaign (30 April 2018) for non-vaccinees living in the inner clusters before the start of the trial, or date of vaccination for those living in the inner clusters and vaccinated at the baseline vaccination campaign, or date of first migration into or birth into the inner clusters for those moved or born into the inner clusters post-baseline. Follow-up of this population will end when, in the same inner cluster, death, migration out, loss of follow up, or 18-month census occurs, whichever comes first. Analyses in vaccinees will follow the subset of this population from the time of vaccination (either at baseline or at catch-up vaccination) until the termination of follow-up, as just defined. The follow up of the rest of inner residents, after excluding the follow-up as vaccinees, will define the analysis population in non-vaccinees.

We will carry out the below analyses in the inner population:

- Total, indirect and overall vaccine protection against blood culture proven typhoid
- Total, indirect and overall vaccine protection against suspected typhoid and clinical typhoid

7 EXPLORATORY ANALYSES

7.1 Hospitalisation

Incidence of all cause hospitalisation in community-based surveys of all residents of the Vi-TCV clusters compared to the control vaccine clusters

7.1.1 Population for analysis

The population for analysis will be all residents in the whole cluster defined in section ***5.1***.

7.1.2 Statistical analysis

The incidence rates of all cause hospitalisation be estimated as the number of hospitalisation by the total number of person-years of follow-up. We will count all hospitalisations recorded at weekly hospitalisation visits, passive surveillance, 6-month vaccine follow-up and the community census. The follow-up time will be computed according to ***5.1***. The statistical model to estimate IRR will follow ***5.2***. In the case the data is over- dispersed, i.e. variance >> mean, we will use zero-inflated Poisson or zero-inflated negative binomial model depending on the variability and distribution of the data.

7.2 Immunogenicity

a. Assay of anti-Vi antibodies in blood samples collected at baseline (Day 0), at one month (Day 28), in a subset of participants from each treatment arm
b. Assay of anti-Vi IgG antibodies in blood samples collected at baseline (day 0), 18 months (day 545) and two years (day 730) in a subset of participants receiving intervention and control vaccinations

7.2.1 Population for analysis

The population for immunogenicity analysis will be the ~1,500 vaccinees that are selected into the subset of immunogenicity study in 18 clusters. Only those participants with Vi-IgG results available will be included in the analysis.

7.2.2 Statistical analysis

Data will be log-transformed prior to analysis. At each time point the number of samples below the lower limit of quantification will be summarised. The geometric mean concentration of anti-Vi IgG and associated 95% confidence interval will be summarised for each group at each visit, by computing the anti-log of the mean difference of the log-transformed data. Comparisons between groups will be made using Mann-Whitney U Test.

We will study the association between immunogenicity and vaccination using mixed effects regression model, adjusting for covariates. Estimates of anti-Vi IgG with the corresponding 95% confidence intervals (CI) will be delivered from the model at different time points to compare the antibody response between the two arms. Interactions with time of samples will be included in the models for each comparison as appropriate (e.g. time x vaccination). The results from the trial will be presented as comparative summary statistics (difference in log_{10}GMC) with 95% CIs, which will be calculated as the mean of log_{10}GMC in TCV arm compared with that in JE arm. Two levels of random effects (cluster level and individual level) will be included in the model, while age and sex will be included as fix effects. An unstructured correlation matrix will be used to model the within-participant error correlation structure. We will also perform various sensitivity analyses, e.g. multiple imputation, to test whether the results are robust to different assumptions about the missing data.

In the situation that normal transformation cannot be rendered by appropriate transformation, e.g. high proportion of samples below the lower limit of quantification, the data will be dichotomised by the lower limit of quantification, and mixed effects logistic regression will be used following the same analysis strategy.

7.3 Absenteeism

a. Incidence of absenteeism from school/work in **vaccinated children** and their guardians as a result of confirmed typhoid infection in intervention clusters compared to control clusters
b. Incidence of absenteeism from school/work in **all cluster residents** and their guardians/carers (if applicable) as a result of culture confirmed typhoid infection in intervention clusters compared to control clusters

7.3.1 Population for analysis

The analysis in vaccinees follows the population definition for total vaccine protection, while that in all residents follows the population definition for overall vaccine protection, see section ***5.1***.

7.3.2 Statistical analysis

The incidence rates of absenteeism will be estimated as the number of vaccinees/residents with any duration of absenteeism from school/work by the total number of person-years of follow-up, defined in section ***5.1***. The statistical analysis will follow section ***5.2***.

The number of days absent from school/work will be summarised for each arm and overall. If the data are normally distributed, then a t-test will be used to compare the two arms, otherwise a Mann Whitney U test will be used. If the number of vaccinees/residents with any duration of absenteeism is ≤ 20, the analyses will be descriptive in nature and no hypothesis

testing will be carried out. We will further explore the effect of vaccination on the days of absenteeism by using zero-inflated Poisson regression.

7.4 Mortality

a. Rates of overall and cause-specific mortality, recorded from hospital records and verbal autopsies via six-monthly census updates, in **vaccinees** in intervention clusters compared to control clusters
b. Rates of overall and cause-specific mortality, recorded from hospital records via six-monthly census updates, in **all residents** of the Vi-TCV clusters compared to the control vaccine clusters

7.4.1 Population for analysis

The analysis in vaccinees follows the population definition for total vaccine protection, while that in all residents follows the population definition for overall vaccine protection, see section ***5.1***.

7.4.2 Statistical analysis

The incidence rates of mortality will be estimated as the number of death in vaccinees/residents by the total number of person-years of follow-up, defined in section ***5.1***. The statistical analysis will follow section ***5.2***. Further survival analyses will also be carried out using Cox regression model.

7.5 Abdominal surgery

a. Rates of acute abdominal surgery, and of surgery for intestinal perforation, in **vaccinees** in intervention clusters compared to control clusters.
b. Rates of acute abdominal surgery, and of surgery for intestinal perforation, in **all residents** of the Vi-TCV clusters compared to the control vaccine clusters

7.5.1 Population for analysis

The analysis in vaccinees follows the population definition for total vaccine protection, while that in all residents follows the population definition for overall vaccine protection, see section ***5.1***.

7.5.2 Statistical analysis

The incidence rates of mortality will be estimated as the number of abdominal surgeries in vaccinees/residents by the total number of person-years of follow-up, defined in section ***5.1***. The statistical analysis will follow section ***5.2***.

7.6 Vaccine Protection by Different Size of Inner Clusters

If the transmission of typhoid happens from JE clusters into the TCV clusters, the herd protection can be different between the innermost and outermost populations. We will further explore this hypothesis by changing the inner cluster size from 80% (Section ***6.5***) to 50% and 25%, respectively. We will follow section ***6.5*** to redefine the inner cluster household so that the inner clusters cover 50% and 25% population for each cluster, respectively. All the analysis will follow section ***6.5.***

8 REFERENCE

1. Clemens J, Shin S, Ali M. New approaches to the assessment of vaccine herd protection in clinical trials. Lancet Infect Dis [Internet]. 2011 Jun 1;11(6):482–7.
